# Stereodivergent Synthesis of Camphor-Derived Diamines and Their Application as Thiourea Organocatalysts

**DOI:** 10.3390/molecules25132978

**Published:** 2020-06-29

**Authors:** Sebastijan Ričko, Franc Požgan, Bogdan Štefane, Jurij Svete, Amalija Golobič, Uroš Grošelj

**Affiliations:** Faculty of Chemistry and Chemical Technology, University of Ljubljana, Večna pot 113, 1000 Ljubljana, Slovenia; sebastijan.ricko@fkkt.uni-lj.si (S.R.); franc.pozgan@fkkt.uni-lj.si (F.P.); bogdan.stefane@fkkt.uni-lj.si (B.Š.); jurij.svete@fkkt.uni-lj.si (J.S.); amalija.golobic@fkkt.uni-lj.si (A.G.)

**Keywords:** (+)-camphor, diamines, thiourea, bifunctional organocatalysts, asymmetric synthesis

## Abstract

A series of 18 regio- and stereo-chemically diverse chiral non-racemic 1,2-, 1,3-, and 1,4-diamines have been synthesized from commercial (1*S*)-(+)-ketopinic acid and (1*S*)-(+)-10-camphorsulfonic acid. The structures of the diamines are all based on the d-(+)-camphor scaffold and feature isomeric diversity in terms of regioisomeric attachment of the primary and the tertiary amine function and the *exo/endo*-isomerism. Diamines were transformed into the corresponding noncovalent bifunctional thiourea organocatalysts, which have been evaluated for catalytic activity in the conjugative addition of 1,3-dicarbonyl nucleophiles (dimethyl malonate, acetylacetone, and dibenzoylmethane) to *trans*-β-nitrostyrene. The highest enantioselectivity was achieved in the reaction with acetylacetone as nucleophile using *endo*-1,3-diamine derived catalyst **52** (91.5:8.5 er). All new organocatalysts **48**–**63** have been fully characterized. The structures and the absolute configurations of eight intermediates and thiourea derivative **52** were also determined by X-ray diffraction.

## 1. Introduction

**Figure 1:**
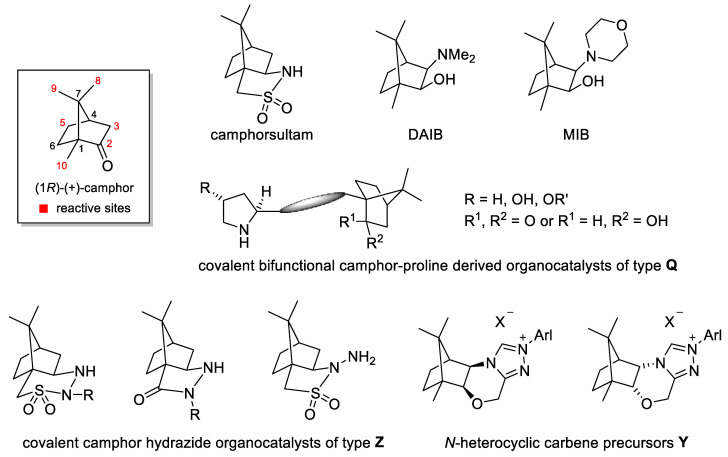
Selected examples of successful camphor-derivatives applied in asymmetric synthesis and catalysis.

Camphor is a privileged chiral pool building block available in both enantiomeric forms. More importantly, camphor undergoes a wide array of different chemical transformations including fragmentation reactions and sigmatropic rearrangements such as the Wagner-Meerwein rearrangement [1,2], which functionalizes, at first glance, inactivated positions (Figure 1), thus enabling the preparation of structurally and functionally very diverse products [3,4], including natural product paclitaxel (Taxol) [5,6].

In the field of asymmetric synthesis and catalysis, camphor derivatives have found their application as efficient chiral auxiliaries i.e. camphorsultam [7,8] and as ligands in metal-catalyzed reactions, as exemplified by DAIB [9] and MIB [10] used for the addition of organozinc reagents to aldehydes. In the field of asymmetric organocatalysis [11,12], camphor-derived organocatalysts [13,14] appeared in 2005 [15], shortly after the first seminal works at the turn of the millennium reported by List, Barbas, and Lerner (for enamine catalysis) [16], MacMillan (for iminium catalysis) [17], Jacobsen [18,19] and Takemoto [20] (for noncovalent bifunctional thiourea organocatalysis), and Enders and Kallfass (for *N*-heterocyclic carbene organocatalysis) [21]. With respect to the structure of camphor-derived organocatalysts, they can be divided into two groups (Figure 1) [13]. The largest group of camphor-derived organocatalysts are mostly covalent bifunctional catalysts characterized by the two chiral fragments, i.e. the camphor framework, which is covalently connected through a suitable spacer (amide, sulfonamide, sulfonate, sulfide, sulfone, or amine linker) to a chiral pyrrolidine i.e. proline derivative or other chiral *α*-amino acid derivative as generalized by catalyst **Q** [22,23,24,25,26,27,28,29]. The minor group consists of catalysts with the camphor skeleton as the exclusive chiral framework. The typical representatives thereof are camphor-derived *N*-heterocyclic carbene precursors **Y** [30,31,32] and camphor-hydrazide derived organocatalysts **Z** [33,34,35,36,37,38,39] (Figure 1).

Within our continuing study on camphor-based diamines as potential organocatalyst scaffolds with camphor as the exclusive chiral framework, we previously reported on camphor C2-, C3-, and C8-derived 1,2-, 1,3-, and 1,4-diamines [40,41,42]. The corresponding noncovalent thiourea organocatalysts have been evaluated for the 1,4-addition of dimethyl malonate to *trans*-β-nitrostyrene. Reaction with 1,2- and 1,4-diamine-derived catalysts resulted in low enantioselectivity (er up to 61.5:38.5) [40], while reaction with 2-*endo*-3-*endo*-1,3-diamine-derived catalyst gave the addition product with moderate enantioselectivity (80:20 er) [42]. On the other hand, the camphor-1,3-diamine-derived squaramide organocatalyst was highly effective in conjugative additions of 1,3-dicarbonyls to *trans*-β-nitrostyrenes [43,44]. These preliminary results prompted us to further investigate the structure-activity relationship of camphor-derived bifunctional organocatalysts in order to expand the regio- and stereochemical space allocated to camphor-based 1,2-, 1,3-, and 1,4-diamines and to probe, if the activities of the respective thiourea organocatalyst can match those of the established thiourea and squaramide noncovalent bifunctional organocatalysts [20,45,46,47,48,49,50,51,52,53,54]. Thus, synthetic access to the desired nonracemic diamines has to be provided in the first place. Herein, we report a stereodivergent synthesis of novel types of camphor-based 1,2-, 1,3-, and 1,4-diamines and the application of their thiourea derivatives as noncovalent bifunctional organocatalysts in Michael additions of dimethyl malonate and 1,3-diketones to *trans*-β-nitrostyrene, a typical model reaction for the evaluation of novel organocatalysts [20,45].

## 2. Results and Discussion

### 2.1. Synthesis of Camphor-Derived 1,2-Diamines

**Scheme 1:**
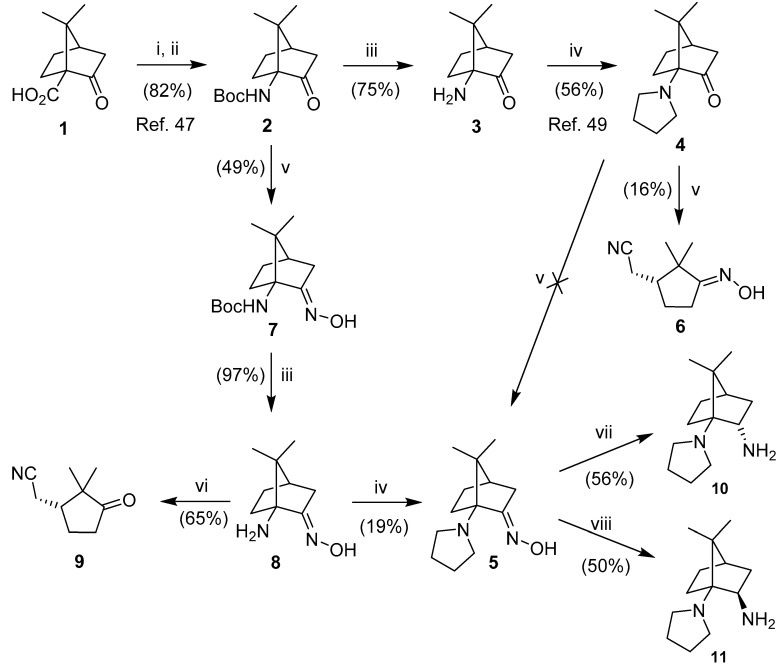
Synthesis of 1,2-diamines **10** and **11**. *Reaction conditions*: (**i**) DPPA, Et_3_N, toluene, reflux; (**ii**) *t*-BuOH, toluene, reflux; (**iii**) TFA, CH_2_Cl_2_, r.t.; (**iv**) 1,4-dibromobutane, K_2_CO_3_, MeCN, reflux; (**v**) NH_2_OH·HCl, pyridine, EtOH, reflux; (**vi**) 1,4-dibromobutane, K_2_CO_3_, H_2_O, μ-waves, 20 min, 125 °C, 100 W; (**vii**) Na, *n*-PrOH, reflux; (**viii**) Raney-Ni, H_2_, MeOH, r.t.

**Scheme 2:**
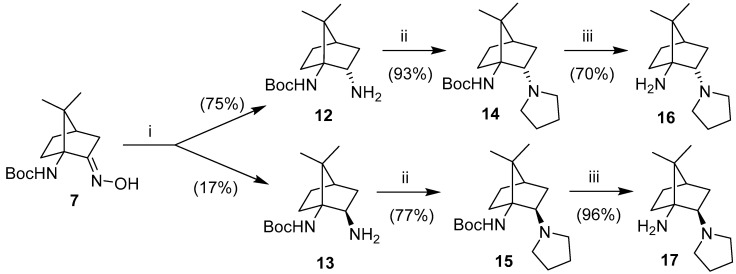
Synthesis of 1,2-diamines **16** and **17**. *Reaction conditions*: (**i**) Raney-Ni, H_2_, MeOH, r.t., then chromatographic separation; (**ii**) 1,4-dibromobutane, K_2_CO_3_, H_2_O, μ-waves, 20 min, 125 °C, 100 W; (**iii**) TFA, CH_2_Cl_2_, r.t.

Commercially available (1*S*)-(+)-ketopinic acid (**1**) was the common precursor for the synthesis of 1,2-diamines (Scheme 1 and Scheme 2) [55,56]. First, epimeric 1,2-diamines **10** and **11** with the primary amino function attached at position 2 and the tertiary amino function attached at position 1 were prepared. Curtius rearrangement of the acid **1** with diphenylphosphoryl azide (DPPA) followed by treatment with *t*-BuOH gave the Boc-protected amino ketone **2** [57], followed by removal of the Boc group to give the free amine **3** [58]. S_N_2-type cyclization of primary amine **3** with 1,4-dibromobutane gave pyrrolidino-ketone **4** [59]. Surprisingly, subsequent oximation of **4** with hydroxylamine failed to give the desired oxime **5**; instead, Grob fragmentation [60] to nitrile **6** [61] took place. Retracting to Boc-protected amino ketone **2** and effecting the oximation furnished the desired oxime **7**, which, upon amine deprotection and cyclization of amino-oxime **8** with 1,4-dibromobutane in acetonitrile under thermal conditions furnished in the desired pyrrolidino-oxime **5**. Interestingly, cyclization of **8** under microwave conditions in water gave the Grob fragmentation product **9** [62]. Finally, reduction of oxime **5** with sodium in isopropanol furnished the *endo*-amine **10** [63], while catalytic hydrogenation in the presence of Raney-Ni gave the *exo*-amine **11** (Scheme 1).

For the preparation of regioisomeric 1,2-diamines with the primary amino function attached at position 1 and the tertiary amine at position 2, Boc-amino-oxime **7** was reduced by applying catalytic hydrogenation in the presence of Raney-Ni, which furnished a chromatographically separable major *endo*-amine **12** in 75% yield and the minor *exo*-amine **13** [64] in 17% yield. Cyclization with 1,4-dibromobutane gave the corresponding pyrrolidine derivatives **14** and **15** [64], followed by concomitant Boc-deprotection into respective free diamines **16** [63] and **17** [63] (Scheme 2).

### 2.2. Synthesis of Camphor Derived 1,3-Diamines

**Scheme 3:**
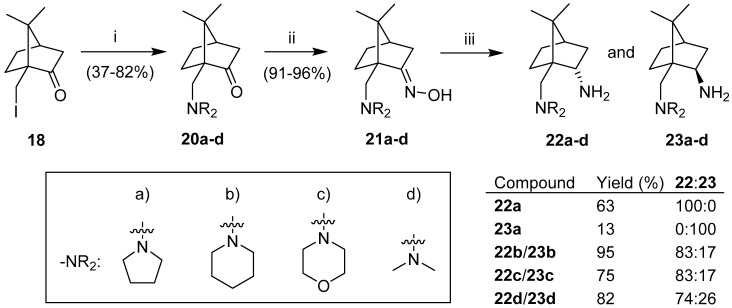
Synthesis of 1,3-diamines **22** and **23**. *Reaction conditions*: (**i**) secondary amine **19a**–**d**, K_2_CO_3_, DMSO, 110 °C; (**ii**) NH_2_OH·HCl, pyridine, EtOH, reflux; (**iii**) Na, *n*-PrOH, reflux, then chromatographic separation.

In this part of the study, we also first aimed to synthesize epimeric 1,3-diamines **22** and **23** with the primary amino function attached at position 2 and the tertiary amino function attached at position 10. In this context, Martínez et al. have previously developed a multistep synthesis of 10-(triflyloxy)camphor [65,66] to access 10-*N*-substituted camphor-derivatives. On the other hand, 10-iodocamphor (**18**) [43,67], available from commercial (1*S*)-(+)-10-camphorsulfonic acid in one step, turned out as a viable alternative in terms of large scale preparation and further transformations. 10-Iodocamphor (**18**) was used as a common precursor for the synthesis of camphor-based 1,3-diamines and the 1,4-diamines. In our hands, nucleophilic substitution of 10-iodocamphor (**18**) with secondary amines **19a**–**d** resulted in amino-ketones **20a**–**d** [65,68,69], which were, upon treatment with hydroxylamine, routinely transformed into oximes **21a**–**d**. Subsequent reduction of the oxime functionality with sodium in isopropanol gave epimeric mixtures of the *endo*-diamines **22a**–**d** and the *exo*-diamines **23a**–**d**, which were chromatographically separable only in the case of a mixture of epimeric pyrrolidine derivatives **22a** and **23a** [43] (Scheme 3). Mixtures of epimers **22b**–**d** and **23b**–**d** were used in further transformations into thiourea derivatives.

**Scheme 4:**
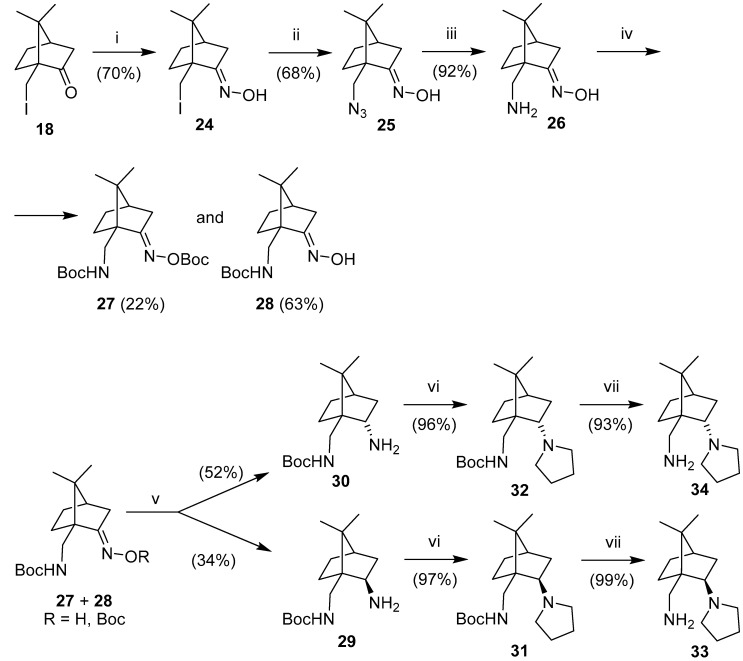
Synthesis of 1,3-diamines **33** and **34**. *Reaction conditions*: (**i**) NH_2_OH·HCl, pyridine, EtOH, reflux; (**ii**) NaN_3_, DMSO, 75 °C (**iii**) PPh_3_,THF, reflux, then H_2_O, reflux; (**iv**) Boc_2_O, Et_3_N, CH_2_Cl_2_, r.t.; (**v**) Raney-Ni, H_2_, MeOH, r.t., then chromatographic separation; (**vi**) 1,4-dibromobutane, K_2_CO_3_, H_2_O, μ-waves, 20 min, 125 °C, 100 W; (**vii**) TFA, CH_2_Cl_2_, r.t.

The regioisomeric 1,3-diamines with the primary amino function attached at position 10 and the tertiary amine at position 2 were prepared in seven steps starting from 10-iodocamphor (**18**) [43], which was initially transformed into iodo-oxime **24** [67], followed by the formation of azido-oxime **25** with sodium azide in DMSO. The route via phthalimide as a source of the amino group failed to give the desired primary amine [70]. Reduction of azide **25** with PPh_3_ gave amino-oxime **26**, which upon treatment with Boc_2_O furnished a chromatographically separable mixture of the minor *N*,*O*-diprotected product **27** and the major *N*-mono protected oxime **28**. Catalytic hydrogenation of either oxime **27** or **28**, or a mixture of oximes **27**/**28** with Raney-Ni furnished a chromatographically separable mixture of the minor *exo*-epimer **29** and the major *endo*-epimer **30**. Subsequent alkylation of amines **29** and **30** with 1,4-dibromobutane furnished pyrrolidines **31** and **32**, respectively, and the final TFA mediated *N*-Boc deprotection gave the respective diamines **33** and **34** (Scheme 4).

### 2.3. Synthesis of Camphor Derived 1,4-Diamines

**Scheme 5:**
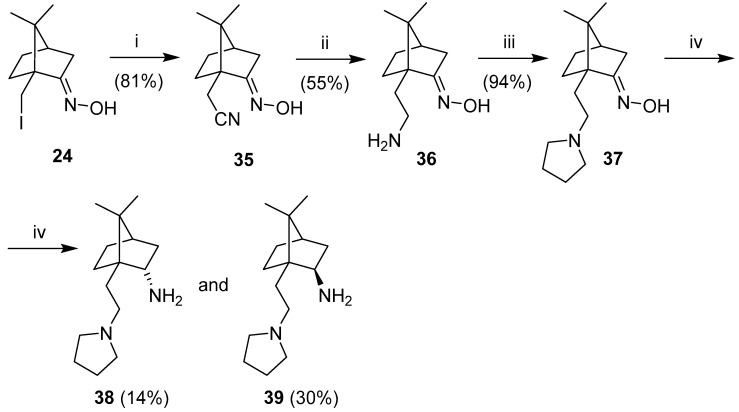
Synthesis of 1,4-diamines **38** and **39**. *Reaction conditions*: (**i**) KCN, DMSO, 75 °C, 16 h; (**ii**) LiAlH_4_, Et_2_O, r.t.; (**iii**) 1,4-dibromobutane, K_2_CO_3_, H_2_O, μ-waves, 20 min, 125 °C, 100 W; (**iv**) Raney-Ni, H_2_, MeOH, r.t., then chromatographic separation.

To prepare the desired 1,4-diamines, we followed the idea to introduce an additional methylene group at position 10 in 10-iodocamphor derivatives via cyanation followed by reduction. However, our attempt to prepare 10-cyanocamphor by treatment of 10-iodocamphor (**18**) with potassium cyanide in DMSO at 75 °C failed and gave only the Groß fragmentation products. On the other hand, treatment of 10-iodocamphor oxime (**24**) under the same conditions resulted in the desired nucleophilic displacement reaction to yield the corresponding nitrile **35**. For the following selective reduction of the cyano group of **35**, several reagents were tested i.e. various catalytic hydrogenations and hydride reducing agents. In most cases, mixtures of products were formed, while reduction with LiAlH_4_ furnished the desired amino oxime **36** as the central intermediate for the preparation of isomeric 1,4-diamines. Following previous examples, epimeric 1,4-diamines **38** and **39** with the primary amino function attached at position 2 were prepared first. Microwave promoted cyclization with 1,4-dibromobutane yielded pyrrolidino-oxime **37**, which upon catalytic hydrogenation with Raney-Ni furnished a chromatographically separable major *exo*-epimer **38** and minor *endo*-epimer **39** (Scheme 5).

**Scheme 6:**
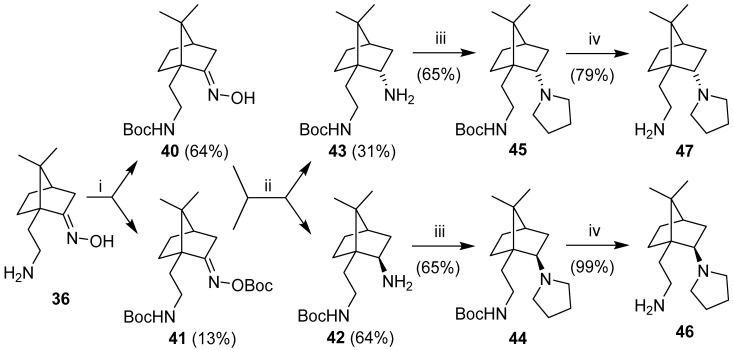
Synthesis of 1,4-diamines **47** and **48**. *Reaction conditions*: (**i**) Boc_2_O, Et_3_N, CH_2_Cl_2_, r.t., then chromatographic separation; (**ii**) Raney-Ni, H_2_, MeOH, r.t., then chromatographic separation; (**iii**) 1,4-dibromobutane, K_2_CO_3_, H_2_O, μ-waves, 20 min, 125 °C, 100 W; (**iv**) TFA, CH_2_Cl_2_, r.t.

The synthesis of epimeric 1,4-diamines with the tertiary amino function at position 2 also commenced from amino-oxime **36**. Boc protection of amino-oxime **36** gave a mixture of *N*-mono and *N*,*O*-diprotected oximes **40** and **41**, which could be separated by column chromatography. Catalytic hydrogenation of either oxime **40** or **41**, or a mixture of oximes **40**/**41** with Raney-Ni furnished a chromatographically separable major *exo*-epimer **42** and the minor *endo*-epimer **43**. The ensuing microwave mediated cyclization with 1,4-dibromobutane gave amino-pyrrolidines **44** [64] and **45**, respectively, while the final TFA mediated *N*-Boc deprotection furnished the corresponding diamines **46** [63] and **47** [63] (Scheme 6).

### 2.4. Synthesis of Noncovalent Thiourea Organocatalysts 48–63

**Figure 2:**
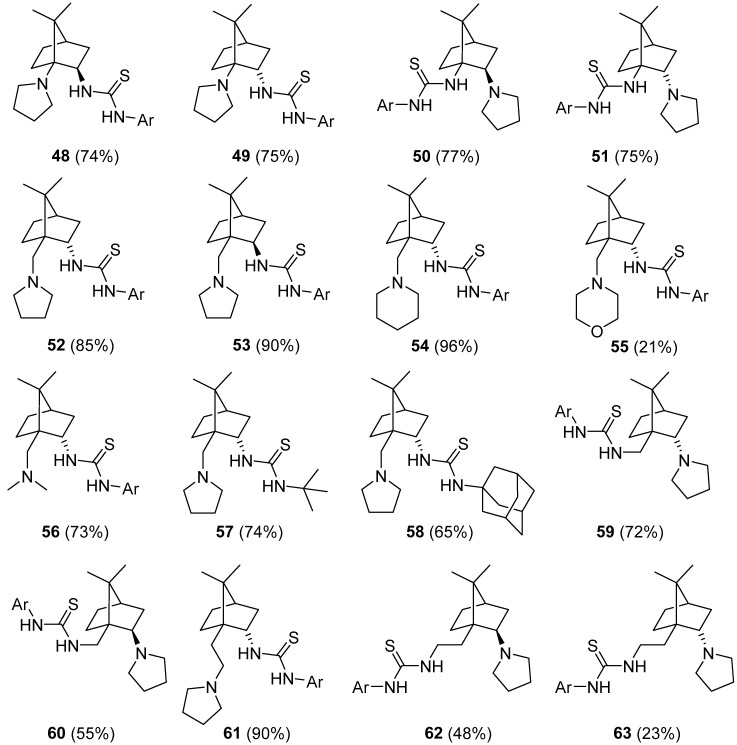
Thiourea derivatives **48**–**63** (Ar = 3,5-bis(trifluoromethyl)phenyl) prepared by treatment of respective primary amine with respective isothiocyanate in Et_2_O at r.t.

Noncovalent thiourea organocatalysts **48**–**63** have been prepared in 23–96% yields by treatment of primary amines **10**, **11**, **16**, **17**, **22a**–**d**, **23a**, **33**, **34**, **38**, **39**, **46**, and **47** with the corresponding isothiocyanate (1-adamantyl isothiocyanate, *tert*-butyl isothiocyanate, and 3,5-bis(trifluoromethyl)phenyl isothiocyanate) (Figure 2).

### 2.5. Structure Determination

**Figure 3:**
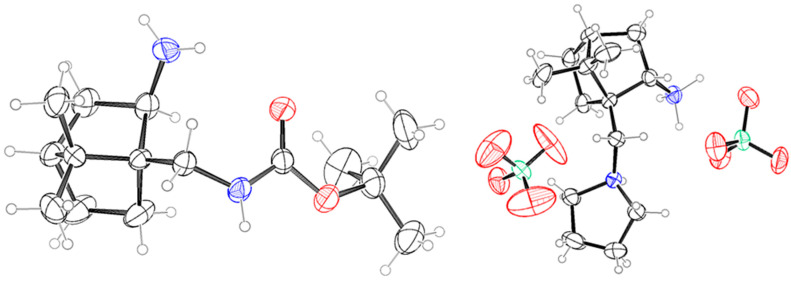
Single-crystal X-ray structures of *exo*-diamine **29** (**left**) and *endo*-diamine·**22a**·HClO_4_ (**right**).

Novel compounds were characterized by spectroscopic methods (^1^H and ^13^C-NMR, HRMS, IR) and by elemental analyses for C, H, and N. Compounds **10**, **13**, **15**, **16**, **17**, **22b**/**23b**, **41**, **44**, **46**, and **47** were not isolated pure form and were used for further transformations without purification. The structures of compounds **8**, **18**, **21a**, **22a**, **24**, **28**, **29**, **37**, and **52** were also determined by single crystal X-ray analysis (Figures S3–S11) [71]. X-ray structures of the representative diamines **22a** and **29** are shown in Figure 3.

**Figure 4:**
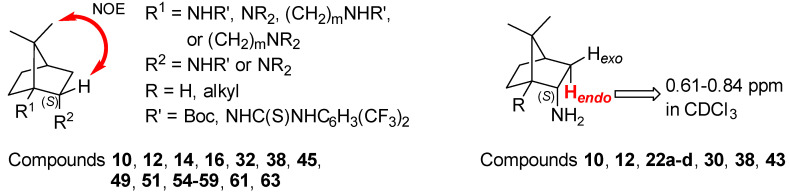
Determination of the absolute configuration at C-2 based on the observed NOE correlation spectroscopy cross peaks and chemical shift correlations in the series of primary *endo*-amines.

The configuration of the newly formed stereocenter at C-2 for the *endo*-isomers **10**, **12**, **14**, **16**, **32**, **38**, **45**, **49**, **51**, **54**–**59**, **61**, and **63** was also determined by 2D NOESY spectroscopy. NOE between the 2-H and the 8-Me group were in agreement with the (2*S*)-configuration (Figure 4) [71]. The (2*S*)-configuration of the *endo*-isomers **10**, **12**, **22a**–**d**, **30**, **38**, and **43** was additionally confirmed on the basis of chemical shift correlations of the highly up-field shifted *endo*-proton at position 3, which appears in the range of 0.61 ppm to 0.84 ppm (Figure 3). Interestingly, in all successful cases of chromatographic separation of the epimeric mixtures of primary amines, the *exo*-amine eluted first, followed by elution of the *endo*-amine.

### 2.6. Performance of Noncovalent Thiourea Organocatalysts 48–63 in 1,4-Addition of 1,3-Dicarbonyl Compounds to trans-β-Nitrostyrene

The model reaction for the evaluation of catalyst **48**–**63** efficiency was the 1,4-addition of dimethyl malonate to *trans*-β-nitrostyrene performed in anhydrous toluene at 25 °C and −25 °C for 2–3 days (Table 1, cf. Figure 2). The full conversion at 25 °C was achieved with the *endo*-1,3-diamine-based catalysts **52**, **56**, and **59** (Table 1, Entries 9, 16, and 20) and 1,4-diamine-based catalysts **62** and **63** (Table 1, Entries 25 and 27). At the lower temperature (−25 °C) only catalysts **56** and **63** gave full conversion (Entries 17 and 28), while all other catalysts displayed diminished conversion. Generally, the *endo*-catalysts achieved better conversions than the *exo*-epimers, except in the case of 1,2-diamine-based catalysts **50** and **51** (Entries 5 and 7). This was not surprising, since better performance of the *endo*-isomers in these 1,4-additions has also been observed previously with related camphor-based organocatalysts [42,43,44]. In terms of enantioselectivity, decreasing the reaction temperature resulted in either retention or diminished selectivity, except with catalysts **60** and **62** (Entries 22/23 and 25/26). As expected [43], the best enantioselectivity was achieved with the *endo*-1,3-diamine catalyst **52** (78.5:21.5 er (*R*), 100% conversion, Entry 9) followed by the *exo*-epimer **53** (77.5:22.5 er (*S*), Entry 11) though incomplete conversion (69%). In the *endo*-1,3-diamine type catalyst series **52**–**56**, catalyst **52** with the pyrrolidine functionality displayed the best performance (Entry 9), while catalyst **55** with the morpholine functionality performed the worst both in terms of conversion (10%) and selectivity (54.5:45.5 er) (Entry 15) (Table 1).

Finally, the 1,2-diamine-based catalysts **48** and **49** and the 1,3-diamine-based catalysts **52** and **53** were evaluated for the Michael addition of acetylacetone and dibenzoylmethane to *trans*-β-nitrostyrene in anhydrous toluene (Table 2). Surprisingly, the performance of *endo*-1,2-diamine derived catalyst **49** for the addition of acetylacetone to *trans*-β-nitrostyrene gave the product in full conversion and 80.5:19.5 er (*R*) (Entry 2). Expectedly, all reactions resulted in higher conversion and selectivity when catalyzed with *endo*-**52**, furnishing the corresponding acetylacetone adduct quantitatively at −25 °C with 91.5:8.5 er (*S*) (Entry 4), flanked by the epimer *exo*-**53** at the same temperature with complementary 82:18 er (*R*) though 63% conversion (Entry 9). Changing the solvent to CH_2_Cl_2_ or THF in the same reaction catalyzed with **52** resulted in diminished selectivity (Entries 5 and 6) (Table 2).

## 3. Materials and Methods

### 3.1. Materials and General Methods

Solvents for extractions and chromatography were of technical grade and were distilled prior to use. Extracts were dried over technical grade Na_2_SO_4_. Melting points were determined on a Kofler micro hot stage and on SRS OptiMelt MPA100–Automated Melting Point System (Stanford Research Systems, Sunnyvale, CA, USA). The NMR spectra were obtained on a Bruker UltraShield 500 plus (Bruker, Billerica, MA, USA) at 500 MHz for ^1^H and 126 MHz for ^13^C nucleus, using DMSO-*d*_6_ and CDCl_3_ with TMS as the internal standard, as solvents. Mass spectra were recorded on an Agilent 6224 Accurate Mass TOF LC/MS (Agilent Technologies, Santa Clara, CA, USA), IR spectra on a Perkin-Elmer Spectrum BX FTIR spectrophotometer (PerkinElmer, Waltham, MA, USA). Microanalyses were performed on a Perkin-Elmer CHN Analyzer 2400 II (PerkinElmer). Column chromatography (CC) was performed on silica gel (Silica gel 60, particle size: 0.035–0.070 mm (Sigma-Aldrich, St. Louis, MO, USA). HPLC analyses were performed on an Agilent 1260 Infinity LC (Agilent Technologies, Santa Clara, CA, USA) using CHIRALPAK AD-H (0.46 cm ø × 25 cm) and CHIRALCEL OD-H (0.46 cm ø × 25 cm) as chiral columns (Chiral Technologies, Inc., West Chester, PA, USA). Organocatalyzed reactions were performed on EasyMax 102 Advanced synthesis workstation (Mettler-Toledo, LLC., Columbus, OH, USA). Catalytic hydrogenation was performed on a Parr Pressure Reaction Hydrogenation Apparatus (Moline, IL, USA). The optical rotation of optical active substances was measured on a Perkin Elmer 241 MC Polarimeter (PerkinElmer, Waltham, MA, USA) equipped with a Na lamp (sodium emission lines at 589.0 nm) at 20 °C. 

The compounds (1*S*,4*R*)-7,7-dimethyl-1-(pyrrolidin-1-ylmethyl)bicyclo[2.2.1]heptan-2-one (**20a**), (1*S*,4*R*,2*E*)-7,7-dimethyl-1-(pyrrolidin-1-ylmethyl)bicyclo[2.2.1]heptan-2-one oxime (**21a**), (1*S*,2*S*,4*R*)-7,7-dimethyl-1-(pyrrolidin-1-ylmethyl)bicyclo[2.2.1]heptan-2-amine (**22a**), and (1*S*,2*R*,4*R*)-7,7-dimethyl-1-(pyrrolidin-1-ylmethyl)bicyclo[2.2.1]heptan-2-amine (**23a**) [43] were prepared following the referenced literature procedures. Racemic products of 1,4-addition of *trans*-β-nitrostyrene to dimethyl malonate [40,42], acetylacetone [43], and dibenzoylmethane [43], used as standards for determination of er by HPLC, were prepared following the corresponding literature procedures.

### 3.2. General Procedures

#### 3.2.1. General Procedure 1. Synthesis of Free Diamines by Deprotection of *N*-Boc-Amines

To a solution of Boc-protected amine in anhydrous CH_2_Cl_2_ at room temperature was, under stirring, slowly added the same volume of anhydrous trifluoroacetic acid. The resulting reaction mixture was stirred at room temperature for 16 h. Volatile components were evaporated in vacuo. The residue was dissolved in Et_2_O and washed with NaOH (1 M in H_2_O, 1/5 of the volume of Et_2_O) and NaCl (aq. sat., 1/5 of the volume of Et_2_O). The organic phase was dried over anhydrous Na_2_SO_4_, filtered, and volatile components evaporated in vacuo. If necessary, the so obtained product was purified by CC. Fractions containing the pure product were combined and volatile components evaporated in vacuo to give the corresponding amine [71].

#### 3.2.2. General Procedure 2. Synthesis of Oximes

For the oxime synthesis, a modified procedure from the literature was applied [72]. To a solution of ketone (1 equiv.) in anhydrous EtOH, NH_2_OH·HCl (2 equiv.) and pyridine (1.5 equiv.) were added, and the resulting reaction mixture was stirred under reflux for 6–16 h. Volatile components were evaporated in vacuo, the residue was suspended in H_2_O (15 mL) followed by the addition of finely powdered NaOH till pH ≈ 10–12. The resulting mixture was extracted with Et_2_O (3 × 50 mL). The combined organic phase was washed with H_2_O (1/5 of the volume of Et_2_O) and NaCl (aq. sat., 1/5 of the volume of Et_2_O). The organic phase was dried over anhydrous Na_2_SO_4_, filtered, and volatile components evaporated in vacuo. If necessary, the so obtained oxime was purified by CC. Fractions containing the pure product were combined and volatile components evaporated in vacuo to give the corresponding oxime [71].

#### 3.2.3. General Procedure 3. Synthesis of Tertiary Amines (Pyrrolidines) by Cyclative Bis-Alkylation of Primary Amines

For the alkylation of primary amines, a modified procedure from the literature was applied [73]. To a suspension of amine (1 equiv.) in H_2_O (2 mL), K_2_CO_3_ (1.1 equiv.) and 1,4-dibromobutane (1.1 equiv.) were added, and the resulting reaction mixture was stirred under microwave irradiation (MW) for 20 min (100 W, 125 °C, ~5 bar). The reaction mixture was extracted with EtOAc (3 × 25 mL). The combined organic phase was washed with NaCl (aq. sat., 1/5 of the volume of EtOAc). The organic phase was dried over anhydrous Na_2_SO_4_, filtered, and volatile components evaporated in vacuo. If necessary, the so obtained tertiary amine was purified by CC. Fractions containing the pure product were combined and volatile components evaporated in vacuo to give the corresponding tertiary amine [71].

#### 3.2.4. General Procedure 4. Synthesis of Primary Amines by Reduction of Oximes with Sodium

To a solution of oxime in anhydrous *n*-PrOH under argon at 95 °C, sodium (ca. 100–200 mg) was added. Before all the added sodium reacted, another chunk of sodium (ca. 100–200 mg) was added, followed by addition of further sodium to ensure a continuous evolution of hydrogen for 1 h. After all the sodium reacted, volatile components were evaporated in vacuo, and to the residue, H_2_O was added followed by extraction with Et_2_O (5 × 25 mL). The combined organic phase was washed with H_2_O (1/5 of the volume of Et_2_O) and NaCl (aq. sat., 1/5 of the volume of Et_2_O). The organic phase was dried over anhydrous Na_2_SO_4_, filtered, and volatile components evaporated in vacuo. If necessary, the so obtained amine was purified/separated by CC. Fractions containing the pure product were combined and volatile components evaporated in vacuo to give the corresponding amine [71].

#### 3.2.5. General Procedure 5. Synthesis of Primary Amines by Hydrogenation of Oximes in the Presence of Raney-Ni Catalyst

Raney-Ni (ca. 100–200 mg) was added to a solution of oxime and triethylamine in MeOH under Argon. The reaction vessel was thoroughly flushed with hydrogen and the reaction mixture was hydrogenated in a Parr shaker hydrogenation apparatus in the atmosphere of hydrogen (60 psi) at room temperature for 6 h. The reaction mixture was filtered through a plague of Celite^®^ and washed with MeOH. Volatile components were evaporated in vacuo. The residue was dissolved in Et_2_O (100 mL) and washed with H_2_O (1/10 of the volume of Et_2_O) and NaCl (aq. sat., 1/10 of the volume of Et_2_O). If the residue after filtration through a plague of Celite^®^ was of green color, due to the presence of nickel species, the residue was dissolved in Et_2_O (100 mL) and washed consecutive with NH_4_OH (25% aq., 1/10 of the volume of Et_2_O) till the disappearance of the green color (the aqueous phase turns violet) and finally NaCl (aq. sat., 1/10 of the volume of Et_2_O). The organic phase was dried over anhydrous Na_2_SO_4_, filtered, and volatile components evaporated in vacuo. If necessary, the so obtained amine was purified/separated by CC. Fractions containing the pure product were combined and volatile components evaporated in vacuo to give the corresponding amine [71].

#### 3.2.6. General Procedure 6. Synthesis of Thiourea Derivatives

To a solution of amine (1 equiv.) in anhydrous Et_2_O under Argon at 0 °C isothiocyanate (0.95 equiv.) was added. The resulting reaction mixture was stirred at 0 °C for 30 min and at room temperature for 24 h. Volatile components were evaporated in vacuo and the residue was purified by CC. Fractions containing the pure product were combined and volatile components evaporated in vacuo to give the corresponding thiourea derivative [71].

#### 3.2.7. General Procedure 7. Synthesis of *N*-Boc Protected Primary Amines

To a solution of amine (1 equiv.) in anhydrous CH_2_Cl_2_ under Argon at room temperature Boc_2_O (1.5 equiv.) and Et_3_N (2 equiv.) were added. The resulting reaction mixture was stirred at room temperature for 16 h. Volatile components were evaporated in vacuo and the residue was purified/separated by CC. Fractions containing the pure product were combined and volatile components evaporated in vacuo to give the corresponding *N*-Boc protected amine [71].

#### 3.2.8. General Procedure 8. Synthesis of Tertiary Amines by Amination of 10-Iodocamphor (18)

To a suspension of 10-iodocamphor (**18**) (1 equiv.) and K_2_CO_3_ (1.5 equiv.) in anhydrous DMSO under Argon secondary amine (15 equiv.) was added, and the resulting reaction mixture was stirred at 110 °C for 16 h. The addition of the secondary amine is accompanied with an intense blue or violet coloration, which eventually fades away. The cooled reaction mixture was diluted with H_2_O (ca. 10 mL of H_2_O per 1 mL DMSO) and extracted with EtOAc (ca. 3 × (15 mL EtOAc per 10 mL H_2_O)). The combined organic phase was washed with H_2_O (1/5 of the volume of EtOAc) and NaCl (aq. sat., 1/5 of the volume of EtOAc). The organic phase was dried over anhydrous Na_2_SO_4_, filtered, and volatile components evaporated in vacuo. If necessary, the so obtained tertiary amino-ketone was purified/separated by CC. Fractions containing the pure product were combined and volatile components evaporated in vacuo to give the corresponding tertiary amine [71].

#### 3.2.9. General Procedure 10. Testing the Catalytic Activity of Thiourea Derivatives in 1,4-Additions of 1,3-Dicarbonyl Compounds to *trans*-β-Nitrostyrene

Dimethyl malonate (92 μL, 0.8 mmol) or acetylacetone (82 μL, 0.8 mmol) or dibenzoylmethane (180 mg, 0.8 mmol) was added to a solution of *trans*-β-nitrostyrene (60 mg, 0.4 mmol) and thiourea organocatalyst **48**–**63** (10 mol%, relative to *trans*-β-nitrostyrene) in anhydrous toluene, CH_2_Cl_2_, or THF (1 mL) under argon at 25 or −25 °C. The resulting reaction mixture was stirred at 25 or −25 °C for 48–72 h. The reaction mixture was then quickly passed through a short column filled with Silica gel 60 (1 cm diameter, 5 cm length) using a mixture of EtOAc and petroleum ether in a 1:1 ratio as a mobile phase to remove the tested organocatalyst **48**–**63**. Volatile components were evaporated in vacuo and the residue was used to determine the conversion by ^1^H-NMR and enantioselectivity by HPLC.

### 3.3. General Procedures

#### 3.3.1. Synthesis of 1-[3,5-Bis(trifluoromethyl)phenyl]-3-[(1*S*,2*R*,4*R*)-7,7-dimethyl-1-(pyrrolidin-1-yl)-bicyclo[2.2.1]heptan-2-yl]thiourea (**48**)

Following General Procedure 6 the title compound was prepared from diamine **11** (96 mg, 0.461 mmol), Et_2_O (3 mL), 1-isothiocyanato-3,5-bis(trifluoromethyl)benzene (82 µL, 0.44 mmol, 98%); purified by CC (Et_3_N:EtOAc:petroleum ether = 1:1:20). Yield: 164 mg (0.341 mmol, 74%) of colorless solid; mp = 171–176 °C.

$${\left[\alpha \right]}_{D}^{20}$$

= −7.7 (c = 0.34, CH_2_Cl_2_). CHN analysis for C_22_H_27_F_6_N_3_S requires: C, 55.10; H, 5.68; N, 8.76 and found: C, 55.07; H, 5.73; N, 8.57. EI-HRMS: *m/z* = 480.1899 (MH^+^); C_22_H_28_F_6_N_3_S requires: *m/z* = 480.1903 (MH^+^). *ν*_max_ 3133, 2965, 2876, 1620, 1503, 1462, 1371, 1273, 1175, 1139, 1108, 947, 876, 847, 808, 797, 735, 704, 681, 645, 624 cm^−1^. ^1^H-NMR (500 MHz, DMSO-*d*_6_): *δ* 1.05 (*s*, 3H, Me); 1.07 (*s*, 3H, Me); 1.16–1.30 (*m*, 2H); 1.50–1.58 (*m*, 1H); 1.60–1.70 (*m*, 4H); 1.72–1.92 (*m*, 3H); 1.94–2.02 (*m*, 1H); 2.44–2.60 (*m*, 4H); 3.87–3.98 (*m*, 1H); 7.72 (*s*, 1H, 1H of Ar); 7.99 (*d*, *J* = 4.1 Hz, 1H, NH); 8.40 (*s*, 2H, 2H of Ar); 10.67 (br *s*, 1H, NH). ^13^C-NMR (126 MHz, DMSO-*d*_6_): *δ* 20.0, 21.5, 23.0, 23.1, 25.8, 39.3, 45.1, 46.9, 47.4, 60.5, 69.4, 115.7, 120.9, 123.3 (*q*, *J* = 272.8 Hz), 130.2 (*q*, *J* = 32.7 Hz), 142.0, 178.2. 

#### 3.3.2. Synthesis of 1-(3,5-Bis(trifluoromethyl)phenyl)-3-((1*S*,2*S*,4*R*)-7,7-dimethyl-1-(pyrrolidin-1-yl)-bicyclo[2.2.1]heptan-2-yl)thiourea (**49**)

Following General Procedure 6 compound **49** was prepared from diamine **10** (95 mg, 0.456 mmol), Et_2_O (5 mL), 1-isothiocyanato-3,5-bis(trifluoromethyl)benzene (80 µL, 0.43 mmol, 98%); purified by CC (1. Et_3_N:EtOAc:petroleum ether = 1:1:10 for the elution of nonpolar impurities; 2. Et_3_N:EtOAc:petroleum ether = 1:1:1 for the elution of product **49**). Yield: 164 mg (0.342 mmol, 75%) of colorless solid; mp = 59–64 °C.

$${\left[\alpha \right]}_{D}^{20}$$

= –35.5 (c = 0.25, CH_2_Cl_2_). CHN analysis for C_22_H_27_F_6_N_3_S requires: C, 55.10; H, 5.68; N, 8.76 and found: C, 54.34; H, 5.78; N, 8.53. EI-HRMS: *m/z* = 480.1898 (MH^+^); C_22_H_28_F_6_N_3_S requires: *m/z* = 480.1903 (MH^+^). *ν*_max_ 3251, 2960, 2879, 1610, 1537, 1512, 1470, 1380, 1348, 1274, 1170, 1125, 993, 964, 884, 847, 809, 755, 725, 701, 680 cm^−1^. ^1^H-NMR (500 MHz, DMSO-*d*_6_): *δ* 0.95–1.01 (*m*, 1H); 0.98 (*s*, 3H, Me); 1.10 (*s*, 3H, Me); 1.29–1.36 (*m*, 1H); 1.43–1.49 (*m*, 1H); 1.54–1.61 (*m*, 4H); 1.79–2.02 (*m*, 3H); 2.31–2.42 (*m*, 1H); 2.72–2.85 (*m*, 4H); 5.18–5.27 (*m*, 1H); 7.70 (*s*, 1H, 1H of Ar); 8.28 (*d*, *J* = 9.1 Hz, 1H, NH); 8.34 (*s*, 2H, 2H of Ar); 10.03 (br *s*, 1H, NH). ^13^C-NMR (126 MHz, DMSO-*d*_6_): *δ* 20.8, 21.4, 23.7, 26.1, 27.1, 36.5, 39.9, 43.9, 47.6, 48.9, 51.6, 70.5, 115.6, 123.29 (*q*, *J* = 273 Hz), 130.15 (*q*, *J* = 33 Hz), 142.1, 179.0. 

#### 3.3.3. Synthesis of 1-(3,5-Bis(trifluoromethyl)phenyl)-3-((1*S*,2*R*,4*R*)-7,7-dimethyl-2-(pyrrolidin-1-yl)-bicyclo[2.2.1]heptan-1-yl)thiourea (**50**)

Prepared from diamine **17** (55 mg, 0.264 mmol), Et_2_O (2.5 mL), 1-isothiocyanato-3,5-bis(trifluoromethyl)benzene (63 µL, 0.340 mmol, 98%) following General Procedure 6. Purified by CC (Et_3_N:EtOAc:petroleum ether = 1:1:30). Yield: 98 mg (0.203 mmol, 77%) of colorless solid; mp = 123–126 °C.

$${\left[\alpha \right]}_{D}^{20}$$

= −13.3 (c = 0.32, CH_2_Cl_2_). EI-HRMS: *m/z* = 480.1898 (MH^+^); C_22_H_28_F_6_N_3_S requires: *m/z* = 480.1903 (MH^+^). *ν*_max_ 2963, 1671, 1542, 1474, 1385, 1335, 1274, 1201, 1167, 1119, 1000, 973, 916, 880, 831, 799, 720, 698, 676 cm^−1^. ^1^H-NMR (500 MHz, DMSO-*d*_6_): *δ* 0.93 (*s*, 3H, Me); 1.16–1.22 (*m*, 1H); 1.30 (*s*, 3H, Me); 1.49–1.67 (*m*, 6H); 1.70–1.83 (*m*, 2H); 1.92–2.03 (*m*, 1H); 2.43–2.80 (*m*, 5H); 2.88–2.99 (*m*, 1H); 7.55 (br *s*, 1H, NH); 7.72 (*s*, 1H, 1H of Ar); 8.37 (*s*, 2H, 2H of Ar); 10.36 (br *s*, 1H). ^13^C-NMR (126 MHz, DMSO-*d*_6_): *δ* 19.2, 20.4, 23.0, 27.3, 33.7, 36.8, 41.4, 48.7, 51.8, 68.7, 69.2, 115.8, 121.2, 123.3 (*q*, *J* = 273 Hz), 130.1 (*q*, *J* = 33 Hz), 142.0, 180.9.

#### 3.3.4. Synthesis of 1-(3,5-Bis(trifluoromethyl)phenyl)-3-((1*S*,2*S*,4*R*)-7,7-dimethyl-2-(pyrrolidin-1-yl)-bicyclo[2.2.1]heptan-1-yl)thiourea (**51**)

Prepared following General Procedure 6 from diamine **16** (122 mg, 0.586 mmol), Et_2_O (2.5 mL), 1-isothiocyanato-3,5-bis(trifluoromethyl)benzene (106 µL, 0.570 mmol, 98%); purified by CC (1. Et_3_N:Et_2_O:petroleum ether = 1:6:25 for the elution of nonpolar impurities; 2. Et_3_N:Et_2_O = 1:5 for the elution of product **51**). Yield: 211 mg (0.440 mmol, 75%) of colorless solid; mp = 89–99 °C.

$${\left[\alpha \right]}_{D}^{20}$$

= −22.7 (c = 0.41, CH_2_Cl_2_). EI-HRMS: *m/z* = 480.1899 (MH^+^); C_22_H_28_F_6_N_3_S requires: *m/z* = 480.1903 (MH^+^). *ν*_max_ 3409, 2963, 2884, 2838, 1659, 1612, 1564, 1488, 1472, 1381, 1273, 1256, 1167, 1122, 979, 881, 847, 808, 774, 723, 700, 679 cm^−1^. ^1^H-NMR (500 MHz, DMSO-*d*_6_): *δ* 1.03 (*s*, 3H, Me); 1.12 (*s*, 3H, Me); 1.37–1.46 (*m*, 1H); 1.47–1.74 (*m*, 7H); 1.84–1.97 (*m*, 1H); 2.01–2.11 (*m*, 1H); 2.48–2.53 (*m*, 1H); 2.74–2.86 (*m*, 2H); 2.89–3.05 (*m*, 2H); 3.49–3.60 (*m*, 1H); 7.22 (br *s*, 1H, NH); 7.79 (*s*, 1H, 1H of Ar); 8.11 (*s*, 2H, 2H of Ar); 14.28 (br *s*, 1H, NH). ^13^C-NMR (126 MHz, DMSO-*d*_6_): *δ* 17.9, 18.5, 23.5, 25.8, 26.4, 27.2, 41.8, 49.1, 51.4, 66.3, 71.2, 116.8, 124.1, 123.27 (*q*, *J* = 272.8 Hz), 130.02 (*q*, *J* = 32.8 Hz), 143.0, 183.0. 

#### 3.3.5. Synthesis of 1-(3,5-Bis(trifluoromethyl)phenyl)-3-((1*R*,2*S*,4*R*)-7,7-dimethyl-1-(pyrrolidin-1-yl-methyl)bicyclo[2.2.1]heptan-2-yl)thiourea (**52**)

Using General Procedure 6 this substance was prepared from diamine **22a** (157 mg, 0.706 mmol), Et_2_O (3 mL), 1-isothiocyanato-3,5-bis(trifluoromethyl)benzene (125 µL, 0.670 mmol, 98%); purified by CC (Et_3_N:Et_2_O:petroleum ether = 1:25:4). Yield: 296 mg (0.600 mmol, 85%) of colorless solid; mp = 87–92 °C.

$${\left[\alpha \right]}_{D}^{20}$$

= –79.3 (c = 0.16, CH_2_Cl_2_). CHN analysis for C_23_H_29_F_6_N_3_S requires: C, 55.97; H, 5.92; N, 8.51 and found: C, 56.23; H, 6.20; N, 8.26. EI-HRMS: *m/z* = 494.2060 (MH^+^); C_23_H_30_F_6_N_3_S requires: *m/z* = 508.2216 (MH^+^). *ν*_max_ 3147, 2034, 2990, 2958, 2885, 2678, 2605, 2504, 1633, 1601, 1556, 1473, 1461, 1386, 1329, 1316, 1271, 1224, 1207, 1173, 1120, 965, 876, 782, 678 cm^−1^. ^1^H-NMR (500 MHz, DMSO-*d*_6_): *δ* 0.90 (*s*, 3H, Me); 0.96 (*s*, 3H, Me); 0.98 (br *s*, 1H, Me); 1.26 (br *s*, 1H); 1.39–1.87 (*m*, 8H); 2.07–2.76 (*m*, 6H); 3.35 (*s*, 1H); 4.50 (br *s*, 1H); 7.71 (*s*, 1H); 8.01–8.67 (*m*, 3H), 10.21 (br *s*, 1H). ^13^C-NMR (126 MHz, DMSO-*d*_6_): *δ* 19.22, 19.98, 23.50, 25.97, 27.57, 37.11, 39.52, 44.61, 47.74, 51.52, 55.98, 56.90, 58.53, 115.69, 121.19, 123.26 (*q*, *J* = 272.7 Hz), 130.27 (*m*), 141.87, 180.64.

#### 3.3.6. Synthesis of 1-(3,5-Bis(trifluoromethyl)phenyl)-3-((1*R*,2*R*,4*R*)-7,7-dimethyl-1-(pyrrolidin-1-yl-methyl)bicyclo[2.2.1]heptan-2-yl)thiourea (**53**)

Compound **53** Prepared from diamine **23a** (177 mg, 0.796 mmol), Et_2_O (2.5 mL), 1-isothiocyanato-3,5-bis(trifluoromethyl)benzene (142 µL, 0.760 mmol, 98%) using General Procedure 6 and purified by CC (Et_3_N:Et_2_O:petroleum ether = 1:35:25). Yield: 353 mg (0.716 mmol, 90%) of colorless solid; mp = 57–58 °C.

$${\left[\alpha \right]}_{D}^{20}$$

= +80.5 (c = 0.65, CH_2_Cl_2_). CHN analysis for C_23_H_29_F_6_N_3_S requires: C, 55.97; H, 5.92; N, 8.51 and found: C, 56.10; H, 6.13; N, 8.43. EI-HRMS: *m/z* = 494.2057 (MH^+^); C_23_H_30_F_6_N_3_S requires: *m/z* = 494.2059 (MH^+^). *ν*_max_ 3415, 3220, 2958, 2881, 2821, 1597, 1508, 1470, 1378, 1274, 1252, 1215, 1169, 1126, 1106, 1076, 1001, 985, 936, 880, 847, 799, 721, 700, 681, 619 cm^−1^. ^1^H-NMR (500 MHz, DMSO-*d*_6_): *δ* 0.88 (*s*, 3H, Me); 1.04 (*s*, 3H); 1.10–1.19 (*m*, 1H); 1.28–2.26 (*m*, 10H); 2.47 (br *s*, 4H); 2.56–3.00 (*m*, 2H); 4.27 (*s*, 1H), 7.71 (*s*, 1H); 8.25 (*s*, 1H); 8.34 (*s*, 2H); 10.29 (*s*, 1H). ^13^C-NMR (126 MHz, DMSO-*d*_6_): *δ* 20.51, 20.68, 23.42, 26.69, 33.88, 39.52, 45.04, 47.62, 51.11, 54.07, 55.46, 60.40, 115.61, 120.93, 123.27 (*q*, *J* = 272.5 Hz), 130.14 (*m*), 142.04, 178.81.

#### 3.3.7. Synthesis of 1-(3,5-Bis(trifluoromethyl)phenyl)-3-((1*R*,2*S*,4*R*)-7,7-dimethyl-1-(piperidin-1-yl-methyl)bicyclo[2.2.1]heptan-2-yl)thiourea (**54**)

Following General Procedure 6 this compound was prepared from diamine **22b**/**23b** (54 mg, 0.228 mmol, **22b**:**23b** = 4.8:1), Et_2_O (2.5 mL), 1-isothiocyanato-3,5-bis(trifluoromethyl)benzene (41 µL, 0.220 mmol, 98%); purified by CC (1. Et_3_N:Et_2_O:petroleum ether = 1:6:25 for the elution of nonpolar impurities; 2. Et_3_N:Et_2_O = 1:5 for the elution of product **54**). Yield: 111 mg (0.219 mmol, 96%) of yellow solid; mp = 131–134 °C.

$${\left[\alpha \right]}_{D}^{20}$$

= −51.2 (c = 0.40, CH_2_Cl_2_). CHN analysis for C_24_H_31_F_6_N_3_S requires: C, 56.79; H, 6.16; N, 8.28 and found: C, 57.03; H, 6.23; N, 8.09. EI-HRMS: *m/z* = 508.2226 (MH^+^); C_24_H_32_F_6_N_3_S requires: *m/z* = 508.2216 (MH^+^). *ν*_max_ 3130, 2939, 1618, 1539, 1506, 1468, 1374, 1352, 1272, 1242, 1172, 1129, 1096, 1050, 1036, 982, 944, 884, 784, 701, 681, 651, 617 cm^−1^. ^1^H-NMR (500 MHz, DMSO-*d*_6_): *δ* 0.90 (*s*, 3H, Me); 0.95 (*s*, 3H, Me); 0.92–1.01 (*m*, 1H); 1.14–1.35 (*m*, 7H); 1.54–1.64 (*m*, 2H); 1.68–1.82 (*m*, 2H); 2.22–2.43 (*m*, 7H); 4.44 (br *s*, 1H); 7.74 (*s*, 1H); 8.15–8.33 (*m*, 3H, 3H of Ar); 10.24 (br *s*, 1H). ^13^C-NMR (126 MHz, DMSO-*d*_6_): *δ* 19.2, 20.0, 23.3, 25.5, 25.8, 27.5, 37.3, 44.7, 47.6, 51.4, 56.3, 57.8, 59.5, 115.9, 121.4, 123.22 (*q*, *J* = 273 Hz), 130.47 (*q*, *J* = 33 Hz), 141.9, 180.5.

#### 3.3.8. Synthesis of 1-(3,5-Bis(trifluoromethyl)phenyl)-3-((1*R*,2*S*,4*R*)-7,7-dimethyl-1-(morpholino-methyl)bicyclo[2.2.1]heptan-2-yl)thiourea (**55**)

Following General Procedure 6 compound **55** was prepared from diamine **22c**/**23c** (460 mg, 1.93 mmol, **22c**:**23c** = 4.6:1), Et_2_O (5 mL), 1-isothiocyanato-3,5-bis(trifluoromethyl)benzene (335 µL, 1.80 mmol, 98%); purified by CC (Et_2_O:Et_3_N = 50:1). Yield: 207 mg (0.405 mmol, 21%) of yellow solid; mp = 52–55 °C.

$${\left[\alpha \right]}_{D}^{20}$$

= −39.5 (c = 0.44, CH_2_Cl_2_). CHN analysis for C_23_H_29_F_6_N_3_OS requires: C, 54.21; H, 5.74; N, 8.25 and found: C, 53.95; H, 5.75; N, 7.95. EI-HRMS: *m/z* = 510.2010 (MH^+^); C_23_H_30_F_6_N_3_OS requires: *m/z* = 510.2008 (MH^+^). *ν*_max_ 3194, 2959, 2882, 2854, 2818, 1619, 1507, 1469, 1375, 1349, 1318, 1276, 1171, 1118, 1068, 1051, 1034, 1006, 993, 977, 964, 944, 931, 910, 885, 864, 802, 730, 701, 681, 653, 619 cm^−1^. ^1^H-NMR (500 MHz, DMSO-*d*_6_): *δ* 0.91 (*s*, 3H, Me); 0.89–0.94 (*m*, 1H); 0.98 (*s*, 3H, Me); 1.19–1.32 (*m*, 1H); 1.55–1.66 (*m*, 2H); 1.70–1.83 (*m*, 2H); 2.27–2.45 (*m*, 7H); 3.39–3.50 (*m*, 4H); 4.62 (br *s*, 1H); 7.73 (*s*, 1H, 1H of Ar); 8.16 (br *s*, 1H); 8.31 (*s*, 2H, 2H of Ar); 10.25 (br *s*, 1H). ^13^C-NMR (126 MHz, DMSO-*d*_6_): *δ* 19.3, 20.0, 25.5, 27.6, 37.2, 44.6, 47.9, 51.6, 55.4, 57.2, 59.2, 66.4, 115.8, 121.1, 123.26 (*q*, *J* = 273 Hz), 130.28 (*q*, *J* = 32 Hz), 142.0, 180.2.

#### 3.3.9. Synthesis of 1-(3,5-Bis(trifluoromethyl)phenyl)-3-((1*R*,2*S*,4*R*)-1-((dimethylamino)methyl)-7,7-dimethylbicyclo[2.2.1]heptan-2-yl)thiourea (**56**)

Prepared from diamine **22d**/**23d** (310 mg, 1.58 mmol, **22d**:**23d** = 2.8:1), Et_2_O (3 mL), 1-isothiocyanato-3,5-bis(trifluoromethyl)benzene (274 µL, 1.47 mmol, 98%) following General Procedure 6. Purified by CC (1. Et_3_N:Et_2_O:petroleum ether = 1:6:25 for the elution of nonpolar impurities; 2. Et_3_N:Et_2_O = 1:5 for the elution of product **56**). Yield: 538 mg (1.15 mmol, 73%) of yellow solid; mp = 61–68 °C.

$${\left[\alpha \right]}_{D}^{20}$$

= −70.8 (c = 0.37, CH_2_Cl_2_). CHN analysis for C_21_H_27_F_6_N_3_S requires: C, 53.95; H, 5.82; N, 8.99 and found: C, 54.00; H, 5.91; N, 8.79. EI-HRMS: *m/z* = 468.1903 (MH^+^); C_21_H_28_F_6_N_3_S requires: *m/z* = 468.1903 (MH^+^). *ν*_max_ 3412, 3205, 2954, 2882, 2830, 2783, 1600, 1516, 1470, 1375, 1322, 1274, 1216, 1169, 1124, 1034, 1016, 1001, 980, 942, 880, 846, 833, 726, 699, 681, 620 cm^−1^. ^1^H-NMR (500 MHz, DMSO-*d*_6_): *δ* 0.89 (*s*, 3H, Me); 0.95 (*s*, 3H, Me); 1.03 (br *s*, 1H); 1.11–1.39 (*m*, 1H); 1.52–1.84 (*m*, 4H); 2.00–2.43 (*m*, 9H); 4.51 (*s*, 1H); 7.71 (*s*, 1H); 7.89–8.89 (*m*, 3H); 10.29 (*s*, 1H). ^13^C-NMR (126 MHz, DMSO-*d*_6_): *δ* 19.18, 20.02, 25.19, 27.46, 33.41, 37.16, 44.49, 47.83, 51.66, 57.84, 60.25, 115.67, 121.26, 123.22 (*q*, *J* = 272.8 Hz), 130.22 (*m*), 141.85, 180.39.

#### 3.3.10. Synthesis of 1-(tert-butyl)-3-((1*R*,2*S*,4*R*)-7,7-dimethyl-1-(pyrrolidin-1-ylmethyl)bicyclo-[2.2.1]heptan-2-yl)thiourea (**57**)

Using General Procedure 6 this substance was prepared from diamine **22a** (50 mg, 0.225 mmol), Et_2_O (1 mL), 2-isothiocyanato-2-methylpropane (28 μL, 0.214 mmol); purified by CC (Et_2_O:MeOH:Et_3_N = 100:1:1). Yield: 56 mg (0.1665 mmol, 74%) of colorless solid; mp = 40–42 °C.

$${\left[\alpha \right]}_{D}^{20}$$

= −167.0 (c = 0.12, CH_2_Cl_2_). EI-HRMS: *m*/*z* = 338.2626 (MH^+^); C_19_H_36_N_3_S requires: *m/z* = 338.2624 (MH^+^). *ν*_max_ 3274, 2956, 2876, 2803, 1517, 1477, 1459, 1390, 1336, 1309, 1274, 1251, 1225, 1197, 1110, 1069, 1034, 1000, 966, 940, 921, 908, 873, 780, 763, 701, 657, 609 cm^−1^. ^1^H-NMR (500 MHz, DMSO-*d*_6_): *δ* 0.70–0.78 (*m*, 1H); 0.89 (*s*, 3H, Me); 0.93 (*s*, 3H, Me); 1.10–1.21 (*m*, 1H); 1.40 (*s*, 9H, Boc); 1.47–1.58 (*m*, 2H); 1.58–1.67 (*m*, 4H); 1.66–1.75 (*m*, 2H); 2.22–2.32 (*m*, 1H); 2.33–2.54 (*m*, 6H); 4.48 (br *s*, 1H); 7.17 (br *s*, 1H); 7.25 (*s*, 1H). ^13^C-NMR (126 MHz, DMSO-*d*_6_): *δ* 19.3, 20.1, 23.7, 25.6, 27.7, 29.0, 38.1, 44.7, 48.0, 51.7, 51.9, 55.9, 56.3, 56.4, 181.7.

#### 3.3.11. Synthesis of 1-(Adamantan-1-yl)-3-((1*R*,2*S*,4*R*)-7,7-dimethyl-1-(pyrrolidin-1-ylmethyl)-bicyclo[2.2.1]heptan-2-yl)thiourea (**58**)

Following General Procedure 6 this compound was prepared from diamine **22a** (47 mg, 0.211 mmol), Et_2_O (2 mL), 1-isothiocyanatoadamantane (39 mg, 0.200 mmol); purified by CC (Et_2_O:MeOH:Et_3_N = 100:1:1). Yield: 57 mg (0.137 mmol, 65%) of colorless solid; mp = 144–145 °C.

$${\left[\alpha \right]}_{D}^{20}$$

= −58.7 (c = 0.2, CH_2_Cl_2_). EI-HRMS: *m/z* = 416.3095 (MH^+^); C_25_H_42_N_3_S requires: *m/z* = 416.3094 (MH^+^). *ν*_max_ 3234, 2904, 2850, 2788, 1505, 1453, 1358, 1339, 1305, 1278, 1224, 1189, 1115, 1092, 1068, 1051, 1038, 939, 875, 841, 775, 624 cm^−1^. ^1^H-NMR (500 MHz, DMSO-*d*_6_): *δ* 0.68–0.78 (*m*, 1H); 0.88 (*s*, 3H, Me); 0.93 (*s*, 3H, Me); 1.11–1.21 (*m*, 1H); 1.53 (*q*, *J* = 5.6, 4.3 Hz, 2H); 1.58–1.66 (*m*, 11H); 1.66–1.74 (*m*, 2H); 1.98–2.05 (*m*, 3H); 2.09–2.17 (*m*, 6H); 2.21–2.31 (*m*, 1H); 2.35–2.49 (*m*, 5H); 4.47 (br *s*, 1H); 7.09–7.21 (*m*, 2H). ^13^C-NMR (126 MHz, DMSO-*d*_6_): *δ* 19.3, 20.2, 23.8, 25.7, 27.8, 29.0, 36.1, 38.1, 41.3, 44.7, 45.7, 48.0, 51.8, 52.5, 56.0, 56.5, 64.9, 181.0. 

#### 3.3.12. Synthesis of 1-(3,5-bis(trifluoromethyl)phenyl)-3-(((1*R*,2*S*,4*R*)-7,7-dimethyl-2-(pyrrolidin-1-yl)bicyclo[2.2.1]heptan-1-yl)methyl)thiourea (**59**)

Prepared from diamine **34** (248 mg, 1.115 mmol), Et_2_O (2.5 mL), 1-isothiocyanato-3,5-bis(trifluoromethyl)benzene (186 µL, 1.00 mmol, 98%) following General Procedure 6. Purified by CC (Et_3_N:EtOAc:petroleum ether = 1:1:20). Yield: 396 mg (0.803 mmol, 72%) of yellow solid; mp = 53–57 °C.

$${\left[\alpha \right]}_{D}^{20}$$

= −21.1 (c = 0.25, CH_2_Cl_2_). EI-HRMS: *m/z* = 494.2063 (MH^+^); C_23_H_30_F_6_N_3_S requires: *m/z* = 494.2059 (MH^+^). *ν*_max_ 3141, 2953, 2880, 2819, 1667, 1619, 1521, 1466, 1374, 1274, 1171, 1128, 1106, 947, 884, 849, 701, 680, 650 cm^−1^. ^1^H-NMR (600 MHz, DMSO-*d*_6_): *δ* 0.95 (*s*, 3H, Me); 0.98 (*s*, 3H, Me); 1.14–1.23 (*m*, 2H); 1.33–1.41 (*m*, 1H); 1.48–1.54 (*m*, 1H); 1.56–1.67 (*m*, 4H); 1.68–1.76 (*m*, 1H); 1.92–2.05 (*m*, 2H); 2.42–2.61 (*m*, 4H); 2.73–2.78 (*m*, 1H); 3.51 (*dd*, *J* = 3.5; 14.4 Hz, 1H); 3.93 (*dd*, *J* = 5.1; 15.0 Hz, 1H); 7.73 (*s*, 1H, 1H of Ar); 7.87 (br *s*, 1H, NH); 8.32 (*s*, 2H, 2H of Ar); 10.29 (br *s*, 1H, NH). ^13^C-NMR (151 MHz, DMSO-*d*_6_): *δ* 19.6, 20.5, 22.9, 24.9, 27.9, 34.9, 45.0, 45.3, 48.8, 51.6, 53.0, 65.4, 115.9, 121.3, 123.23 (*q*, *J* = 273 Hz), 130.25 (*q*, *J* = 33 Hz), 141.9, 180.0.

#### 3.3.13. Synthesis of 1-(3,5-bis(trifluoromethyl)phenyl)-3-(((1*R*,2*R*,4*R*)-7,7-dimethyl-2-(pyrrolidin-1-yl)bicyclo[2.2.1]heptan-1-yl)methyl)thiourea (**60**)

Following General Procedure 6 the title compound was prepared from diamine **33** (143 mg, 0.643 mmol), Et_2_O (2.5 mL), 1-isothiocyanato-3,5-bis(trifluoromethyl)benzene (108 µL, 0.580 mmol, 98%); purified by CC (Et_3_N:Et_2_O:petroleum ether = 1:20:20). Yield: 175 mg (0.345 mmol, 55%) of yellow solid; mp = 53–55 °C.

$${\left[\alpha \right]}_{D}^{20}$$

= −47.8 (c = 0.24, CH_2_Cl_2_). CHN analysis for C_23_H_29_F_6_N_3_S requires: C, 55.97; H, 5.92; N, 8.51 and found: C, 55.77; H, 6.14; N, 8.14. EI-HRMS: *m/z* = 494.2061 (MH^+^); C_23_H_30_F_6_N_3_S requires: *m/z* = 494.2059 (MH^+^). *ν*_max_ 3144, 2953, 2877, 2815, 1667, 1508, 1468, 1376, 1274, 1171, 1128, 947, 884, 848, 720, 701, 681, 649 cm^−1^. ^1^H-NMR (600 MHz, DMSO-*d*_6_): *δ* 0.89 (*s*, 3H, Me); 1.05–1.15 (*m*, 1H); 1.14 (*s*, 3H, Me); 1.17–1.27 (*m*, 1H); 1.47–1.59 (*m*, 6H); 1.58–1.64 (*m*, 1H); 1.66–1.75 (*m*, 1H); 1.92–2.01 (*m*, 1H); 2.37–2.54 (*m*, 5H); 3.45 (*dd*, *J* = 3.1; 14.2 Hz, 1H); 3.78 (*dd*, *J* = 5.0; 14.2 Hz, 1H); 7.74 (*s*, 1H, 1H of Ar); 8.06 (br *s*, 1H); 8.33 (*s*, 2H, 2H of Ar); 10.32 (br *s*, 1H). ^13^C-NMR (151 MHz, DMSO-*d*_6_): *δ* 20.7, 20.9, 22.8, 26.5, 33.0, 35.6, 45.2, 45.5, 47.1, 50.9, 52.9, 72.2, 116.0, 121.5, 123.22 (*q*, *J* = 273 Hz), 130.26 (*q*, *J* = 33 Hz), 141.8, 180.0.

#### 3.3.14. Synthesis of 1-(3,5-bis(trifluoromethyl)phenyl)-3-((1*S*,2*S*,4*R*)-7,7-dimethyl-1-(2-(pyrrolidin-1-yl)ethyl)bicyclo[2.2.1]heptan-2-yl)thiourea (**61**)

Following General Procedure 6. Prepared from diamine **38** (25 mg, 0.106 mmol), Et_2_O (2.5 mL), 1-isothiocyanato-3,5-bis(trifluoromethyl)benzene (18.6 µL, 0.10 mmol, 98%); purified by CC (1. Et_3_N:Et_2_O:petroleum ether = 1:6:25 for the elution of nonpolar impurities; 2. Et_3_N:Et_2_O = 1:5 for the elution of product **61**). Yield: 49 mg (0.0954 mmol, 90%) of yellow solid; mp = 66–70 °C.

$${\left[\alpha \right]}_{D}^{20}$$

= −16.1 (c = 0.18, CH_2_Cl_2_). CHN analysis for C_24_H_31_F_6_N_3_S requires: C, 56.79; H, 6.16; N, 8.28 and found: C, 56.48; H, 6.21; N, 8.00. EI-HRMS: *m/z* = 508.2224 (MH^+^); C_24_H_32_F_6_N_3_S requires: *m/z* = 508.2216 (MH^+^). *ν*_max_ 3178, 3142, 2959, 2880, 2808, 1618, 1533, 1468, 1375, 1346, 1274, 1171, 1129, 882, 701, 681, 655, 620 cm^−1^. ^1^H-NMR (500 MHz, DMSO-*d*_6_): *δ* 0.88 (*s*, 3H, Me); 0.90–0.98 (*m*, 1H); 0.94 (*s*, 3H, Me); 1.20–1.30 (*m*, 1H); 1.40–1.59 (*m*, 7H); 1.64 (*s*, 1H); 1.69–1.80 (*m*, 2H); 2.28–2.46 (*m*, 7H); 4.53 (br *s*, 1H); 7.71 (*s*, 1H); 8.22 (*s*, 2H); 8.74 (br *s*, 1H); 10.08 (br *s*, 1H). ^13^C-NMR (126 MHz, DMSO-*d*_6_): *δ* 18.9, 19.9, 22.8, 25.7, 27.7, 28.9, 44.4, 45.7, 48.8, 51.0, 52.8, 53.4, 58.3, 115.5, 120.8, 123.22 (*q*, *J* = 273 Hz), 130.29 (*q*, *J* = 32 Hz), 142.2, 180.6.

#### 3.3.15. Synthesis of 1-(3,5-bis(trifluoromethyl)phenyl)-3-(2-((1*S*,2*R*,4*R*)-7,7-dimethyl-2-(pyrrolidin-1-yl)bicyclo[2.2.1]heptan-1-yl)ethyl)thiourea (**62**)

Following General Procedure 6 this compound was prepared from diamine **46** (100 mg, 0.423 mmol), Et_2_O (2.5 mL), 1-isothiocyanato-3,5-bis(trifluoromethyl)benzene (74.5 µL, 0.400 mmol, 98%); purified by CC (EtOAc:MeOH = 10:1). Yield: 103 mg (0.203 mmol, 48%) of colorless solid; mp = 60–62 °C.

$${\left[\alpha \right]}_{D}^{20}$$

= −40.3 (c = 0.12, CH_2_Cl_2_). EI-HRMS: *m/z* = 508.2215 (MH^+^); C_24_H_32_F_6_N_3_S requires: *m/z* = 508.2216 (MH^+^). *ν*_max_ 3238, 2951, 2878, 2815, 1676, 1622, 1546, 1471, 1380, 1348, 1276, 1172, 1130 999, 946, 884, 847, 724, 701, 680 cm^−1^. ^1^H-NMR (500 MHz, DMSO-*d*_6_): *δ* 0.83 (*s*, 3H, Me); 1.02 (*s*, 3H, Me); 0.97–1.15 (*m*, 2H); 1.39–1.52 (*m*, 1H); 1.52–1.72 (*m*, 8H); 1.72–1.86 (*m*, 1H); 1.87–1.97 (*m*, 1H); 2.21–2.35 (*m*, 1H); 2.42–2.63 (*m*, 4H); 3.44–3.70 (*m*, 2H); 7.72 (*s*, 1H); 8.25 (*s*, 2H); 8.40 (*s*, 1H, NH); 10.03 (br *s*, 1H, NH). ^13^C-NMR (126 MHz, DMSO-*d*_6_): *δ* 20.0, 21.3, 22.9, 26.7, 26.9, 32.6, 35.9, 41.5, 44.3, 47.7, 51.2, 53.4, 72.4, 115.8, 121.6, 123.28 (*q*, *J* = 273 Hz), 130.11 (*q*, *J* = 34 Hz), 142.1, 180.2.

#### 3.3.16. Synthesis of 1-(3,5-bis(trifluoromethyl)phenyl)-3-(2-((1*S*,2*S*,4*R*)-7,7-dimethyl-2-(pyrrolidin-1-yl)bicyclo[2.2.1]heptan-1-yl)ethyl)thiourea (**63**)

Prepared from diamine **47** (189 mg, 0.80 mmol), Et_2_O (2.5 mL), 1-isothiocyanato-3,5-bis(trifluoromethyl)benzene (152 µL, 0.80 mmol, 98%) following General Procedure 6 and purified by CC (Et_3_N:EtOAc:MeOH = 0.2:20:1). Yield: 95 mg (0.187 mmol, 23%) of colorless solid; mp = 47–48 °C. ${\left[\alpha \right]}_{D}^{20}$ = +49.3 (c = 0.28, CH_2_Cl_2_). EI-HRMS: *m/z* = 508.2210 (MH^+^); C_24_H_32_F_6_N_3_S requires: *m/z* = 508.2216 (MH^+^). *ν*_max_ 3241, 2946, 2879, 2808, 1620, 1541, 1376, 1344, 1273, 1169, 1125, 999, 947, 882, 847, 725, 700, 680 cm^−1^. ^1^H-NMR (500 MHz, DMSO-*d*_6_): *δ* 0.94 (*s*, 3H, Me); 1.00 (*s*, 3H, Me); 1.08–1.14 (*m*, 1H); 1.14–1.20 (*m*, 1H); 1.29–1.38 (*m*, 1H); 1.45–1.50 (*m*, 1H); 1.59–1.76 (*m*, 7H); 1.86–1.94 (*m*, 1H); 1.94–2.02 (*m*, 1H); 2.52–2.71 (*m*, 6H); 3.60 (br *s*, 1H); 7.70 (*s*, 1H); 8.21 (*s*, 2H); 8.40 (br *s*, 1H, NH); 9.99 (br *s*, 1H, NH). ^13^C-NMR (126 MHz, DMSO-*d*_6_): *δ* 20.1, 20.6, 22.9, 26.2, 28.1, 29.8, 35.5, 41.2, 45.1, 49.6, 50.5, 53.2, 66.8, 115.8, 121.8, 123.28 (*q*, *J* = 273 Hz), 130.11 (*q*, *J* = 33 Hz), 142.1, 180.2.

## 4. Conclusions

Starting from two commercial building blocks, (1*S*)-(+)-10-camphorsulfonic acid (for the preparation of 10-iodocamphor (**18**)) and (1*S*)-(+)-ketopinic acid (**1**), a series of 18 camphor-derived 1,2-, 1,3-, and 1,4-diamines (both regioisomers; *exo*- and *endo*-epimers) have been synthesized. The applied synthetic methods are robust and structural diversity-oriented – they enable the preparation of both diastereomers (*exo* and *endo*) as well as both regioisomers of each type of diamine. Amines have been transformed with selected isothiocyanates into the corresponding noncovalent bifunctional thiourea organocatalysts (16 compounds), which have been evaluated for the Michael addition of dimethyl malonate, acetylacetone, and/or dibenzoylmethane to *trans*-β-nitrostyrene. The highest selectivity was achieved with the *endo*-1,3-diamine derived catalyst **52** (91.5:8.5 er (*S*), full conversion), while the corresponding *exo*-1,3-diamine-derived catalyst of **53**, gave the same product with complementary selectivity (82:18% er (*R*), 63% conversion). The preference of the 1,3-diamine derived organocatalysts versus the 1,2- and 1,4-diamine-derived organocatalysts needs to be further investigated mechanistically, both experimentally and theoretically (mechanistic model of activation [20,74,75,76], geometry of the transition state). After all, most of the established efficient organocatalysts are derivatives of 1,2-diamnies [20,45], while efficient 1,3-diamine derived organocatalysts are extremely rare [43,77,78,79,80,81]. The performance of the reported camphor-derived noncovalent, bifunctional, thiourea organocatalysts has so far not matched the efficiency of the established organocatalysts of this type [20,45]. The reported organocatalyst need to be evaluated further in various other organocatalyzed transformations, while their diamine precursors will be transformed into other types of organocatalysts and evaluated in selected model organocatalyzed transformations to acquire a bigger picture of their efficiency in comparison to already established catalysts [20,45]. All the new organocatalysts have been fully characterized including the determination of the absolute configuration at the newly formed stereogenic center. In terms of structure, camphor-derived diamines **48**–**63** cover the representative part of (regio and stereo) chemical space allocated to camphor-based 1,2-, 1,3-, and 1,4-diamines. With regard to accessibility, synthetic methods reported in this paper allow for straightforward access to the title nonracemic diamines, which are very useful building blocks for the preparation of a different kind of nonracemic organocatalysts and ligands for the use in transition-metal catalysis.

## Figures and Tables

**Table 1:** Catalytic activity of camphor-diamine derived noncovalent bifunctional thiourea organocatalysts **48**–**63** in 1,4-additions of dimethyl malonate to *trans*-β-nitrostyrene ^1^.

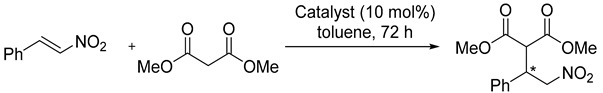				
Entry	Catalyst	Temperature (°C)	Conversion (%)	Er
1	**48**	25	38	68.5:31.5; (*S*)
2	**48**	−25	30	66.5:33.5; (*S*)
3	**49**	25	61	61:39; (*R*)
4	**49**	−25	16	53:47; (*R*)
5	**50**	25	20	66.5:33.5; (*R*)
6	**50**	−25	6	61:39; (*R*)
7	**51**	25	5	57:43; (*S*)
8	**51**	−25	2	59:41; (*S*)
9	**52**	25	>99	78.5:21.5; (*R*)
10	**52**	−25	90	78.5:21.5; (*R*)
11	**53**	25	69	77.5:22.5; (*S*)
12	**53**	−25	15	66.5:33.5; (*S*)
13	**54**	25	80	61.5:38:5; (*R*)
14	**54**	−25	64	55:45; (*R*)
15	**55**	25	10	54.5:45.5; (*R*)
16	**56**	25	>99	63:37; (*R*)
17	**56**	−25	>99	61.5:38.5; (*R*)
18	**57**	25	79	50.5:49.5 (*R*)
19	**58**	25	85	50.5:49.5 (*S*)
20 ^2^	**59**	25	99	66.5:33.5; (*S*)
21	**59**	−25	61	64:36; (*S*)
22 ^2^	**60**	25	9	56:44; (*S*)
23	**60**	−25	6	67:33; (*S*)
24	**61**	−25	93	55:45; (*R*)
25	**62**	25	>99	57.5:42.5; (*S*)
26	**62**	−25	74	62.5:37.5; (*S*)
27	**63**	25	>99	54.5:45.5; (*S*)
28	**63**	−25	>99	50.5:49.5; (*S*)

^1^ Unless otherwise noted, the following conditions were applied: *trans*-β-Nitrostyrene (60 mg, 0.4 mmol), dimethyl malonate (92 μL, 0.8 mmol), thiourea organocatalyst **48**–**63** (10 mol%, relative to *trans*-β-nitrostyrene), anhydrous toluene (1 mL), −25 °C or 25 °C, 72 h; conversion was determined by ^1^H-NMR (CDCl_3_); er was determined by HPLC (chiral column: Chiralpak AD-H; mobile phase: *n*-Hexane/*i*-PrOH = 90:10; flow rate 1.0 mL/min; λ = 210 nm; product: tR = 9.2 minutes and 13.3 min). ^2^ Reaction time was 48 h.

**Table 2:** Catalytic activity of selected camphor-diamine derived noncovalent bifunctional thiourea organocatalysts **48**, **49**, **52**, and **53** in 1,4-additions of 1,3-diketones to *trans*-β-nitrostyrene ^1^.

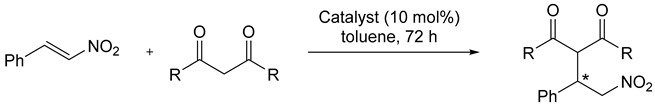					
Entry	Catalyst	R	Temperature (°C)	Conversion (%)	Er
1	**48**	Me	25	96	69:31; (*S*)
2	**49**	Me	25	>99	80.5:19.5; (*R*)
3	**52**	Me	25	>99	87:13; (*S*)
4	**52**	Me	−25	>99	91.5:8.5; (*S*)
5 ^2^	**52**	Me	25	>99	74.5:25.5; (*S*)
6 ^3^	**52**	Me	25	>99	81:19; (*S*)
7	**52**	Ph	25	>99	87:13; (*S*)
8	**53**	Me	25	96	79.5:20.5; (*R*)
9	**53**	Me	−25	63	82:18; (*R*)
10	**53**	Ph	25	>99	81:19; (*R*)

^1^ Unless otherwise noted, the following conditions were applied: *trans-*β-Nitrostyrene (60 mg, 0.4 mmol), acetylacetone (82 μL, 0.8 mmol) or dibenzoylmethane (180 mg, 0.8 mmol), thiourea organocatalyst (10 mol%, relative to *trans*-β-nitrostyrene), anhydrous toluene (1 mL), −25 °C or 25 °C, 72 h; conversion was determined by ^1^H-NMR (CDCl_3_); er was determined by HPLC (1. for the reaction with acetylacetone: chiral column: Chiralpak AD-H; mobile phase: *n*-Hexane/*i*-PrOH = 90:10; flow rate 1.0 mL/min; λ = 210 nm; product: tR = 8.8 minutes and 11.4 min; 2. for the reaction with dibenzoylmethane: chiral column: Chiracel OD-H; mobile phase: *n*-Hexane/*i*-PrOH = 80:20; flow rate 1.0 mL/min; λ = 210 nm; product: tR = 9.6 minutes and 22.4 min). ^2^ Reaction was performed in CH_2_Cl_2_. ^3^ Reaction was performed in THF.

## Supplementary Materials

The following are available online. Synthesis and Characterization Data for Compounds **2**–**17**, **20b**–**d**, **21b**–**d**, **22b**–**d**, **23b**–**d**, and **24**–**63**; Structure Determination by NMR, Figure S1: Determination of the absolute configuration at the C-2 based on the observed NOE correlation spectroscopy cross peaks, Figure S2: Determination of the absolute configuration at C-2 based on chemical shift correlations in the series of primary endo-amines; Structure Determination by X-ray Diffraction Analysis, Figures S3–S11: Ortep drawings of compounds **8**, **18**, **21a**, **22a**, **24**, **28**, **29**, **37**, and **52**.
