# Supplementary material for: Stereodivergent Synthesis of Camphor-Derived Diamines and Their Application as Thiourea Organocatalysts
Source: Molecules. 2020 Jun 29;25(13):2978. doi: 10.3390/molecules25132978 (PMC7412124; doi:10.3390/molecules25132978)
Supplement: Supplementary file 1 [file molecules-25-02978-s001.pdf]

Supporting Information

Stereodivergent Synthesis of Camphor-Derived Diamines and Their Application as Thiourea Organocatalysts

Sebastijan Ričko, Franc Požgan, Bogdan Štefane, Jurij Svete, Amalija Golobič, and Uroš Grošelj*

University of Ljubljana, Faculty of Chemistry and Chemical Technology, Večna pot 113, 1000 Ljubljana, Slovenia

* Correspondence: uros.groselj@fkkt.uni-lj.si; Tel.: +386-1-479-8565 (U.G.)

Received: date; Accepted: date; Published: date

Table of Contents

1. Materials and Methods, Synthesis and Characterization Data of Compounds **2–17**, **20b–d**, **21b–d**, **22b–d**, **23b–d**, and **24–63**.

2. Structure Determination by NMR.

3. Structure Determination by X-Ray Diffraction Analysis.

4. Copies of NMR spectra of Compounds **5**, **7**, **8**, **10–16**, **20b–d**, **21b–d**, **22b–d**, **23b–d**, **24–43**, **45**, **48–63** (^{1}H-, ^{13}C-NMR, 2D NOESY)

5. Selected HPLC data

6. References.

1. Materials and Methods

1.1. Materials and General Methods

Solvents for extractions and chromatography were of technical grade and were distilled prior to use. Extracts were dried over technical grade Na_2SO_4. Melting points were determined on a Kofler micro hot stage and on SRS OptiMelt MPA100 – Automated Melting Point System (Stanford Research Systems, Sunnyvale, California, United States). The NMR spectra, including 2D NOESY spectra, were obtained on a Bruker UltraShield 500 plus (Bruker, Billerica, Massachusetts, United States) at 500 MHz for ^{1}H and 126 MHz for ^{13}C nucleus, using DMSO-d_6 and $CDCl_3$ with TMS as the internal standard, as solvents. Factory default experiments were used for the acquisition of the aforementioned NMR spectra. Mass spectra were recorded on an Agilent 6224 Accurate Mass TOF LC/MS (Agilent Technologies, Santa Clara, California, United States), IR spectra on a Perkin-Elmer Spectrum BX FTIR spectrophotometer (PerkinElmer, Waltham, Massachusetts, United States). Microanalyses were performed on a Perkin-Elmer CHN Analyzer 2400 II (PerkinElmer, Waltham, Massachusetts, United States). Column chromatography (CC) was performed on silica gel (Silica gel 60, particle size: 0.035-0.070 mm (Sigma-Aldrich, St. Louis, Missouri, United States)). HPLC analyses were performed on an Agilent 1260 Infinity LC (Agilent Technologies, Santa Clara, California, United States) using CHIRALPAK AD-H (0.46 cm ø × 25 cm) and CHIRALCEL OD-H (0.46 cm ø × 25 cm) as chiral columns (CHIRAL TECHNOLOGIES, INC., West Chester, Pennsylvania, United States). Organocatalyzed reactions were performed on EasyMax 102 Advanced synthesis workstation (Mettler-Toledo, LLC). Catalytic hydrogenation was performed on a Parr Pressure Reaction Hydrogenation Apparatus (Moline, IL, USA). The optical rotation of optical active substances was measured on a Perkin Elmer 241 MC Polarimeter (PerkinElmer, Waltham, Massachusetts, United States) with Na lamp (sodium emission lines at 589.0 nm) at 20°C.

Compounds (1*S*,4*R*)-7,7-dimethyl-1-(pyrrolidin-1-ylmethyl)bicyclo[2.2.1]heptan-2-one (**20a**), (1*S*,4*R*,2*E*)-7,7-dimethyl-1-(pyrrolidin-1-ylmethyl)bicyclo[2.2.1]heptan-2-one oxime (**21a**), (1*S*,2*S*,4*R*)-7,7-dimethyl-1-(pyrrolidin-1-ylmethyl)bicyclo[2.2.1]heptan-2-amine (**22a**), and (1*S*,2*R*,4*R*)-7,7-dimethyl-1-(pyrrolidin-1-ylmethyl)bicyclo[2.2.1]heptan-2-amine (**23a**) [1] were prepared following literature procedures.

Racemic products of 1,4-addition of *trans*-β-nitrostyrene to dimethyl malonate [2,3], acetylacetone [1], and dibenzoylmethane [1], used as standards for determination of ee by HPLC, were prepared following literature procedures.

1.2. General Procedures

1.2.1. General Procedure 1. Synthesis of Free Diamines by Deprotection of *N*-Boc-Amines.

To a solution of Boc-protected amine in anhydrous CH_2Cl_2 at room temperature was, under stirring, slowly added the same volume of anhydrous trifluoroacetic acid. The resulting reaction mixture was stirred at room temperature for 16 h. Volatile components were evaporated *in vacuo*. The residue was dissolved in Et_2O and washed with NaOH (1 M in H_2O, 1/5 of the volume of Et_2O) and NaCl (aq. sat., 1/5 of the volume of Et_2O). The organic phase was dried over anhydrous Na_2SO_4, filtered, and volatile components evaporated *in vacuo*. If necessary, the so obtained product was purified by CC. Fractions containing the pure product were combined and volatile components evaporated *in vacuo* to give the corresponding amine.

1.2.2. General Procedure 2. Synthesis of Oximes.

For the oxime synthesis, a modified procedure from the literature was applied [4]. To a solution of ketone (1 equiv.) in anhydrous EtOH, $NH_2OH·HCl$ (2 equiv.) and pyridine (1.5 equiv.) were added, and the resulting reaction mixture was stirred under reflux for 6-16 h. Volatile components were evaporated *in vacuo*, the residue was suspended in H_2O (15 mL) followed by the addition of finely powdered NaOH till pH ≈ 10-12. The resulting mixture was extracted with Et_2O (3 × 50 mL). The combined organic phase was washed with H_2O (1/5 of the volume of Et_2O) and NaCl (aq. sat., 1/5 of

the volume of Et_2O). The organic phase was dried over anhydrous Na_2SO_4, filtered, and volatile components evaporated *in vacuo*. If necessary, the so obtained oxime was purified by CC. Fractions containing the pure product were combined and volatile components evaporated *in vacuo* to give the corresponding oxime.

1.2.3. General Procedure 3. Synthesis of Tertiary Amines (Pyrrolidines) by Cyclative Bis-Alkylation of Primary Amines.

For the alkylation of primary amines, a modified procedure from the literature was applied [5]. To a suspension of amine (1 equiv.) in H_2O (2 mL), K_2CO_3 (1.1 equiv.) and 1,4-dibromobutane (1.1 equiv.) were added, and the resulting reaction mixture was stirred under microwave irradiation (MW) for 20 minutes (100 W, 125°C, ~5 bar). The reaction mixture was extracted with EtOAc (3 × 25 mL). The combined organic phase was washed with NaCl (aq. sat., 1/5 of the volume of EtOAc). The organic phase was dried over anhydrous Na_2SO_4, filtered, and volatile components evaporated *in vacuo*. If necessary, the so obtained tertiary amine was purified by CC. Fractions containing the pure product were combined and volatile components evaporated *in vacuo* to give the corresponding tertiary amine.

1.2.4. General Procedure 4. Synthesis of Primary Amines by Reduction of Oximes with Sodium.

To a solution of oxime in anhydrous *n*-PrOH under Argon at 95°C, sodium (*ca*. 100-200 mg) was added. Before all the added sodium reacted, another chunk of sodium (*ca*. 100-200 mg) was added, followed by addition of further sodium to ensure a continuous evolution of hydrogen for 1 h. After all the sodium reacted, volatile components were evaporated *in vacuo*, and to the residue, H_2O was added followed by extraction with Et_2O (5 × 25 mL). The combined organic phase was washed with H_2O (1/5 of the volume of Et_2O) and NaCl (aq. sat., 1/5 of the volume of Et_2O). The organic phase was dried over anhydrous Na_2SO_4, filtered, and volatile components evaporated *in vacuo*. If necessary, the so obtained amine was purified/separated by CC. Fractions containing the pure product were combined and volatile components evaporated *in vacuo* to give the corresponding amine.

1.2.5. General Procedure 5. Synthesis of Primary Amines by Hydrogenation of Oximes in the Presence of *Raney*-Ni Catalyst.

Raney-Ni (*ca*. 100-200 mg) was added to a solution of oxime and triethylamine in MeOH under Argon. The reaction vessel was thoroughly flushed with hydrogen and the reaction mixture was hydrogenated in a Parr shaker hydrogenation apparatus in the atmosphere of hydrogen (60 psi) at room temperature for 6 h. The reaction mixture was filtered through a plague of Celite® and washed with MeOH. Volatile components were evaporated *in vacuo*. The residue was dissolved in Et_2O (100 mL) and washed with H_2O (1/10 of the volume of Et_2O) and NaCl (aq. sat., 1/10 of the volume of Et_2O). If the residue after filtration through a plague of Celite® was of green color, due to the presence of nickel species, the residue was dissolved in Et_2O (100 mL) and washed consecutive with NH_4OH (25% aq., 1/10 of the volume of Et_2O) till the disappearance of the green color (the aqueous phase turns violet) and finally NaCl (aq. sat., 1/10 of the volume of Et_2O). The organic phase was dried over anhydrous Na_2SO_4, filtered, and volatile components evaporated *in vacuo*. If necessary, the so obtained amine was purified/separated by CC. Fractions containing the pure product were combined and volatile components evaporated *in vacuo* to give the corresponding amine.

1.2.6. General Procedure 6. Synthesis of Thiourea Derivatives.

To a solution of amine (1 equiv.) in anhydrous Et_2O under Argon at 0°C isothiocyanate (0.95 equiv.) was added. The resulting reaction mixture was stirred at 0°C for 30 minutes and at room temperature for 24 h. Volatile components were evaporated *in vacuo* and the residue was purified by CC. Fractions containing the pure product were combined and volatile components evaporated *in vacuo* to give the corresponding thiourea derivative.

1.2.7. General Procedure 7. Synthesis of *N*-Boc Protected Primary Amines.

To a solution of amine (1 equiv.) in anhydrous CH_2Cl_2 under Argon at room temperature Boc_2O (1.5 equiv.) and Et_3N (2 equiv.) were added. The resulting reaction mixture was stirred at room temperature for 16 h. Volatile components were evaporated *in vacuo* and the residue was purified/separated by CC. Fractions containing the pure product were combined and volatile components evaporated *in vacuo* to give the corresponding *N*-Boc protected amine.

1.2.8. General Procedure 8. Synthesis of Tertiary Amines by Amination of 10-Iodocamphor (**18**).

To a suspension of 10-iodocamphor (**18**) (1 equiv.) and K_2CO_3 (1.5 equiv.) in anhydrous DMSO under Argon secondary amine (15 equiv.) was added, and the resulting reaction mixture was stirred at 110°C for 16 h. The addition of the secondary amine is accompanied with an intense blue or violet coloration, which eventually fades away. The cooled reaction mixture was diluted with H_2O (*ca.* 10 mL of H_2O per 1 mL DMSO) and extracted with EtOAc (*ca.* 3 × (15 mL EtOAc per 10 mL H_2O)). The combined organic phase was washed with H_2O (1/5 of the volume of EtOAc) and NaCl (aq. sat., 1/5 of the volume of EtOAc). The organic phase was dried over anhydrous Na_2SO_4, filtered, and volatile components evaporated *in vacuo*. If necessary, the so obtained tertiary amino-ketone was purified/separated by CC. Fractions containing the pure product were combined and volatile components evaporated *in vacuo* to give the corresponding tertiary amine.

1.2.9. General Procedure 10. Testing the Catalytic Activity of Thiourea Derivatives in 1,4-Additions of 1,3-Dicarbonyl Compounds to *trans*-β-Nitrostyrene.

Dimethyl malonate (92 µL, 0.8 mmol) or acetylacetone (82 µL, 0.8 mmol) or dibenzoylmethane (180 mg, 0.8 mmol) was added to a solution of *trans*-β-nitrostyrene (60 mg, 0.4 mmol) and thiourea organocatalyst **48**–**63** (10 mol%, relative to *trans*-β-nitrostyrene) in anhydrous toluene, CH_2Cl_2, or THF (1 mL) under argon at 25 or –25 °C. The resulting reaction mixture was stirred at 25 or -25 °C for 48–72 h. The reaction mixture was then quickly passed through a short column filled with Silica gel 60 (1 cm diameter, 5 cm length) using a mixture of EtOAc and petroleum ether in a 1:1 ratio as a mobile phase to remove the tested organocatalyst **48**–**63**. Volatile components were evaporated *in vacuo* and the residue was used to determine the conversion by ^{1}H-NMR and enantioselectivity by HPLC.

1.3. Synthesis and Characterization Data of Compounds 2–17, 20b–d, 21b–d, 22b–d, 23b–d, and 24–63.

1.3.1. Synthesis of *tert*-butyl [(1*S*,4*R*)-7,7-dimethyl-2-oxobicyclo[2.2.1]heptan-1-yl]carbamate (**2**) [6].

Prepared following a modified literature procedure [6,7]. To a solution of (1*S*)-(+)-ketopinic acid (**1**) (1.00 g, 5.49 mmol) in anhydrous toluene (25 mL) under argon, Et_3N (888 µL, 6.37 mmol) and DPPA (1.28 mL, 5.82 mmol, 98%) were added. The resulting reaction mixture was stirred under reflux for 3 h. Volatile components were evaporated *in vacuo*, followed by the addition of anhydrous *t*BuOH (20 mL). The resulting reaction mixture was stirred under reflux for 96 h. Volatile components were evaporated *in vacuo*, the residue was dissolved in EtOAc (150 mL) and washed with H_2O (2 × 25 mL) and NaCl (aq. sat., 2 × 25 mL). The organic phase was dried over anhydrous Na_2SO_4, filtered, and volatile components evaporated *in vacuo*. The so obtained Boc-amine **2** was used in the subsequent transformation without further purification. Yield: 1.14 g (4.50 mol, 82%) of colorless solid. Physical and spectral data of compound **2** were in accordance with the literature data [6].

1.3.2. Synthesis of (1*S*,4*R*)-1-amino-7,7-dimethylbicyclo[2.2.1]heptan-2-one (**3**) [8,9].

Following *General procedure 1*. Prepared from Boc-amine **2** (3.57 g, 14.1 mmol), CH_2Cl_2 (25 mL), CF_3CO_2H (50 mL). The amine **3**, obtained after extraction workup, was used in the following transformation without further purification. Yield: 1.62 g (10.57 mmol, 75%) of white solid. Physical and spectral data of compound **3** were in accordance with the literature data [8,9].

1.3.3 Synthesis of (1*S*,4*R*)-7,7-dimethyl-1-(pyrrolidin-1-yl)bicyclo[2.2.1]heptan-2-one (**4**) [10].

Prepared following a modified literature procedure [6, 10]. To a solution of amino-ketone **3** (1.61 g, 10.51 mmol) in MeCN (30 mL) under Argon, K_2CO_3 (3.244 g, 23.4 mmol) and 1,4-dibromobutane (2.44 g, 11.3 mmol) were added, and the resulting reaction mixture was stirred under reflux for 24 h. Volatile components were evaporated *in vacuo*. Water was added (10 mL) to the residue, and the mixture was extracted with EtOAc (3 × 25 mL). The combined organic phase was washed with NaCl (aq. sat., 15 mL). The organic phase was dried over anhydrous Na_2SO_4, filtered, and volatile components evaporated *in vacuo*. The obtained mixture was purified by CC (Et_3N:EtOAc:petroleum ether = 1:1:40). Fractions containing the pure product **4** were combined and volatile components evaporated *in vacuo*. Yield: 1.22 g (5.889 mmol, 56%) of white solid. Physical and spectral data of compound **4** were in accordance with the literature data [10].

1.3.4. Synthesis of (1*S*,4*R*,*E*)-7,7-dimethyl-1-(pyrrolidin-1-yl)bicyclo[2.2.1]heptan-2-one oxime (**5**).

Prepared following a modified literature procedure [6, 10]. To a solution of amino-oxime **8** (953 mg, 5.66 mmol) in anhydrous MeCN (25 mL) under Argon, K_2CO_3 (1.74 g, 12.6 mmol) and 1,4-dibromobutane (1.32 g, 6.12 mmol) were added, and the resulting reaction mixture was stirred under reflux for 48 h. Volatile components were evaporated *in vacuo.* Water was added (10 mL) to the residue and the mixture was extracted with EtOAc (3 × 25 mL). The combined organic phase was washed with NaCl (aq. sat., 15 mL). The organic phase was dried over anhydrous Na_2SO_4, filtered, and volatile components evaporated *in vacuo*. The obtained mixture was purified by CC (Et_3N:EtOAc:petroleum ether = 1:1:20). Fractions containing the pure product **5** were combined and volatile components evaporated *in vacuo*. Yield: 239 mg (1.075 mmol, 19%) of colorless solid; mp = 130–132°C. $[\alpha]_D^{20}$ = –4.87 (c = 0.25, CH_2Cl_2). EI-HRMS: *m/z* = 223.1804 (MH^+); $C_{13}H_{23}N_2O$ requires: *m/z* = 223.1805 (MH^+). ν_{max} 2943, 2875, 1673, 1478, 1441, 1429, 1387, 1371, 1318, 1192, 1167, 1128, 1107, 1065, 1048, 978, 952, 938, 924, 861, 755, 682, 622 cm^{-1}. ^1H-NMR (500 MHz, $CDCl_3$): δ 1.04 (*s*, 3H, Me); 1.05 (*s*, 3H, Me); 1.23 – 1.33 (*m*, 1H); 1.71 (*t*, *J* = 4.7 Hz, 1H); 1.72 – 1.85 (*m*, 5H); 1.85 – 1.98 (*m*, 1H); 2.04 – 2.14 (*m*, 2H); 2.64 (*dt*, *J* = 3.8; 17.9 Hz, 1H); 2.90 – 2.98 (*m*, 2H); 3.01 – 3.11 (*m*, 2H); 8.89 (br *s*, 1H, OH). ^{13}C-NMR (126 MHz, $CDCl_3$): δ 19.7, 22.6, 24.8, 26.9, 32.6, 32.8, 43.9, 48.7, 49.3, 73.7, 161.

1.3.5. Synthesis of (1*R*,*E*)-2-(3-(hydroxyimino)-2,2-dimethylcyclopentyl)acetonitrile (**6**) [11].

Following *General procedure 2*. Prepared from pyrrolidino-ketone **4** (1.23 g, 5.93 mmol), $NH_2OH·HCl$ (821 mg, 11.8 mmol), pyridine (717 µL, 8.87 mmol), EtOH (25 mL), t = 6 h; purified by CC (1. Et_3N:EtOAc:petroleum ether = 1:1:20 for the elution of nonpolar impurities, 2. Et_3N:EtOAc:petroleum ether = 1:1:10 for the elution of product **6**). Yield: 158 mg (0.945 mmol, 16%) of colorless solid. Physical and spectral data of compound **6** were in accordance with the literature data [11].

1.3.6 Synthesis of *tert*-butyl [(1*S*,4*R*,*E*)-2-(hydroxyimino)-7,7-dimethylbicyclo[2.2.1]heptan-1-yl]carbamate (**7**).

Following *General procedure 2*. Prepared from ketone **2** (1.14 g, 4.50 mmol), $NH_2OH·HCl$ (763 mg, 10.98 mmol), pyridine (666 µL, 8.23 mmol), EtOH (20 mL), t = 10 h; purified by CC (EtOAc:petroleum ether = 1:5). Yield: 592 mg (2.205 mmol, 49%) of colorless solid; mp = 121–123°C. $[\alpha]_D^{20}$ = 0 (c = 0.19, MeOH). EI-HRMS: *m*/*z* = 269.2859 (MH^+); $C_{14}H_{25}N_2O_3$ requires: *m*/*z* = 169.1860 (MH^+); ν_{max} 3340, 3287, 3209, 3078, 2920, 2871, 2852, 1690, 1604, 1510, 1470, 1444, 1432, 1385, 1370, 1311, 1258, 1198, 1163, 1149, 1101, 1092, 1062, 1037, 1017, 943, 928, 863, 781, 730, 685, 621 cm^{-1}. ^1H-NMR (500 MHz, DMSO-d_6): δ 0.79 (*s*, 3H, Me); 0.97 (*s*, 3H, Me); 1.23 – 1.30 (*m*, 1H); 1.38 (*s*, 9H, Boc); 1.71 – 1.82 (*m*, 2H); 1.82 – 1.89 (*m*, 1H); 1.92 (*d*, *J* = 17.7 Hz, 1H); 2.40 (*dt*, *J* = 4.1; 17.6 Hz, 2H); 5.87 (br *s*, 1H, NH); 10.29 (br *s*, 1H, OH). ^{13}C-NMR (126 MHz, DMSO-d_6): δ 18.9, 19.2, 26.5, 28.0, 28.2, 32.0, 40.8, 48.5, 66.7, 77.6, 154.5, 162.0.

1.3.7. Synthesis of (1*S*,4*R*,*E*)-1-amino-7,7-dimethylbicyclo[2.2.1]heptan-2-one oxime (**8**).

Following *General procedure 1*. Prepared from Boc-amine **7** (431 mg, 1.606 mmol), CH_2Cl_2 (8 mL), CF_3CO_2H (8 mL). The amine **8**, obtained after extraction workup, was used in the following transformation without further purification. Yield: 262 mg (1.558 mmol, 97%) of colorless solid; mp = 142–144°C. $[\alpha]_D^{20}$ = +34.9 (c = 0.41, MeOH). EI-HRMS: *m*/*z* = 169.1335 (MH^+); $C_9H_{17}N_2O$ requires: *m*/*z* = 169.1335 (MH^+); ν_{max} 3340, 3287, 3077, 2960, 2948, 2872, 2789, 2748, 1731, 1689, 1605, 1515, 1471, 1444, 1432, 1385, 1370, 1311, 1288, 1258, 1199, 1163, 14149, 1101, 1092, 1062, 1037, 1017, 956, 944, 864, 781, 722, 685, 610 cm^{-1}. ^1H-NMR (500 MHz, DMSO-d_6): δ 0.67 (*s*, 3H, Me); 0.87 (*s*, 3H, Me); 1.16 – 1.25 (*m*, 1H); 1.31 – 1.39 (*m*, 1H); 1.46 (*s*, 2H); 1.66 – 1.74 (*m*, 1H); 1.76 – 1.83 (*m*, 2H); 1.91 (*d*, *J* = 17.6 Hz, 1H); 2.36 (*dt*, *J* = 4.0; 17.6 Hz, 1H); 10.10 (br *s*, 1H, OH). ^{13}C-NMR (126 MHz, DMSO-d_6): δ 17.9, 18.4, 26.5, 32.3, 32.4, 40.7, 47.4, 66.8, 165.0.

1.3.8. Synthesis of (*R*)-2-(2,2-dimethyl-3-oxocyclopentyl)acetonitrile (**9**) [12].

Following *General procedure 3*. Prepared from amino-oxime **8** (107 mg, 0.636 mmol), H_2O (2 mL), K_2CO_3 (97 mg, 0.70 mmol), 1,4-dibromobutane (151.1 mg, 0.70 mmol); purified by CC (1. Et_3N:EtOAc:petroleum ether = 1:1:20 for the elution of nonpolar impurities; 2 Et_3N:EtOAc:petroleum ether = 1:1:5 for the elution of product **9**). Yield: 63 mg (0.413 mmol, 65%) of colorless solid; mp = 56–58°C. $[\alpha]_D^{20}$ = –66.8 (c = 0.17, CH_2Cl_2), lit [12] $[\alpha]_D^{20}$ = 54.6 (c = 0.64, MeOH). EI-HRMS: *m*/*z* = 152.1070 (MH^+); $C_9H_{14}NO$ requires: *m*/*z* = 152.1070 (MH^+). ν_{max} 2964, 2919, 2886, 2871, 2242, 1729, 1690, 1532, 1464, 1406, 1361, 1273, 1248, 1102, 1085, 1050, 1026, 991, 975, 931, 922, 863, 810, 762, 709, 638 cm^{-1}. ^1H-NMR (500 MHz, DMSO-d_6): δ 0.78 (*s*, 3H, Me); 1.00 (*s*, 3H, Me); 1.61 (*dtd*, *J* = 8.8; 10.7; 12.6 Hz, 1H); 2.02 – 2.08 (*m*, 1H); 2.11 – 2.19 (*m*, 1H); 2.19 – 2.28 (*m*, 1H); 2.33 (*ddd*, *J* = 2.4; 8.8; 18.9 Hz, 1H); 2.56 (*dd*, *J* = 8.5; 17.0 Hz, 1H); 2.68 (*dd*, *J* = 6.3; 17.0 Hz, 1H). ^{13}C-NMR (126 MHz, DMSO-d_6): δ 17.0, 17.5, 22.4, 24.3, 35.4, 42.8, 47.0, 119.9, 220.8. ^{13}C NMR data are in accordance with the literature data [12].

1.3.9 Synthesis of (1*S*,2*S*,4*R*)-7,7-dimethyl-1-(pyrrolidin-1-yl)bicyclo[2.2.1]heptan-2-amine (**10**).

Following *General procedure 4*. Prepared from pyrrolidino-oxime **5** (207 mg, 0.931 mmol), *n*PrOH (25 mL); purified by CC (Et_3N:EtOAc:petroleum ether = 1:1:20). The crude compound **10**, obtained after chromatographic workup, was used in the following transformation without further purification. Yield: 109 mg (0.521 mmol, 56%) of colorless oil. EI-HRMS: *m*/*z* = 209.2012 (MH^+); $C_{13}H_{25}N$ requires: *m*/*z* = 209.2012 (MH^+). ^1H-NMR (500 MHz, $CDCl_3$): δ 0.78 (*dd*, *J* = 4.2; 13.0 Hz, 1H); 1.10 (*s*, 3H, Me); 1.12 (*s*, 3H, Me); 1.20 – 1.27 (*m*, 1H); 1.33 – 1.37 (*m*, 1H); 1.58 – 2.08 (*m*, 2H, NH_2); 1.68

– 1.75 (*m*, 4H); 1.78 – 1.86 (*m*, 2H); 1.91 – 1.98 (*m*, 1H); 2.31 – 2.40 (*m*, 1H); 2.87 – 2.99 (*m*, 4H); 3.72 – 3.81 (*m*, 1H). ^{13}C-NMR (126 MHz, $CDCl_3$): δ 21.2, 22.4, 22.8, 24.1, 27.6, 39.5, 45.4, 48.4, 49.6, 52.0, 71.0.

1.3.10. Synthesis of (1*S*,2*R*,4*R*)-7,7-dimethyl-1-(pyrrolidin-1-yl)bicyclo[2.2.1]heptan-2-amine (**11**).

Following *General procedure 5*. Prepared from pyrrolidino-oxime **5** (226 mg, 1.02 mmol), MeOH (60 mL); purified by CC (MeOH:$CHCl_3$:NH_3 (aq. 25%) = 9:90:1). Yield: 106 mg (0.510 mmol, 50%) of colorless oil. $[\alpha]_D^{20}$ –44.1 (c = 0.21, CH_2Cl_2). EI-HRMS: *m*/*z* = 209.2013 (MH^+); $C_{13}H_{25}N$ requires: *m*/*z* = 209.2012 (MH^+). ν_{max} 3372, 3304, 2951, 2874, 2806, 1740, 1680, 1583, 1457, 1386, 1457, 1386, 1368, 1356, 1295, 1278, 1249, 1208, 1168, 1137, 1095, 1033, 952, 913, 858, 826, 634 cm^{-1}. ^{1}H-NMR (500 MHz, $CDCl_3$): δ 1.03 – 1.07 (*m*, 1H); 1.05 (*s*, 3H, Me); 1.13 (*s*, 3H, Me); 1.16 – 1.26 (*m*, 1H); 1.53 – 1.59 (*m*, 1H); 1.67 – 1.76 (*m*, 4H); 1.76 – 1.87 (*m*, 4H); 2.23 – 2.47(*br*, 2H, NH_2); 2.57 – 2.73 (*m*, 4H); 2.98 – 3.09 (*m*, 1H). ^{13}C-NMR (126 MHz, $CDCl_3$): δ 20.7, 22.9, 23.6, 23.9, 26.5, 40.0, 46.2, 47.1, 47.8, 58.6, 70.5.

1.3.11. Synthesis of *tert*-butyl [(1*S*,2*S*,4*R*)-2-amino-7,7-dimethylbicyclo[2.2.1]heptan-1-yl]carbamate (**12**) and *tert*-butyl [(1*S*,2*R*,4*R*)-2-amino-7,7-dimethylbicyclo[2.2.1]heptan-1-yl]carbamate (**13**).

Following *General procedure 5*. Prepared from oxime **7** (566 mg, 2.11 mmol), MeOH (40 mL), Et_3N (1 mL); purified by CC (1. Et_3N:Et_2O = 1:80 for the elution of nonpolar impurities; 2. Et_3N:Et_2O = 1:40 → 1:10 for the elution/separation of products **12** and **13**).

Compound **13**: Elutes first from the column. The crude compound **13**, obtained after chromatographic workup, was used in the following transformation without further purification. Yield: 92 mg (0.3587 mmol, 17%) of colorless solid; mp = 93–94°C. EI-HRMS: *m*/*z* = 255.2066 (MH^+); $C_{14}H_{27}N_2O_2$ requires: *m*/*z* = 255.2067 (MH^+). ν_{max} 3264, 3127, 2942, 2878, 1691, 1464, 1363, 1307, 1250, 1167, 1057, 1006, 857, 775, 7588, 708, 658, 606 cm^{-1}. ^{1}H-NMR (500 MHz, $CDCl_3$): δ 0.94 (s, 3H, Me); 1.02 (*s*, 3H, Me); 1.16 – 1.34 (*m*, 3H); 1.46 (*s*, 9H, Boc); 1.60 – 1.71 (*m*, 2H); 1.73 – 1.92 (*m*, 4H); 3.29 (br *s*, 1H); 4.75 (*s*, 1H, NH). ^{13}C-NMR (126 MHz, $CDCl_3$): δ 19.9, 21.0, 27.2, 28.5, 32.1, 40.2, 42.6, 47.3, 56.8, 65.3, 79.2, 156.0.

Compound **12**: Elutes second from the column. Yield: 403 mg (1.58 mmol, 75%) of colorless solid; mp = 55–56°C. $[\alpha]_D^{20}$ +38.8 (c = 0.08, CH_2Cl_2). EI-HRMS: *m*/*z* = 255.2064 (MH^+); $C_{14}H_{27}N_2O_2$ requires: *m*/*z* = 255.2067 (MH^+). ν_{max} 3371, 3286, 3212, 2939, 2882, 1707, 1591, 1557, 1529, 1458, 1389, 1362, 1607, 1299, 1275, 1244, 1162, 1097, 1085, 1072, 1059, 1038, 1013, 979, 947, 926, 901, 889, 855, 798, 777, 639 cm^{-1}. ^{1}H-NMR (500 MHz, $CDCl_3$): δ 0.84 (*dd*, *J* = 4.4; 13.1 Hz, 1H); 0.97 (*s*, 3H, Me); 0.99 (*s*, 3H, Me); 1.27 – 1.34 (*m*, 1H); 1.43 (*s*, 9H, Boc); 1.52 – 1.67 (*m*, 4H); 1.81 – 1.90 (*m*, 1H); 2.29 – 2.37 (*m*, 1H); 2.39 – 2.48 (*m*, 1H); 3.53 – 3.68 (*m*, 1H); 4.43 (br *s*, 1H). ^{13}C-NMR (126 MHz, $CDCl_3$): δ 11.7, 18.6, 20.3, 24.4, 28.1, 28.4, 39.0, 42.6, 46.3, 49.2, 55.4, 67.0.

1.3.12. Synthesis of *tert*-butyl [(1*S*,2*S*,4*R*)-7,7-dimethyl-2-(pyrrolidin-1-yl)bicyclo[2.2.1]heptan-1-yl]carbamate (**14**).

Following *General procedure 3*. Prepared from amine **12** (284 mg, 1.12 mmol), H_2O (1 mL), K_2CO_3 (170 mg, 1.23 mmol), 1,4-dibromobutane (266 mg, 1.23 mmol); purified by CC (1. Et_3N:EtOAc:petroleum ether = 1:1:40 for the elution of nonpolar impurities; 2. Et_3N:EtOAc:petroleum ether = 1:1:20 for the elution of product **14**). Yield: 321 mg (1.04 mmol, 93%) of white solid; mp = 112–114°C. $[\alpha]_D^{20}$ = +16.3 (c = 0.16, CH_2Cl_2). EI-HRMS: *m*/*z* = 309.2539 (MH^+); $C_{18}H_{33}N_2O_2$ requires: *m*/*z* = 309.2537 (MH^+). ν_{max} 3344, 2966, 2943, 2874, 2798, 1706, 1686, 1533, 1454, 1386, 1365, 1279, 1252, 1168, 1145, 1083, 1066, 1040, 1016, 681, 928, 881, 777, 752, 680, 644 cm^{-1}. ^{1}H-NMR (500 MHz, DMSO-*d*$_6$): δ 0.83 (*s*, 3H, Me); 0.97 (*s*, 3H, Me); 1.08 (*dd*, *J* = 3.8; 12.4 Hz, 1H); 1.10 – 1.19 (*m*, 1H); 1.36 (*s*, 9H, Boc); 1.43 (*t*, *J* = 4.6 Hz, 1H); 1.53 – 1.73 (*m*, 5H); 1.89 – 2.12 (*m*, 3H); 2.40 – 2.47 (*m*, 4H); 3.09 – 3.23 (*m*, 1H); 6.30 (br *s*, 1H, NH). ^{13}C-NMR (126 MHz, DMSO-*d*$_6$): δ 20.0, 20.2, 22.7, 24.6, 27.6, 28.3, 35.8, 41.7, 49.5, 52.1, 62.7, 66.9, 76.9, 154.7.

1.3.13. Synthesis of *tert*-butyl [(1*S*,2*R*,4*R*)-7,7-dimethyl-2-(pyrrolidin-1-yl)bicyclo[2.2.1]heptan-1-yl]carbamate (**15**).

Following *General procedure 3*. Prepared from amine **13** (92 mg, 0.362 mmol), H_2O (1 mL), K_2CO_3 (56 mg, 0.40 mmol), 1,4-dibromobutane (87 mg, 0.40 mmol). The crude compound **15**, obtained after extraction workup, was used in the following transformation without further purification. Yield: 86 mg (0.2787 mmol, 77%) of colorless oil. EI-HRMS: *m/z* = 309.2538 (MH^+); $C_{18}H_{33}N_2O_2$ requires: *m/z* = 309.2537 (MH^+). ^1H-NMR (500 MHz, $CDCl_3$): δ 0.90 (*s*, 3H, Me); 1.12 (*s*, 3H, Me); 1.15 – 1.23 (*m*, 1H); 1.43 (*s*, 9H, Boc); 1.55 – 1.75 (*m*, 6H); 1.75 – 1.86 (*m*, 1H); 1.91 – 2.08 (*m*, 3H); 2.60 (s, 5H); 4.76 (br *s*, 1H, NH). ^{13}C-NMR (126 MHz, $CDCl_3$): δ 19.5, 20.8, 23.6, 27.4, 28.7, 32.9, 36.5, 42.0, 47.9, 53.1, 65.9, 71.1, 78.8, 155.9.

1.3.14. Synthesis of (1*S*,2*S*,4*R*)-7,7-dimethyl-2-(pyrrolidin-1-yl)bicyclo[2.2.1]heptan-1-amine (**16**).

Following *General procedure 1*. Prepared from Boc-amine **14** (284 mg, 0.921 mmol), CH_2Cl_2 (3 mL), CF_3CO_2H (3 mL). The amine **16**, obtained after extraction workup, was used in the following transformation without further purification. Yield: 134 mg (0.645 mmol, 70%) of colorless oil. H-NMR (500 MHz, DMSO-*d*$_6$): δ 0.82 (*s*, 3H, Me); 0.85 (*s*, 3H, Me); 1.14 (*dd*, *J* = 4.1; 12.7 Hz, 1H); 1.17 – 1.28 (*m*, 2H); 1.54 (*t*, *J* = 4.6 Hz, 1H); 1.62 – 1.72 (*m*, 5H); 1.91 – 2.09 (*m*, 2H); 2.28 – 2.41 (*m*, 1H); 2.54 – 2.72 (*m*, 4H); 2.84 – 3.64 (*m*, 2H). ^{13}C-NMR (126 MHz, DMSO-*d*$_6$): δ 17.9, 19.1, 22.6, 26.6, 27.5, 35.4, 41.4, 48.3, 53.6, 66.1, 68.3.

1.3.15. Synthesis of (1*S*,2*R*,4*R*)-7,7-dimethyl-2-(pyrrolidin-1-yl)bicyclo[2.2.1]heptan-1-amine (**17**).

Following *General procedure 1*. Prepared from Boc-amine **15** (86 mg, 0.279 mmol), CH_2Cl_2 (2 mL), CF_3CO_2H (2 mL). The amine **17**, obtained after extraction workup, was used in the following transformation without further purification. Yield: 56 mg (0.268 mmol, 96%) of colorless oil.

1.3.16. Synthesis of (1*R*,4*R*)-7,7-dimethyl-1-(piperidin-1-ylmethyl)bicyclo[2.2.1]heptan-2-one (**20b**) [13].

Following *General procedure 8*. Prepared from 10-iodocamphor (**18**) (1.36 g, 4.89 mmol), K_2CO_3 (1.01 g, 7.31 mmol), DMSO (10 mL), piperidine (7.25 mL, 73.35 mmol); purified by CC (1. Et_3N:EtOAc:petroleum ether = 1:1:30 for the elution on nonpolar impurities; 2. Et_3N:EtOAc:petroleum ether = 1:1:20 for the elution of product **20b**). Yield: 760 mg (3.23 mmol, 66%) of colorless oil. Physical and spectral data for compound **20b** were in accordance with the literature data [13].

1.3.17. Synthesis of (1*S*,4*R*)-7,7-dimethyl-1-(morpholinomethyl)bicyclo[2.2.1]heptan-2-one (**20c**) [14].

Following *General procedure 8*. Prepared from 10-iodocamphor (**18**) (1.64 g, 5.90 mmol), K_2CO_3 (1.22 g, 8.84 mmol), DMSO (10 mL), morpholine (7.74 mL, 88 mmol); purified by CC (1. Et_3N:EtOAc:petroleum ether = 1:1:30 for the elution on nonpolar impurities; 2. Et_3N:EtOAc:petroleum ether = 1:1:20 for the elution of product **20c**). Yield: 840 mg (3.54 mmol, 60%) of colorless oil. $[\alpha]_D^{20}$ = +48.0 (c =0.56, CH_2Cl_2). EI-HRMS: *m/z* = 238.1802 (MH^+); $C_{14}H_{24}NO_2$ requires: *m/z* = 238.1802 (MH^+). ν_{max} 2955, 2888, 2849, 2804, 1737, 1453, 1417, 1277, 1116, 1052, 1005, 997, 977, 864, 799 cm^{-1}. ^1H-NMR (500 MHz, $CDCl_3$): δ 0.94 (*s*, 3H, Me); 1.04 (*s*, 3H, Me); 1.31 – 1.44 (*m*, 2H); 1.83 (*d*, *J* = 18.2 Hz, 1H); 1.93 – 2.05 (*m*, 3H); 2.33 – 2.39 (*m*, 1H); 2.40 (*d*, *J* = 14.2 Hz, 1H); 2.46 – 2.57 (*m*, 5H); 3.63 (*t*, *J* = 4.6 Hz, 4H). ^{13}C-NMR (126 MHz, $CDCl_3$): δ 19.9, 20.7, 26.6, 26.8, 43.5, 43.6, 47.4, 54.5, 55.6, 61.3, 67.3, 218.8.

1.3.18. Synthesis of (1*S*,4*R*)-1-[(dimethylamino)methyl]-7,7-dimethylbicyclo[2.2.1]heptan-2-one (**20d**) [14,15].

To 10-iodocamphor (**18**) (1.80 g, 6.47 mmol) in a high pressure cylinder a solution of $NHMe_2$ in EtOH (10 mL, 33%) was added and the resulting reaction mixture was stirred at 150°C for 7 h. Volatile components were evaporated *in vacuo* and the residue was purified by CC (1. Et_3N:EtOAc:petroleum ether = 1:1:30 for the elution on nonpolar impurities; 2. Et_3N:EtOAc:petroleum ether = 1:1:20 for the elution of product **20d**). Fractions containing the pure product 20d were combined and volatile components evaporated *in vacuo*. Yield: 468 mg (2.39 mmol, 37%) of yellowish oil. $[\alpha]_D^{20}$ = +65.4 (c = 0.46, CH_2Cl_2). EI-HRMS: *m/z* = 196.1694 (MH^+); $C_{12}H_{22}NO$ requires: *m/z* = 196.1696 (MH^+). ν_{max} 2941,

2887, 2818, 2764, 1738, 1453, 1417, 1389, 1372, 1264, 1243, 1152, 1096, 1039, 1016, 998, 842 cm^{-1}. ^{1}H-NMR (500 MHz, $CDCl_3$): δ 0.89 (*s*, 3H, Me); 1.02 (*s*, 3H, Me); 1.32 – 1.41 (*m*, 1H); 1.45 – 1.54 (*m*, 1H); 1.84 (*d*, *J* = 18.2 Hz, 1H); 1.94 – 2.07 (*m*, 3H); 2.27 (*s*, 6H, NMe_2); 2.30 – 2.37 (*m*, 2H); 2.50 (*d*, *J* = 13.7 Hz, 1H). ^{13}C-NMR (126 MHz, $CDCl_3$): δ 20.0, 20.4, 25.8, 27.1, 43.4, 43.6, 47.8, 48.2, 55.4, 61.1, 218.7.

1.3.19. Synthesis of (1*R*,4*R*,*E*)-7,7-dimethyl-1-[(piperidin-1-yl)methyl]bicyclo[2.2.1]heptan-2-one oxime (**21b**).

Following *General procedure 2*. Prepared from amino-ketone **20b** (1.17 g, 4.97 mmol), $NH_2OH{\cdot}HCl$ (691 mg, 9.94 mmol), pyridine (604 μL, 7.46 mmol), EtOH (30 mL), t = 10 h; purified by CC (1. Et_3N:EtOAc:petroleum ether = 1:1:30 for the elution on nonpolar impurities; 2. Et_3N:EtOAc:petroleum ether = 1:1:20 for the elution of product **21b**). Yield: 1.13 g (4.52 mmol, 91%) of colorless solid; mp = 113–116°C. $[\alpha]_D^{20}$ = –10.2 (c = 0.33, CH_2Cl_2). EI-HRMS: *m*/*z* = 251.2115 (MH^+); $C_{15}H_{27}N_2O$ requires: *m*/*z* = 251.2118 (MH^+); ν_{max} 3283, 2932, 2845, 2787, 2756, 1694, 1440, 1307, 1268, 1198, 1168, 1152, 1118, 1039, 973, 926, 854, 788, 726, 662 cm^{-1}. ^{1}H-NMR (500 MHz, $CDCl_3$): δ 0.85 (*s*, 3H, Me); 1.03 (*s*, 3H, Me); 1.18 – 1.27 (*m*, 1H); 1.31 – 1.40 (*m*, 2H); 1.38 – 1.48 (*m*, 1H); 1.46 – 1.56 (*m*, 4H); 1.81 (*t*, *J* = 4.5 Hz, 1H); 1.83 – 1.91 (*m*, 1H); 2.03 (*d*, *J* = 17.8 Hz, 1H); 2.13 (*td*, *J* = 12.1, 4.4 Hz, 1H); 2.33 – 2.61 (*m*, 7H); 8.58 (*s*, 1H, OH). ^{13}C-NMR (126 MHz, $CDCl_3$): δ 20.1, 20.3, 24.3, 26.4, 27.4, 28.7, 33.3, 44.2, 49.3, 55.5, 56.2, 57.1, 169.6.

1.3.20 Synthesis of (1*R*,4*R*,*E*)-7,7-dimethyl-1-(morpholinomethyl)bicyclo[2.2.1]heptan-2-one oxime (**21c**).

Following *General procedure 2*. Prepared from amino-ketone **20c** (700 mg, 2.96), $NH_2OH{\cdot}HCl$ (412 mg, 5.92 mmol), pyridine (360 μL, 4.44 mmol), EtOH (80 mL), t = 10 h; purified by CC (1. Et_3N:EtOAc:petroleum ether = 1:1:30 for the elution on nonpolar impurities; 2. Et_3N:EtOAc:petroleum ether = 1:1:20 for the elution of product **21c**). Yield: 679 mg (2.69 mmol, 91%) of colorless solid; mp = 160–163°C. $[\alpha]_D^{20}$ = –16.9 (c = 0.26, CH_2Cl_2). EI-HRMS: *m*/*z* = 253.1912 (MH^+); $C_{14}H_{25}N_2O_2$ requires: *m*/*z* = 253.1911 (MH^+); ν_{max} 3273, 3150, 2963, 2911, 2845, 2807, 1692, 1451, 1388, 1355, 1298, 1280, 1249, 1201, 1139, 1116, 1087, 1069, 1037, 1008, 947, 924, 876, 584, 803, 737, 687 cm^{-1}. ^{1}H-NMR (500 MHz, $CDCl_3$): δ = 0.87 (*s*, 3H, Me); 1.01 (*s*, 3H, Me); 1.22 – 1.29 (*m*, 1H); 1.49 – 1.57 (*m*, 1H); 1.82 – 1.92 (*m*, 2H); 2.02 – 2.07 (*m*, 2H); 2.39 (*d*, *J* = 14.0 Hz, 1H); 2.47 – 2.60 (*m*, 5H); 2.65 (*d*, *J* = 14.0 Hz, 1H); 3.67 (*h*, *J* = 6.6 Hz, 4H); 8.77 (*s*, 1H, OH). ^{13}C-NMR (126 MHz, $CDCl_3$): δ 19.7, 20.2, 27.3, 29.4, 33.2, 43.9, 49.3, 55.1, 55.8, 56.6, 67.1, 168.9.

1.3.21. Synthesis of (1*R*,4*R*,*E*)-1-[(dimethylamino)methyl]-7,7-dimethylbicyclo[2.2.1]heptan-2-one oxime (**21d**).

Following *General procedure 2*. Prepared from amino-ketone **20d** (520 mg, 2.66 mmol), $NH_2OH{\cdot}HCl$ (370 mg, 5.32 mmol), pyridine (323 μL, 3.99 mmol), EtOH (15 mL), t = 10 h; purified by CC (1. Et_3N:EtOAc:petroleum ether = 1:1:30 for the elution on nonpolar impurities; 2. Et_3N:EtOAc:petroleum ether = 1:1:20 for the elution of product **21d**). Yield: 526 mg (2.50 mmol, 94%) of colorless solid; mp = 97–98°C. $[\alpha]_D^{20}$ = –23.2 (c = 0.22, CH_2Cl_2). EI-HRMS: *m*/*z* = 211.1806 (MH^+); $C_{12}H_{23}N_2O$ requires: *m*/*z* = 211.1805 (MH^+); ν_{max} 3278, 3208, 3086, 2947, 2884, 2871, 2855, 2825, 2791, 2772, 1740, 1453, 1381, 1370, 1339, 1299, 1252, 1193, 1183, 1164, 1099, 999, 929, 841, 641 cm^{-1}. ^{1}H-NMR (500 MHz, $CDCl_3$): δ (*s*, 3H, Me); 0.98 (*s*, 3H, Me); 1.22 – 1.29 (*m*, 1H); 1.61 (*ddd*, *J* = 4.0; 9.3; 13.1 Hz, 1H); 1.80 – 1.91 (*m*, 2H); 1.98 – 2.07 (*m*, 2H); 2.29 (*s*, 6H); 2.37 (*d*, *J* = 48.6 Hz, 1H); 2.55 (*dt*, *J* = 4.0; 17.7 Hz, 1H); 2.62 (*d*, *J* = 13.7 Hz, 1H); 7.55 – 9.54 (br *s*, 1H). ^{13}C-NMR (126 MHz, $CDCl_3$): δ 19.6, 20.1, 27.5, 28.8, 33.2, 43.8, 48.3, 49.4, 55.2, 57.6, 168.7.

1.3.22. Synthesis of a mixture of (1*R*,2*S*,4*R*)-7,7-dimethyl-1-[(piperidin-1-yl)methyl]bicyclo[2.2.1]heptan-2-amine (**22b**) and (1*R*,2*R*,4*R*)-7,7-dimethyl-1-[(piperidin-1-yl)methyl]bicyclo[2.2.1]heptan-2-amine (**23b**).

Following *General procedure 4*. Prepared from amino-oxime **21b** (720 mg, 2.85 mmol), *n*PrOH (15 mL); purified by extraction and used in the following transformation. Yield: 646 mg (2.71 mmol, 95%) of yellowish oil; *endo*:*exo* = 4.8:1. $[\alpha]_D^{20}$ = –10.5 (c = 0.60, CH_2Cl_2). ν_{max} 3368, 3339, 3284, 2984, 2929, 2875, 2853, 2782, 2755, 2730, 2669, 1648, 1577, 1455, 1442, 1385, 1366, 1352, 1315, 1296, 1270, 1260, 1241, 1148, 1106, 1049, 1039, 983, 898, 860, 827, 793, 773, 744, 618 cm^{-1}.

^{1}H-NMR (500 MHz, $CDCl_3$) for **22b**: δ 0.67 (*dd*, *J* = 12.9; 4.2 Hz, 1H); 0.86 (*s*, 3H, Me); 0.89 (*s*, 3H, Me); 1.16 – 1.25 (*m*, 1H); 1.30 – 1.42 (*m*, 3H); 1.43 – 1.58 (*m*, 5H); 1.69 – 1.81 (*m*, 1H); 1.87 – 2.05 (*m*, 2H); 2.04 – 2.13 (*m*, 1H); 2.20 (*d*, *J* = 13.4 Hz, 1H); 2.15 – 2.27 (*m*, 1H); 2.15 – 2.32 (*m*, 2H); 2.36 (*d*, *J* = 13.3 Hz, 1H); 2.37 – 2.58 (*m*, 2H); 3.33 (*ddd*, *J* = 2.0; 4.3; 10.6 Hz, 1H). ^{13}C-NMR (126 MHz, $CDCl_3$) for 22b: δ 19.1, 20.7, 24.2, 25.4, 26.8, 28.9, 39.2, 45.0, 49.2, 51.3, 56.2, 56.9, 62.2.

^{1}H-NMR (500 MHz, $CDCl_3$) for **23b**: δ 0.80 (*s*, 3H, Me); 1.05 (*s*, 3H, Me); 2.62 (*d*, *J* = 13.2 Hz, 1H); 3.05 (*dd*, *J* = 5.1; 8.8 Hz, 1H). ^{13}C-NMR (126 MHz, $CDCl_3$) for 23b: δ 21.4, 24.4, 26.6, 26.8, 27.8, 31.1, 35.3, 39.8, 45.1, 48.1, 56.6, 58.6, 59.1.

1.3.23. Synthesis of (1*R*,2*S*,4*R*)-7,7-dimethyl-1-[(morpholin-4-yl)methyl]bicyclo[2.2.1]heptan-2-amine (**22c**) and (1*R*,2*R*,4*R*)-7,7-dimethyl-1-[(morpholin-4-yl)methyl]bicyclo[2.2.1]heptan-2-amine (**23c**).

Following *General procedure 4*. Prepared from amino-oxime **21c** (677 mg, 2.70 mmol), *n*PrOH (15 mL); purified by CC (Et_3N:EtOAc:petroleum ether = 1:1:20). Yield: 480 mg (2.03 mmol, 75%) of yellowish oil; *endo*:*exo* = 4.6:1. $[\alpha]_D^{20}$ = –10.5 (c = 0.60, CH_2Cl_2). EI-HRMS: *m*/*z* = 237.2327 (MH^+); $C_{15}H_{29}N_2$ requires: *m*/*z* = 237.2325 (MH^+). ν_{max} 3368, 3293, 2946, 2873, 2850, 2804, 1579, 1454, 1386, 1356, 1337, 1317, 1298, 1277, 1260, 1206, 1155, 1069, 1035, 989, 903, 863, 802, 732, 621 cm^{-1}.

^{1}H NMR (500 MHz, $CDCl_3$) for **22c**: δ 0.68 (*dd*, *J* = 4.2; 12.9 Hz, 1H); 0.88 (*s*, 3H, Me); 0.90 (*s*, 3H, Me); 1.17 – 1.27 (*m*, 1H); 1.38 (*tdd*, *J* = 2.1; 4.5; 12.3 Hz, 1H); 1.51 (*t*, *J* = 4.6 Hz, 1H); 1.71 – 1.79 (*m*, 1H); 1.74 – 1.82 (*m*, 2H); 2.04 – 2.14 (*m*, 1H); 2.18 – 2.28 (*m*, 1H); 2.28 (*d*, *J* = 13.3 Hz, 1H); 2.30 – 2.40 (*m*, 2H); 2.41 (*d*, *J* = 13.3 Hz, 1H); 2.50 – 2.65 (*m*, 2H); 3.36 (*ddd*, *J* = 2.0; 4.2; 10.6 Hz, 1H); 3.60 – 3.71 (*m*, 4H). ^{13}C NMR (126 MHz, $CDCl_3$) for 22c: δ 19.1, 20.8, 25.4, 28.9, 39.3, 44.9, 49.4, 51.4, 55.8, 56.2, 62.3, 67.5.

^{1}H NMR (500 MHz, $CDCl_3$) for **23c**: δ 0.81 (*s*, 3H, Me); 1.07 (*s*, 3H, Me); 1.27 – 1.33 (*m*, 1H); 1.65 (*t*, *J* = 4.3 Hz, 1H); 2.16 (*d*, *J* = 13.0 Hz, 1H); 2.70 (*d*, *J* = 13.1 Hz, 1H); 3.07 (*dd*, *J* = 5.1; 8.8 Hz 1H). ^{13}C NMR (126 MHz, $CDCl_3$) for 23c: δ 20.7, 21.4, 27.8, 35.2, 39.8, 45.0, 48.1, 51.3, 55.6, 58.5, 58.8, 67.3.

1.3.24. Synthesis of (1*R*,2*S*,4*R*)-1-[(dimethylamino)methyl]-7,7-dimethylbicyclo[2.2.1]heptan-2-amine (**22d**) and (1*R*,2*R*,4*R*)-1-[(dimethylamino)methyl]-7,7-dimethylbicyclo[2.2.1]heptan-2-amine (**23d**).

Following *General procedure 4*. Prepared from amino-oxime **21d** (525 mg, 2.496 mmol), *n*PrOH (15 mL); purified by extraction and used in the following transformation. Yield: 402 mg (2.05 mmol, 82%) of yellowish oil; *endo*:*exo* = 2.8:1. $[\alpha]_D^{20}$ = –10.5 (c = 0.60, CH_2Cl_2). EI-HRMS: *m*/*z* = 197.2012 (MH^+); $C_{12}H_{25}N_2$ requires: *m*/*z* = 197.2012 (MH^+). ν_{max} 3367, 3293, 2940, 2875, 2816, 2765, 2723, 1667, 1578, 1454, 1388, 1368, 1300, 1255, 1222, 1174, 1155, 1095, 1022, 990, 837, 743, 614 cm^{-1}.

^{1}H-NMR (500 MHz, $CDCl_3$) for **22d**: δ 0.67 (*dd*, *J* = 4.3; 12.9 Hz, 1H); 0.86 (*s*, 3H, Me); 0.89 (*s*, 3H, Me); 1.18 – 1.25 (*m*, 1H); 1.36 – 1.43 (*m*, 1H); 1.49 (*t*, *J* = 4.6 Hz, 1H); 1.66 – 1.92 (*m*, 2H); 1.72 – 1.79 (*m*, 1H); 2.11 (*d*, *J* = 12.9 Hz, 1H); 2.13 – 2.19 (*m*, 1H); 2.21 (*s*, 6H); 2.22 – 2.26 (*m*, 1H); 2.46 (*d*, *J* = 13.0 Hz, 1H); 3.36 (*ddd*, *J* = 2.0; 4.2; 10.7 Hz, 1H). ^{13}C-NMR (126 MHz, $CDCl_3$) for 22d: δ 19.1, 20.8, 25.2, 28.9, 39.3, 44.9, 48.1, 48.3, 51.5, 56.6, 63.2.

^{1}H-NMR (500 MHz, $CDCl_3$) for **23d**: δ 0.81 (*s*, 3H, Me); 1.05 (*s*, 3H, Me); 2.00 (*d*, *J* = 13.0 Hz, 1H); 1.3.27 (*s*, 6H); 2.73 (*d*, *J* = 13.0 Hz, 1H); 3.09 (*dd*, *J* = 5.1; 8.8 Hz, 1H). ^{13}C-NMR (126 MHz, $CDCl_3$) for 23d: δ 20.7, 21.5, 27.8, 34.4, 40.0, 45.1, 48.1, 48.3 49.5, 58.7, 59.0.

2.25. Synthesis of (1*S*,4*R*,*E*)-1-(iodomethyl)-7,7-dimethylbicyclo[2.2.1]heptan-2-one oxime (**24**) [16].

Prepared following a modified literature procedure [4]. To a solution of 10-iodocamphor (**18**) [1] (1 g, 3.60 mmol) in anhydrous EtOH (25 mL), $NH_2OH{\cdot}HCl$ (500 mg, 7.20 mmol) and pyridine (437 μL, 5.40 mmol) were added, and the resulting reaction mixture was stirred under reflux for 16 h.

Volatile components were evaporated *in vacuo*, the residue was suspended in H_2O (15 mL) followed by extraction with Et_2O (3 × 50 mL). The combined organic phase was washed with NaCl (aq. sat., 30 mL). The organic phase was dried over anhydrous Na_2SO_4, filtered, and volatile components evaporated *in vacuo*. The residue was purified by CC (1. EtOAc:petroleum ether = 1:20 for the elution of nonpolar impurities; 2. EtOAc:petroleum ether = 1:7 for the elution of product **24**). Fractions containing the pure product **24** were combined and volatile components evaporated *in vacuo*. Yield: 739 mg (2.52 mmol, 70%) of white solid; mp = 140–144°C. $[\alpha]_D^{20}$ = –95.9 (c = 0.34, CH_2Cl_2). EI-HRMS: *m/z* = 294.0349 (MH^+); $C_{10}H_{17}INO$ requires: *m/z* = 294.0343 (MH^+). ν_{max} 3353, 2969, 2953, 2916, 2873, 1676, 1423, 1398, 1384, 1366, 1318, 1296, 1277, 1247, 1215, 1168, 963, 940, 922, 879, 861, 773, 739, 688, 667, 611 cm^{-1}. ^1H-NMR (500 MHz, $CDCl_3$): δ 0.86 (*s*, 3H, Me); 1.01 (*s*, 3H, Me); 1.24 – 1.32 (*m*, 1H); 1.68 – 1.77 (*m*, 1H); 1.84 – 1.91 (*m*, 1H); 1.95 (*td*, *J* = 3.7; 12.0 Hz, 1H); 2.06 (*t*, *J* = 4.4 Hz, 1H); 2.11 (*d*, *J* = 18.0 Hz, 1H); 2.58 – 2.70 (*m*, 1H); 3.24 (*d*, *J* = 10.1, 1H), 3.36 (*d*, *J* = 10.2, 1H).; 8.56 (br *s*, 1H). ^{13}C-NMR (126 MHz, $CDCl_3$): δ 3.4, 19.6, 19.8, 26.9, 33.1, 33.7, 45.2, 50.2, 54.2, 167.1.

1.3.26. Synthesis of (1*R*,4*R*,*E*)-1-(azidomethyl)-7,7-dimethylbicyclo[2.2.1]heptan-2-one oxime (**25**).

To a solution of iodo-oxime **24** (294 mg, 1 mmol) in in anhydrous DMSO (2.5 mL) NaN_3 (195 mg, 3 mmol) was added, and the resulting reaction mixture was stirred at 75°C for 16 h. To the reaction mixture H_2O (25 mL) was added, followed by extracted with EtOAc (3 × 40 mL). The combined organic phase was washed with H_2O (2 mL) and NaCl (25 mL). The organic phase was dried over anhydrous Na_2SO_4, filtered, and volatile components evaporated *in vacuo*. The residue was purified by CC (1. EtOAc:petroleum ether = 1:10 for the elution of nonpolar impurities; 2. EtOAc:petroleum ether = 1:5 for the elution of product **25**). Fractions containing the pure product **25** were combined and volatile components evaporated *in vacuo*. Yield: 142 mg (0.680 mmol, 68%) of white solid; mp = 68–71°C. $[\alpha]_D^{20}$ = –22.2 (c = 0.254, CH_2Cl_2). EI-HRMS: *m/z* = 209.1397 (MH^+); $C_{10}H_{17}N_4O$ requires: *m/z* = 209.1397 (MH^+). ν_{max} 3281, 2964, 2929, 2874, 2092, 1688, 1433, 1391, 1373, 1351, 1293, 1280, 1249, 1222, 1198, 1081, 999, 925, 899, 886, 854, 833, 821, 716, 659 cm^{-1}. ^1H-NMR (500 MHz, $CDCl_3$): δ 0.91 (*s*, 3H, Me); 1.02 (*s*, 3H, Me); 1.26 – 1.34 (*m*, 1H); 1.53 – 1.62 (*m*, 1H); 1.86 – 2.00 (*m*, 3H); 2.09 (*d*, *J* = 18.0 Hz, 1H); 2.60 (*dt*, *J* = 3.6; 18.0 Hz, 1H); 3.47 (*d*, *J* = 12.8 Hz, 1H); 3.58 (*d*, *J* = 12.8 Hz, 1H); 8.72 – 8.84 (*m*, 1H). ^{13}C-NMR (126 MHz, $CDCl_3$): δ 9.5, 20.0, 27.1, 29.0, 33.0, 44.5, 48.8, 50.3, 55.4, 167.4.

1.3.27. Synthesis of (1*R*,4*R*,*E*)-1-(aminomethyl)-7,7-dimethylbicyclo[2.2.1]heptan-2-one oxime (**26**).

To a solution of azido-oxime **25** (352 mg, 1.69 mmol) in anhydrous THF (10 mL) PPh_3 (666 mg, 2.54 mmol) was added, and the resulting reaction mixture was stirred under reflux. After the disappearance of the starting azide, as judged by TLC analysis, H_2O (5 mL) was added and the reaction mixture was stirred under reflux for 2 h. Volatile components were evaporated *in vacuo* and the residue was purified by CC ($MeOH:CHCl_3$+1% NH_3 (25% aq.) = 1:10+1%). Fractions containing the pure product **26** were combined and volatile components evaporated *in vacuo*. Yield: 283 mg (1.555 mmol, 92%) of white solid; mp = 81–85°C. $[\alpha]_D^{20}$ = –74.4 (c = 0.25, MeOH). EI-HRMS: *m/z* = 183.1493 (MH^+); $C_{10}H_{19}N_2O$ requires: *m/z* = 183.1492 (MH^+). ν_{max} 3406, 3090, 2934, 2874, 1616, 1555, 1508, 1474, 1436, 1390, 1370, 1333, 1309, 1285, 1262, 1250, 1210, 1153, 1089, 1063, 979, 945, 932, 910, 890, 838, 780, 752, 692 cm^{-1}. ^1H-NMR (500 MHz, $CDCl_3$): δ 0.87 (*s*, 3H, Me); 0.92 (*s*, 3H, Me); 1.25 – 1.32 (*m*, 1H); 1.62 – 1.70 (*m*, 1H); 1.70 – 1.78 (*m*, 1H); 1.81 – 1.92 (*m*, 2H); 2.02 (*d*, *J* = 17.7 Hz, 1H); 2.55 (*dt*, *J* = 3.9; 17.8 Hz, 1H); 2.66 (*d*, *J* = 13.4 Hz, 1H); 3.03 (*d*, *J* = 13.3 Hz, 1H); 3.44 (*s*, 1H); 4.34 (br *s*, 2H). ^{13}C-NMR (126 MHz, $CDCl_3$): δ 19.0, 20.2, 27.1, 29.1, 33.0, 41.0, 44.6, 48.3, 55.7, 168.0.

1.3.28. Synthesis of *tert*-butyl {[(1*R*,4*R*,*E*)-2-{[(*tert*-butoxycarbonyl)oxy]imino}-7,7-dimethylbicyclo[2.2.1]heptan-1-yl]methyl}carbamate (**27**) and *tert*-butyl {[(1*R*,4*R*,*E*)-2-(hydroxyimino)-7,7-dimethylbicyclo[2.2.1]heptan-1-yl]methyl}carbamate (**28**).

Following *General procedure 7*. Prepared from amino-oxime **26** (927 mg, 5.09 mmol), Boc_2O (1.67 g, 7.63 mmol), Et_3N (1.42 mL, 10.18 mmol); purified/separated by CC (Et_3N:EtOAc:petroleum ether = 1:1:20).

Compound **27**: Elutes first from the column. Yield: 428 mg (1.12 mmol, 22%) of colorless solid; mp = 84–88°C. $[\alpha]_D^{20}$ = –36.3 (c = 0.19, CH_2Cl_2). EI-HRMS: *m*/*z* = 383.2538 (MH^+); $C_{20}H_{35}N_2O_5$ requires: *m*/*z* = 383.2540 (MH^+). ν_{max} 3438, 2974, 2888, 1769, 1712, 1504, 1455, 1392, 1367, 1269, 1240, 1150, 1049, 984, 962, 918, 872, 829, 802, 778, 755, 705 cm^{-1}. ^{1}H-NMR (500 MHz, $CDCl_3$): δ 0.89 (*s*, 3H, Me); 1.03 (*s*, 3H, Me); 1.21 – 1.31 (*m*, 1H); 1.43 (*s*, 9H, Boc); 1.41 – 1.60 (*m*, 1H); 1.55 (*s*, 9H, Boc); 1.81 – 1.95 (*m*, 3H); 2.14 (*d*, *J* = 18.3 Hz, 1H); 2.61 – 2.71 (*m*, 1H); 3.32 – 3.52 (*m*, 2H); 5.32 – 5.40 (*m*, 1H). ^{13}C-NMR (126 MHz, $CDCl_3$): δ 18.8, 20.1, 26.7, 28.0, 28.6, 29.1, 34.7, 38.5, 44.6, 48.7, 56.8, 79.1, 83.5, 152.3, 156.3, 175.7.

Compound **28**: Elutes second from the column. Yield: 906 mg (3.21 mmol, 63%) of white solid; mp = 146–151°C. $[\alpha]_D^{20}$ = –37.1 (c = 0.26, CH_2Cl_2). EI-HRMS: *m*/*z* = 283.2017 (MH^+); $C_{15}H_{27}N_2O_3$ requires: *m*/*z* = 283.2016 (MH^+). ν_{max} 3400, 2962, 2884, 2249, 1691, 1507, 1453, 1430, 1391, 1366, 1271, 1165, 1135, 1113, 967, 943, 909, 864, 730, 646, 619 cm^{-1}. ^{1}H-NMR (500 MHz, $CDCl_3$): δ 0.87 (*s*, 3H, Me); 1.00 (*s*, 3H, Me); 1.21 – 1.29 (*m*, 1H); 1.43 (*s*, 9H, Boc); 1.78 – 1.92 (*m*, 3H); 2.03 (*d*, *J* = 17.9 Hz, 1H); 2.52 – 2.62 (*m*, 2H); 3.22 – 3.45 (*m*, 2H); 5.34 – 5.44 (*m*, 1H); 7.53 (br *s*, 1H). ^{13}C-NMR (126 MHz, $CDCl_3$): δ 18.9, 20.1, 27.0, 28.6, 29.4, 33.1, 39.2, 44.7, 48.3, 55.3, 79.0, 156.4, 169.5.

1.3.29. Synthesis of *tert*-butyl {[(1*R*,2*R*,4*R*)-2-amino-7,7-dimethylbicyclo[2.2.1]heptan-1-yl]methyl}carbamate (**29**) and *tert*-butyl {[(1*R*,2*S*,4*R*)-2-amino-7,7-dimethylbicyclo[2.2.1]heptan-1-yl]methyl}carbamate (**30**).

Following *General procedure 5*. Prepared from *N*,*O*-di-Boc-oxime **27** (1.32 g, 3.45 mmol), MeOH (40 mL), Et_3N (1 mL); purified/separated by CC (Et_3N:Et_2O = 1:40).

Compound **29**: Elutes first from the column. Yield: 314 mg (1.17 mmol, 34%) of white solid; mp = 77–81°C. $[\alpha]_D^{20}$ –13.9 (c = 0.52, CH_2Cl_2). EI-HRMS: *m*/*z* = 269.2225 (MH^+); $C_{15}H_{29}N_2O_2$ requires: *m*/*z* = 269.2224 (MH^+). ν_{max} 3377, 3295, 3206, 2955, 2936, 2920, 2878, 1700, 1686, 1558, 1479, 1459, 1387, 1362, 1289, 1251, 1167, 1135, 1096, 1040, 1019, 964, 909, 869, 769 cm^{-1}. ^{1}H-NMR (500 MHz, $CDCl_3$): δ 0.80 (*s*, 3H, Me); 0.98 (*s*, 3H, Me); 1.00 – 1.10 (*m*, 2H); 1.37 (*s*, 11H, Boc and NH_2); 1.44 – 1.54 (*m*, 2H); 1.60 – 1.67 (*m*, 2H); 1.71 (*dd*, *J* = 8.9; 13.0 Hz, 1H); 2.78 – 2.85 (*m*, 1H); 2.96 (*dd*, *J* = 5.0; 13.6 Hz, 1H); 3.45 (*dd*, *J* = 7.1; 13.6 Hz, 1H); 4.82 (br *s*, 1H). ^{13}C-NMR (126 MHz, $CDCl_3$): δ 71.6, 72.1, 77.9, 79.4, 83.6, 90.6, 92.1, 96.8, 97.7, 102.6, 108.9, 130.0, 207.3.

Compound **30**: Elutes second from the column. Yield: 481 mg (1.794 mmol, 52%) of white oil. $[\alpha]_D^{20}$ +27.5 (c = 0.48, CH_2Cl_2). EI-HRMS: *m*/*z* = 269.2223 (MH^+); $C_{15}H_{29}N_2O_2$ requires: *m*/*z* = 269.2224 (MH^+). ν_{max} 3367, 3306, 2934, 2878, 1693, 1508, 1455, 1390, 1364, 1247, 1169, 1131, 1060, 1025, 606, 868, 730, 645 cm^{-1}. ^{1}H-NMR (500 MHz, $CDCl_3$): δ 0.61 (*dd*, *J* = 4.2; 13.0 Hz, 1H); 0.83 (*s*, 6H, 2×Me); 0.93 (*t*, *J* = 7.2 Hz, 1H); 1.24 – 1.31 (*m*, 1H); 1.34 (*s*, 9H, Boc); 1.41 (br *s*, 2H, NH_2); 1.48 (*t*, *J* = 4.7 Hz, 1H); 1.63 – 1.81 (*m*, 2H); 2.19 – 2.27 (*m*, 1H); 2.93 – 3.02 (*m*, 1H); 3.11 – 3.18 (*m*, 1H); 3.18 – 3.23 (*m*, 1H); 6.18 (br *s*, 1H). ^{13}C-NMR (126 MHz, $CDCl_3$): δ 19.0, 20.1, 23.4, 28.2, 28.4, 41.9, 43.0, 45.8, 48.2, 50.2, 55.1, 78.4, 156.5.

1.3.30. Synthesis of *tert*-butyl {[(1*R*,2*R*,4*R*)-7,7-dimethyl-2-(pyrrolidin-1-yl)bicyclo[2.2.1]heptan-1-yl]methyl}carbamate (**31**).

Following *General procedure 3*. Prepared from amine **29** (205 mg, 0.764 mmol), H_2O (1.5 mL), K_2CO_3 (116 mg, 0.840 mmol), 1,4-dibromobutane (181.4 mg, 0.840 mmol); purified by CC (Et_3N:EtOAc:petroleum ether = 1:1:40). Yield: 239 mg (0.741 mmol, 97%) of colorless oil. $[\alpha]_D^{20}$ = –47.0 (c = 0.25, CH_2Cl_2). EI-HRMS: *m*/*z* = 323.2695 (MH^+); $C_{19}H_{35}N_2O_2$ requires: *m*/*z* = 323.2693 (MH^+). ν_{max} 3468, 3360, 2951, 2932, 2875, 2796, 1714, 1498, 1454, 1389, 1364, 1347, 1301, 1285, 1240, 1167, 1082, 908, 880, 779 cm^{-1}. ^{1}H-NMR (500 MHz, $CDCl_3$): δ 0.90 (*s*, 3H, Me); 1.00 – 1.09 (*m*, 2H); 1.13 (*s*, 3H, Me); 1.43 (*s*, 9H, Boc); 1.48 – 1.54 (*m*, 1H); 1.57 (*t*, *J* = 4.4 Hz, 1H); 1.64 – 1.76 (*m*, 5H); 1.87 (*s*, 1H); 1.97 – 2.04 (*m*, 1H); 2.42 (*dd*, *J* = 5.8; 9.1 Hz, 1H); 2.47 – 2.63 (*m*, 4H); 3.21 (*dd*, *J* = 2.5; 13.8 Hz, 1H); 3.47 (*dd*, *J* = 6.9; 13.8 Hz, 1H); 5.96 (br *s*, 1H). ^{13}C-NMR (126 MHz, $CDCl_3$): δ 20.9, 21.3, 23.3, 26.9, 28.7, 34.3, 35.8, 42.2, 46.3, 47.3, 51.1, 53.5, 73.3, 78.5, 156.5.

1.3.31. Synthesis of *tert*-butyl {[(1*R*,2*S*,4*R*)-7,7-dimethyl-2-(pyrrolidin-1-yl)bicyclo[2.2.1]heptan-1-yl]methyl}carbamate (**32**).

Following *General procedure 3*. Prepared from amine **30** (450 mg, 1.68 mmol), H_2O (2 mL), K_2CO_3 (256 mg, 1.85 mmol), 1,4-dibromobutane (400 mg, 1.85 mmol); purified by CC (Et_3N:EtOAc:petroleum ether = 1:1:40). Yield: 519 mg (1.61 mmol, 96%) of colorless oil. $[\alpha]_D^{20}$ = +31.1 (c = 0.33, CH_2Cl_2). EI-HRMS: *m/z* = 323.2695 (MH^+); $C_{19}H_{35}N_2O_2$ requires: *m/z* = 323.2693 (MH^+). ν_{max} 3467, 3360, 3304, 2963, 2934, 2875, 2789, 1700, 1505, 1453, 1389, 1364, 1342, 1244, 1166, 1125, 1077, 1029, 677, 942, 918, 867, 777, 734 cm^{-1}. ^{1}H-NMR (500 MHz, $CDCl_3$): δ 0.91 (*s*, 3H, Me); 0.95 (*s*, 3H, Me); 1.20 – 1.28 (*m*, 2H); 1.28 – 1.38 (*m*, 1H); 1.42 (*s*, 9H, Boc); 1.54 (*t*, *J* = 4.6 Hz, 1H); 1.64 – 1.78 (*m*, 5H); 1.92 – 2.00 (*m*, 2H); 2.50 – 2.65 (*m*, 4H); 2.78 – 2.88 (*m*, 1H); 3.2 (dd, *J*=13.6, 5.5, 1H), 3.3 (dd, *J*=13.7, 5.0, 1H); 5.55 (br *s*, 1H). ^{13}C-NMR (126 MHz, $CDCl_3$): δ 19.7, 20.5, 23.5, 25.5, 28.3, 28.6, 33.2, 42.7, 45.7, 48.7, 51.3, 53.4, 66.0, 78.7, 156.3.

1.3.32. Synthesis of [(1*R*,2*R*,4*R*)-7,7-dimethyl-2-(pyrrolidin-1-yl)bicyclo[2.2.1]heptan-1-yl]methanamine (**33**).

Following *General procedure 1*. Prepared from Boc-amine **31** (217 mg, 0.673 mmol), CH_2Cl_2 (5 mL), CF_3CO_2H (5 mL); purified by CC (Et_3N:Et_2O = 1:40). Yield: 148 mg (0.666 mmol, 99%) of colorless oil. $[\alpha]_D^{20}$ = –62.0 (c = 0.26, CH_2Cl_2). EI-HRMS: *m/z* = 223.21729 (MH^+); $C_{14}H_{27}N_2$ requires: *m/z* = 223.21688 (MH^+); ν_{max} 3382, 3300, 2947, 2929, 2874, 2786, 1737, 1725, 1682, 1577, 1453, 1387, 1347, 1294, 1200, 1177, 1124, 1105, 1044, 1026, 944, 829, 817, 799 cm^{-1}. ^{1}H-NMR (500 MHz, $CDCl_3$): δ 0.89 (*s*, 3H, Me); 1.03 – 1.19 (*m*, 2H); 1.13 (*s*, 3H, Me); 1.44 – 1.54 (*m*, 2H); 1.59 (*t*, *J* = 4.4 Hz, 1H); 1.63 – 1.76 (*m*, 5H); 1.93 – 2.01 (*m*, 1H); 2.22 (*s*, 2H); 2.41 (*dd*, *J* = 5.6; 8.8 Hz, 1H); 2.49 – 2.68 (*m*, 4H); 2.81 (*d*, *J* = 13.7 Hz, 1H); 3.02 (*d*, *J* = 13.6 Hz, 1H). ^{13}C-NMR (126 MHz, $CDCl_3$): δ 20.9, 21.4, 23.4, 27.1, 31.1, 33.5, 36.0, 42.1, 46.2, 47.3, 53.8, 72.8.

1.3.33. Synthesis of [(1*R*,2*S*,4*R*)-7,7-dimethyl-2-(pyrrolidin-1-yl)bicyclo[2.2.1]heptan-1-yl]methanamine (**34**).

Following *General procedure 1*. Prepared from Boc-amine **32** (390 mg, 1.21 mmol), CH_2Cl_2 (5 mL), CF_3CO_2H (5 mL); purified by CC (Et_3N:Et_2O = 1:40). Yield: 250 mg (1.125 mmol, 93%) of colorless oil. $[\alpha]_D^{20}$ = +27.3 (c = 0.34, CH_2Cl_2). EI-HRMS: *m/z* = 223.21703 (MH^+); $C_{14}H_{27}N_2$ requires: *m/z* = 223.21688 (MH^+); ν_{max} 3449, 3394, 3326, 3295, 2942, 2873, 2782, 1571, 1457, 1386, 1365, 1341, 1311, 1294, 1194, 1143, 1108, 1057, 918, 883, 787 cm^{-1}. ^{1}H-NMR (500 MHz, $CDCl_3$): δ 0.92 (*s*, 3H, Me); 1.05 (*s*, 3H, Me); 1.21 (*dd*, *J* = 4.1; 12.6 Hz, 1H); 1.24 – 1.33 (*m*, 2H); 1.51 (*t*, *J* = 4.5 Hz, 1H); 1.66 – 1.83 (*m*, 7H); 1.89 – 1.97 (*m*, 1H); 2.00 – 2.09 (*m*, 1H); 2.57 – 2.66 (*m*, 4H); 2.82 – 2.91 (*m*, 3H). ^{13}C-NMR (126 MHz, $CDCl_3$): δ 20.9, 21.0, 23.4, 25.7, 28.5, 35.6, 43.7, 46.2, 49.3, 52.9, 53.8, 65.4.

1.3.34. Synthesis of 2-((1*S*,4*R*,*E*)-2-(hydroxyimino)-7,7-dimethylbicyclo[2.2.1]heptan-1-yl)acetonitrile (**35**).

To a solution of iodo-oxime **24** (3.16 g, 10.8 mmol) in anhydrous DMSO (50 mL), KCN (2.11 g, 32.4 mmol) and tetrabutylammonium bromide (27 mg, 0.084 mmol) were added, and the resulting reaction mixture was stirred at 75°C for 16 h. To the reaction mixture H_2O (500 mL) was added, followed by extraction with EtOAc (3 × 250 mL EtOAc). The combined organic phase was washed with H_2O (75 mL) and NaCl (aq. sat., 75 mL). The organic phase was dried over anhydrous Na_2SO_4, filtered, and volatile components evaporated *in vacuo*. The residue was purified by CC (1. EtOAc:petroleum ether = 1:20 for the elution of nonpolar impurities; 2. EtOAc:petroleum ether = 1:7 for the elution of product **35**). Fractions containing the pure product **35** were combined and volatile components evaporated *in vacuo*. Yield: 1.68 g (8.748 mmol, 81%) of white solid; isomer ratio: 81:19 (in $CDCl_3$); mp = 118–121°C. $[\alpha]_D^{20}$ = –40.2 (c = 0.24, CH_2Cl_2). EI-HRMS: *m/z* = 193.1337 (MH^+); $C_{11}H_{17}N_2O$ requires: *m/z* = 193.1335 (MH^+). ν_{max} 3456, 2968, 2951, 2937, 2882, 2253, 1675, 1476, 1458, 1444, 1429, 1413, 1391, 1371, 1317, 1306, 1289, 1239, 1198, 1178, 1089, 1063, 1045, 986, 926, 903, 856, 776, 722, 699, 650, 624, 608 cm^{-1}. ^{1}H-NMR (500 MHz, $CDCl_3$) for the major isomer: δ 0.86 (*s*, 3H, Me); 1.01 (*s*, 3H, Me); 1.24 – 1.31 (*m*, 1H); 1.62 – 1.76 (*m*, 1H); 1.83 – 2.16 (*m*, 4H); 2.60 – 2.64 (*m*, 1H); 3.24 (*d*, *J* = 10.2 Hz, 1H); 3.36 (*d*, *J* = 10.1 Hz, 1H); 8.52 (*s*, 1H, OH). ^{1}H-NMR (126 MHz, $CDCl_3$) for the minor isomer: δ 0.90 (*s*, 3H, Me); 1.08 (*s*, 3H, Me); 1.35 (*ddd*, *J* = 4.0; 9.4; 13.3 Hz, 1H); 2.42 (*d*, *J* = 17.1 Hz, 1H); 8.08 (*s*, 1H,

OH). ^{13}C-NMR (126 MHz, $CDCl_3$) for the major isomer: δ 3.42, 19.61, 19.84, 26.92, 33.08, 33.69, 45.22, 50.25, 54.16, 118.15, 167.15. ^{13}C-NMR (126 MHz, $CDCl_3$) for the minor isomer: δ 15.83, 19.16, 19.58, 26.99, 30.47, 32.75, 44.18, 48.98, 51.91, 166.65.

1.3.35. Synthesis of (1*S*,4*R*,*E*)-1-(2-aminoethyl)-7,7-dimethylbicyclo[2.2.1]heptan-2-one oxime (**36**).

To a solution of nitrile **35** (278 mg, 1.45 mmol) in anhydrous Et_2O (10 mL) $LiAlH_4$ (4.34 mL, 1 M in Et_2O) was added dropwise at room temperature. The resulting reaction mixture was stirred at room temperature for 16 h. The excess of $LiAlH_4$ was carefully quenched with NaOH (2 M in H_2O), followed by the addition of H_2O (5 mL), Et_2O (50 mL), and anhydrous Na_2SO_4 (2.5 g, 14.1 mmol). The resulting mixture was stirred at room temperature for 30 minutes followed by careful decanting of the organic phase. To the aqueous residue fresh portion of Et_2O (50 mL) was introduced followed by stirring for 10 minutes at room temperature and decanting. The extraction procedure was repeated twice more. The combined organic phase was dried over anhydrous Na_2SO_4, filtered, and volatile components evaporated *in vacuo*. The residue was purified by CC (MeOH:$CHCl_3$+NH_3 (25% aq.) = 16:83:1). Fractions containing the pure product **36** were combined and volatile components evaporated *in vacuo*. Yield: 157 mg (8.748 mmol, 55%) of white solid; isomer ratio: 2:1 (in $CDCl_3$); mp = 65–70°C. $[\alpha]_D^{20}$ = –57.0 (c = 0.20, CH_2Cl_2). EI-HRMS: *m*/*z* = 197.1649 (MH^+); $C_{11}H_{21}N_2O$ requires: *m*/*z* = 197.1648 (MH^+). ν_{max} 3356, 3290, 2943, 2875, 1671, 1592, 1472, 1452, 1431, 1386, 1370, 1198, 1094, 1057, 943, 926, 844, 695, 625 cm^{-1}. ^{1}H-NMR (400 MHz, $CDCl_3$) for the major isomer: δ 0.81 (*s*, 3H, Me); 0.89 (*s*, 3H, Me); 1.00 – 1.14 (*m*, 1H); 1.17 – 1.29 (*m*, 1H); 1.41 – 1.59 (*m*, 2H); 1.61 – 1.89 (*m*, 4H); 1.98 (*d*, *J* = 17.7 Hz, 1H); 2.52 (*d*, *J* = 17.4 Hz, 1H); 2.63 – 2.78 (*m*, 2H); 3.11 – 3.22 (*m*, 1H). ^{1}H-NMR (400 MHz, $CDCl_3$) for the minor isomer δ: 0.97 (*s*, 3H, Me); 2.35 (*s*, 1H), 2.79 – 2.88 (*m*, 1H). ^{13}C-NMR (126 MHz, $CDCl_3$) for the major isomer: δ 19.0, 19.8, 27.4, 29.6, 31.8, 33.0, 38.5, 43.7, 48.8, 53.6, 168.0. ^{13}C-NMR (126 MHz, $CDCl_3$) for the minor isomer: δ 20.28, 21.16, 27.21, 30.65, 38.62, 40.83, 44.76, 47.28, 50.29, 57.76.

1.3.36. Synthesis of (1*S*,4*R*,*E*)-7,7-dimethyl-1-[2-(pyrrolidin-1-yl)ethyl]bicyclo[2.2.1]heptan-2-one oxime (**37**).

Following *General procedure 3*. Prepared from amino-oxime **36** (199 mg, 1.01 mmol), H_2O (1.5 mL), K_2CO_3 (126 mg, 0.91 mmol), 1,4-dibromobutane (197 mg, 0.91 mmol); purified by CC (Et_3N:Et_2O = 1:25). Yield: 238 mg (1.075 mmol, 94%) of colorless solid; mp = 56–59°C. $[\alpha]_D^{20}$ = –22.4 (c = 0.11, CH_2Cl_2). EI-HRMS: *m*/*z* = 251.2117 (MH^+); $C_{15}H_{27}N_2O$ requires: *m*/*z* = 251.2118 (MH^+). ν_{max} 3468, 3401, 2936, 2875, 1637, 1561, 1443, 1376, 1316, 1201, 1158, 1127, 1049, 933, 818, 775, 710, 621 cm^{-1}. ^{1}H-NMR (500 MHz, $CDCl_3$): δ 0.76 (*s*, 3H, Me); 0.88 (*s*, 3H, Me); 1.19 – 1.28 (*m*, 1H); 1.43 (*td*, *J* = 3.9; 12.8 Hz, 1H); 1.50 – 1.60 (*m*, 1H); 1.61 – 1.71 (*m*, 1H); 1.73 – 1.87 (*m*, 6H); 1.85 – 1.94 (*m*, 1H); 1.94 (*d*, *J* = 17.5 Hz, 1H); 2.20 (*td*, *J* = 3.9; 11.9 Hz, 1H); 2.45 – 2.60 (*m*, 3H); 2.60 – 2.68 (*m*, 2H); 3.39 – 3.50 (*m*, 1H); 10.55 (br *s*, 1H). ^{13}C-NMR (126 MHz, $CDCl_3$): δ 19.2, 19.8, 23.6, 26.7, 27.6, 29.8, 32.8, 43.9, 48.9, 53.2, 53.4, 54.1, 167.5.

1.3.37. Synthesis of (1*S*,2*S*,4*R*)-7,7-dimethyl-1-[2-(pyrrolidin-1-yl)ethyl]bicyclo[2.2.1]heptan-2-amine (**38**) and (1*S*,2*R*,4*R*)-7,7-dimethyl-1-[2-(pyrrolidin-1-yl)ethyl]bicyclo[2.2.1]heptan-2-amine (**39**).

Following *General procedure 5*. Prepared from pyrrolidino-oxime **37** (193 mg, 0.771 mmol), MeOH (50 mL), Et_3N (1 mL); purified by CC (1. MeOH:$CHCl_3$+1% NH_3 (25% aq.) = 1:20+1% for the elution of nonpolar impurities; 2. MeOH:$CHCl_3$+1% NH_3 (25% aq.) = 5:94:1 → 16:93:1 for the elution/separation of products **38** and **39**).

Compound **38**: Elutes first from the column. Yield: 26 mg (0.108 mmol, 14%) of colorless oil. $[\alpha]_D^{20}$ = –33.9 (c = 0.29, CH_2Cl_2). EI-HRMS: *m*/*z* = 237.2326 (MH^+); $C_{15}H_{29}N_2$ requires: *m*/*z* = 237.2325 (MH^+). ν_{max} 3378, 2935, 2878, 1579, 1450, 1371, 1267, 1200, 1158, 1136, 1122, 1075, 1054, 1029, 991, 938, 818, 781, 711, 635 cm^{-1}. ^{1}H-NMR (500 MHz, $CDCl_3$): δ 0.88 (*s*, 3H, Me); 0.91 (*s*, 3H, Me); 0.72 – 1.05 (*m*, 1H); 1.17 – 1.29 (*m*, 1H); 1.30 – 1.39 (*m*, 1H); 1.44 – 1.51 (*m*, 1H); 1.52 – 1.61 (*m*, 2H); 1.68 – 1.88 (*m*, 7H); 2.23 – 2.32 (*m*, 1H); 2.33 – 2.40 (*m*, 1H); 2.43 – 2.57 (*m*, 4H); 2.57 – 2.66 (*m*, 1H); 3.24 (br *s*, 1H), 3.80 – 4.33 (*m*, 1H). ^{13}C-NMR (126 MHz, $CDCl_3$): δ 18.96, 20.59, 23.32, 24.30, 28.18, 29.50, 38.71, 45.23, 49.51, 51.15, 53.07, 53.75, 55.57.

Compound **39**: Elutes second from the column. Yield: 55 mg (0.231 mmol, 30%) of colorless oil. $[\alpha]_D^{20}$ = –67.4 (c = 0.54, CH_2Cl_2). EI-HRMS: *m/z* = 237.2328 (MH^+); $C_{15}H_{29}N_2$ requires: *m/z* = 237.2325 (MH^+). ν_{max} 3383, 2936, 2878, 2798, 1631, 1576, 1460, 1377, 1323, 1311, 1295, 1205, 1148, 1069, 883, 814, 795, 750, 636, 625 cm^{-1}. ^{1}H-NMR (500 MHz, $CDCl_3$): δ 0.81 (*s*, 3H, Me); 1.00 (*s*, 3H, Me); 1.05 – 1.14 (*m*, 2H); 1.38 – 1.53 (*m*, 2H); 1.55 – 1.64 (*m*, 1H); 1.64 – 1.72 (*m*, 2H); 1.73 – 1.89 (*m*, 6H); 2.47 (*td*, *J* = 11.3, 5.4, 1H), 2.55 (*td*, *J* = 11.5, 5.1, 1H); 2.59 – 2.68 (*m*, 4H); 2.77 (br *s*, 2H); 2.86 (*dd*, *J* = 5.0; 8.9 Hz, 1H). ^{13}C-NMR (126 MHz, $CDCl_3$): δ 20.34, 21.10, 23.40, 25.73, 27.15, 33.05, 40.39, 44.90, 47.37, 50.41, 52.56, 54.20, 57.47.

1.3.38 Synthesis of *tert*-butyl {2-[(1*S*,4*R*,*E*)-2-(hydroxyimino)-7,7-dimethylbicyclo-[2.2.1]heptan-1-yl]ethyl}carbamate (**40**) and *tert*-butyl {2-[(1*S*,4*R*,*E*)-2-[{(*tert*-butoxycarbonyl)oxy]imino}-7,7-dimethylbicyclo[2.2.1]heptan-1-yl]ethyl}carbamate (**41**).

Following *General procedure 7*. Prepared from amine **36** (655 mg, 3.34 mmol), Boc_2O (729 g, 3.34 mmol), Et_3N (466 µL, 3.34 mmol); purified/separated by CC (Et_3N:EtOAc:petroleum ether = 1:1:20).

Compound **41**: Elutes first from the column. Yield: 172 mg (0.434 mmol, 13%) of colorless oil. $[\alpha]_D^{20}$ = –23.2 (c = 0.16, CH_2Cl_2). ν_{max} 3465, 3351, 2956, 2933, 2881, 1692, 1504, 1455, 1389, 1365, 1052, 1002, 920, 856, 778, 731, 646 cm^{-1}. ^{1}H-NMR (500 MHz, $CDCl_3$): δ 0.83 (*s*, 3H, Me); 0.89 (*s*, 3H, Me); 1.09 – 1.19 (*m*, 1H); 1.19 – 1.34 (*m*, 1H); 1.44 (*s*, 18H, 2×Boc); 1.51 – 1.65 (*m*, 2H); 1.70 (*s*, 2H); 1.86 (*t*, *J* = 11.3 Hz, 1H); 3.01 – 3.25 (*m*, 2H); 3.69 (br *s*, 1H, NH); 4.47 – 4.68 (*m*, 1H); 4.73 (br *s*, 1H, NH). ^{13}C-NMR (126 MHz, $CDCl_3$): δ 20.4, 20.8, 27.1, 27.5, 28.5, 28.5, 32.4, 37.5, 39.8, 44.6, 47.9, 50.4, 55.8, 78.8, 79.3, 155.5, 156.0.

Compound **40**: Elutes first from the column. Yield: 634 mg (0.434 mmol, 64%) of colorless semi-solid. $[\alpha]_D^{20}$ = –25.7 (c = 0.27, CH_2Cl_2). EI-HRMS: *m/z* = 297.2174 (MH^+); $C_{16}H_{29}N_2O_3$ requires: *m/z* = 297.2173 (MH^+). ν_{max} 3298, 2961, 2878, 1687, 1510, 1452, 1390, 1365, 1290, 1242, 1247, 1168, 1060, 982, 941, 923, 847, 779, 732 cm^{-1}. ^{1}H-NMR (500 MHz, $CDCl_3$): δ 0.82 (*s*, 3H, Me); 0.91 (*s*, 3H, Me); 1.22 – 1.30 (*m*, 1H); 1.45 (*s*, 9H, Boc); 1.41 – 1.58 (*m*, 2H); 1.66 – 1.80 (*m*, 2H); 1.82 – 1.91 (*m*, 2H); 2.02 (*d*, *J* = 17.9 Hz, 1H); 2.49 – 2.61 (*m*, 1H); 3.13 – 3.36 (*m*, 2H); 5.51 (br *s*, 1H, NH); 7.54 (br *s*, 1H, OH). ^{13}C-NMR (126 MHz, $CDCl_3$): δ 19.1, 19.8, 27.4, 27.5, 28.7, 29.2, 33.1, 37.9, 43.8, 49.3, 54.0, 79.0, 156.2, 170.2.

1.3.39. Synthesis of *tert*-butyl {2-[(1*S*,2*R*,4*R*)-2-amino-7,7-dimethylbicyclo[2.2.1]heptan-1-yl]ethyl}carbamate (**42**) and *tert*-butyl {2-[(1*S*,2*S*,4*R*)-2-amino-7,7-dimethylbicyclo[2.2.1]heptan-1-yl]ethyl}carbamate (**43**).

Following *General procedure 5*. Prepared from pyrrolidino-oxime **40** (600 mg, 2.02 mmol), MeOH (60 mL), Et_3N (1 mL); purified/separated by CC (1) Et_3N:Et_2O = 1:80 for the elution of nonpolar impurities; 2. Et_3N:Et_2O = 1:40 → 1:10 for the elution/separation of products **42** and **43**).

Compound **42**: Elutes first from the column. Yield: 365 mg (1.29 mmol, 64%) of colorless oil. $[\alpha]_D^{20}$ = –40.9 (c = 0.16, CH_2Cl_2). EI-HRMS: *m/z* = 283.2384 (MH^+); $C_{16}H_{31}N_2O_2$ requires: *m/z* = 283.2380 (MH^+). ν_{max} 3340, 2948, 2932, 2877, 1691, 1515, 1480, 1454, 1388, 1365, 1277, 1249, 1170, 1093, 1063, 999, 981, 951, 870, 846, 779 cm^{-1}. ^{1}H-NMR (500 MHz, $CDCl_3$): δ 0.80 (*s*, 3H, Me); 0.97 (*s*, 3H, Me); 1.04 – 1.16 (*m*, 2H); 1.29 – 1.38 (*m*, 1H); 1.44 (*s*, 9H, Boc); 1.41 – 1.52 (*m*, 1H); 1.52 – 1.63 (*m*, 3H); 1.63 – 1.72 (*m*, 2H); 1.73 – 1.80 (*m*, 2H); 2.87 (*dd*, *J* = 4.9; 8.8 Hz, 1H); 3.03 – 3.23 (*m*, 2H); 5.50 (br *s*, 1H, NH). ^{13}C-NMR (126 MHz, $CDCl_3$): δ 20.5, 21.2, 26.9, 27.2, 28.6, 33.0, 38.0, 41.6, 45.0, 47.6, 50.5, 57.7, 79.0, 156.3.

Compound **43**: Elutes second from the column. Yield: 177 mg g (0.626 mmol, 31%) of colorless oil. $[\alpha]_D^{20}$ = +60.23 (c = 0.44, CH_2Cl_2). EI-HRMS: *m/z* = 283.2385 (MH^+); $C_{16}H_{31}N_2O_2$ requires: *m/z* = 283.2380 (MH^+). ν_{max} 3350, 2760, 2932, 2877, 1695, 1518, 1455, 1390, 1365, 1309, 1290, 1269, 1248, 1172, 1092, 1024, 984, 942, 871, 778, 759 cm^{-1}. ^{1}H-NMR (500 MHz, $CDCl_3$): δ 0.74 (*dd*, *J* = 3.9; 12.9 Hz, 1H); 0.86 (*s*, 3H, Me); 0.87 (*s*, 3H, Me); 1.16 – 1.24 (*m*, 1H); 1.32 – 1.39 (*m*, 1H); 1.44 (*s*, 9H, Boc); 1.47 – 1.56 (*m*, 3H); 1.58 (*t*, *J* = 4.7 Hz, 1H); 1.67 – 1.83 (*m*, 3H); 2.29 – 2.38 (*m*, 1H); 2.89 – 3.00 (*m*, 1H); 3.12 (*ddd*, *J* = 2.0; 3.8; 10.8 Hz, 1H); 3.30 – 3.40 (*m*, 1H); 7.06 (br *s*, 1H, NH). ^{13}C-NMR (126 MHz, $CDCl_3$): δ 18.9, 20.5, 23.6, 28.6, 28.7, 31.1, 38.0, 42.7, 45.5, 49.9, 51.5, 55.7, 78.4, 156.5.

1.3.40. Synthesis of *tert*-butyl (2-((1*S*,2*R*,4*R*)-7,7-dimethyl-2-(pyrrolidin-1-yl)bicyclo[2.2.1]heptan-1-yl)ethyl)carbamate (**44**).

Following *General procedure 3*. Prepared from diamine **42** (124 mg, 0.439 mmol), H_2O (2 mL), K_2CO_3 (66 mg, 0.480 mmol), 1,4-dibromobutane (104 mg, 0.480 mmol); the crude compound **44**, isolated by extraction, was used in the following transformation without further purification. Yield: 96 mg (0.285 mmol, 65%) of colorless oil. EI-HRMS: *m*/*z* = 337.2851 (MH^+); $C_{20}H_{37}N_2O_2$ requires: *m*/*z* = 337.2850 (MH^+). ^{1}H-NMR (500 MHz, $CDCl_3$): δ 0.83 (*s*, 3H, Me); 0.98 (*s*, 3H, Me); 1.06 – 1.13 (*m*, 1H); 1.13 – 1.21 (*m*, 1H); 1.44 (*s*, 9H, Boc); 1.36 – 1.52 (*m*, 1H); 1.61 – 1.78 (*m*, 8H); 1.85 – 1.93 (*m*, 1H); 2.51 – 2.61 (*m*, 5H); 2.65 – 2.72 (*m*, 1H); 3.06 – 3.16 (*m*, 1H); 3.16 – 3.25 (*m*, 1H); 6.61 (br *s*, 1H, NH).

1.3.41. Synthesis of *tert*-butyl {2-[(1*S*,2*S*,4*R*)-7,7-dimethyl-2-(pyrrolidin-1-yl)bicyclo[2.2.1]heptan-1-yl]ethyl}carbamate (**45**).

Following *General procedure 3*. Prepared from diamine **43** (175 mg, 0.620 mmol), H_2O (2 mL), K_2CO_3 (94 mg, 0.680 mmol), 1,4-dibromobutane (147 mg, 0.680 mmol); purified by CC (Et_3N:EtOAc:petroleum ether = 1:1:40). Yield: 136 mg (0.403 mmol, 65%) of colorless oil. $[\alpha]_D^{20}$ = +43.9 (c = 0.17, CH_2Cl_2). EI-HRMS: *m*/*z* = 337.2850 (MH^+); $C_{20}H_{37}N_2O_2$ requires: *m*/*z* = 337.2850 (MH^+). ν_{max} 3341, 3148, 2932, 1875, 2799, 1709, 1693, 1525, 1454, 1399, 1364, 1342, 1309, 1291, 1268, 1248, 1170, 1107, 1058, 1044, 1021, 984, 952, 917, 869, 777, 759 cm^{-1}. ^{1}H-NMR (500 MHz, $CDCl_3$): δ 0.82 (*s*, 3H, Me); 0.90 (*s*, 3H, Me); 1.17 – 1.26 (*m*, 2H); 1.31 – 1.40 (*m*, 1H); 1.44 (*s*, 10H, 9H of Boc, 1H of CH_2); 1.56 (*dt*, *J* = 4.6; 14.4 Hz, 1H); 1.62 – 1.66 (*m*, 1H); 1.66 – 1.83 (*m*, 6H); 1.83 – 1.92 (*m*, 1H); 2.62 – 2.78 (*m*, 4H); 2.79 – 2.88 (*m*, 1H); 3.02 – 3.09 (*m*, 1H); 3.33 – 3.42 (*m*, 1H); 8.15 (br *s*, 1H, NH). ^{13}C-NMR (126 MHz, $CDCl_3$): δ 19.3, 20.1, 24.1, 25.5, 28.1, 28.2, 28.7, 30.8, 38.4, 44.8, 49.5, 51.6, 52.7, 66.8, 78.1, 156.9.

1.3.42. Synthesis of 2-[(1*S*,2*R*,4*R*)-7,7-dimethyl-2-(pyrrolidin-1-yl)bicyclo[2.2.1]heptan-1-yl]ethan-1-amine (**46**).

Following *General procedure 1*. Prepared from Boc-amine **44** (108 mg, 0.321 mmol), CH_2Cl_2 (5 mL), CF_3CO_2H (5 mL). The amine **46**, obtained after extraction workup, was used in the following transformation without further purification. Yield: 75 mg (0.318 mmol, 99%) of colorless oil.

1.3.43. Synthesis of 2-((1*S*,2*S*,4*R*)-7,7-dimethyl-2-(pyrrolidin-1-yl)bicyclo[2.2.1]heptan-1-yl)ethan-1-amine (**47**).

Following *General procedure 1*. Prepared from Boc-amine **45** (133 mg, 0.395 mmol), CH_2Cl_2 (5 mL), CF_3CO_2H (5 mL); purified by CC (MeOH:$CHCl_3$+1% NH_3 (25% aq.) = 1:2+1%). The amine **47**, obtained after extraction workup, was used in the following transformation without further purification. Yield: 74 mg (0.312 mmol, 79%) of colorless oil. ν_{max} 3341, 3148, 2932, 2875, 2799, 1709, 1693, 1525, 1454, 1388, 1364, 1342, 1309, 1291, 1268, 1248, 1170, 1107, 1058, 1044, 1021, 984, 952, 917, 869, 776, 759 cm^{-1}.

1.3.44. Synthesis of 1-[3,5-bis(trifluoromethyl)phenyl]-3-[(1*S*,2*R*,4*R*)-7,7-dimethyl-1-(pyrrolidin-1-yl)bicyclo[2.2.1]heptan-2-yl]thiourea (**48**).

Following *General procedure 6*. Prepared from diamine **11** (96 mg, 0.461 mmol), Et_2O (3 mL), 1-isothiocyanato-3,5-bis(trifluoromethyl)benzene (82 μL, 0.44 mmol, 98%); purified by CC (Et_3N:EtOAc:petroleum ether = 1:1:20). Yield: 164 mg (0.341 mmol, 74%) of colorless solid; mp = 171–176°C. $[\alpha]_D^{20}$ = –7.7 (c = 0.34, CH_2Cl_2). CHN analysis for $C_{22}H_{27}F_6N_3S$ requires: C, 55.10; H, 5.68; N, 8.76 and found: C, 55.07; H, 5.73; N, 8.57. EI-HRMS: *m*/*z* = 480.1899 (MH^+); $C_{22}H_{28}F_6N_3S$ requires: *m*/*z* = 480.1903 (MH^+). ν_{max} 3133, 2965, 2876, 1620, 1503, 1462, 1371, 1273, 1175, 1139, 1108, 947, 876, 847, 808, 797, 735, 704, 681, 645, 624 cm^{-1}. ^{1}H-NMR (500 MHz, DMSO-*d*$_6$): δ 1.05 (*s*, 3H, Me); 1.07 (*s*, 3H, Me); 1.16 – 1.30 (*m*, 2H); 1.50 – 1.58 (*m*, 1H); 1.60 – 1.70 (*m*, 4H); 1.72 – 1.92 (*m*, 3H); 1.94 – 2.02 (*m*, 1H); 2.44 – 2.60 (*m*, 4H); 3.87 – 3.98 (*m*, 1H); 7.72 (*s*, 1H, 1H of Ar); 7.99 (*d*, *J* = 4.1 Hz, 1H, NH); 8.40 (*s*, 2H, 2H of Ar); 10.67 (br *s*, 1H, NH). ^{13}C-NMR (126 MHz, DMSO-*d*$_6$): δ 20.0, 21.5, 23.0, 23.1, 25.8, 39.3, 45.1, 46.9, 47.4, 60.5, 69.4, 115.7, 120.9, 123.3 (*q*, *J* = 272.8 Hz), 130.2 (*q*, *J* = 32.7 Hz), 142.0, 178.2.

1.3.45. Synthesis of 1-(3,5-bis(trifluoromethyl)phenyl)-3-((1*S*,2*S*,4*R*)-7,7-dimethyl-1-(pyrrolidin-1-yl)bicyclo[2.2.1]heptan-2-yl)thiourea (**49**).

Following *General procedure 6*. Prepared from diamine **10** (95 mg, 0.456 mmol), Et_2O (5 mL), 1-isothiocyanato-3,5-bis(trifluoromethyl)benzene (80 μL, 0.43 mmol, 98%); purified by CC (1. Et_3N:EtOAc:petroleum ether = 1:1:10 for the elution of nonpolar impurities; 2. Et_3N:EtOAc:petroleum ether = 1:1:1 for the elution of product **49**). Yield: 164 mg (0.342 mmol, 75%) of colorless solid; mp = 59–64°C. $[\alpha]_D^{20}$ = –35.5 (c = 0.25, CH_2Cl_2). CHN analysis for $C_{22}H_{27}F_6N_3S$ requires: C, 55.10; H, 5.68; N, 8.76 and found: C, 54.34; H, 5.78; N, 8.53. EI-HRMS: *m*/*z* = 480.1898 (MH^+); $C_{22}H_{28}F_6N_3S$ requires: *m*/*z* = 480.1903 (MH^+). ν_{max} 3251, 2960, 2879, 1610, 1537, 1512, 1470, 1380, 1348, 1274, 1170, 1125, 993, 964, 884, 847, 809, 755, 725, 701, 680 cm^{-1}. ^{1}H-NMR (500 MHz, DMSO-d_6): δ 0.95 – 1.01 (*m*, 1H); 0.98 (*s*, 3H, Me); 1.10 (*s*, 3H, Me); 1.29 – 1.36 (*m*, 1H); 1.43 – 1.49 (*m*, 1H); 1.54 – 1.61 (*m*, 4H); 1.79 – 2.02 (*m*, 3H); 2.31 – 2.42 (*m*, 1H); 2.72 – 2.85 (*m*, 4H); 5.18 – 5.27 (*m*, 1H); 7.70 (*s*, 1H, 1H of Ar); 8.28 (*d*, *J* = 9.1 Hz, 1H, NH); 8.34 (*s*, 2H, 2H of Ar); 10.03 (br *s*, 1H, NH). ^{13}C-NMR (126 MHz, DMSO-d_6): δ 20.8, 21.4, 23.7, 26.1, 27.1, 36.5, 39.9, 43.9, 47.6, 48.9, 51.6, 70.5, 115.6, 123.29 (*q*, *J* = 273 Hz), 130.15 (*q*, *J* = 33 Hz), 142.1, 179.0.

1.3.46. Synthesis of 1-(3,5-bis(trifluoromethyl)phenyl)-3-((1*S*,2*R*,4*R*)-7,7-dimethyl-2-(pyrrolidin-1-yl)bicyclo[2.2.1]heptan-1-yl)thiourea (**50**).

Following *General procedure 6*. Prepared from diamine **17** (55 mg, 0.264 mmol), Et_2O (2.5 mL), 1-isothiocyanato-3,5-bis(trifluoromethyl)benzene (63 μL, 0.340 mmol, 98%); purified by CC (Et_3N:EtOAc:petroleum ether = 1:1:30). Yield: 98 mg (0.203 mmol, 77%) of colorless solid; mp = 123–126°C. $[\alpha]_D^{20}$ = –13.3 (c = 0.32, CH_2Cl_2). EI-HRMS: *m*/*z* = 480.1898 (MH^+); $C_{22}H_{28}F_6N_3S$ requires: *m*/*z* = 480.1903 (MH^+). ν_{max} 2963, 1671, 1542, 1474, 1385, 1335, 1274, 1201, 1167, 1119, 1000, 973, 916, 880, 831, 799, 720, 698, 676 cm^{-1}. ^{1}H-NMR (500 MHz, DMSO-d_6): δ 0.93 (*s*, 3H, Me); 1.16 – 1.22 (*m*, 1H); 1.30 (*s*, 3H, Me); 1.49 – 1.67 (*m*, 6H); 1.70 – 1.83 (*m*, 2H); 1.92 – 2.03 (*m*, 1H); 2.43 – 2.80 (*m*, 5H); 2.88 – 2.99 (*m*, 1H); 7.55 (br *s*, 1H, NH); 7.72 (*s*, 1H, 1H of Ar); 8.37 (*s*, 2H, 2H of Ar); 10.36 (br *s*, 1H). ^{13}C-NMR (126 MHz, DMSO-d_6): δ 19.2, 20.4, 23.0, 27.3, 33.7, 36.8, 41.4, 48.7, 51.8, 68.7, 69.2, 115.8, 121.2, 123.3 (*q*, *J* = 273 Hz), 130.1 (*q*, *J* = 33 Hz), 142.0, 180.9.

1.3.47. Synthesis of 1-(3,5-bis(trifluoromethyl)phenyl)-3-((1*S*,2*S*,4*R*)-7,7-dimethyl-2-(pyrrolidin-1-yl)bicyclo[2.2.1]heptan-1-yl)thiourea (**51**).

Following *General procedure 6*. Prepared from diamine **16** (122 mg, 0.586 mmol), Et_2O (2.5 mL), 1-isothiocyanato-3,5-bis(trifluoromethyl)benzene (106 μL, 0.570 mmol, 98%); purified by CC (1. Et_3N:Et_2O:petroleum ether = 1:6:25 for the elution of nonpolar impurities; 2. Et_3N:Et_2O = 1:5 for the elution of product **51**). Yield: 211 mg (0.440 mmol, 75%) of colorless solid; mp = 89–99°C. $[\alpha]_D^{20}$ = –22.7 (c = 0.41, CH_2Cl_2). EI-HRMS: *m*/*z* = 480.1899 (MH^+); $C_{22}H_{28}F_6N_3S$ requires: *m*/*z* = 480.1903 (MH^+). ν_{max} 3409, 2963, 2884, 2838, 1659, 1612, 1564, 1488, 1472, 1381, 1273, 1256, 1167, 1122, 979, 881, 847, 808, 774, 723, 700, 679 cm^{-1}. ^{1}H-NMR (500 MHz, DMSO-d_6): δ 1.03 (*s*, 3H, Me); 1.12 (*s*, 3H, Me); 1.37 – 1.46 (*m*, 1H); 1.47 – 1.74 (*m*, 7H); 1.84 – 1.97 (*m*, 1H); 2.01 – 2.11 (*m*, 1H); 2.48 – 2.53 (*m*, 1H); 2.74 – 2.86 (*m*, 2H); 2.89 – 3.05 (*m*, 2H); 3.49 – 3.60 (*m*, 1H); 7.22 (br s, 1H, NH); 7.79 (*s*, 1H, 1H of Ar); 8.11 (*s*, 2H, 2H of Ar); 14.28 (br s, 1H, NH). ^{13}C-NMR (126 MHz, DMSO-d_6): δ 17.9, 18.5, 23.5, 25.8, 26.4, 27.2, 41.8, 49.1, 51.4, 66.3, 71.2, 116.8, 124.1, 123.27 (*q*, *J* = 272.8 Hz), 130.02 (*q*, *J* = 32.8 Hz), 143.0, 183.0.

1.3.48. Synthesis of 1-(3,5-bis(trifluoromethyl)phenyl)-3-((1*R*,2*S*,4*R*)-7,7-dimethyl-1-(pyrrolidin-1-ylmethyl)bicyclo[2.2.1]heptan-2-yl)thiourea (**52**).

Following *General procedure 6*. Prepared from diamine **22a** (157 mg, 0.706 mmol), Et_2O (3 mL), 1-isothiocyanato-3,5-bis(trifluoromethyl)benzene (125 μL, 0.670 mmol, 98%); purified by CC (Et_3N:Et_2O:petroleum ether = 1:25:4). Yield: 296 mg (0.600 mmol, 85%) of colorless solid; mp = 87–92°C. $[\alpha]_D^{20}$ = –79.3 (c = 0.16, CH_2Cl_2). CHN analysis for $C_{23}H_{29}F_6N_3S$ requires: C, 55.97; H, 5.92; N, 8.51 and found: C, 56.23; H, 6.20; N, 8.26. EI-HRMS: *m*/*z* = 494.2060 (MH^+); $C_{23}H_{30}F_6N_3S$ requires: *m*/*z* = 508.2216 (MH^+). ν_{max} 3147, 2034, 2990, 2958, 2885, 2678, 2605, 2504, 1633, 1601, 1556, 1473, 1461, 1386, 1329, 1316, 1271, 1224, 1207, 1173, 1120, 965, 876, 782, 678 cm^{-1}. ^{1}H-NMR (500 MHz, DMSO-d_6): δ 0.90

(*s*, 3H, Me); 0.96 (*s*, 3H, Me); 0.98 (br *s*, 1H, Me); 1.26 (br *s*, 1H); 1.39 – 1.87 (*m*, 8H); 2.07 – 2.76 (*m*, 6H); 3.35 (*s*, 1H); 4.50 (br *s*, 1H); 7.71 (*s*, 1H); 8.01 – 8.67 (*m*, 3H), 10.21 (br *s*, 1H). ^{13}C-NMR (126 MHz, DMSO-d_6): δ 19.22, 19.98, 23.50, 25.97, 27.57, 37.11, 39.52, 44.61, 47.74, 51.52, 55.98, 56.90, 58.53, 115.69, 121.19, 123.26 (*q*, *J* = 272.7 Hz), 130.27 (*m*), 141.87, 180.64.

1.3.49. Synthesis of 1-(3,5-bis(trifluoromethyl)phenyl)-3-((1*R*,2*R*,4*R*)-7,7-dimethyl-1-(pyrrolidin-1-ylmethyl)bicyclo[2.2.1]heptan-2-yl)thiourea (**53**).

Following *General procedure 6*. Prepared from diamine **23a** (177 mg, 0.796 mmol), Et_2O (2.5 mL), 1-isothiocyanato-3,5-bis(trifluoromethyl)benzene (142 μL, 0.760 mmol, 98%); purified by CC (Et_3N:Et_2O:petroleum ether = 1:35:25). Yield: 353 mg (0.716 mmol, 90%) of colorless solid; mp = 57–58°C. $[\alpha]_D^{20}$ = +80.5 (c = 0.65, CH_2Cl_2). CHN analysis for $C_{23}H_{29}F_6N_3S$ requires: C, 55.97; H, 5.92; N, 8.51 and found: C, 56.10; H, 6.13; N, 8.43. EI-HRMS: *m*/*z* = 494.2057 (MH$^+$); $C_{23}H_{30}F_6N_3S$ requires: *m*/*z* = 494.2059 (MH$^+$). ν_{max} 3415, 3220, 2958, 2881, 2821, 1597, 1508, 1470, 1378, 1274, 1252, 1215, 1169, 1126, 1106, 1076, 1001, 985, 936, 880, 847, 799, 721, 700, 681, 619 cm^{-1}. ^{1}H-NMR (500 MHz, DMSO-d_6): δ 0.88 (*s*, 3H, Me); 1.04 (*s*, 3H); 1.10 – 1.19 (*m*, 1H); 1.28 – 2.26 (*m*, 10H); 2.47 (br *s*, 4H); 2.56 – 3.00 (*m*, 2H); 4.27 (*s*, 1H), 7.71 (*s*, 1H); 8.25 (*s*, 1H); 8.34 (*s*, 2H); 10.29 (*s*, 1H). ^{13}C-NMR (126 MHz, DMSO-d_6): δ 20.51, 20.68, 23.42, 26.69, 33.88, 39.52, 45.04, 47.62, 51.11, 54.07, 55.46, 60.40, 115.61, 120.93, 123.27 (*q*, *J* = 272.5 Hz), 130.14 (*m*), 142.04, 178.81.

1.3.50. Synthesis of 1-(3,5-bis(trifluoromethyl)phenyl)-3-((1*R*,2*S*,4*R*)-7,7-dimethyl-1-(piperidin-1-ylmethyl)bicyclo[2.2.1]heptan-2-yl)thiourea (**54**).

Following *General procedure 6*. Prepared from diamine **22b**/**23b** (54 mg, 0.228 mmol, **22b**:**23b** = 4.8:1), Et_2O (2.5 mL), 1-isothiocyanato-3,5-bis(trifluoromethyl)benzene (41 μL, 0.220 mmol, 98%); purified by CC (1. Et_3N:Et_2O:petroleum ether = 1:6:25 for the elution of nonpolar impurities; 2. Et_3N:Et_2O = 1:5 for the elution of product **54**). Yield: 111 mg (0.219 mmol, 96%) of yellow solid; mp = 131–134°C. $[\alpha]_D^{20}$ = –51.2 (c = 0.40, CH_2Cl_2). CHN analysis for $C_{24}H_{31}F_6N_3S$ requires: C, 56.79; H, 6.16; N, 8.28 and found: C, 57.03; H, 6.23; N, 8.09. EI-HRMS: *m*/*z* = 508.2226 (MH$^+$); $C_{24}H_{32}F_6N_3S$ requires: *m*/*z* = 508.2216 (MH$^+$). ν_{max} 3130, 2939, 1618, 1539, 1506, 1468, 1374, 1352, 1272, 1242, 1172, 1129, 1096, 1050, 1036, 982, 944, 884, 784, 701, 681, 651, 617 cm^{-1}. ^{1}H-NMR (500 MHz, DMSO-d_6): δ 0.90 (*s*, 3H, Me); 0.95 (*s*, 3H, Me); 0.92 – 1.01 (*m*, 1H); 1.14 – 1.35 (*m*, 7H); 1.54 – 1.64 (*m*, 2H); 1.68 – 1.82 (*m*, 2H); 2.22 – 2.43 (*m*, 7H); 4.44 (br *s*, 1H); 7.74 (*s*, 1H); 8.15 – 8.33 (*m*, 3H, 3H of Ar); 10.24 (br *s*, 1H). ^{13}C-NMR (126 MHz, DMSO-d_6): δ 19.2, 20.0, 23.3, 25.5, 25.8, 27.5, 37.3, 44.7, 47.6, 51.4, 56.3, 57.8, 59.5, 115.9, 121.4, 123.22 (*q*, *J* = 273 Hz), 130.47 (*q*, *J* = 33 Hz), 141.9, 180.5.

1.3.51. Synthesis of 1-(3,5-bis(trifluoromethyl)phenyl)-3-((1*R*,2*S*,4*R*)-7,7-dimethyl-1-(morpholinomethyl)bicyclo[2.2.1]heptan-2-yl)thiourea (**55**).

Following *General procedure 6*. Prepared from diamine **22c**/**23c** (460 mg, 1.93 mmol, **22c**:**23c** = 4.6:1), Et_2O (5 mL), 1-isothiocyanato-3,5-bis(trifluoromethyl)benzene (335 μL, 1.80 mmol, 98%); purified by CC (Et_2O:Et_3N = 50:1). Yield: 207 mg (0.405 mmol, 21%) of yellow solid; mp = 52–55°C. $[\alpha]_D^{20}$ = –39.5 (c = 0.44, CH_2Cl_2). CHN analysis for $C_{23}H_{29}F_6N_3OS$ requires: C, 54.21; H, 5.74; N, 8.25 and found: C, 53.95; H, 5.75; N, 7.95. EI-HRMS: *m*/*z* = 510.2010 (MH$^+$); $C_{23}H_{30}F_6N_3OS$ requires: *m*/*z* = 510.2008 (MH$^+$). ν_{max} 3194, 2959, 2882, 2854, 2818, 1619, 1507, 1469, 1375, 1349, 1318, 1276, 1171, 1118, 1068, 1051, 1034, 1006, 993, 977, 964, 944, 931, 910, 885, 864, 802, 730, 701, 681, 653, 619 cm^{-1}. ^{1}H-NMR (500 MHz, DMSO-d_6): δ 0.91 (*s*, 3H, Me); 0.89 – 0.94 (*m*, 1H); 0.98 (*s*, 3H, Me); 1.19 – 1.32 (*m*, 1H); 1.55 – 1.66 (*m*, 2H); 1.70 – 1.83 (*m*, 2H); 2.27 – 2.45 (*m*, 7H); 3.39 – 3.50 (*m*, 4H); 4.62 (br *s*, 1H); 7.73 (*s*, 1H, 1H of Ar); 8.16 (br *s*, 1H); 8.31 (*s*, 2H, 2H of Ar); 10.25 (br *s*, 1H). ^{13}C-NMR (126 MHz, DMSO-d_6): δ 19.3, 20.0, 25.5, 27.6, 37.2, 44.6, 47.9, 51.6, 55.4, 57.2, 59.2, 66.4, 115.8, 121.1, 123.26 (*q*, *J* = 273 Hz), 130.28 (*q*, *J* = 32 Hz), 142.0, 180.2.

1.3.52. Synthesis of 1-(3,5-bis(trifluoromethyl)phenyl)-3-((1*R*,2*S*,4*R*)-1-((dimethylamino)methyl)-7,7-dimethylbicyclo[2.2.1]heptan-2-yl)thiourea (**56**).

Following *General procedure 6*. Prepared from diamine **22d**/**23d** (310 mg, 1.58 mmol, **22d**:**23d** = 2.8:1), Et_2O (3 mL), 1-isothiocyanato-3,5-bis(trifluoromethyl)benzene (274 μL, 1.47 mmol, 98%); purified by CC (1. Et_3N:Et_2O:petroleum ether = 1:6:25 for the elution of nonpolar impurities; 2. Et_3N:Et_2O = 1:5 for the elution of product **56**). Yield: 538 mg (1.15 mmol, 73%) of yellow solid; mp = 61–68°C. $[\alpha]_D^{20}$ = –70.8 (c = 0.37, CH_2Cl_2). CHN analysis for $C_{21}H_{27}F_6N_3S$ requires: C, 53.95; H, 5.82; N, 8.99 and found: C, 54.00; H, 5.91; N, 8.79. EI-HRMS: *m*/*z* = 468.1903 (MH^+); $C_{21}H_{28}F_6N_3S$ requires: *m*/*z* = 468.1903 (MH^+). ν_{max} 3412, 3205, 2954, 2882, 2830, 2783, 1600, 1516, 1470, 1375, 1322, 1274, 1216, 1169, 1124, 1034, 1016, 1001, 980, 942, 880, 846, 833, 726, 699, 681, 620 cm^{-1}. ^1H-NMR (500 MHz, DMSO-d_6): δ 0.89 (*s*, 3H, Me); 0.95 (*s*, 3H, Me); 1.03 (br *s*, 1H); 1.11 – 1.39 (*m*, 1H); 1.52 – 1.84 (*m*, 4H); 2.00 – 2.43 (*m*, 9H); 4.51 (*s*, 1H); 7.71 (*s*, 1H); 7.89 – 8.89 (*m*, 3H); 10.29 (*s*, 1H). ^{13}C-NMR (126 MHz, DMSO-d_6): δ 19.18, 20.02, 25.19, 27.46, 33.41, 37.16, 44.49, 47.83, 51.66, 57.84, 60.25, 115.67, 121.26, 123.22 (*q*, *J* = 272.8 Hz), 130.22 (*m*), 141.85, 180.39.

1.3.53. Synthesis of 1-(*tert*-butyl)-3-((1*R*,2*S*,4*R*)-7,7-dimethyl-1-(pyrrolidin-1-ylmethyl)bicyclo[2.2.1]heptan-2-yl)thiourea (**57**).

Following *General procedure 6*. Prepared from diamine **22a** (50 mg, 0.225 mmol), Et_2O (1 mL), 2-isothiocyanato-2-methylpropane (28 μL, 0.214 mmol); purified by CC (Et_2O:MeOH:Et_3N = 100:1:1). Yield: 56 mg (0.1665 mmol, 74%) of colorless solid; mp = 40–42°C. $[\alpha]_D^{20}$ = –167.0 (c = 0.12, CH_2Cl_2). EI-HRMS: *m*/*z* = 338.2626 (MH^+); $C_{19}H_{36}N_3S$ requires: *m*/*z* = 338.2624 (MH^+). ν_{max} 3274, 2956, 2876, 2803, 1517, 1477, 1459, 1390, 1336, 1309, 1274, 1251, 1225, 1197, 1110, 1069, 1034, 1000, 966, 940, 921, 908, 873, 780, 763, 701, 657, 609 cm^{-1}. ^1H-NMR (500 MHz, DMSO-d_6): δ 0.70 – 0.78 (*m*, 1H); 0.89 (*s*, 3H, Me); 0.93 (*s*, 3H, Me); 1.10 – 1.21 (*m*, 1H); 1.40 (*s*, 9H, Boc); 1.47 – 1.58 (*m*, 2H); 1.58 – 1.67 (*m*, 4H); 1.66 – 1.75 (*m*, 2H); 2.22 – 2.32 (*m*, 1H); 2.33 – 2.54 (*m*, 6H); 4.48 (br *s*, 1H); 7.17 (br *s*, 1H); 7.25 (*s*, 1H). ^{13}C-NMR (126 MHz, DMSO-d_6): δ 19.3, 20.1, 23.7, 25.6, 27.7, 29.0, 38.1, 44.7, 48.0, 51.7, 51.9, 55.9, 56.3, 56.4, 181.7.

1.3.54. Synthesis of 1-(adamantan-1-yl)-3-((1*R*,2*S*,4*R*)-7,7-dimethyl-1-(pyrrolidin-1-ylmethyl)bicyclo[2.2.1]heptan-2-yl)thiourea (**58**).

Following *General procedure 6*. Prepared from diamine **22a** (47 mg, 0.211 mmol), Et_2O (2 mL), 1-isothiocyanatoadamantane (39 mg, 0.200 mmol); purified by CC (Et_2O:MeOH:Et_3N = 100:1:1). Yield: 57 mg (0.137 mmol, 65%) of colorless solid; mp = 144–145°C. $[\alpha]_D^{20}$ = –58.7 (c = 0.2, CH_2Cl_2). EI-HRMS: *m*/*z* = 416.3095 (MH^+); $C_{25}H_{42}N_3S$ requires: *m*/*z* = 416.3094 (MH^+). ν_{max} 3234, 2904, 2850, 2788, 1505, 1453, 1358, 1339, 1305, 1278, 1224, 1189, 1115, 1092, 1068, 1051, 1038, 939, 875, 841, 775, 624 cm^{-1}. ^1H-NMR (500 MHz, DMSO-d_6): δ 0.68 – 0.78 (*m*, 1H); 0.88 (*s*, 3H, Me); 0.93 (*s*, 3H, Me); 1.11 – 1.21 (*m*, 1H); 1.53 (*q*, *J* = 5.6, 4.3 Hz, 2H); 1.58 – 1.66 (*m*, 11H); 1.66 – 1.74 (*m*, 2H); 1.98 – 2.05 (*m*, 3H); 2.09 – 2.17 (*m*, 6H); 2.21 – 2.31 (*m*, 1H); 2.35 – 2.49 (*m*, 5H); 4.47 (br *s*, 1H); 7.09 – 7.21 (*m*, 2H). ^{13}C-NMR (126 MHz, DMSO-d_6): δ 19.3, 20.2, 23.8, 25.7, 27.8, 29.0, 36.1, 38.1, 41.3, 44.7, 45.7, 48.0, 51.8, 52.5, 56.0, 56.5, 64.9, 181.0.

1.3.55. Synthesis of 1-(3,5-bis(trifluoromethyl)phenyl)-3-(((1*R*,2*S*,4*R*)-7,7-dimethyl-2-(pyrrolidin-1-yl)bicyclo[2.2.1]heptan-1-yl)methyl)thiourea (**59**).

Following *General procedure 6*. Prepared from diamine **34** (248 mg, 1.115 mmol), Et_2O (2.5 mL), 1-isothiocyanato-3,5-bis(trifluoromethyl)benzene (186 μL, 1.00 mmol, 98%); purified by CC (Et_3N:EtOAc:petroleum ether = 1:1:20). Yield: 396 mg (0.803 mmol, 72%) of yellow solid; mp = 53–57°C. $[\alpha]_D^{20}$ = –21.1 (c = 0.25, CH_2Cl_2). EI-HRMS: *m*/*z* = 494.2063 (MH^+); $C_{23}H_{30}F_6N_3S$ requires: *m*/*z* = 494.2059 (MH^+). ν_{max} 3141, 2953, 2880, 2819, 1667, 1619, 1521, 1466, 1374, 1274, 1171, 1128, 1106, 947, 884, 849, 701, 680, 650 cm^{-1}. ^1H-NMR (600 MHz, DMSO-d_6): δ 0.95 (*s*, 3H, Me); 0.98 (*s*, 3H, Me); 1.14 – 1.23 (*m*, 2H); 1.33 – 1.41 (*m*, 1H); 1.48 – 1.54 (*m*, 1H); 1.56 – 1.67 (*m*, 4H); 1.68 – 1.76 (*m*, 1H); 1.92 – 2.05 (*m*, 2H); 2.42 – 2.61 (*m*, 4H); 2.73 – 2.78 (*m*, 1H); 3.51 (*dd*, *J* = 3.5; 14.4 Hz, 1H); 3.93 (*dd*, *J* = 5.1; 15.0 Hz, 1H); 7.73 (*s*, 1H, 1H of Ar); 7.87 (br *s*, 1H, NH); 8.32 (*s*, 2H, 2H of Ar); 10.29 (br *s*, 1H, NH). ^{13}C-NMR (151 MHz, DMSO-d_6): δ 19.6, 20.5, 22.9, 24.9, 27.9, 34.9, 45.0, 45.3, 48.8, 51.6, 53.0, 65.4, 115.9, 121.3, 123.23 (*q*, *J* = 273 Hz), 130.25 (*q*, *J* = 33 Hz), 141.9, 180.0.

1.3.56. Synthesis of 1-(3,5-bis(trifluoromethyl)phenyl)-3-(((1*R*,2*R*,4*R*)-7,7-dimethyl-2-(pyrrolidin-1-yl)bicyclo[2.2.1]heptan-1-yl)methyl)thiourea (**60**).

Following *General procedure 6*. Prepared from diamine **33** (143 mg, 0.643 mmol), Et_2O (2.5 mL), 1-isothiocyanato-3,5-bis(trifluoromethyl)benzene (108 µL, 0.580 mmol, 98%); purified by CC (Et_3N:Et_2O:petroleum ether = 1:20:20). Yield: 175 mg (0.345 mmol, 55%) of yellow solid; mp = 53–55°C. $[\alpha]_D^{20}$ = –47.8 (c = 0.24, CH_2Cl_2). CHN analysis for $C_{23}H_{29}F_6N_3S$ requires: C, 55.97; H, 5.92; N, 8.51 and found: C, 55.77; H, 6.14; N, 8.14. EI-HRMS: *m*/*z* = 494.2061 (MH^+); $C_{23}H_{30}F_6N_3S$ requires: *m*/*z* = 494.2059 (MH^+). ν_{max} 3144, 2953, 2877, 2815, 1667, 1508, 1468, 1376, 1274, 1171, 1128, 947, 884, 848, 720, 701, 681, 649 cm^{-1}. ^{1}H-NMR (600 MHz, DMSO-d_6): δ 0.89 (*s*, 3H, Me); 1.05 – 1.15 (*m*, 1H); 1.14 (*s*, 3H, Me); 1.17 – 1.27 (*m*, 1H); 1.47 – 1.59 (*m*, 6H); 1.58 – 1.64 (*m*, 1H); 1.66 – 1.75 (*m*, 1H); 1.92 – 2.01 (*m*, 1H); 2.37 – 2.54 (*m*, 5H); 3.45 (*dd*, *J* = 3.1; 14.2 Hz, 1H); 3.78 (*dd*, *J* = 5.0; 14.2 Hz, 1H); 7.74 (*s*, 1H, 1H of Ar); 8.06 (br *s*, 1H); 8.33 (*s*, 2H, 2H of Ar); 10.32 (br *s*, 1H). ^{13}C-NMR (151 MHz, DMSO-d_6): δ 20.7, 20.9, 22.8, 26.5, 33.0, 35.6, 45.2, 45.5, 47.1, 50.9, 52.9, 72.2, 116.0, 121.5, 123.22 (*q*, *J* = 273 Hz), 130.26 (*q*, *J* = 33 Hz), 141.8, 180.0.

1.3.57. Synthesis of 1-(3,5-bis(trifluoromethyl)phenyl)-3-((1*S*,2*S*,4*R*)-7,7-dimethyl-1-(2-(pyrrolidin-1-yl)ethyl)bicyclo[2.2.1]heptan-2-yl)thiourea (**61**).

Following *General procedure 6*. Prepared from diamine **38** (25 mg, 0.106 mmol), Et_2O (2.5 mL), 1-isothiocyanato-3,5-bis(trifluoromethyl)benzene (18.6 µL, 0.10 mmol, 98%); purified by CC (1. Et_3N:Et_2O:petroleum ether = 1:6:25 for the elution of nonpolar impurities; 2. Et_3N:Et_2O = 1:5 for the elution of product **61**). Yield: 49 mg (0.0954 mmol, 90%) of yellow solid; mp = 66–70°C. $[\alpha]_D^{20}$ = –16.1 (c = 0.18, CH_2Cl_2). CHN analysis for $C_{24}H_{31}F_6N_3S$ requires: C, 56.79; H, 6.16; N, 8.28 and found: C, 56.48; H, 6.21; N, 8.00. EI-HRMS: *m*/*z* = 508.2224 (MH^+); $C_{24}H_{32}F_6N_3S$ requires: *m*/*z* = 508.2216 (MH^+). ν_{max} 3178, 3142, 2959, 2880, 2808, 1618, 1533, 1468, 1375, 1346, 1274, 1171, 1129, 882, 701, 681, 655, 620 cm^{-1}. ^{1}H-NMR (500 MHz, DMSO-d_6): δ 0.88 (*s*, 3H, Me); 0.90 – 0.98 (*m*, 1H); 0.94 (*s*, 3H, Me); 1.20 – 1.30 (*m*, 1H); 1.40 – 1.59 (*m*, 7H); 1.64 (*s*, 1H); 1.69 – 1.80 (*m*, 2H); 2.28 – 2.46 (*m*, 7H); 4.53 (br *s*, 1H); 7.71 (*s*, 1H); 8.22 (*s*, 2H); 8.74 (br *s*, 1H); 10.08 (br *s*, 1H). ^{13}C-NMR (126 MHz, DMSO-d_6): δ 18.9, 19.9, 22.8, 25.7, 27.7, 28.9, 44.4, 45.7, 48.8, 51.0, 52.8, 53.4, 58.3, 115.5, 120.8, 123.22 (*q*, *J* = 273 Hz), 130.29 (*q*, *J* = 32 Hz), 142.2, 180.6.

1.3.58. Synthesis of 1-(3,5-bis(trifluoromethyl)phenyl)-3-(2-((1*S*,2*R*,4*R*)-7,7-dimethyl-2-(pyrrolidin-1-yl)bicyclo[2.2.1]heptan-1-yl)ethyl)thiourea (**62**).

Following *General procedure 6*. Prepared from diamine **46** (100 mg, 0.423 mmol), Et_2O (2.5 mL), 1-isothiocyanato-3,5-bis(trifluoromethyl)benzene (74.5 µL, 0.400 mmol, 98%); purified by CC (EtOAc:MeOH = 10:1). Yield: 103 mg (0.203 mmol, 48%) of colorless solid; mp = 60–62°C. $[\alpha]_D^{20}$ = –40.3 (c = 0.12, CH_2Cl_2). EI-HRMS: *m*/*z* = 508.2215 (MH^+); $C_{24}H_{32}F_6N_3S$ requires: *m*/*z* = 508.2216 (MH^+). ν_{max} 3238, 2951, 2878, 2815, 1676, 1622, 1546, 1471, 1380, 1348, 1276, 1172, 1130 999, 946, 884, 847, 724, 701, 680 cm^{-1}. ^{1}H-NMR (500 MHz, DMSO-d_6): δ 0.83 (*s*, 3H, Me); 1.02 (*s*, 3H, Me); 0.97 – 1.15 (*m*, 2H); 1.39 – 1.52 (*m*, 1H); 1.52 – 1.72 (*m*, 8H); 1.72 – 1.86 (*m*, 1H); 1.87 – 1.97 (*m*, 1H); 2.21 – 2.35 (*m*, 1H); 2.42 – 2.63 (*m*, 4H); 3.44 – 3.70 (*m*, 2H); 7.72 (*s*, 1H); 8.25 (*s*, 2H); 8.40 (*s*, 1H, NH); 10.03 (br *s*, 1H, NH). ^{13}C-NMR (126 MHz, DMSO-d_6): δ 20.0, 21.3, 22.9, 26.7, 26.9, 32.6, 35.9, 41.5, 44.3, 47.7, 51.2, 53.4, 72.4, 115.8, 121.6, 123.28 (*q*, *J* = 273 Hz), 130.11 (*q*, *J* = 34 Hz), 142.1, 180.2.

1.3.59. Synthesis of 1-(3,5-bis(trifluoromethyl)phenyl)-3-(2-((1*S*,2*S*,4*R*)-7,7-dimethyl-2-(pyrrolidin-1-yl)bicyclo[2.2.1]heptan-1-yl)ethyl)thiourea (**63**).

Following *General procedure 6*. Prepared from diamine **47** (189 mg, 0.80 mmol), Et_2O (2.5 mL), 1-isothiocyanato-3,5-bis(trifluoromethyl)benzene (152 µL, 0.80 mmol, 98%); purified by CC (Et_3N:EtOAc:MeOH = 0.2:20:1). Yield: 95 mg (0.187 mmol, 23%) of colorless solid; mp = 47–48°C. $[\alpha]_D^{20}$ = +49.3 (c = 0.28, CH_2Cl_2). EI-HRMS: *m*/*z* = 508.2210 (MH^+); $C_{24}H_{32}F_6N_3S$ requires: *m*/*z* = 508.2216 (MH^+). ν_{max} 3241, 2946, 2879, 2808, 1620, 1541, 1376, 1344, 1273, 1169, 1125, 999, 947, 882, 847, 725, 700, 680 cm^{-1}. ^{1}H-NMR (500 MHz, DMSO-d_6): δ 0.94 (*s*, 3H, Me); 1.00 (*s*, 3H, Me); 1.08 – 1.14 (*m*, 1H); 1.14 – 1.20 (*m*, 1H); 1.29 – 1.38 (*m*, 1H); 1.45 – 1.50 (*m*, 1H); 1.59 – 1.76 (*m*, 7H); 1.86 – 1.94 (*m*, 1H); 1.94 – 2.02 (*m*, 1H);

2.52 – 2.71 (*m*, 6H); 3.60 (br *s*, 1H); 7.70 (*s*, 1H); 8.21 (*s*, 2H); 8.40 (br *s*, 1H, NH); 9.99 (br *s*, 1H, NH). ^{13}C-NMR (126 MHz, DMSO-d_6): δ 20.1, 20.6, 22.9, 26.2, 28.1, 29.8, 35.5, 41.2, 45.1, 49.6, 50.5, 53.2, 66.8, 115.8, 121.8, 123.28 (*q*, *J* = 273 Hz), 130.11 (*q*, *J* = 33 Hz), 142.1, 180.2.

2. Structure Determination by NMR.

The configuration of the newly formed stereocenter at C-2 for the *endo*-isomers **10**, **12**, **14**, **16**, **32**, **38**, **45**, **49**, **51**, **54-59**, **61**, and **63** was determined by 2D-NOESY spectroscopy. NOE between the 2-H and the 8-Me group were in agreement with the (2*S*)-configuration. The NOE between the NH and 8-Me of catalyst **48** is in agreement with the (2*R*)-configuration at the C-2 stereocenter (Figure S1). The (2*S*)-configuration of the *endo*-isomers **10**, **12**, **22a-d**, **30**, **38**, and **43** was additionally confirmed on the basis of chemical shift correlations of the highly up-field shifted *endo*-proton at the position 3, which appears as a doublet of doublet in a range of 0.61 ppm to 0.84 ppm. On the other hand, for the *exo*-epimers **11**, **13**, **23a-d**, **29**, **39**, and **42**, no such signal at those chemical shifts appears (Figure S2).

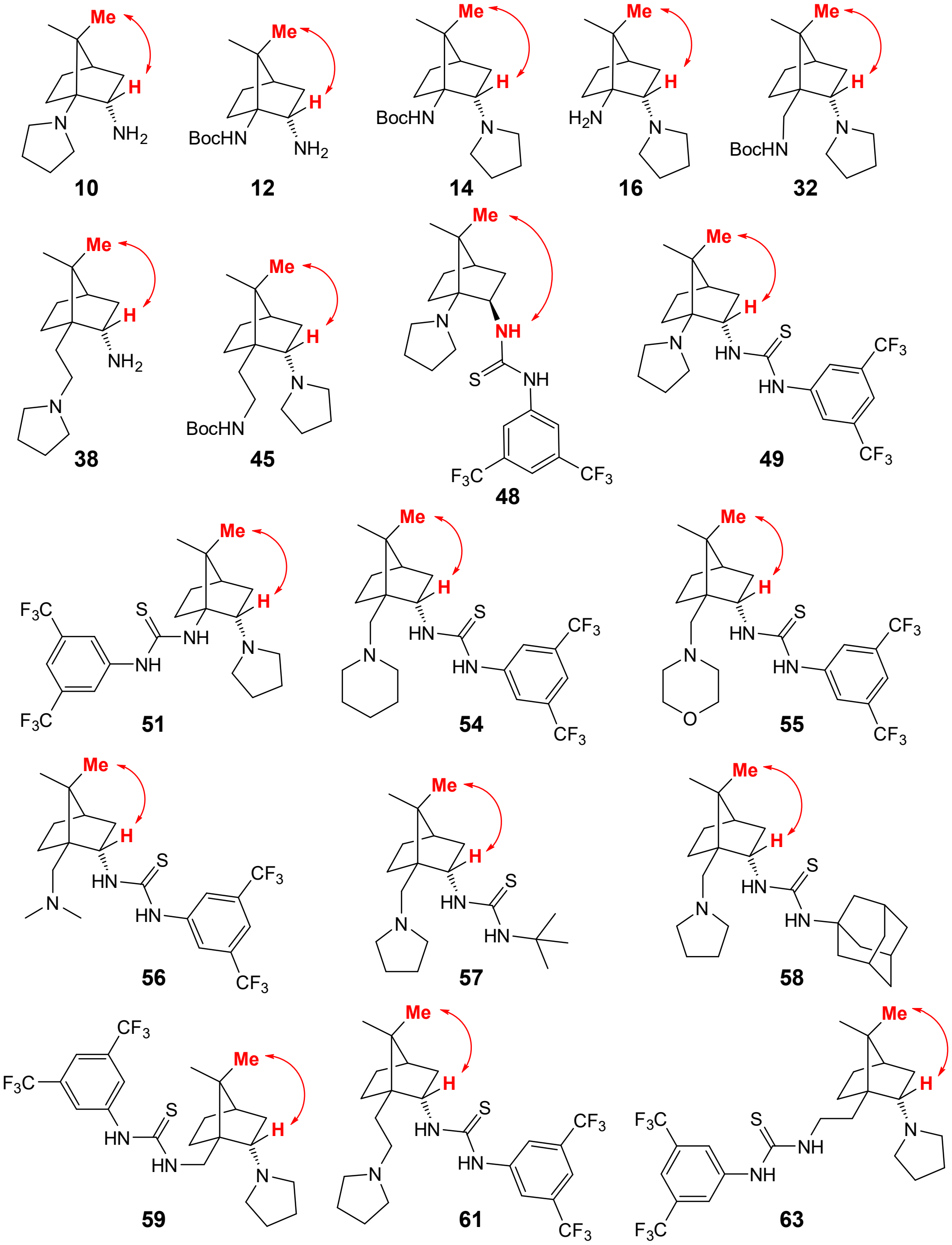

Figure S1. Determination of the absolute configuration at the C-2 based on the observed NOE correlation spectroscopy cross peaks.

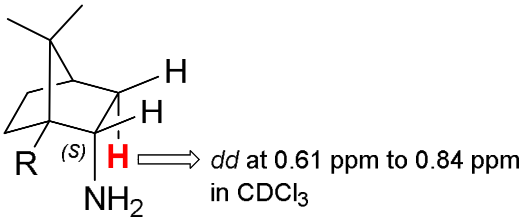

amine	*endo*-2-H
**10**	0.78 ppm
**12**	0.84 ppm
**22a**	0.61 ppm; Ref. 14
**22b**	0.68 ppm
**22c**	0.67 ppm
**22d**	0.67 ppm
**30**	0.61 ppm
**38**	0.72 ppm
**43**	0.74 ppm

Figure S2. Determination of the absolute configuration at C-2 based on chemical shift correlations in the series of primary *endo*-amines.

3. Structure Determination by X-Ray Diffaction Analysis.

Single-crystal X-ray structure analysis of compounds 8, 18, 21a, 22a, 24, 28, 29, 37, and 52.

Single-crystal diffraction data for all eight compounds have been collected on an Agilent SuperNova dual source diffractometer with an Atlas detector at room temperature using a mirror monochromator. CuKα radiation with λ = 1.54184 Å was used in data collection of **8**, **18**, **21a**, **22a**, **29**, and **52** and MoKα radiation with λ = 0,71073 Å in data collection of **24**, **28**, and **37**. The diffraction data were processed using CRYSALIS PRO software [17]. All structures were solved by direct methods, using SIR97 [18]. Full-matrix least-squares refinements on F^2 were done with anisotropic displacement parameters for all non-hydrogen atoms. H atoms bonded to O or N atoms were located from difference Fourier maps and the remaining were placed at calculated positions and treated as riding model. SHELXL-97 software [19] was used for structure refinement and interpretation. Drawings of the structures (Figures S3-S11) were produced using ORTEP-III [20]. Structural and other crystallographic details on data collection and refinement have been deposited with the Cambridge Crystallographic Data Centre as supplementary publication numbers CCDC Deposition Number 2006164-2006172 for **18**, **21a**, **24**, **29**, **52**, **8**, **28**, **37** and **22a**, respectively. These data can be obtained free of charge via www.ccdc.cam.ac.uk/conts/retrieving.html (or from the CCDC, 12 Union Road, Cambridge CB2 1EZ, UK; fax: +44 1223 336033; e-mail: deposit@ccdc.cam.ac.uk).

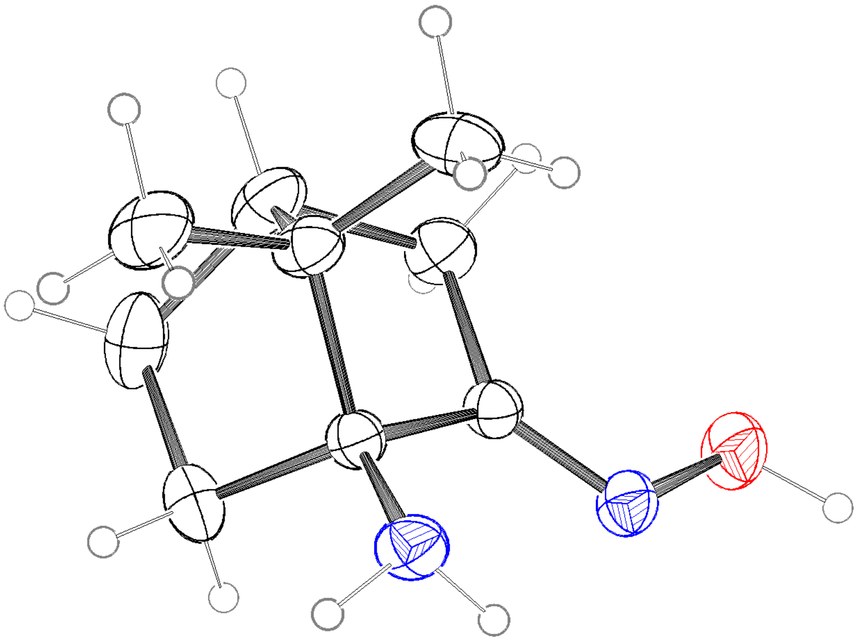

Figure S3. Ortep drawing of amino-oxime **8**.

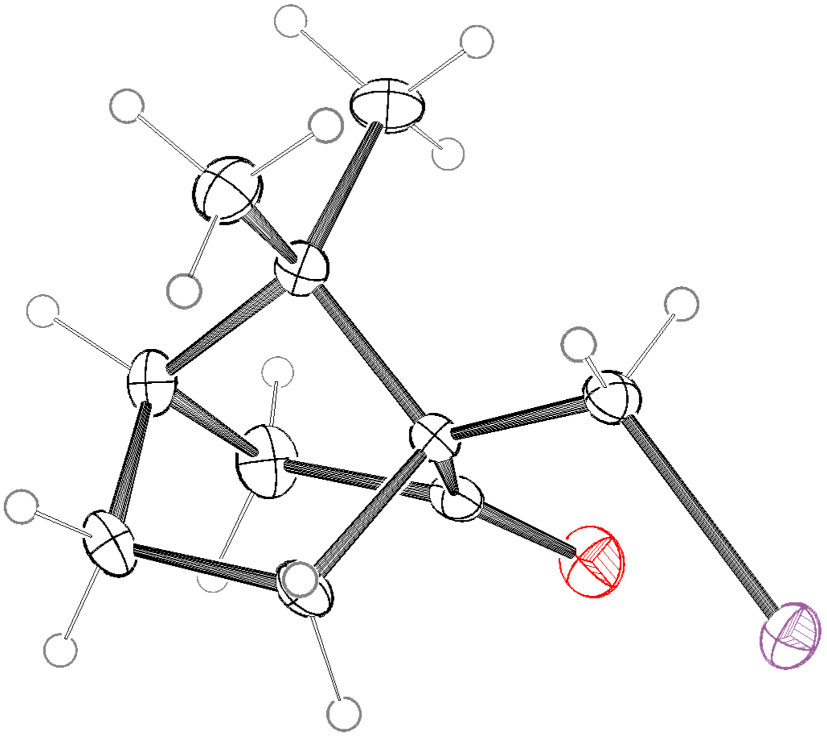

Figure S4. Ortep drawing of 10-iodocamphor (**18**).

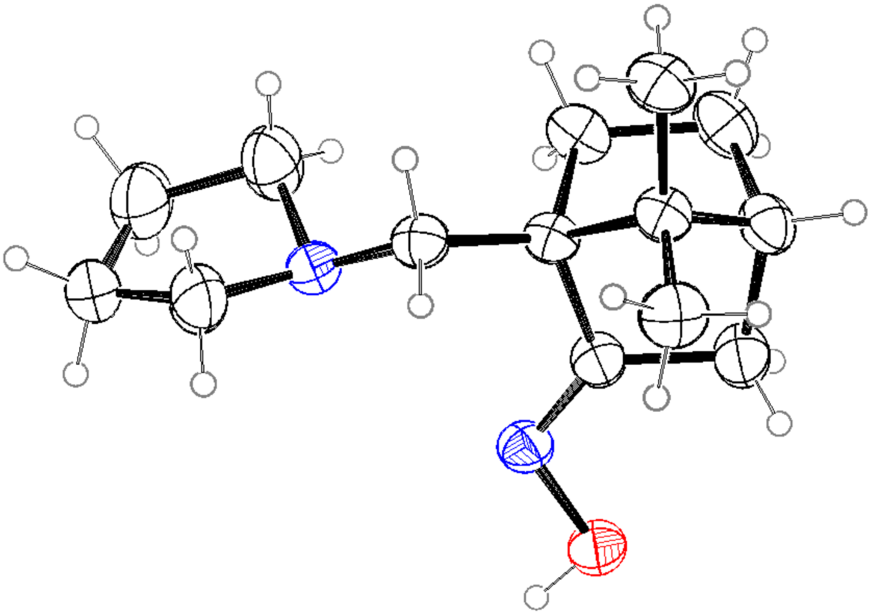

Figure S5. Ortep drawing of oxime **21a**.

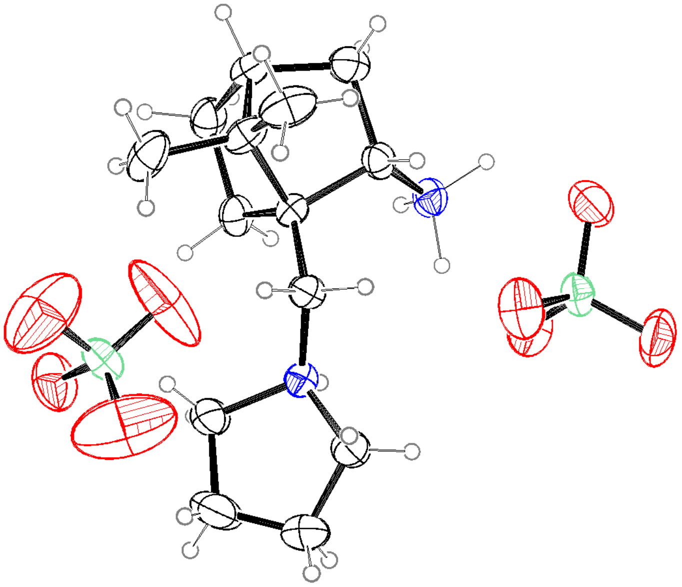

Figure S6. Ortep drawing of diamine **22a**•$HClO_4$.

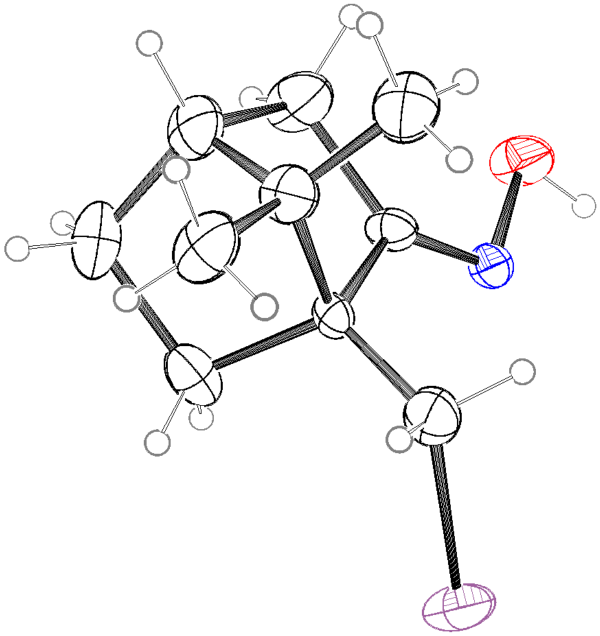

Figure S7. Ortep drawing of iodo-oxime **24**.

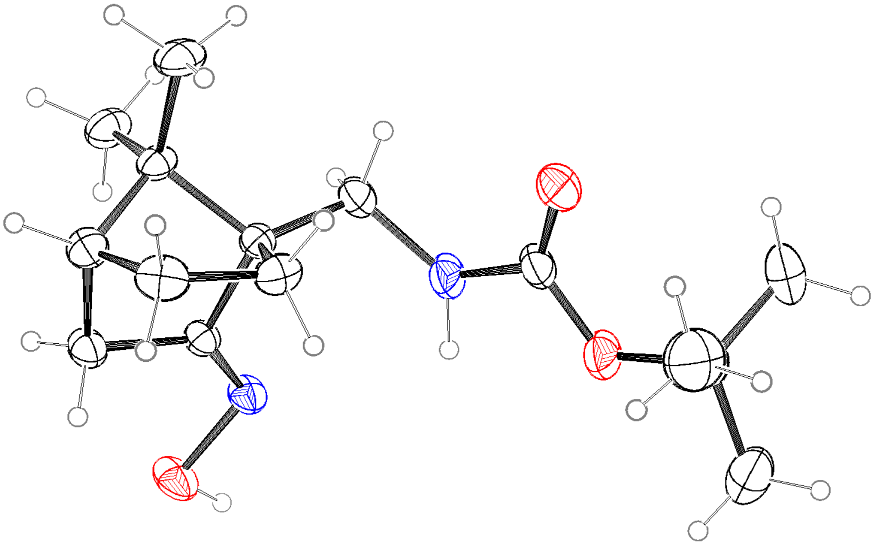

Figure S8. Ortep drawing of oxime **28**.

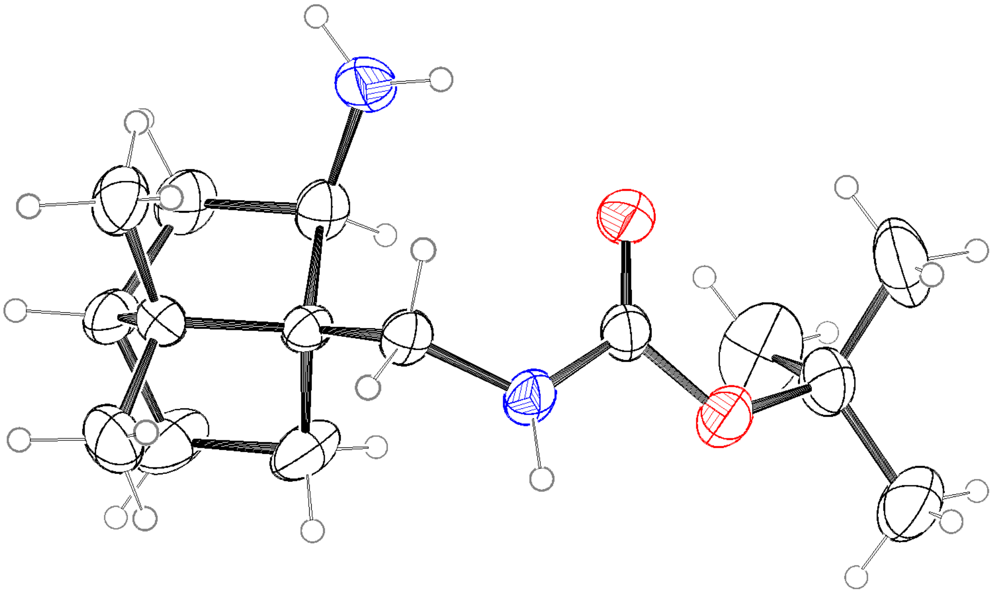

Figure S9. Ortep drawing of amine **29**.

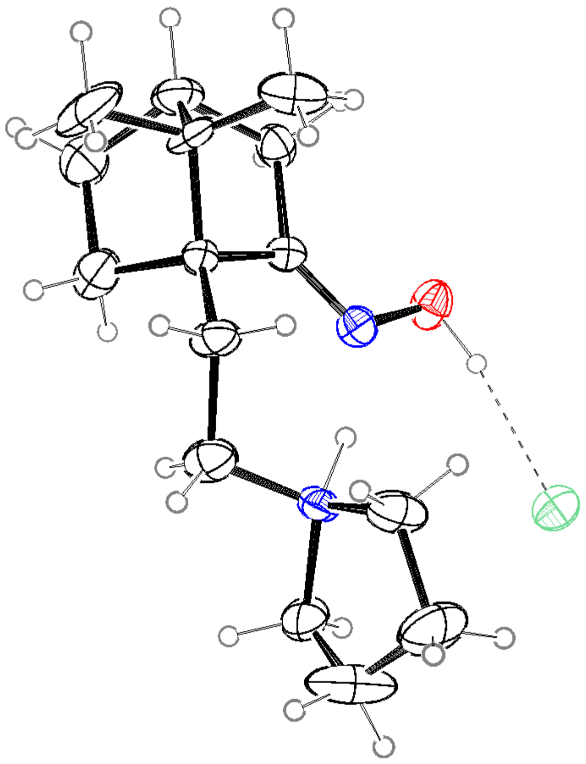

Figure S10. Ortep drawing of amino-oxime **37**.

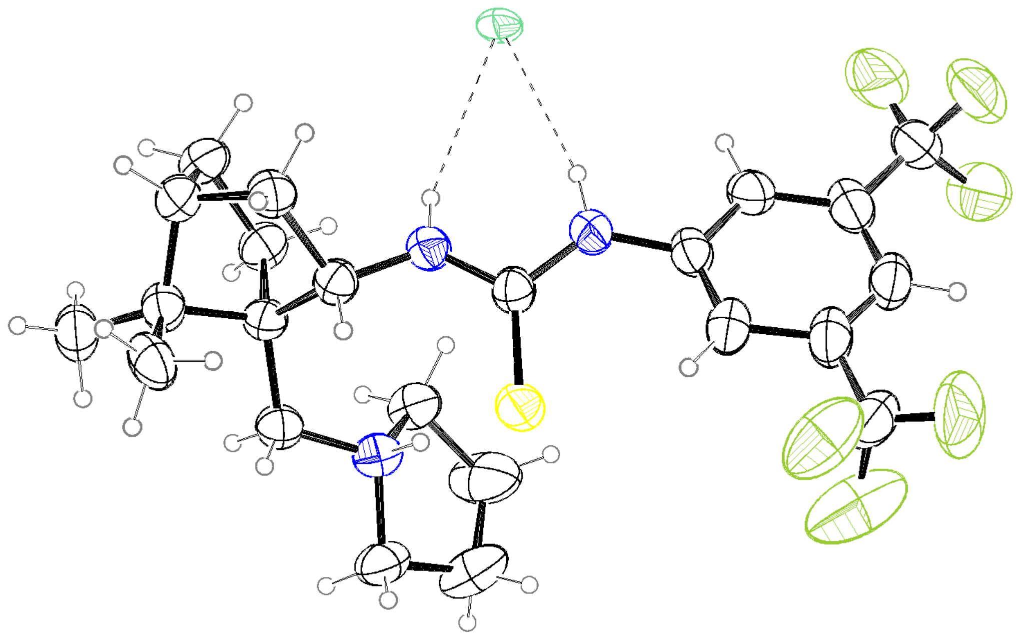

Figure S11. Ortep drawing of organocatalyst **52•HCl**.

4. Copies of NMR spectra of Compounds 5, 7, 8, 10–16, 20b–d, 21b–d, 22b–d, 23b–d, 24–43, 45, 48–63 (1H-, 13C-NMR, 2D NOESY)

(1*S*,4*R*,*E*)-7,7-Dimethyl-1-(pyrrolidin-1-yl)bicyclo[2.2.1]heptan-2-one oxime (**5**)

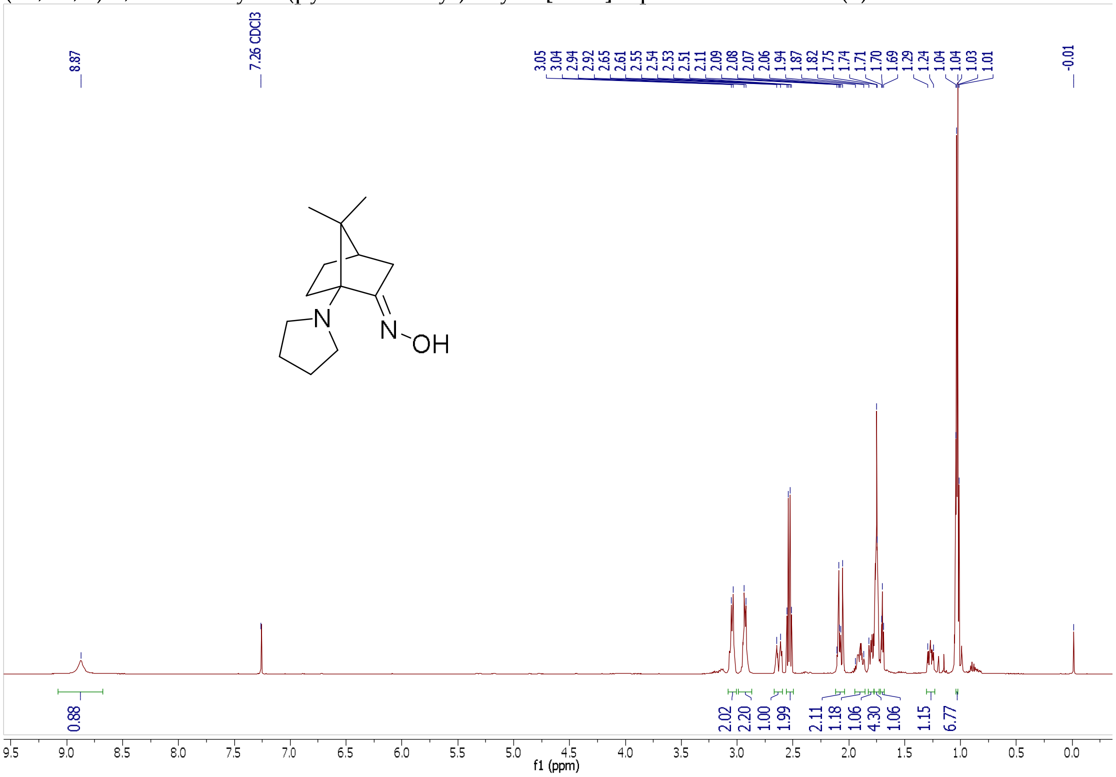

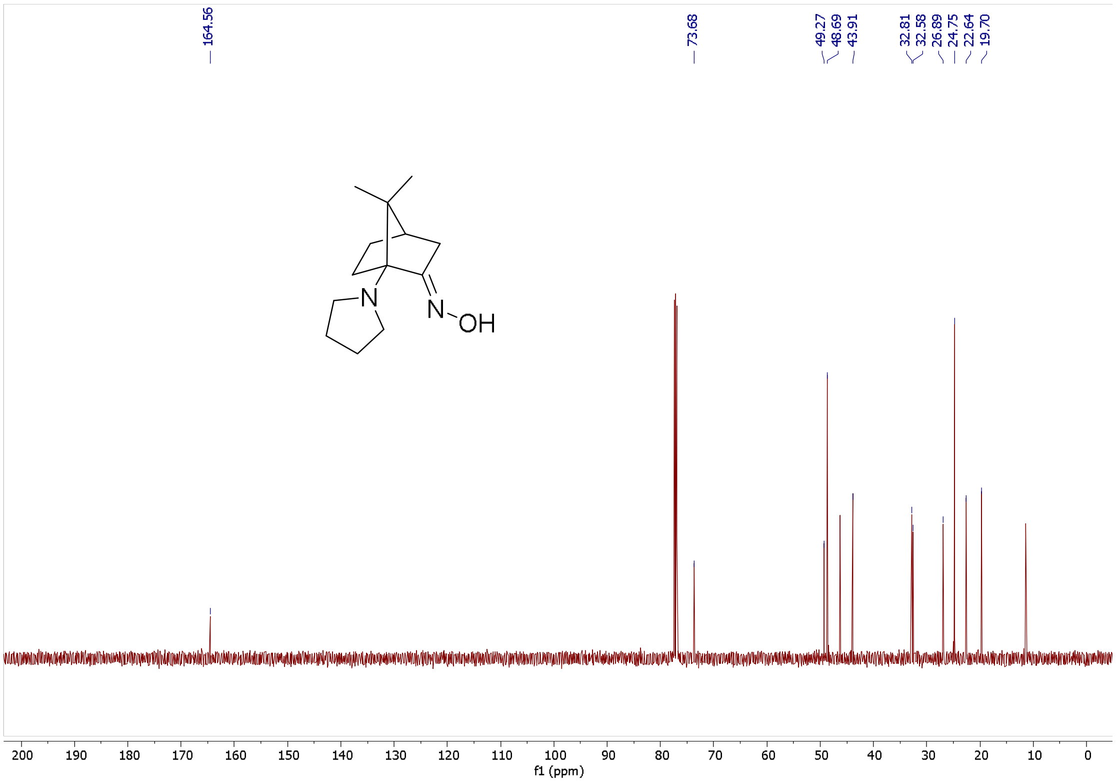

tert-Butyl ((1*S*,4*R*,*E*)-2-(hydroxyimino)-7,7-dimethylbicyclo[2.2.1]heptan-1-yl)carbamate (**7**)

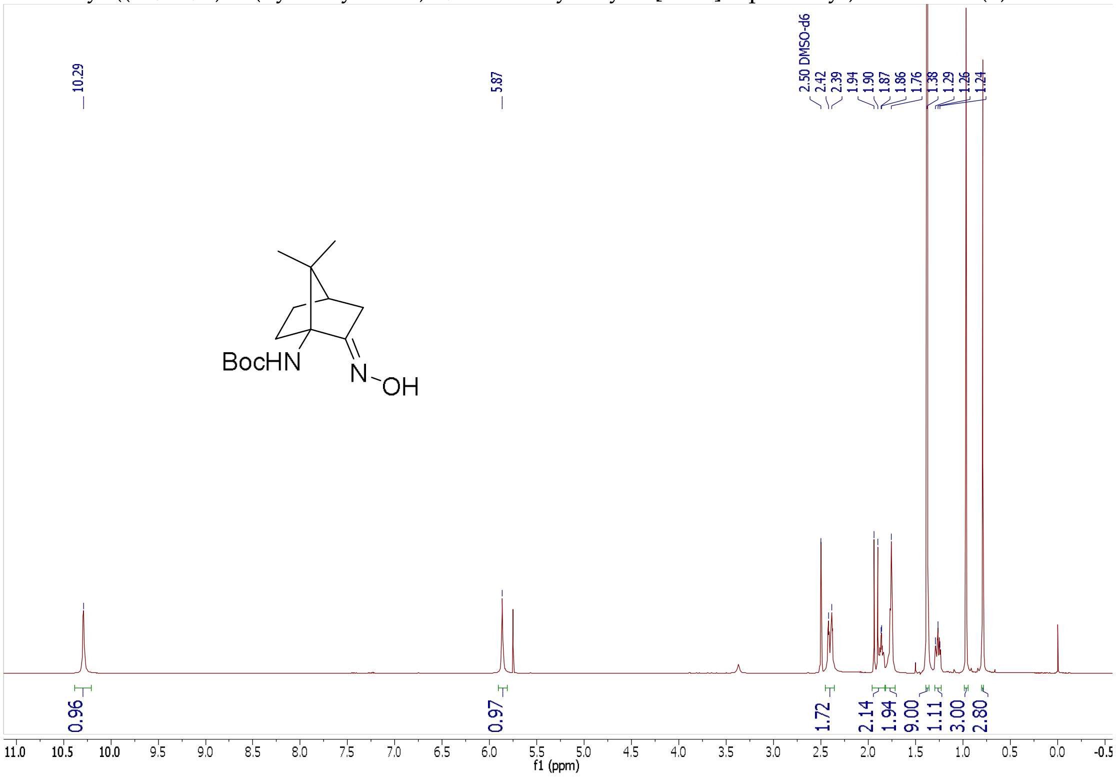

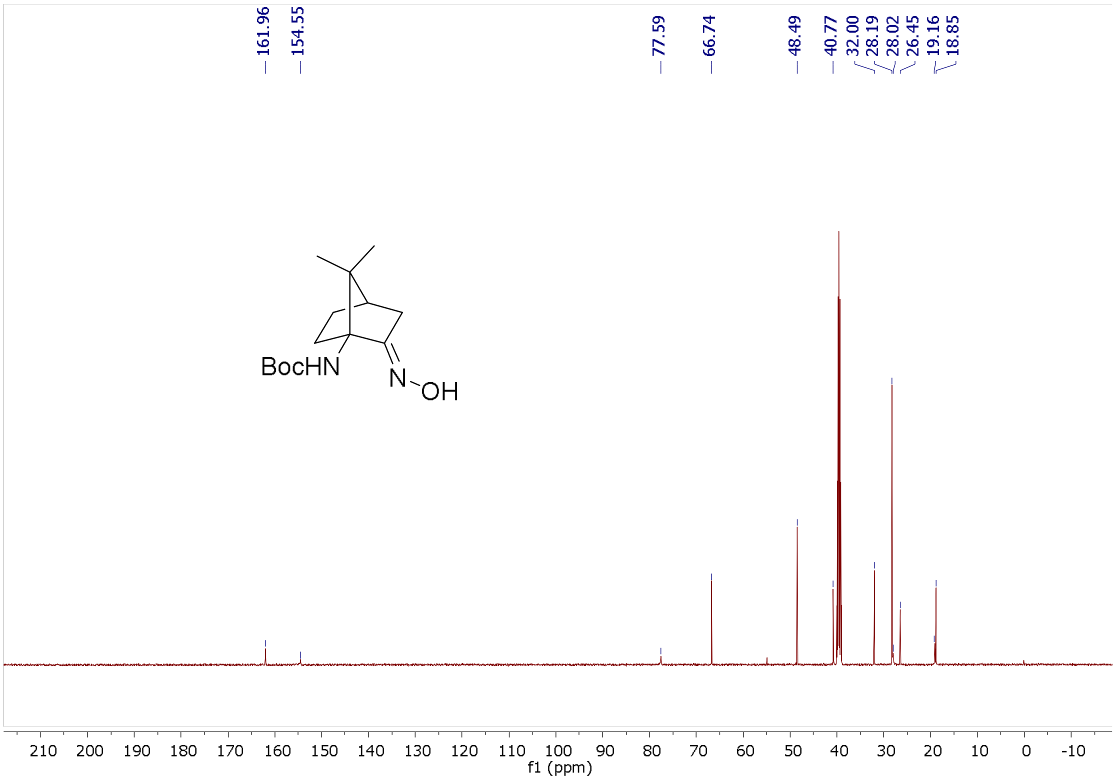

(1*S*,4*R*,*E*)-1-Amino-7,7-dimethylbicyclo[2.2.1]heptan-2-one oxime (**8**)

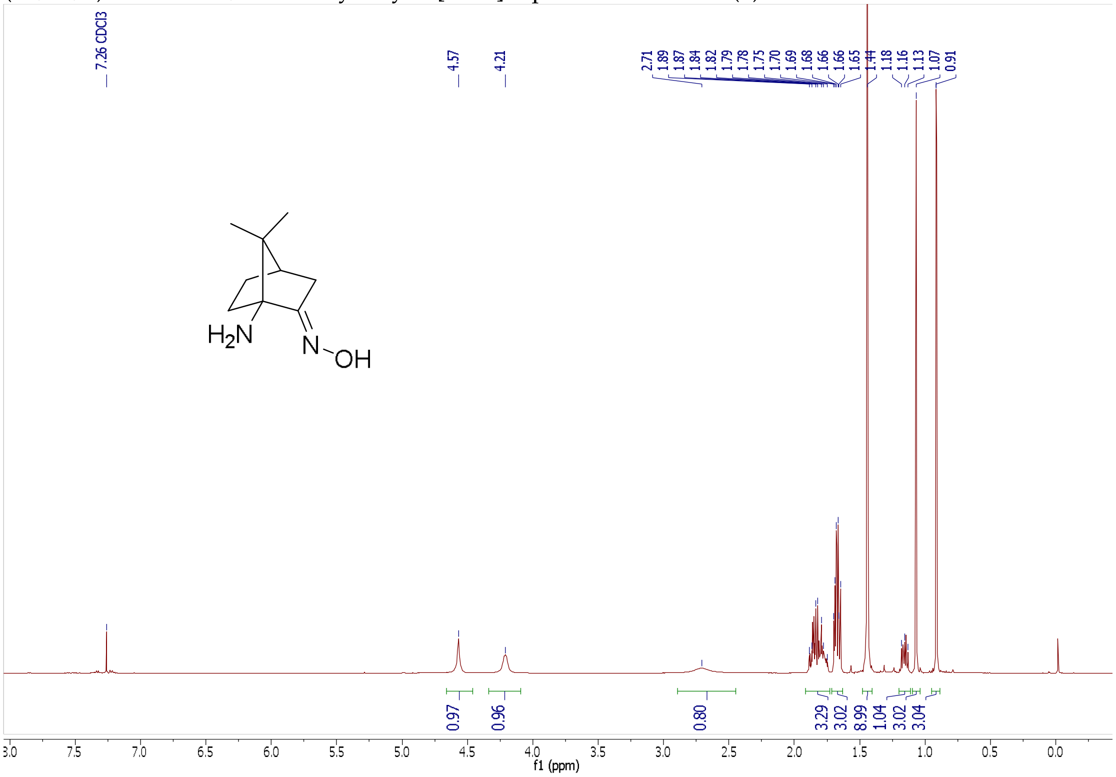

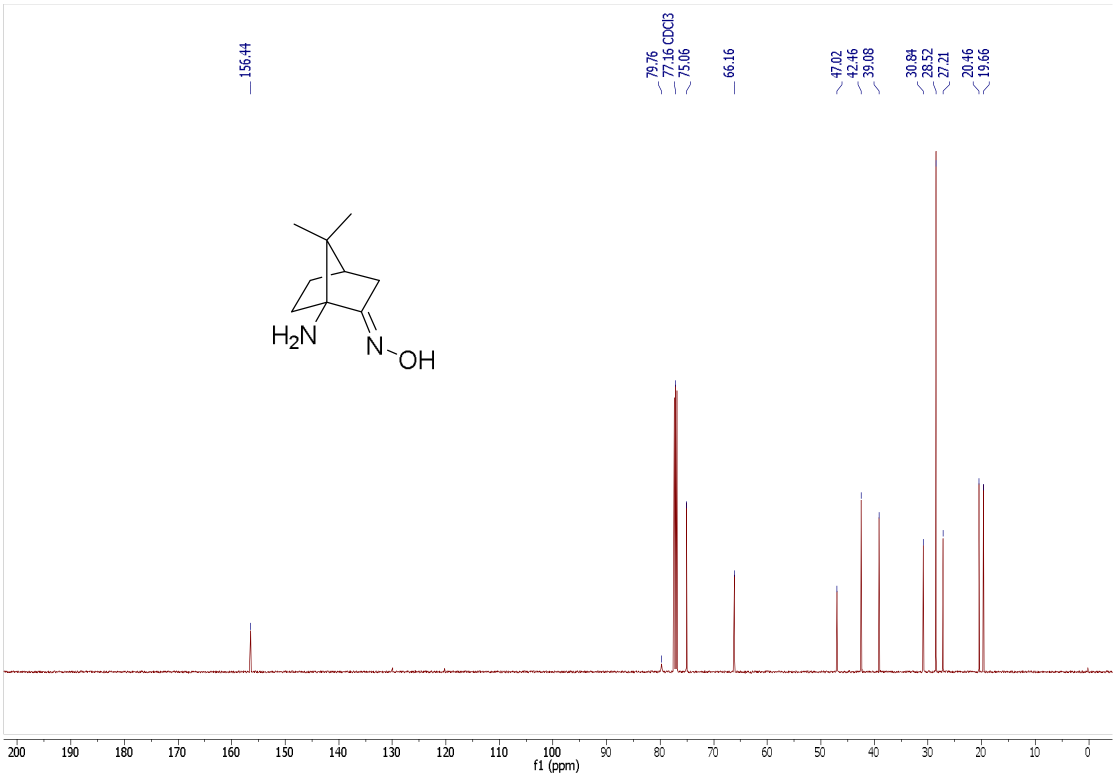

(1*S*,2*S*,4*R*)-7,7-Dimethyl-1-(pyrrolidin-1-yl)bicyclo[2.2.1]heptan-2-amine (**10**)

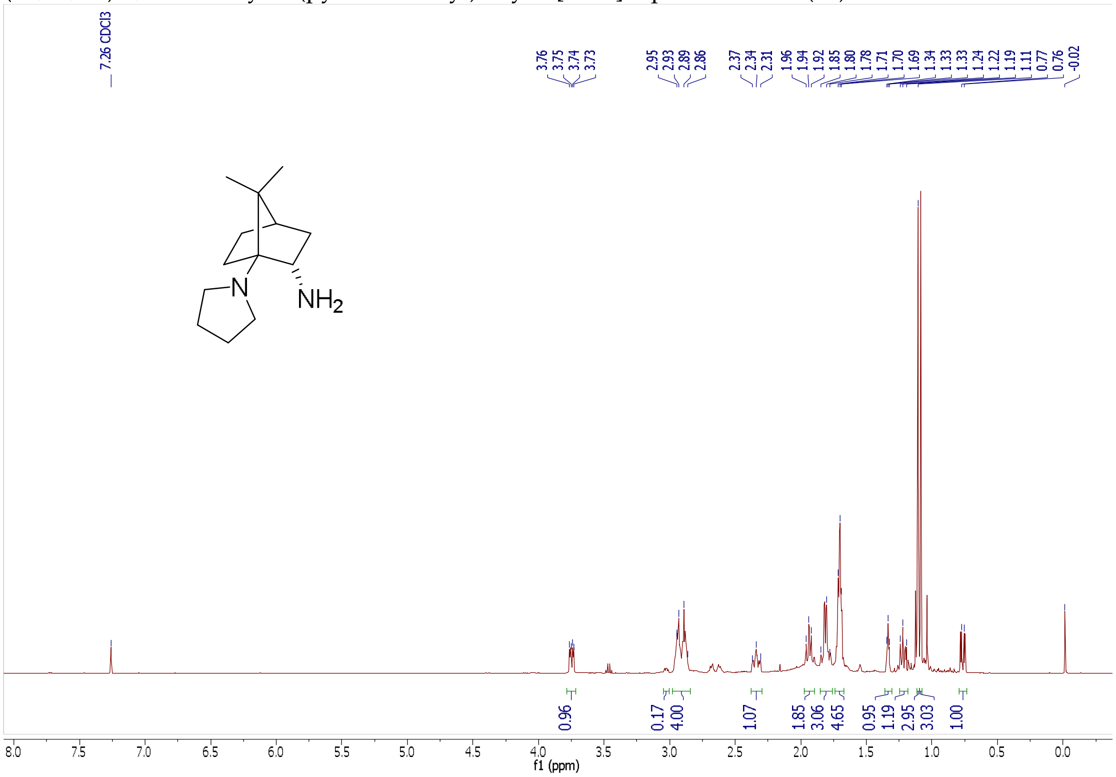

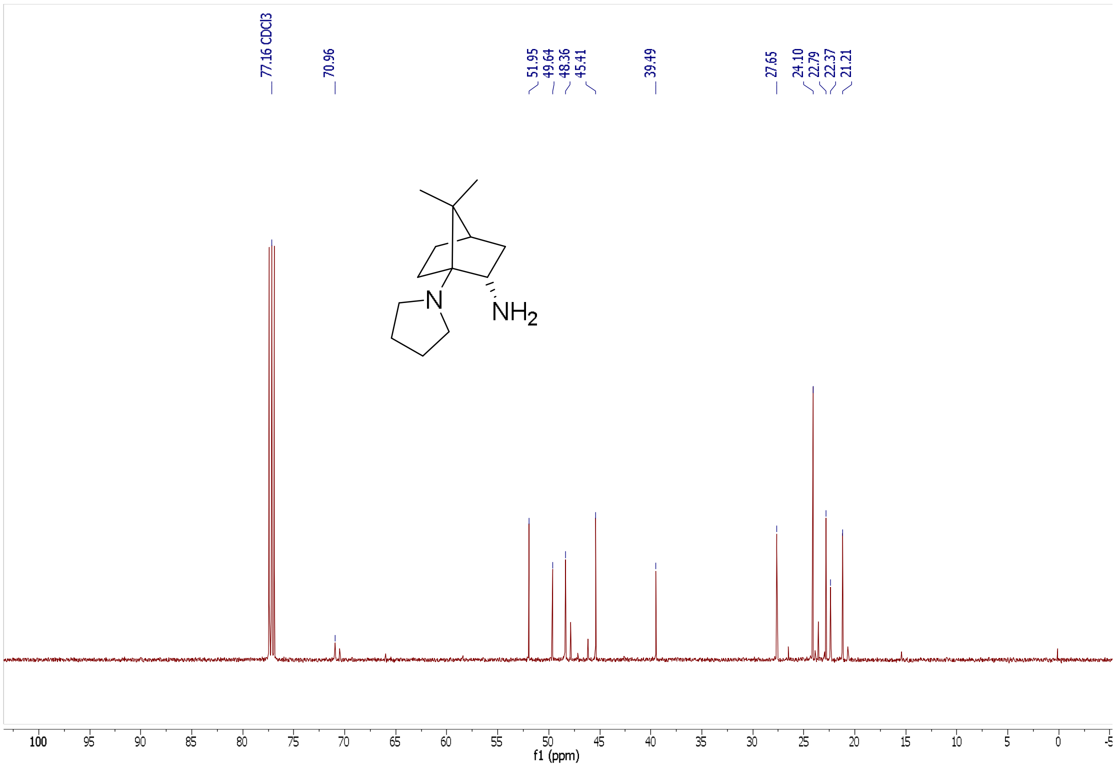

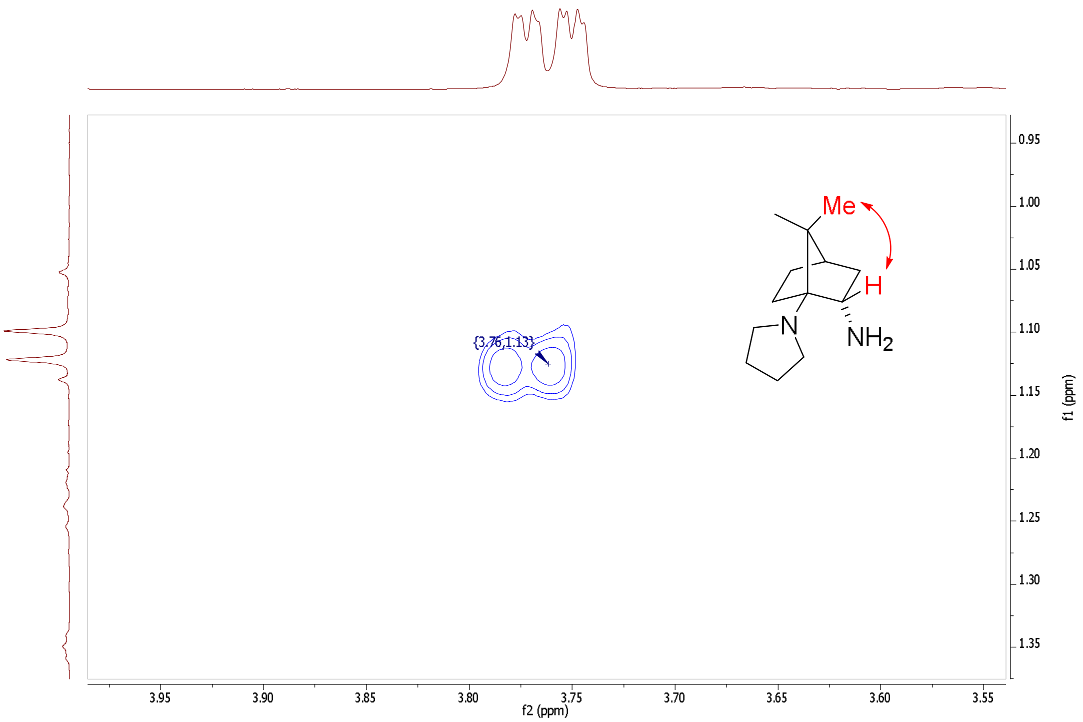

2D NOESY

(1*S*,2*R*,4*R*)-7,7-Dimethyl-1-(pyrrolidin-1-yl)bicyclo[2.2.1]heptan-2-amine (**11**)

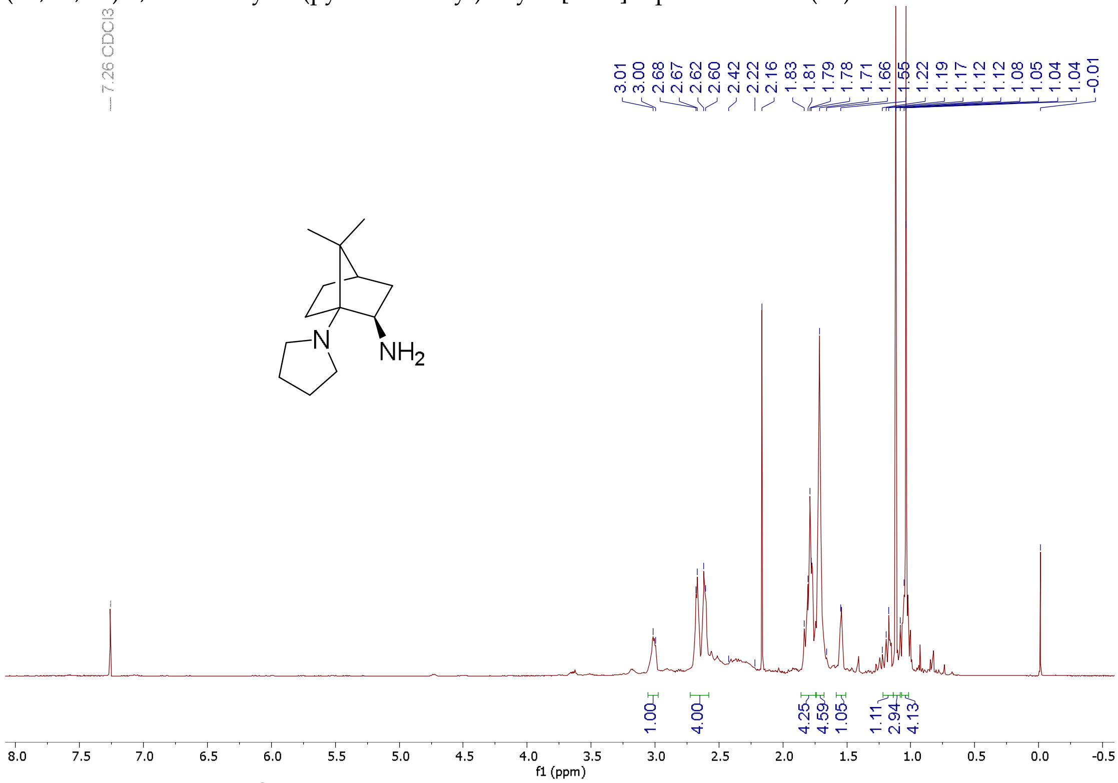

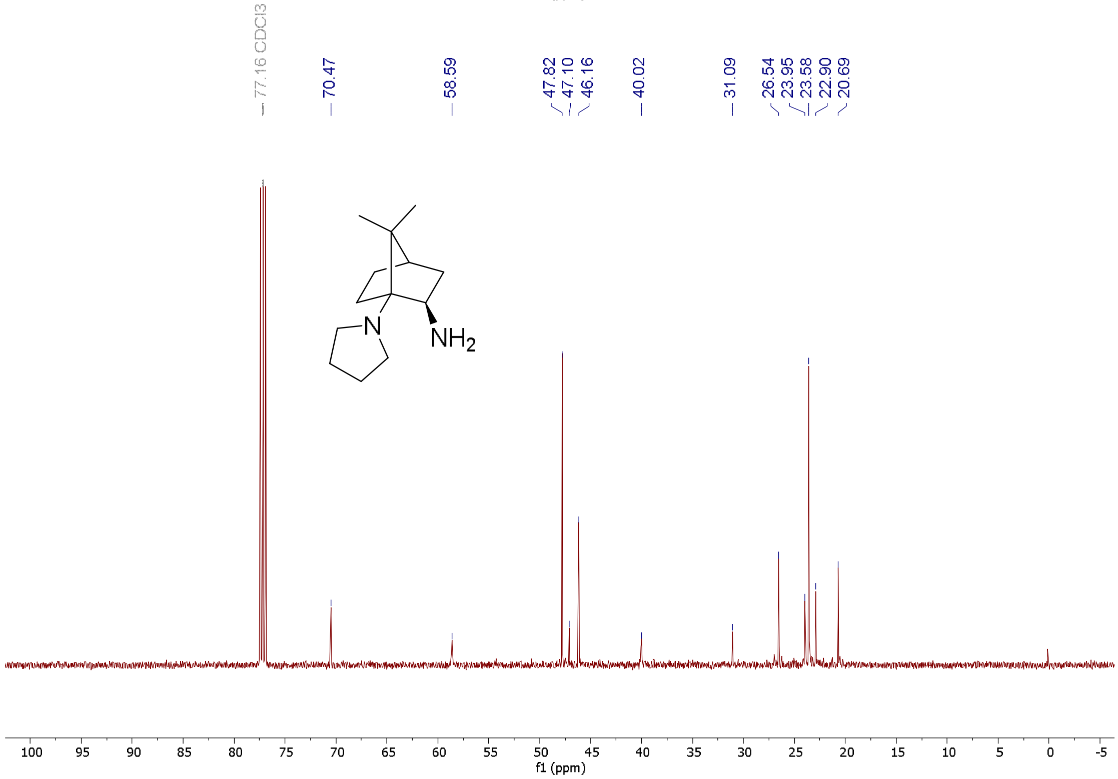

tert-Butyl ((1*S*,2*S*,4*R*)-2-amino-7,7-dimethylbicyclo[2.2.1]heptan-1-yl)carbamate (**12**)

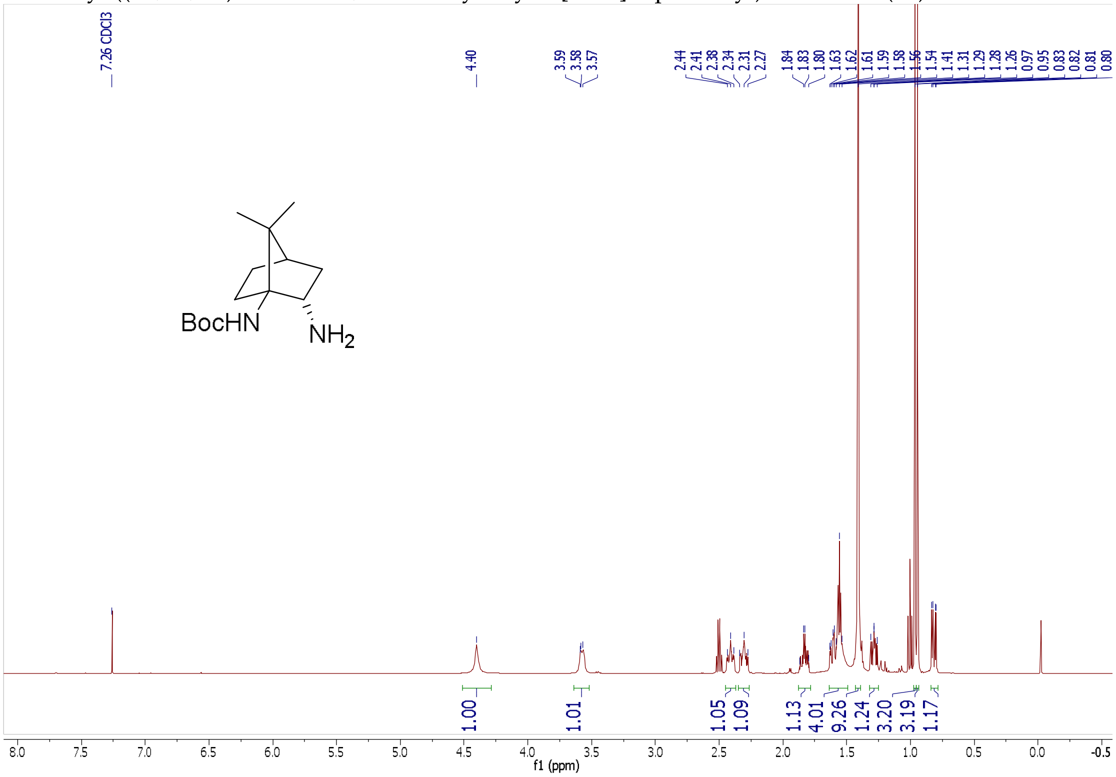

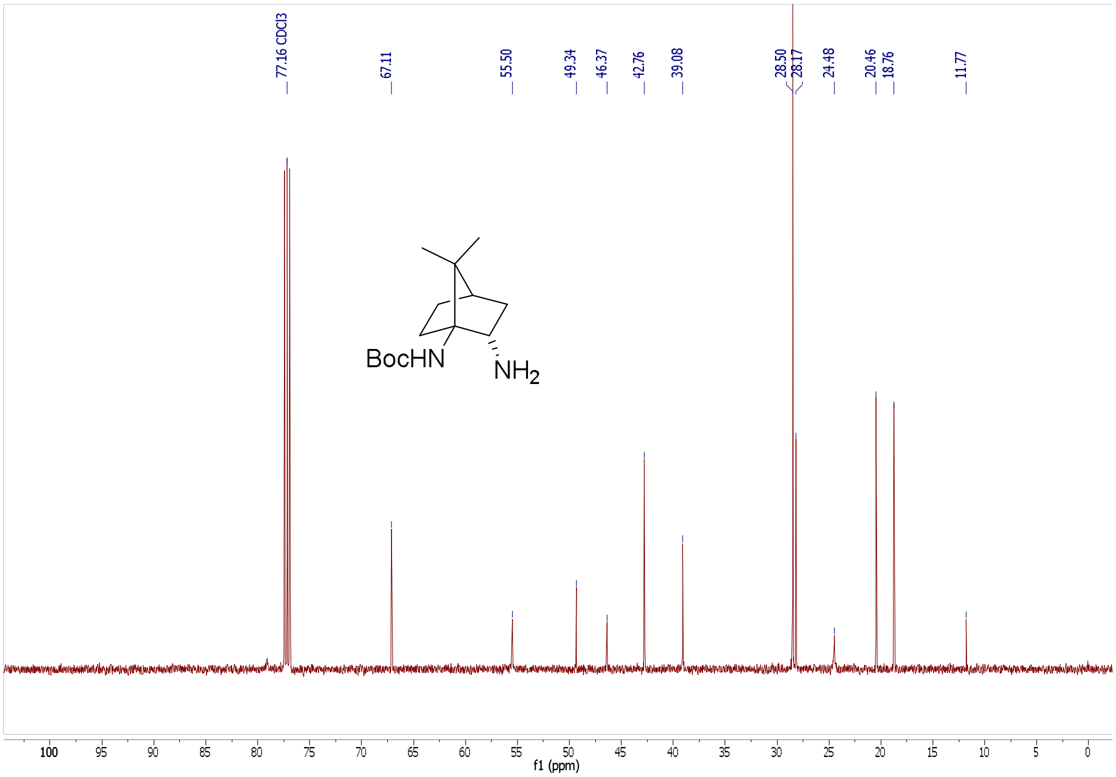

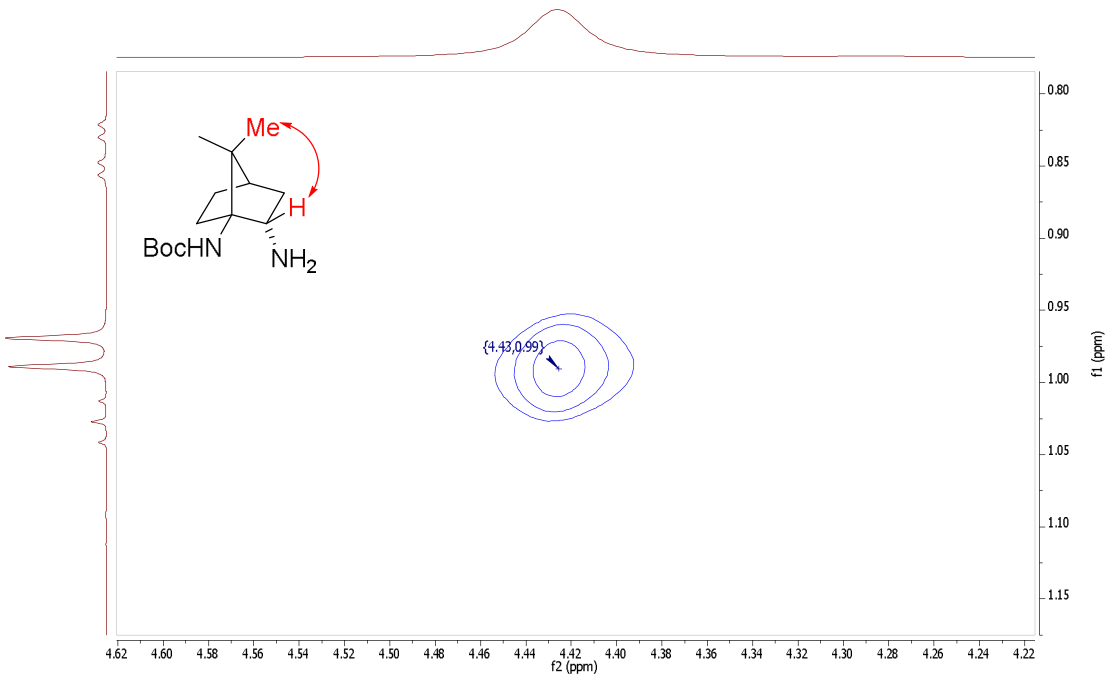
Me
H
BocHN
NH_2
{4.43,0.99}
0.80
0.85
0.90
0.95
1.00
1.05
1.10
1.15
f1 (ppm)
4.62 4.60 4.58 4.56 4.54 4.52 4.50 4.48 4.46 4.44 4.42 4.40 4.38 4.36 4.34 4.32 4.30 4.28 4.26 4.24 4.22
f2 (ppm)

2D NOESY

tert-Butyl ((1*S*,2*R*,4*R*)-2-amino-7,7-dimethylbicyclo[2.2.1]heptan-1-yl)carbamate (**13**)

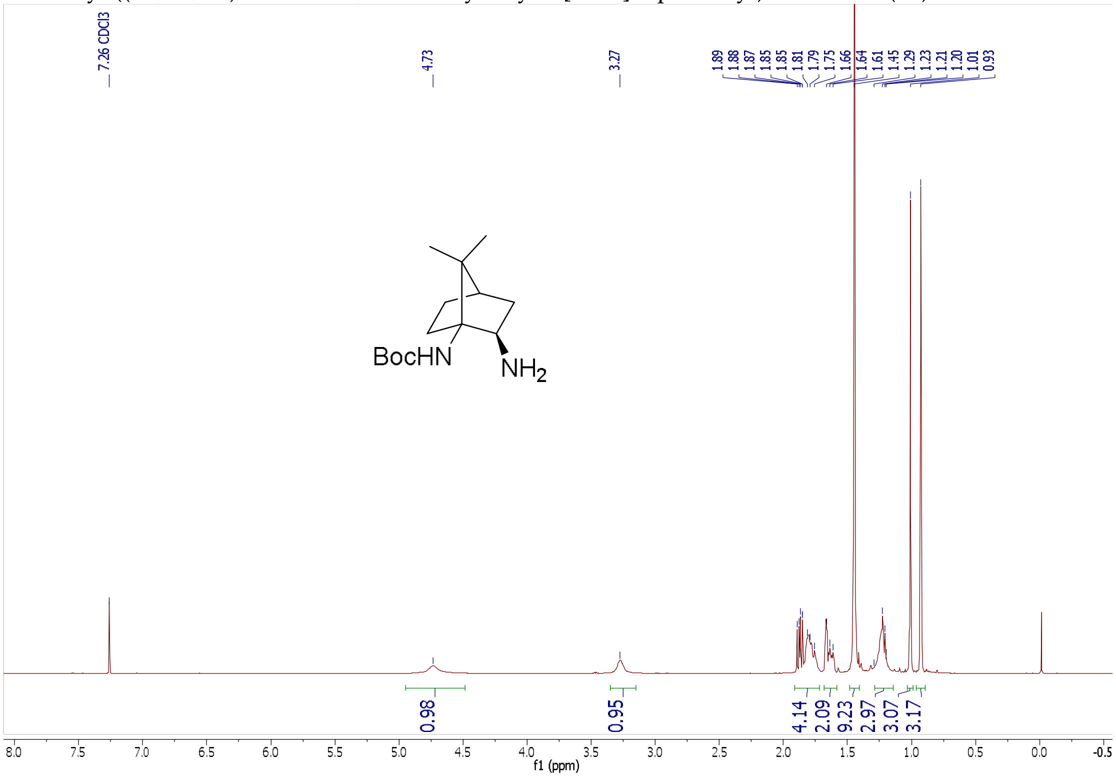

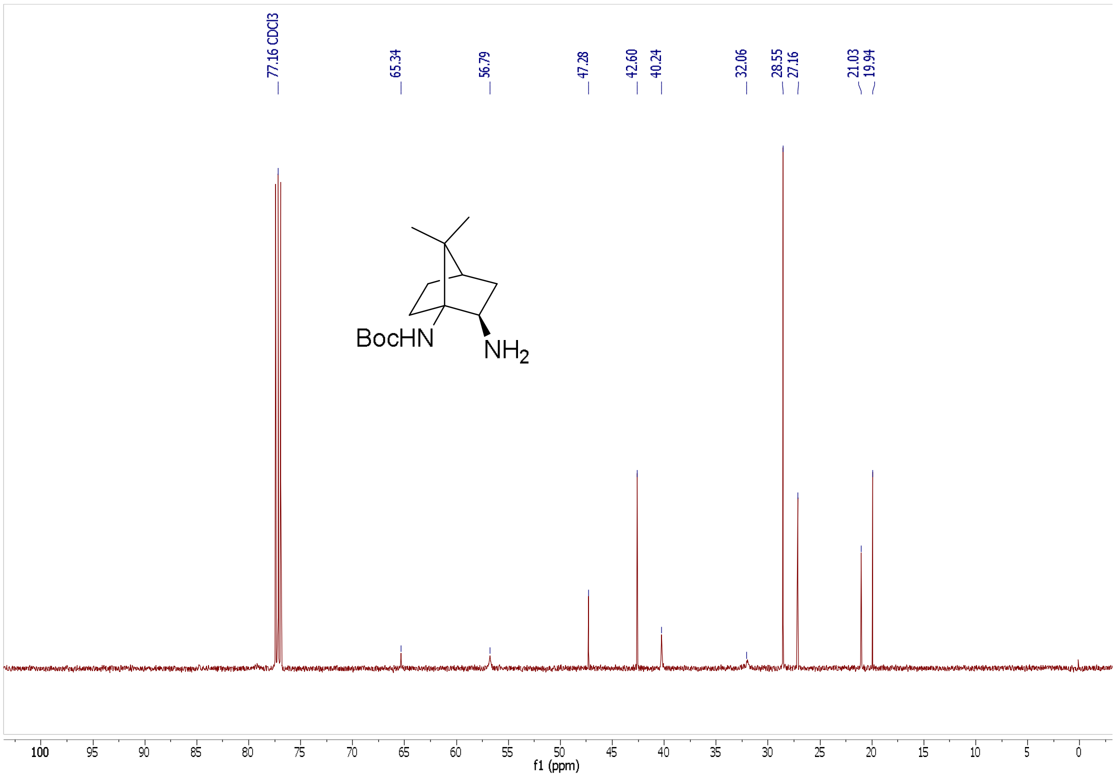

tert-Butyl ((1*S*,2*S*,4*R*)-7,7-dimethyl-2-(pyrrolidin-1-yl)bicyclo[2.2.1]heptan-1-yl)carbamate **(14)**

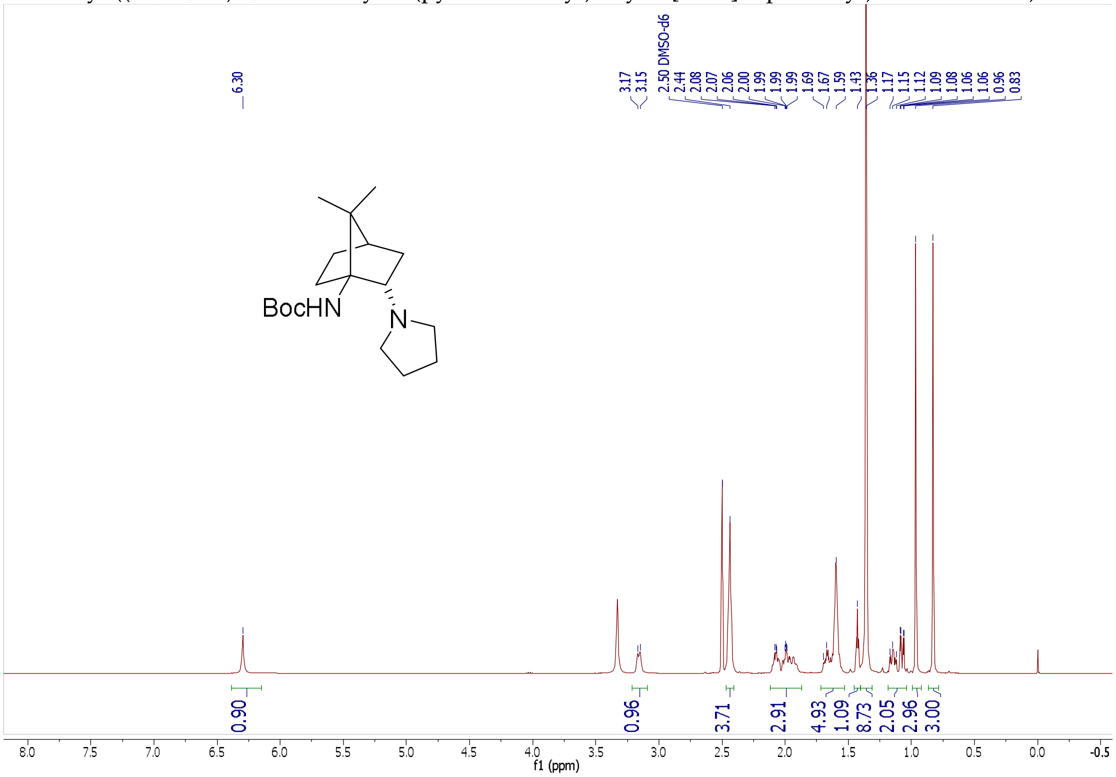

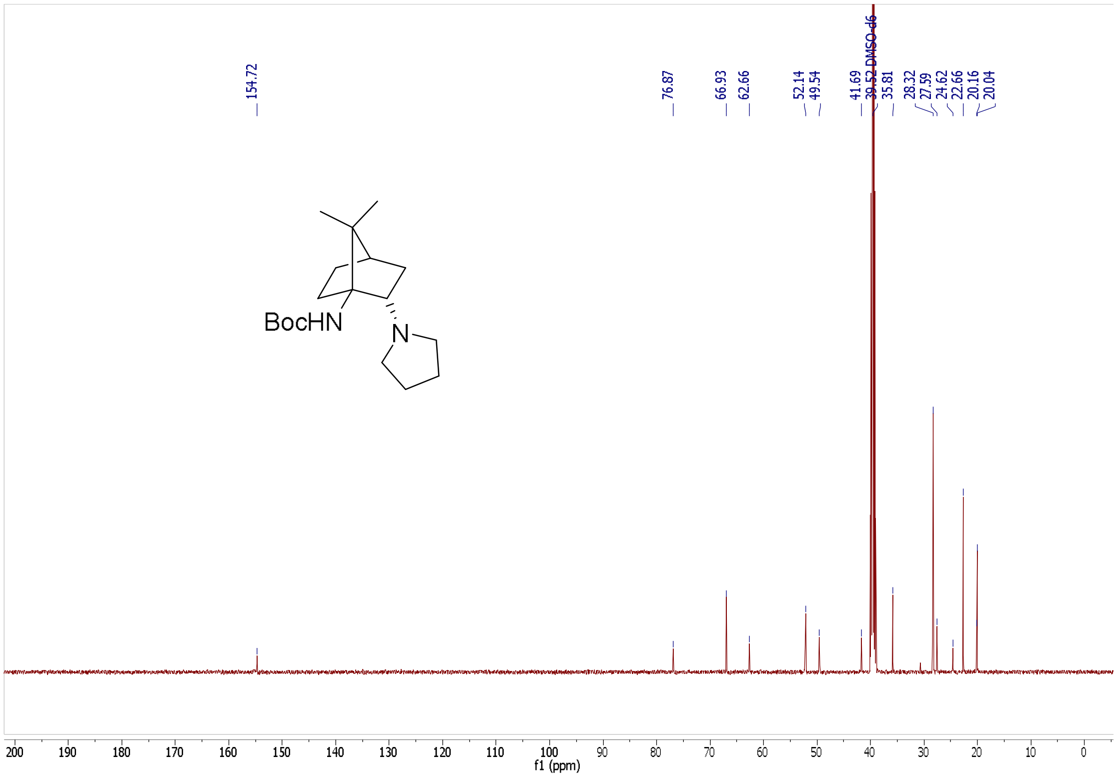

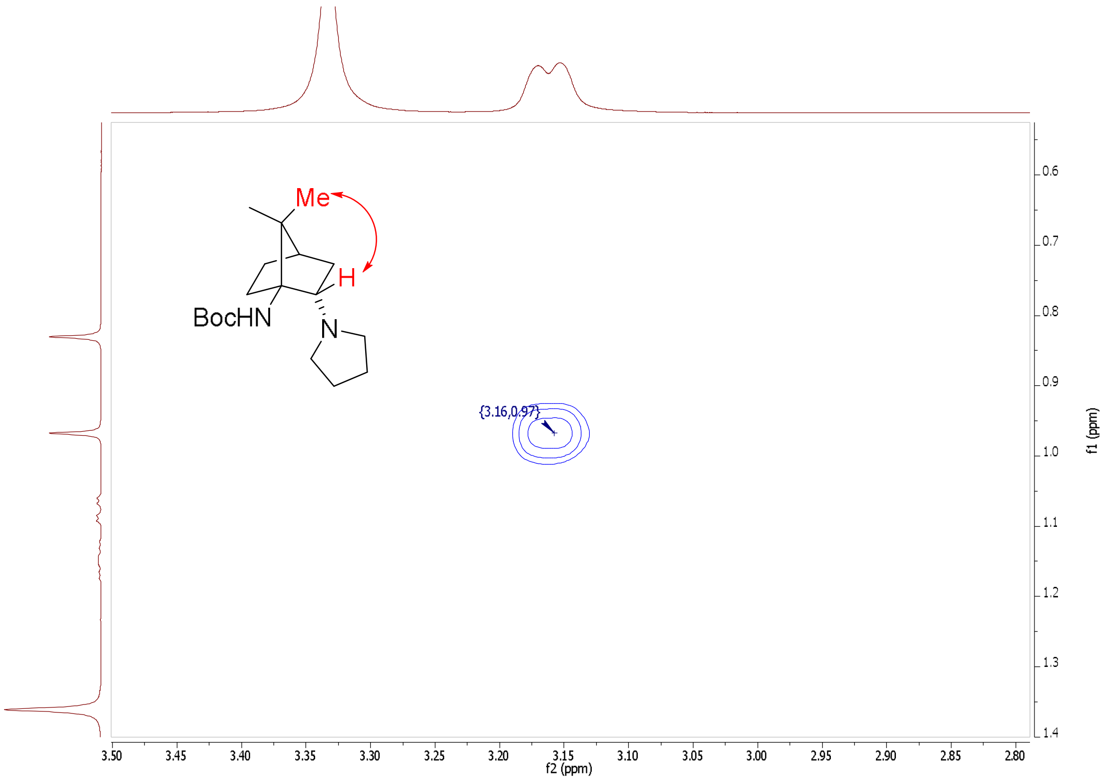

2D NOESY

((1*S*,2*R*,4*R*)-7,7-dimethyl-2-(pyrrolidin-1-yl)bicyclo[2.2.1]heptan-1-yl)carbamate (**15**)

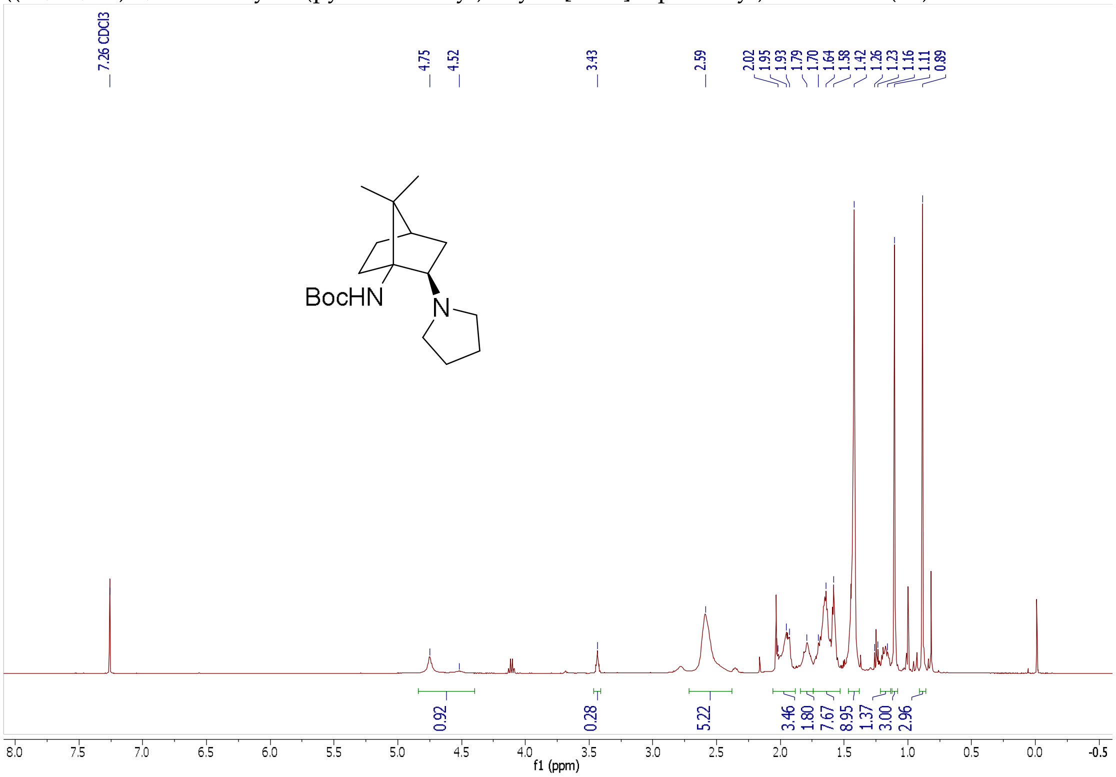

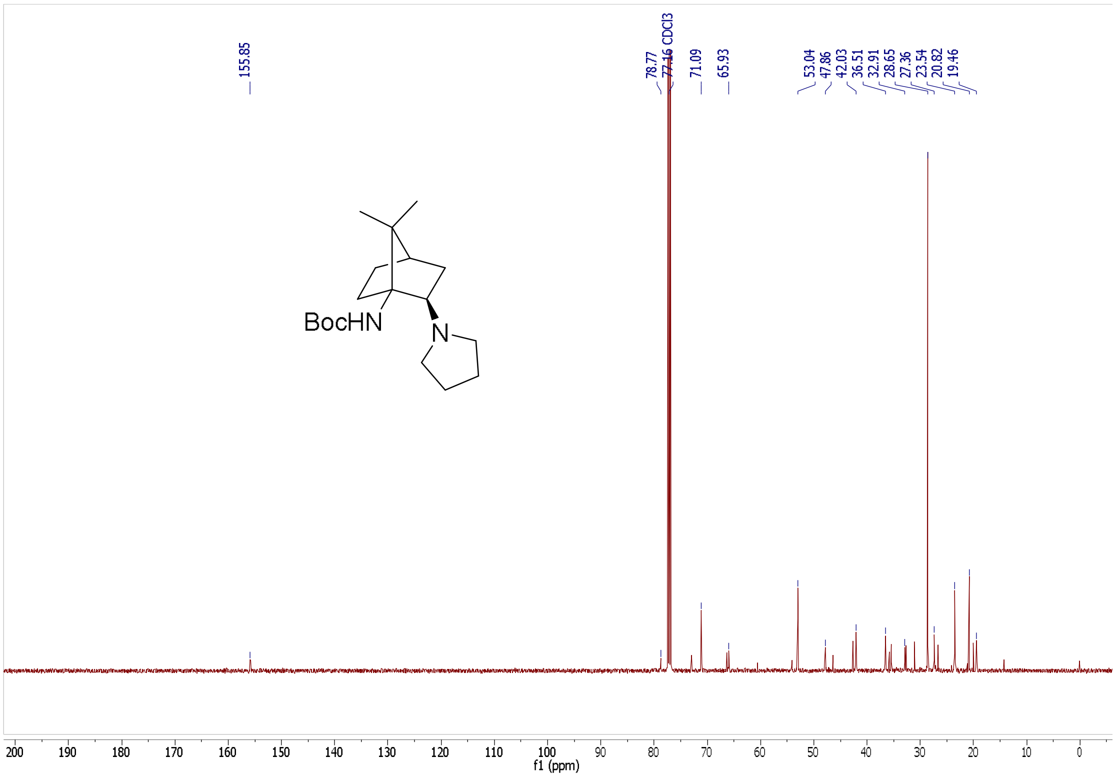

(1*S*,2*S*,4*R*)-7,7-Dimethyl-2-(pyrrolidin-1-yl)bicyclo[2.2.1]heptan-1-amine (**16**)

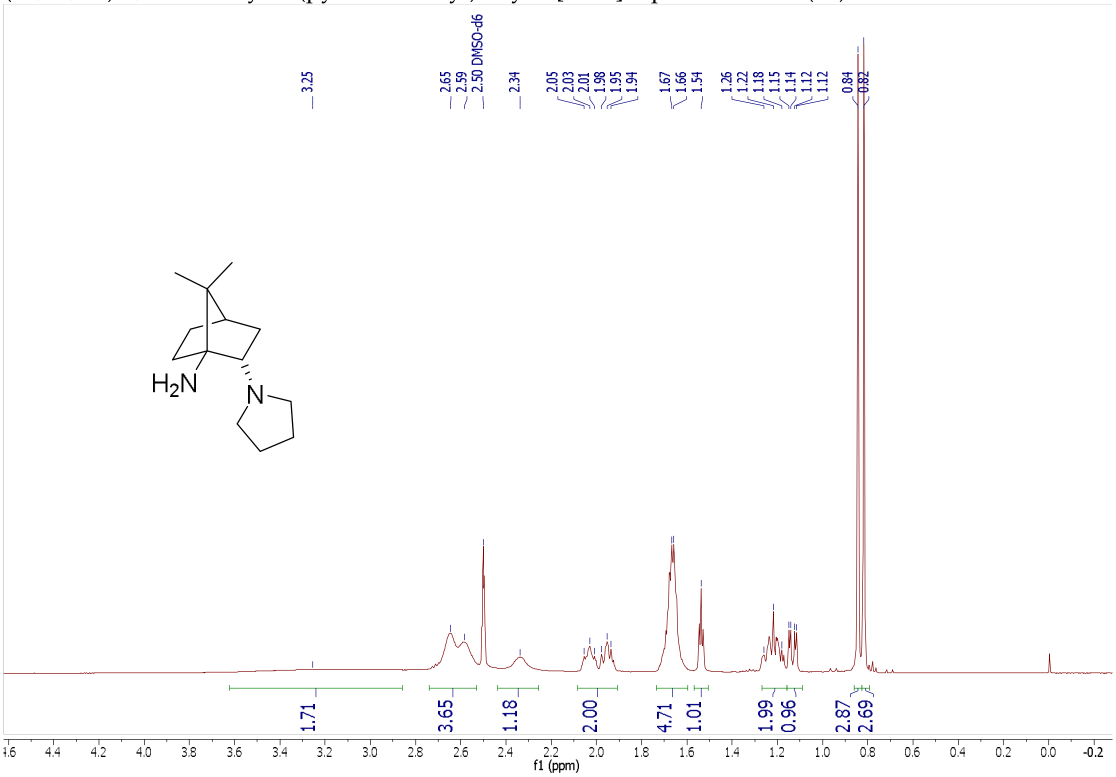

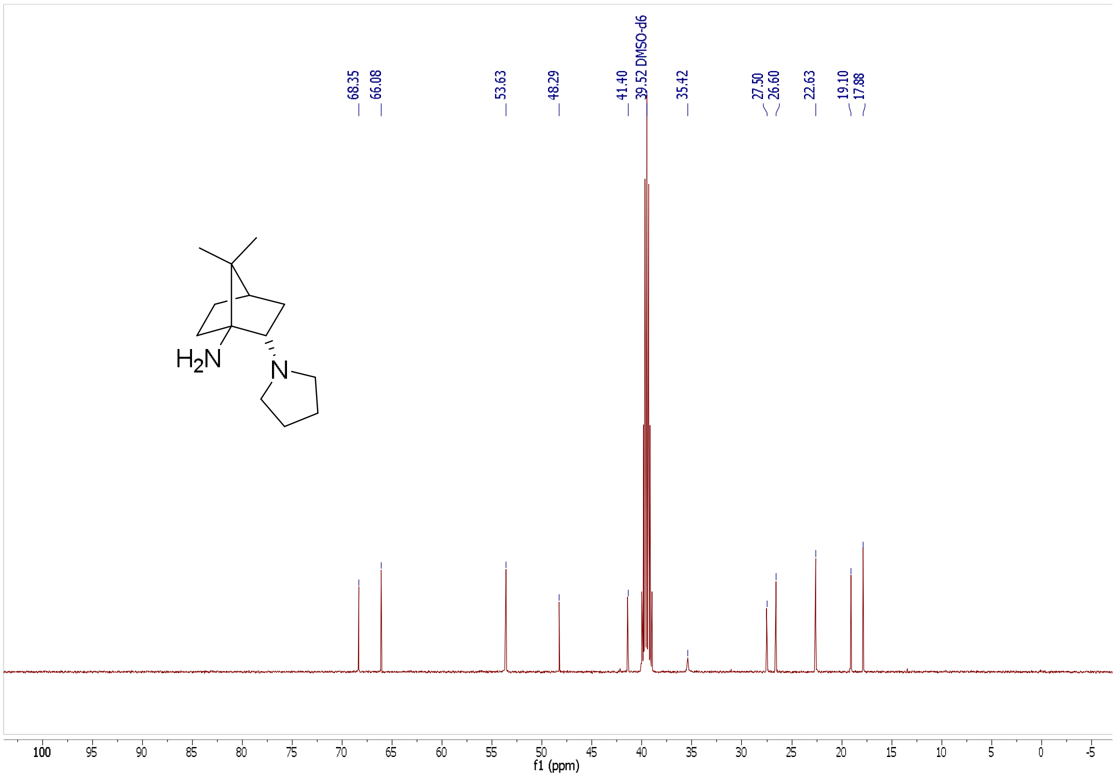

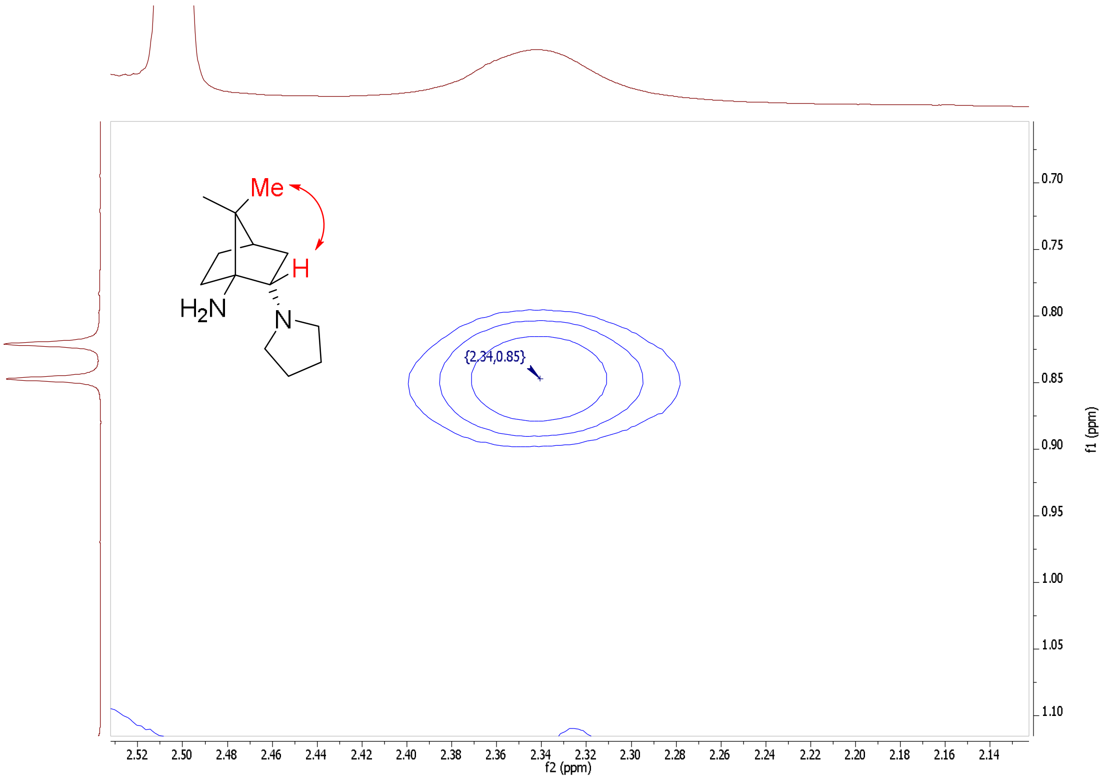
Me
H
H2N
N
{2.34,0.85}
0.70
0.75
0.80
0.85
0.90
0.95
1.00
1.05
1.10
f1 (ppm)
2.52
2.50
2.48
2.46
2.44
2.42
2.40
2.38
2.36
2.34
2.32
2.30
2.28
2.26
2.24
2.22
2.20
2.18
2.16
2.14
f2 (ppm)

2D NOESY

(1*R*,4*R*,*E*)-7,7-Dimethyl-1-(piperidin-1-ylmethyl)bicyclo[2.2.1]heptan-2-one oxime (**21b**)

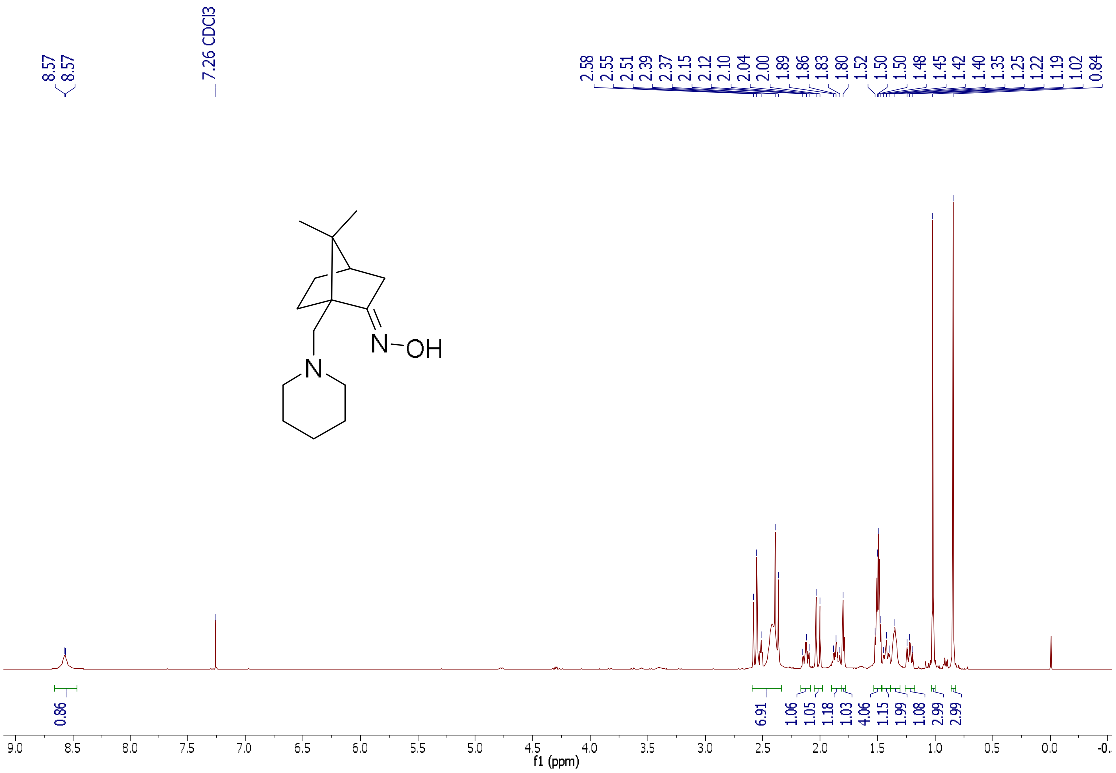

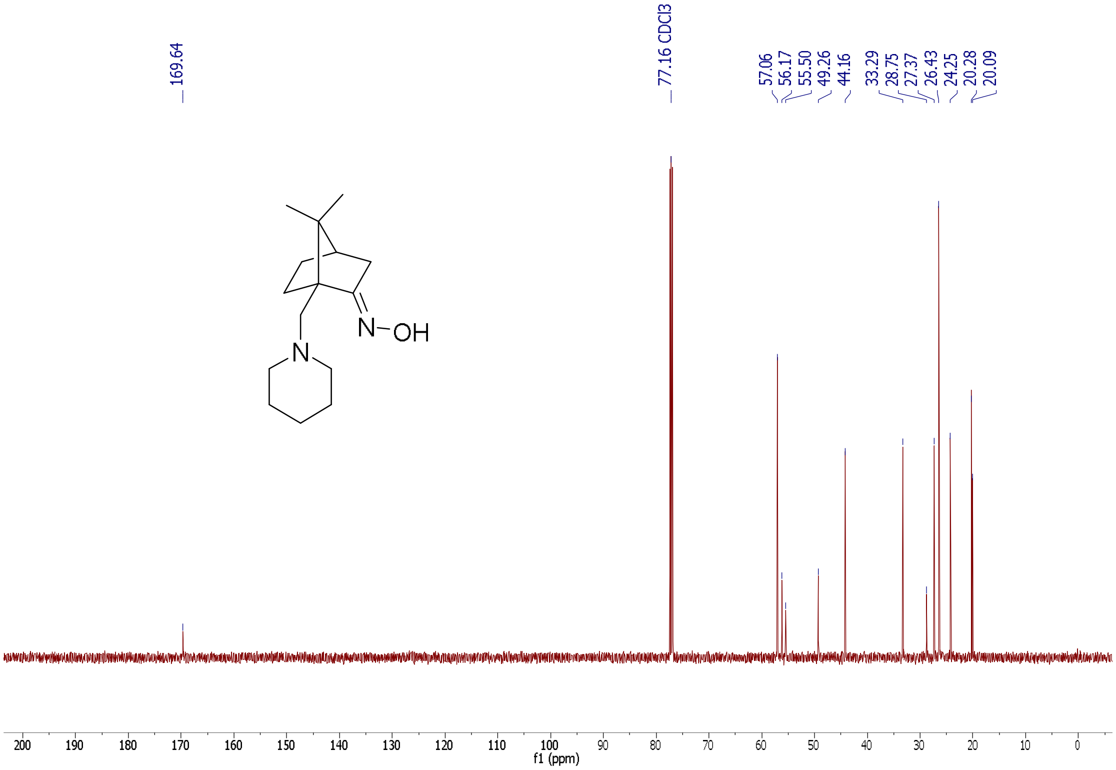

(1*R*,4*R*,*E*)-7,7-Dimethyl-1-(morpholinomethyl)bicyclo[2.2.1]heptan-2-one oxime (**21c**)

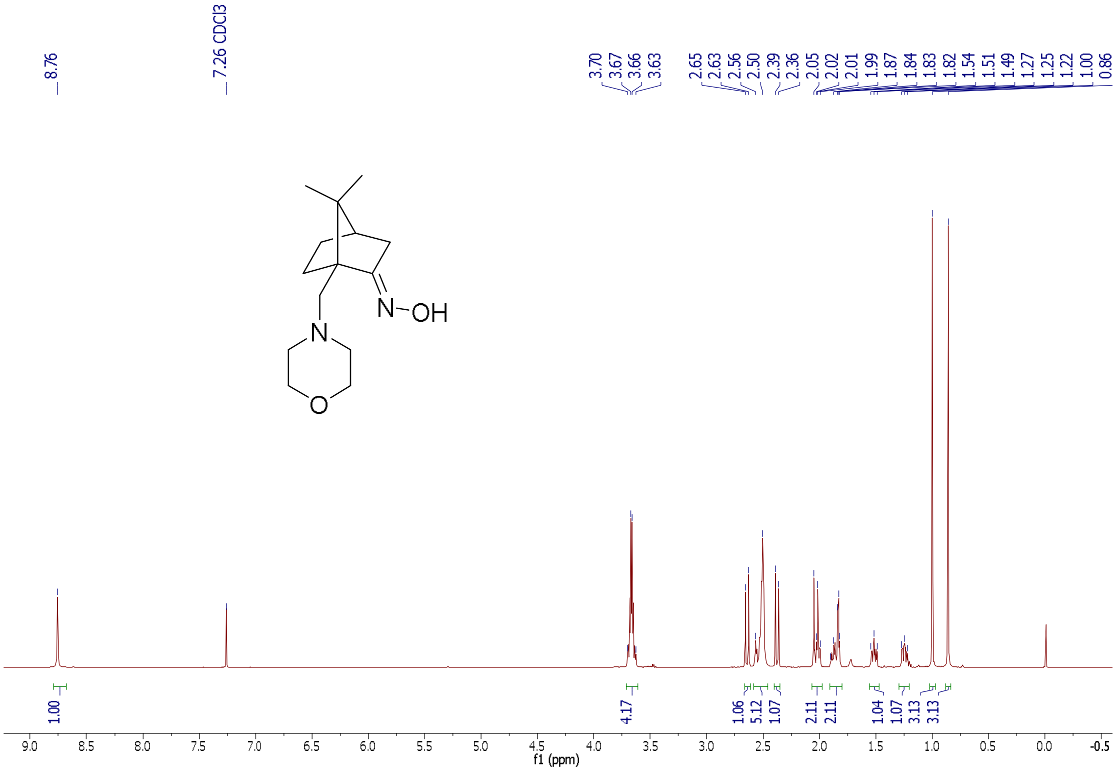

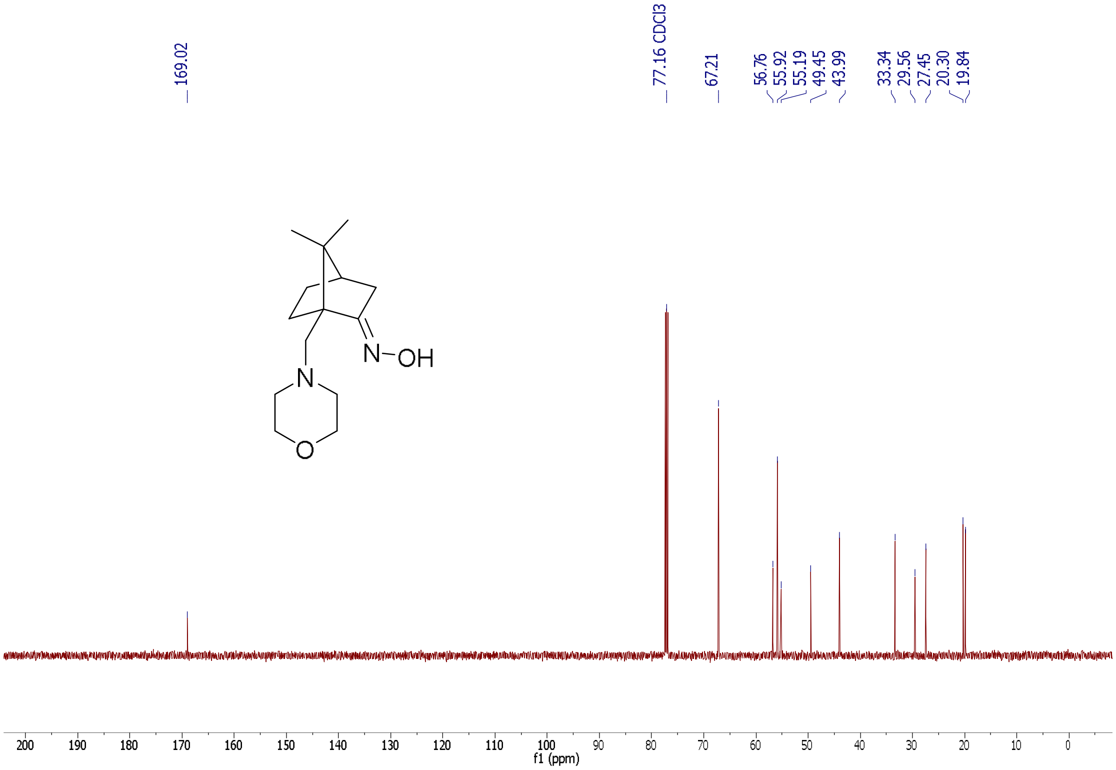

(1*R*,4*R*,*E*)-1-((Dimethylamino)methyl)-7,7-dimethylbicyclo[2.2.1]heptan-2-one oxime (**21d**)

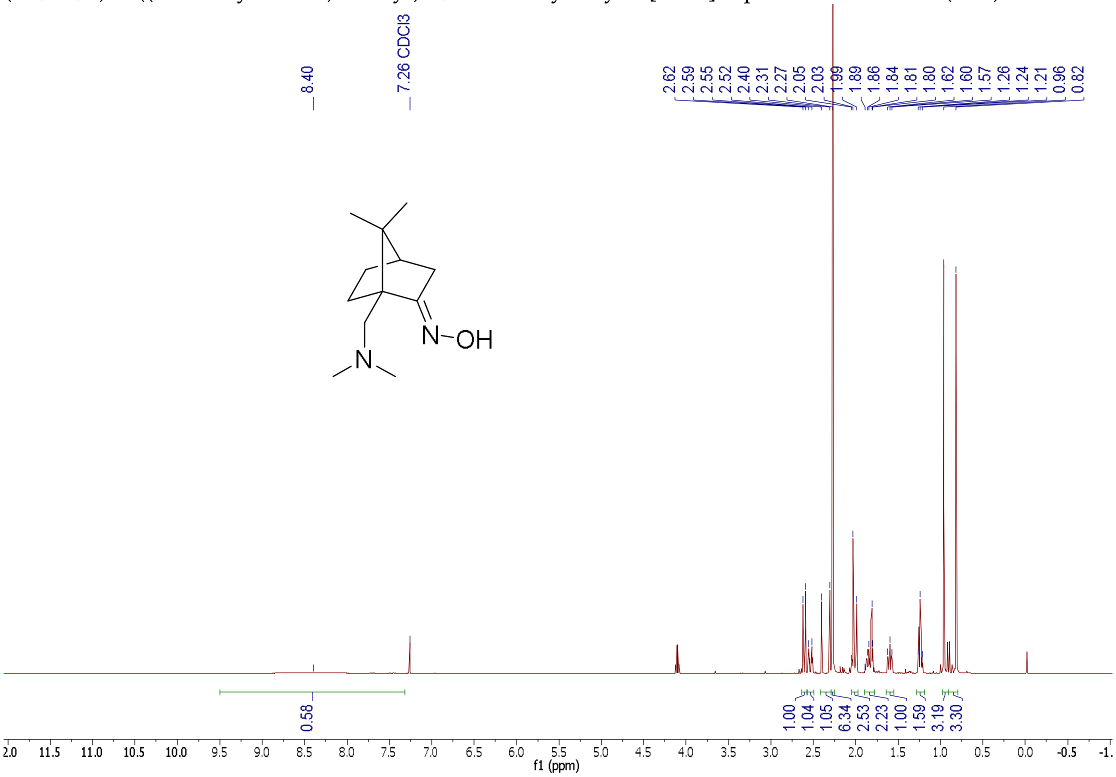

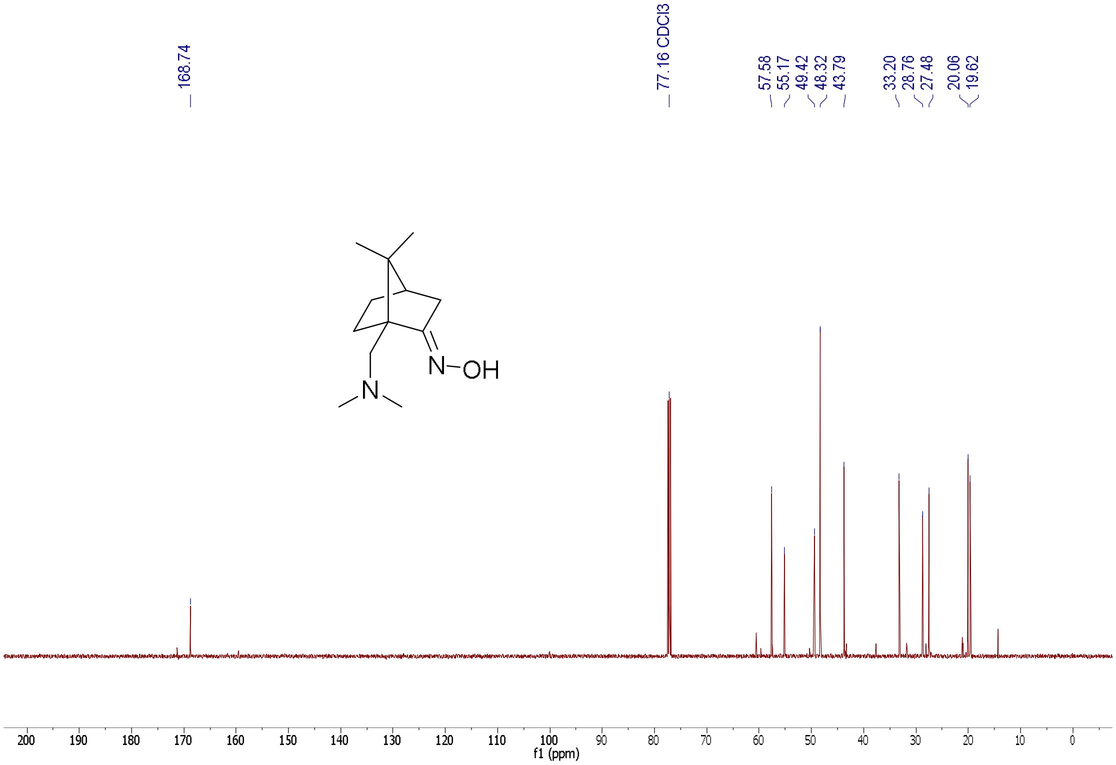

(1*R*,2*S*,4*R*)-7,7-Dimethyl-1-(piperidin-1-ylmethyl)bicyclo[2.2.1]heptan-2-amine (**22b**) and (1*R*,2*R*,4*R*)-7,7-dimethyl-1-(piperidin-1-ylmethyl)bicyclo[2.2.1]heptan-2-amine (**23b**)

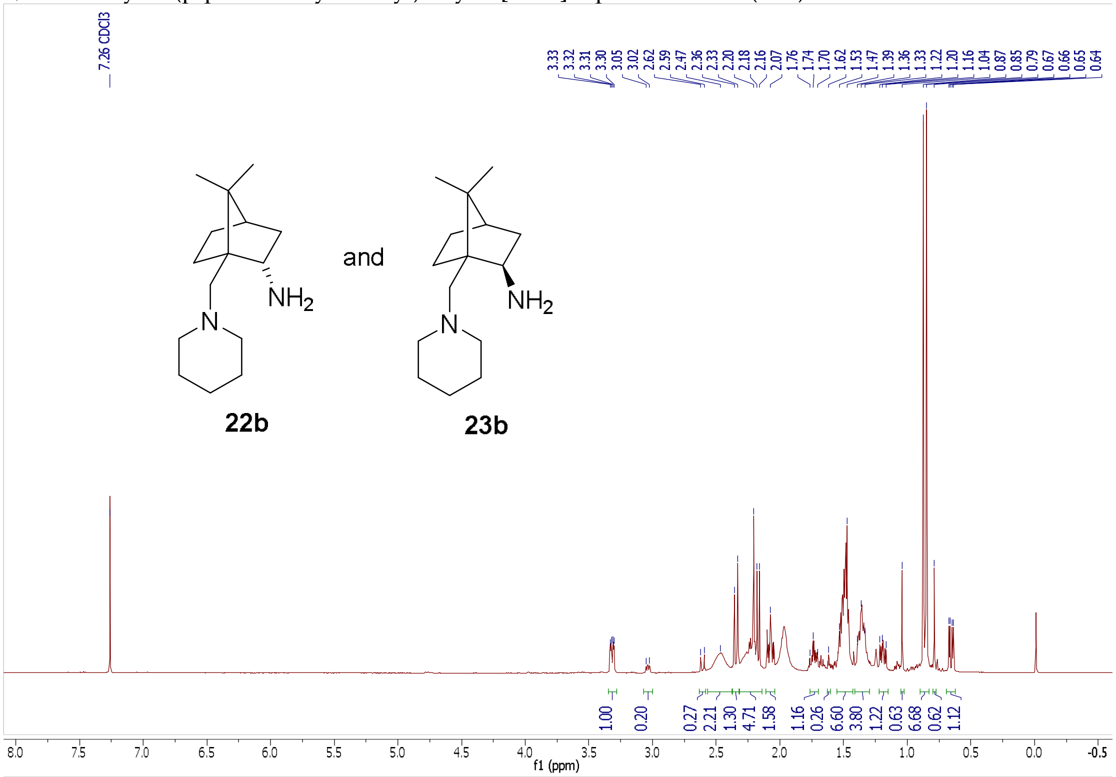

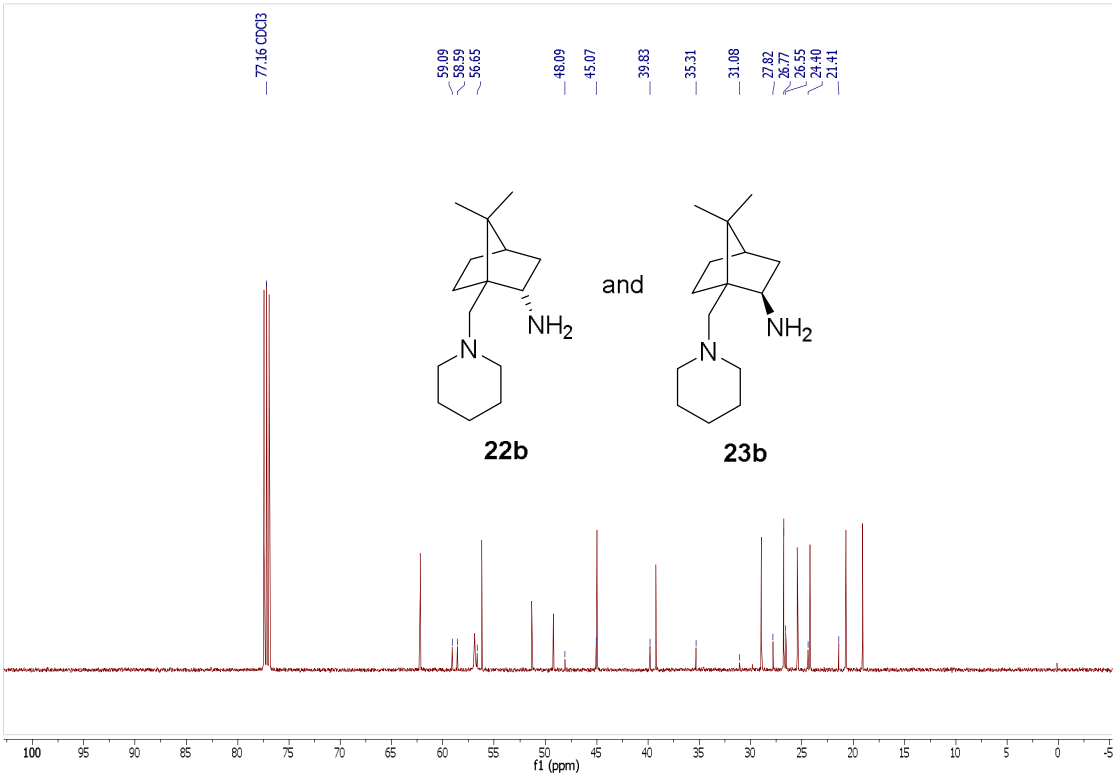

(1*R*,2*S*,4*R*)-7,7-Dimethyl-1-(morpholinomethyl)bicyclo[2.2.1]heptan-2-amine (**22c**) and (1*R*,2*R*,4*R*)-7,7-dimethyl-1-(morpholinomethyl)bicyclo[2.2.1]heptan-2-amine (**23c**)

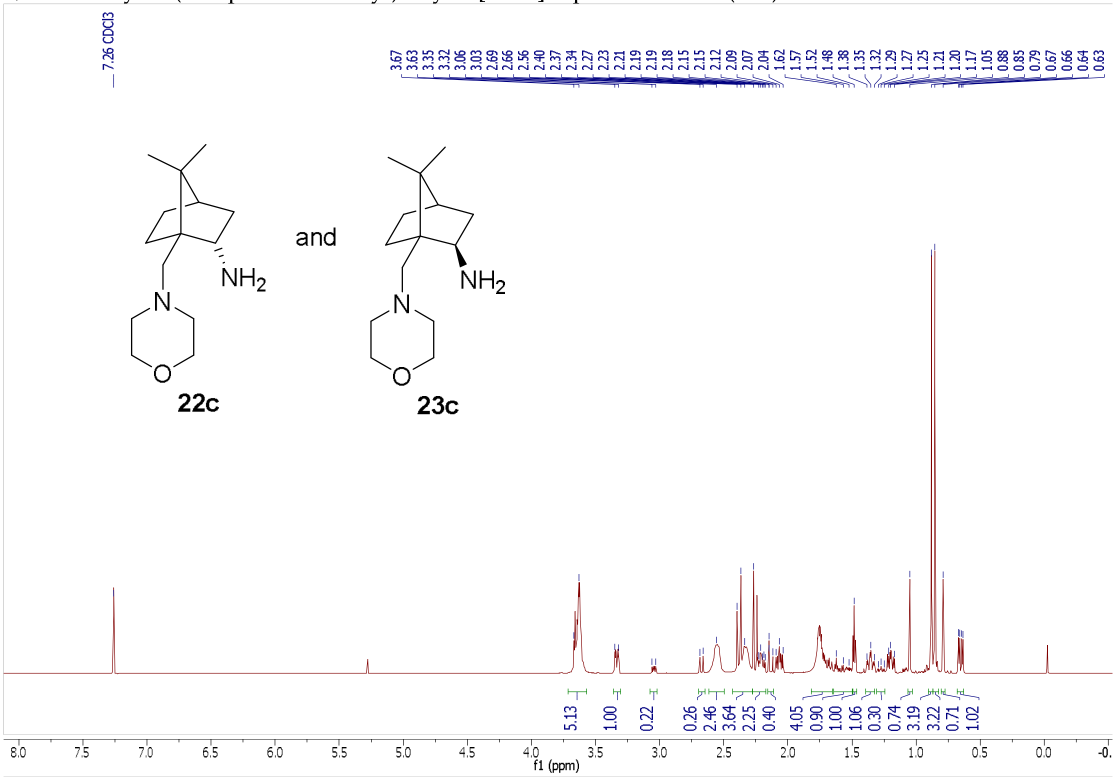

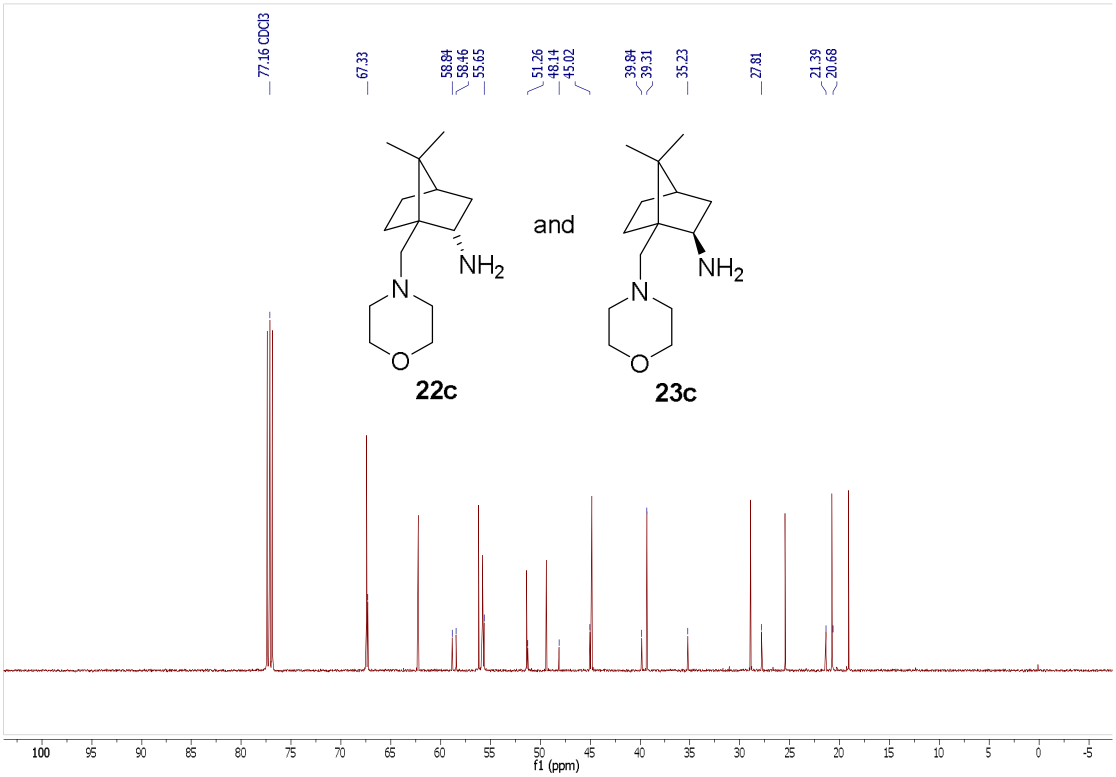

(1*R*,2*S*,4*R*)-1-((Dimethylamino)methyl)-7,7-dimethylbicyclo[2.2.1]heptan-2-amine (**22d**) and (1*R*,2*R*,4*R*)-1-((dimethylamino)methyl)-7,7-dimethylbicyclo[2.2.1]heptan-2-amine (**23d**)

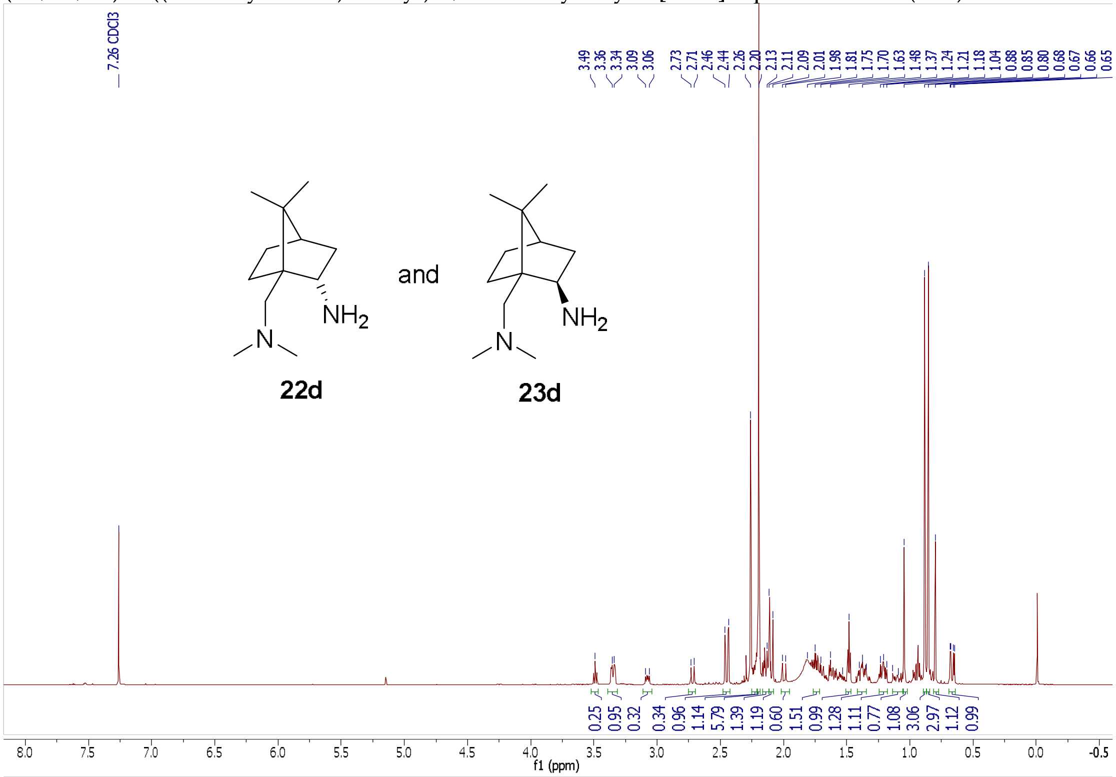

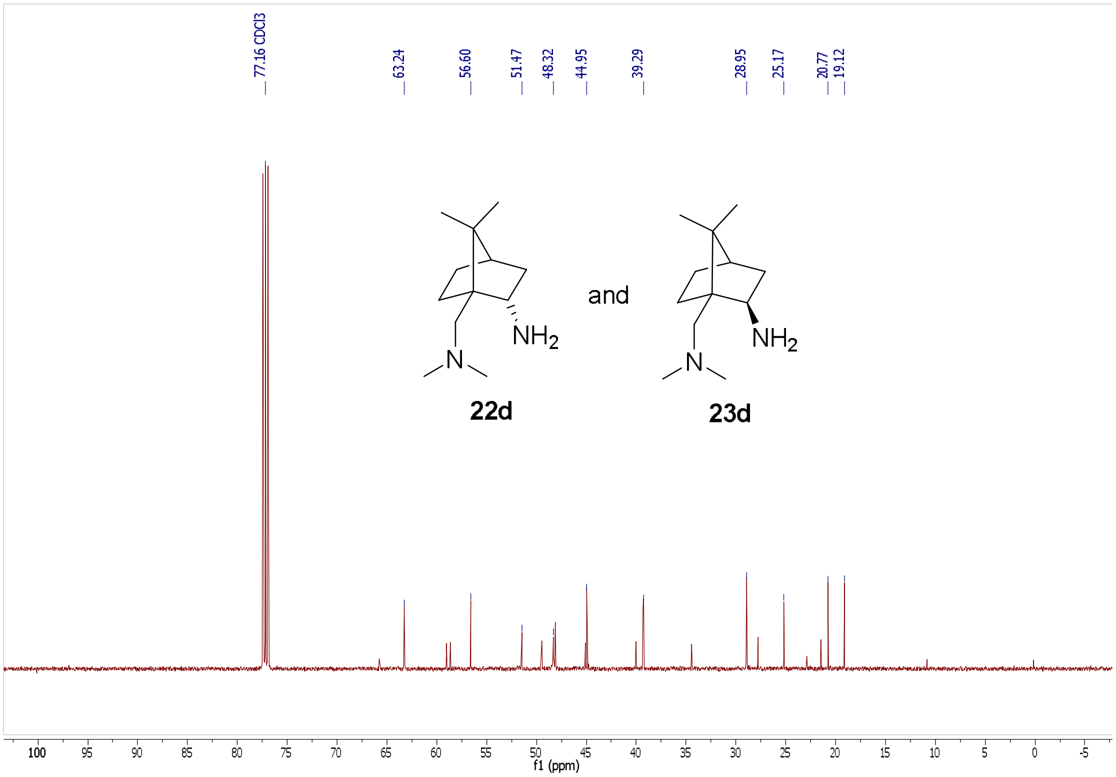

(1*S*,4*R*,*E*)-1-(Iodomethyl)-7,7-dimethylbicyclo[2.2.1]heptan-2-one oxime (**24**)

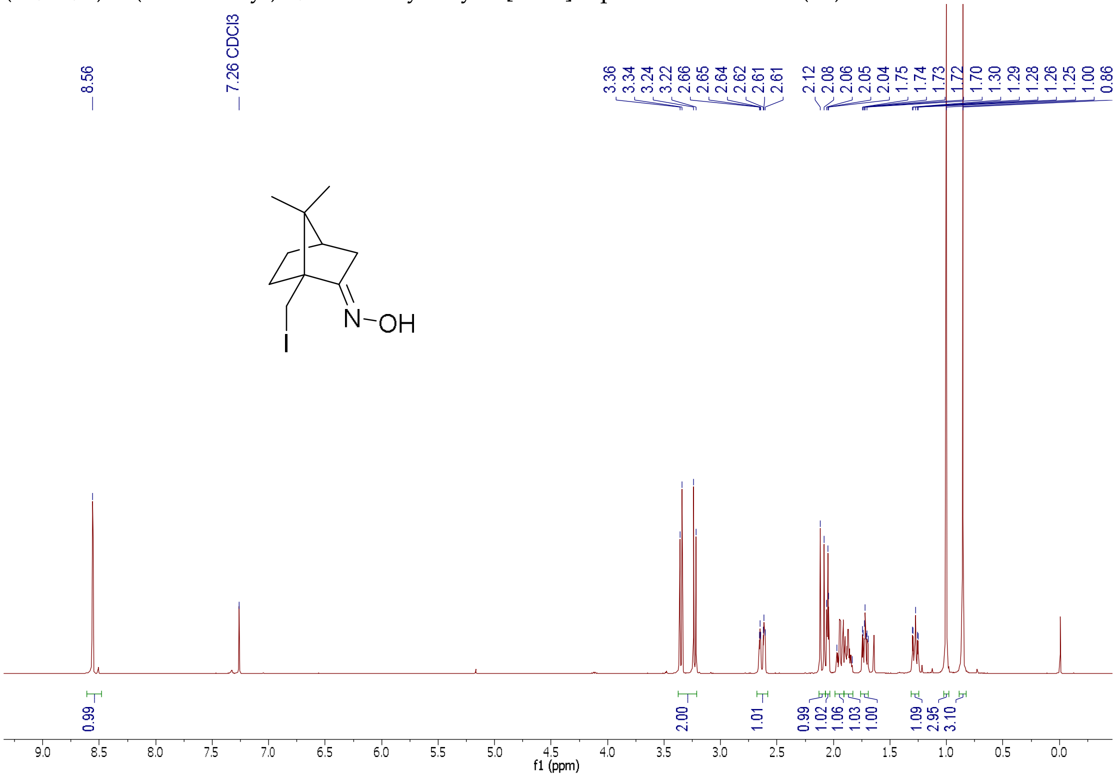

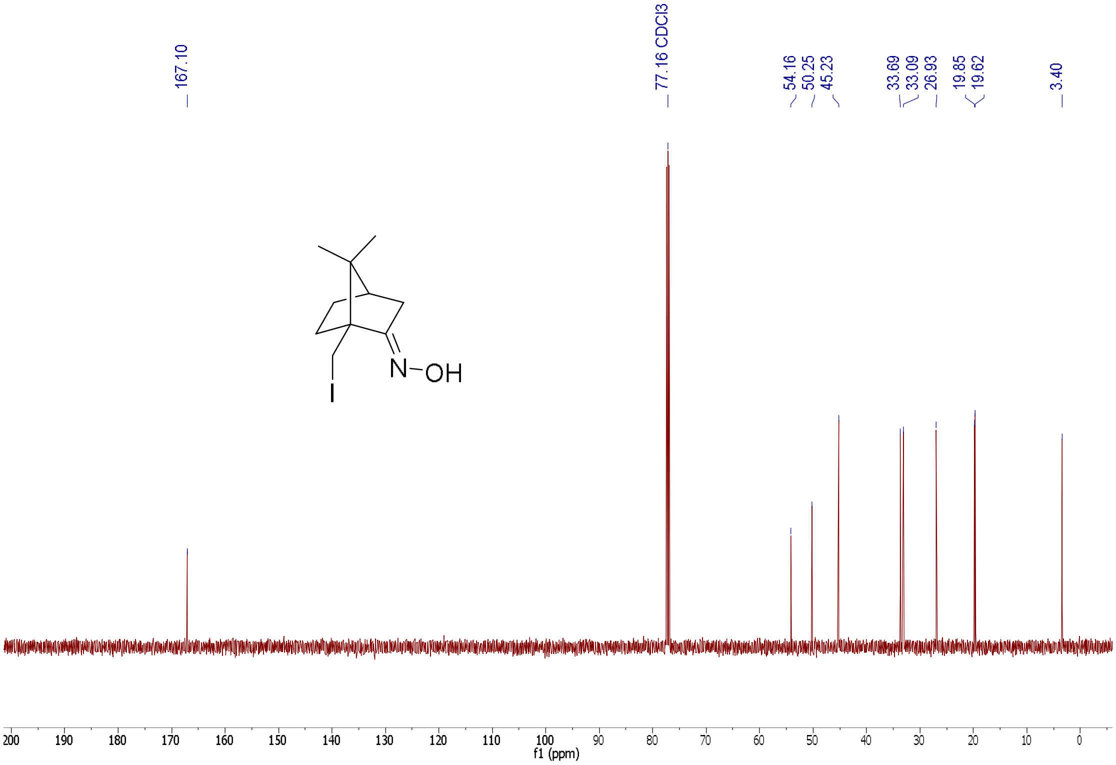

(1*R*,4*R*,*E*)-1-(Azidomethyl)-7,7-dimethylbicyclo[2.2.1]heptan-2-one oxime (**25**)

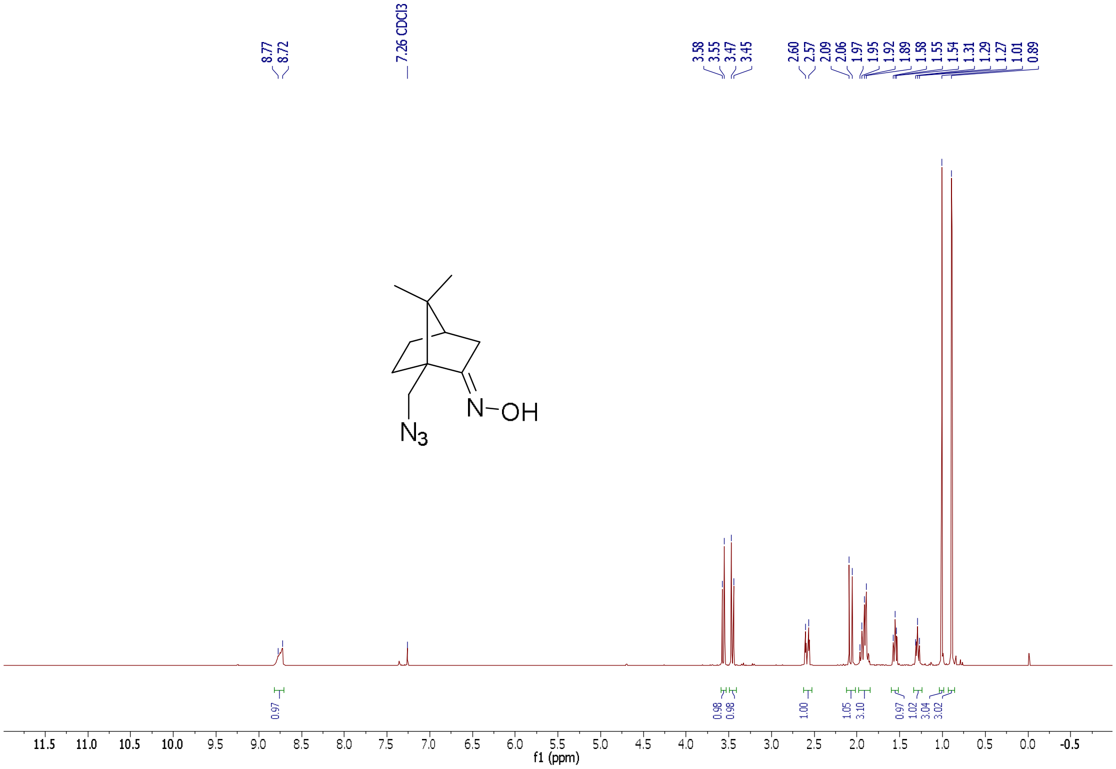

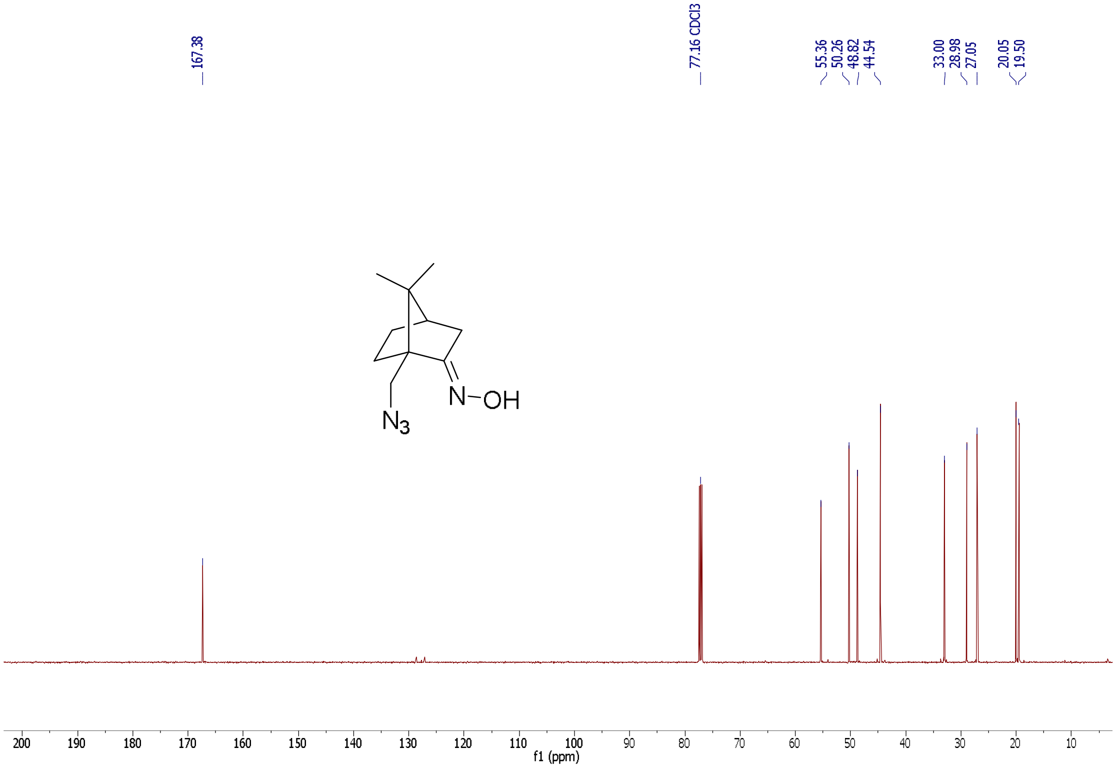

(1*R*,4*R*,E)-1-(Aminomethyl)-7,7-dimethylbicyclo[2.2.1]heptan-2-one oxime (**26**)

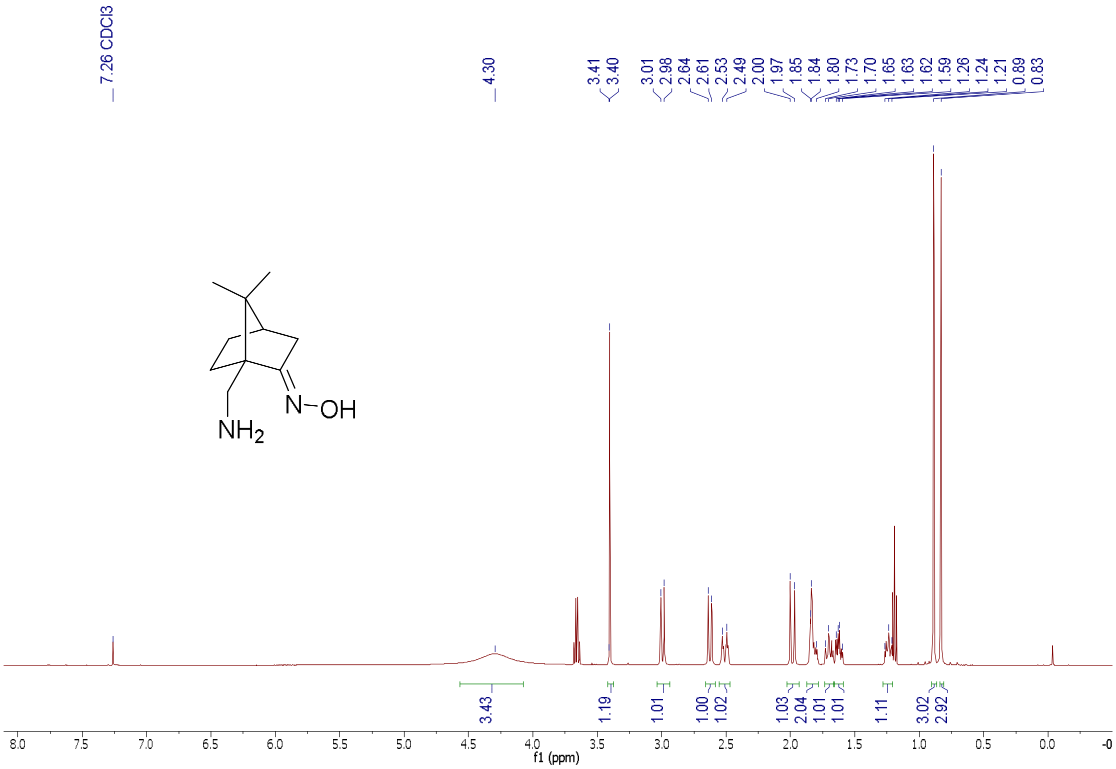

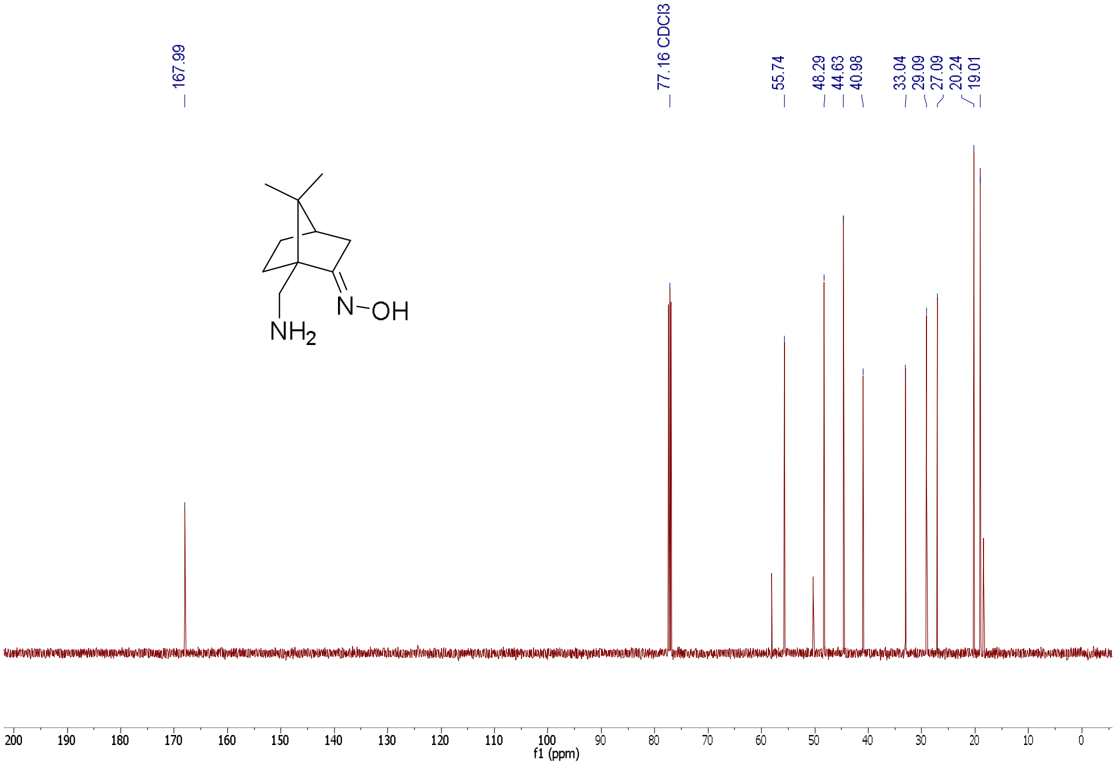

tert-Butyl (((1*R*,4*R*,*E*)-2-(((tert-butoxycarbonyl)oxy)imino)-7,7-dimethylbicyclo[2.2.1]heptan-1-yl)methyl)carbamate (**27**)

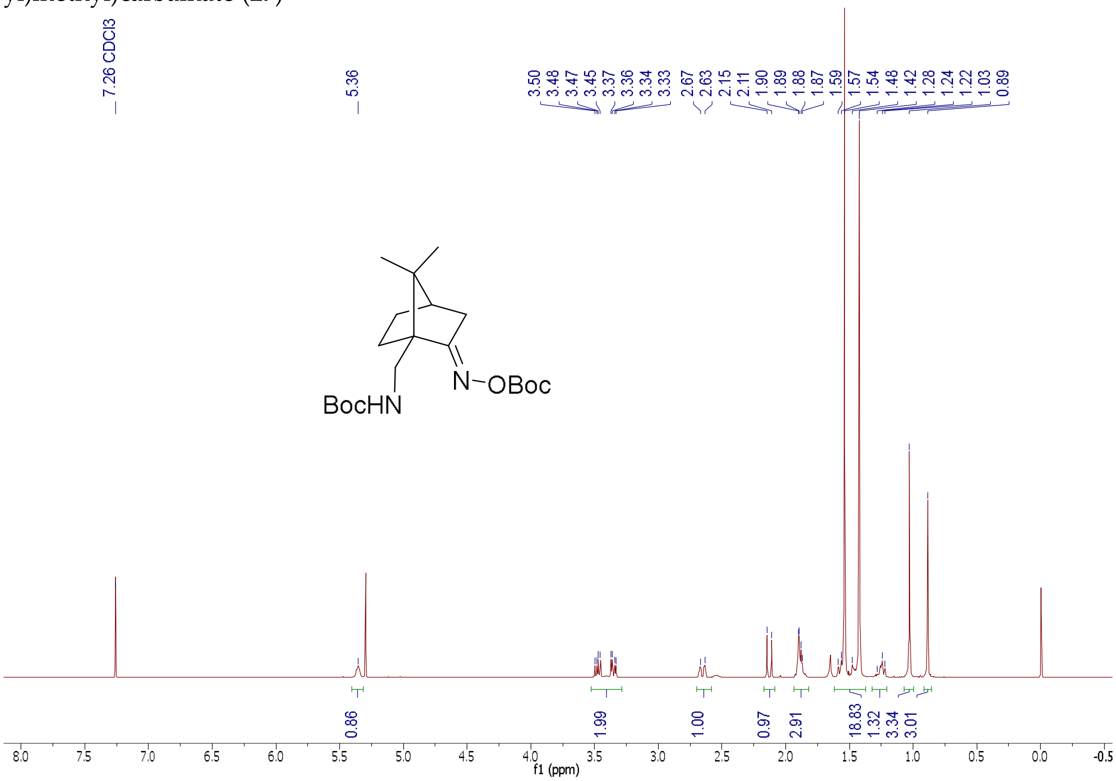

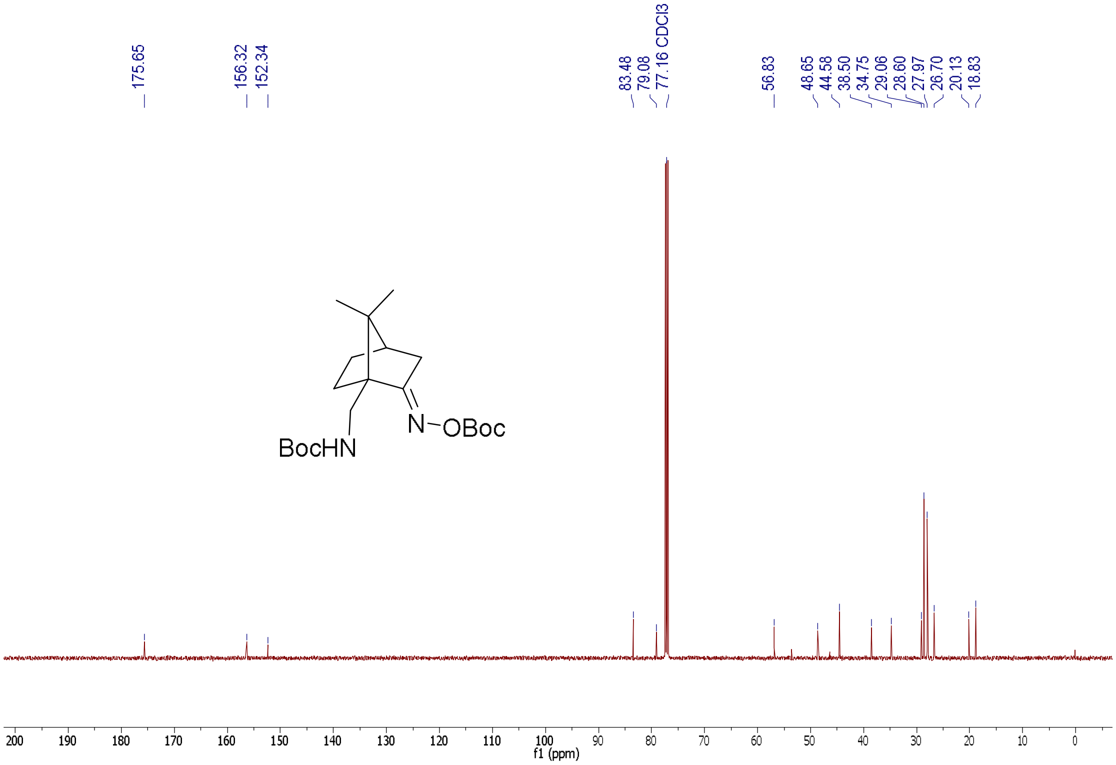

tert-Butyl (((1*R*,4*R*,*E*)-2-(hydroxyimino)-7,7-dimethylbicyclo[2.2.1]heptan-1-yl)methyl)carbamate (**28**)

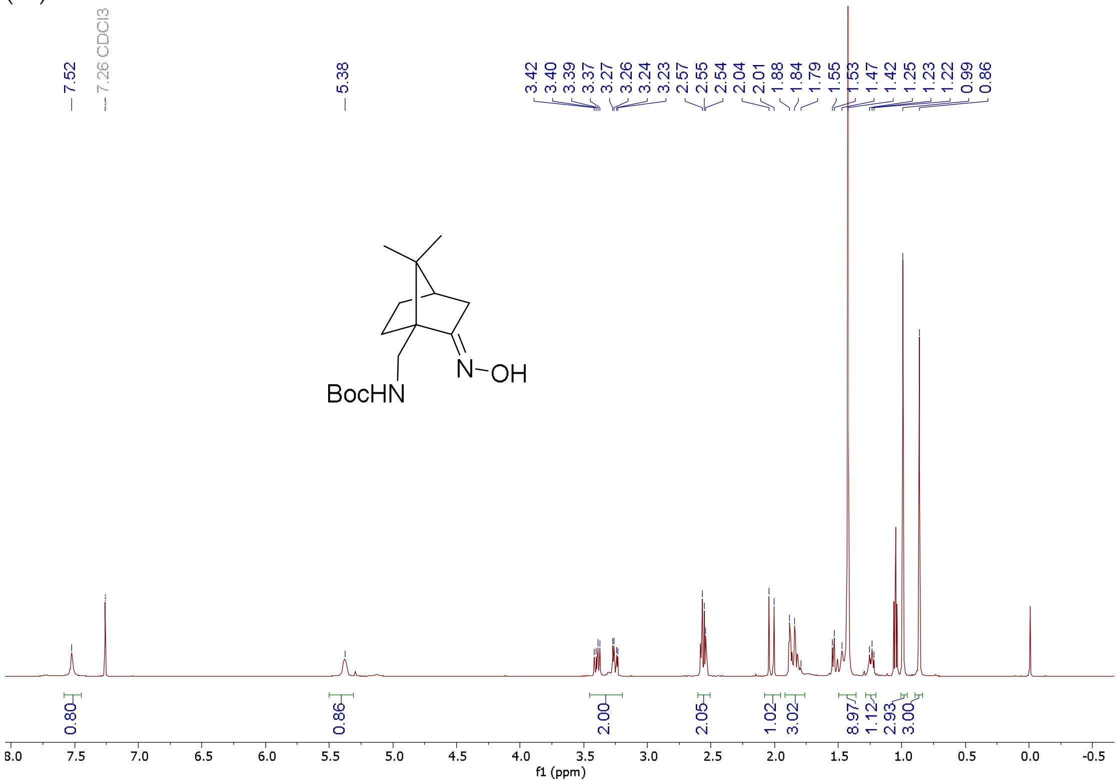

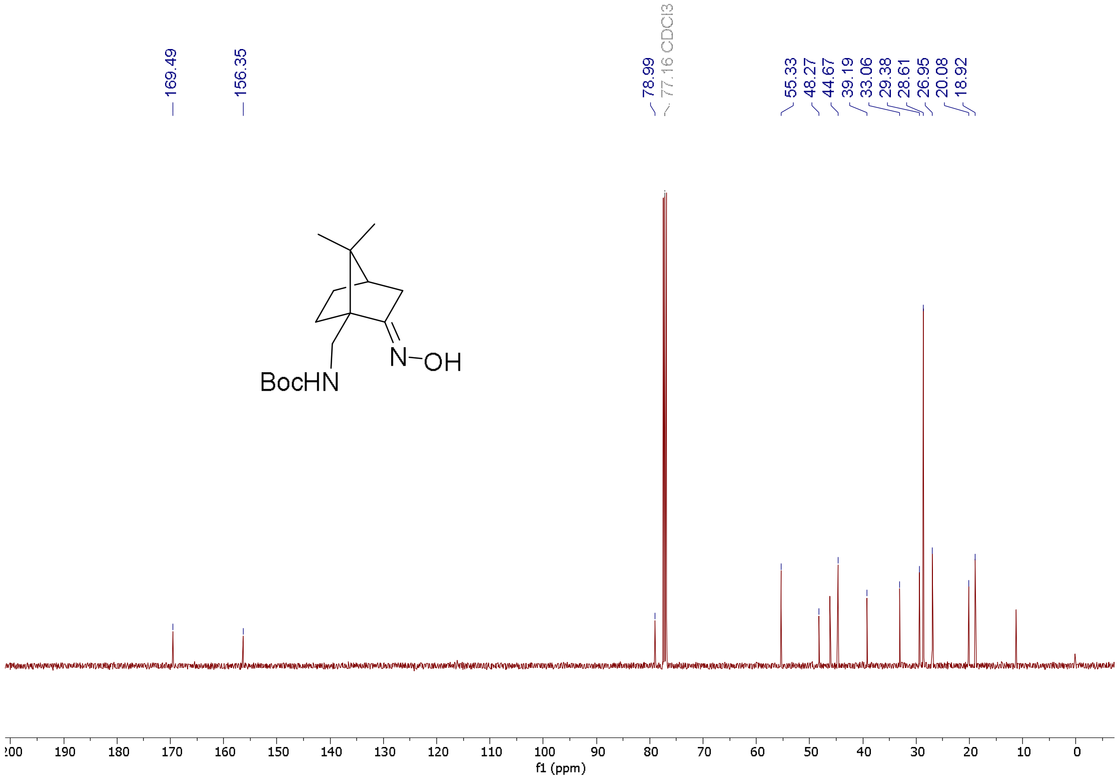

tert-Butyl (((1*R*,2*R*,4*R*)-2-amino-7,7-dimethylbicyclo[2.2.1]heptan-1-yl)methyl)carbamate (**29**)

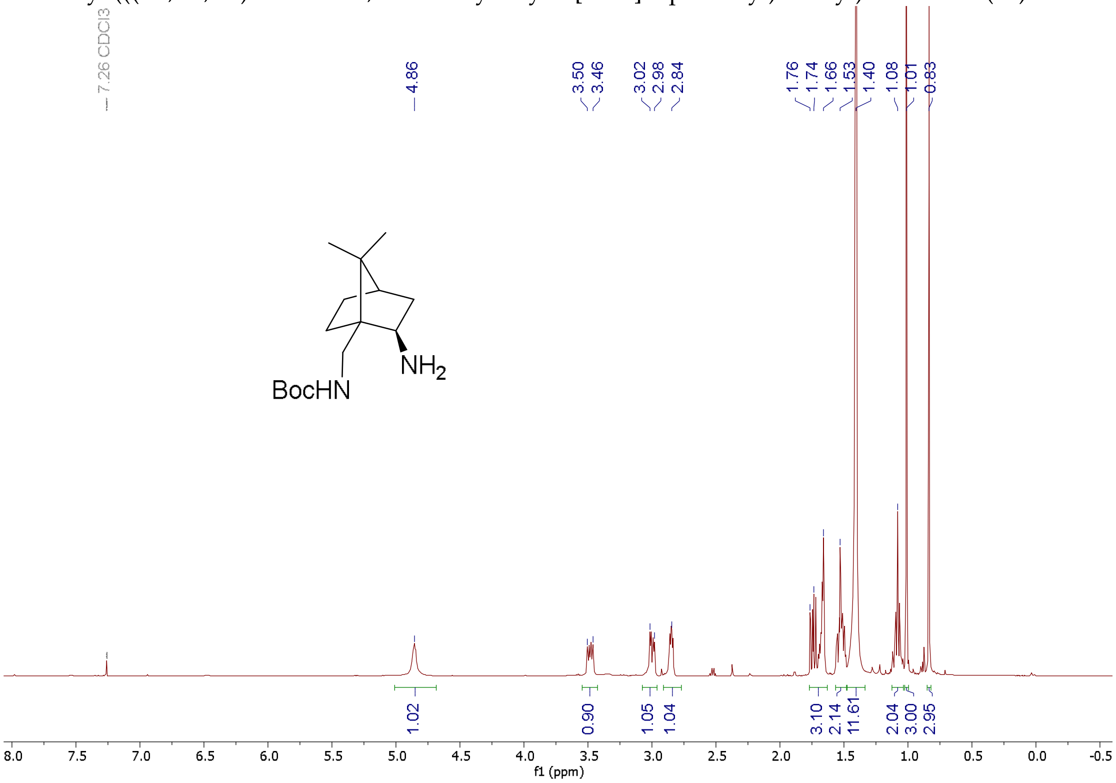

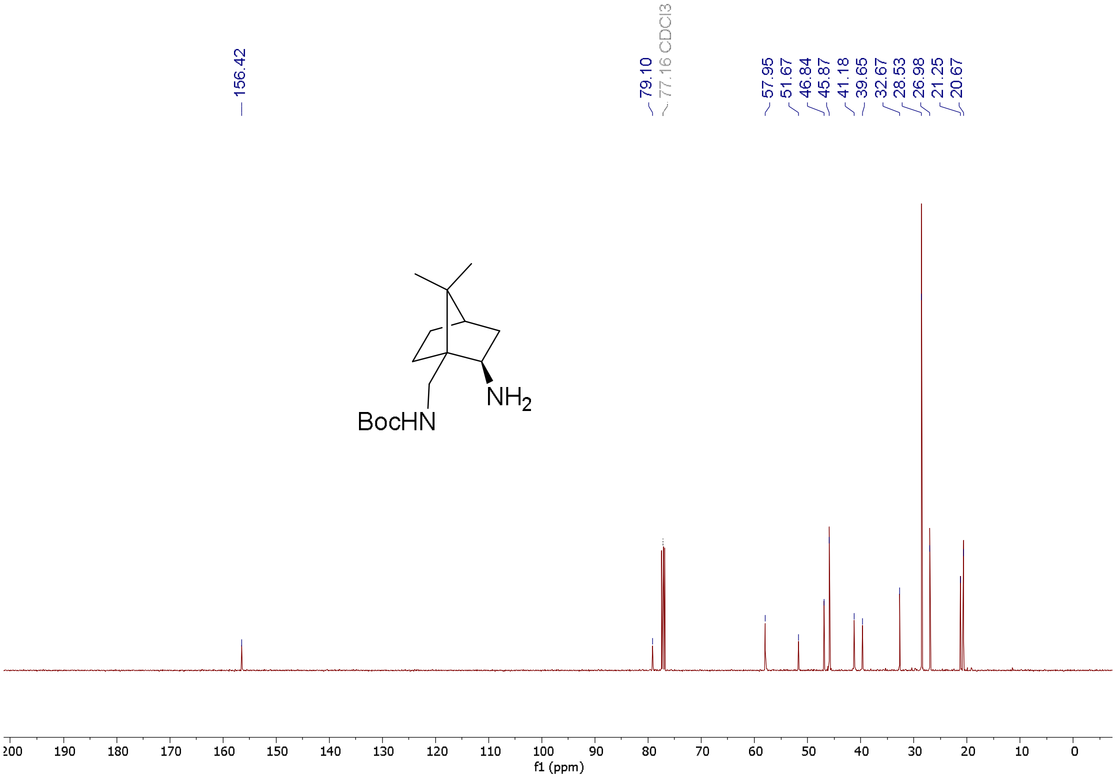

tert-Butyl (((1*R*,2*S*,4*R*)-2-amino-7,7-dimethylbicyclo[2.2.1]heptan-1-yl)methyl)carbamate (**30**)

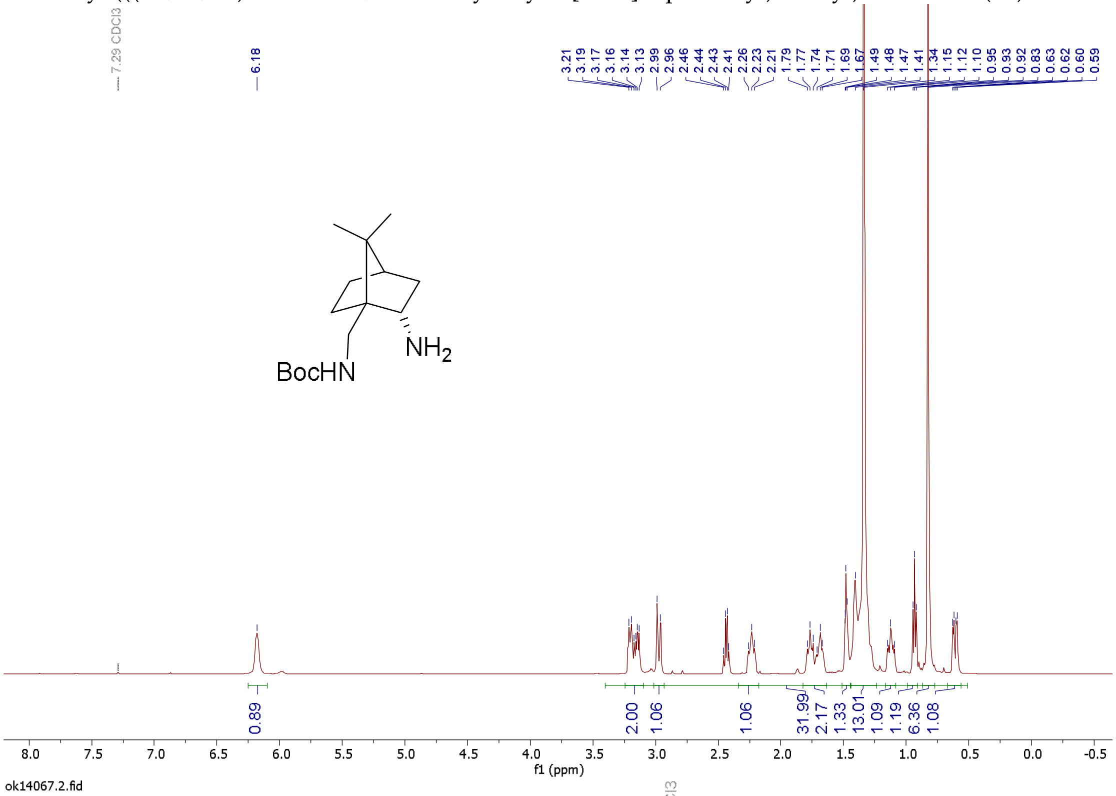

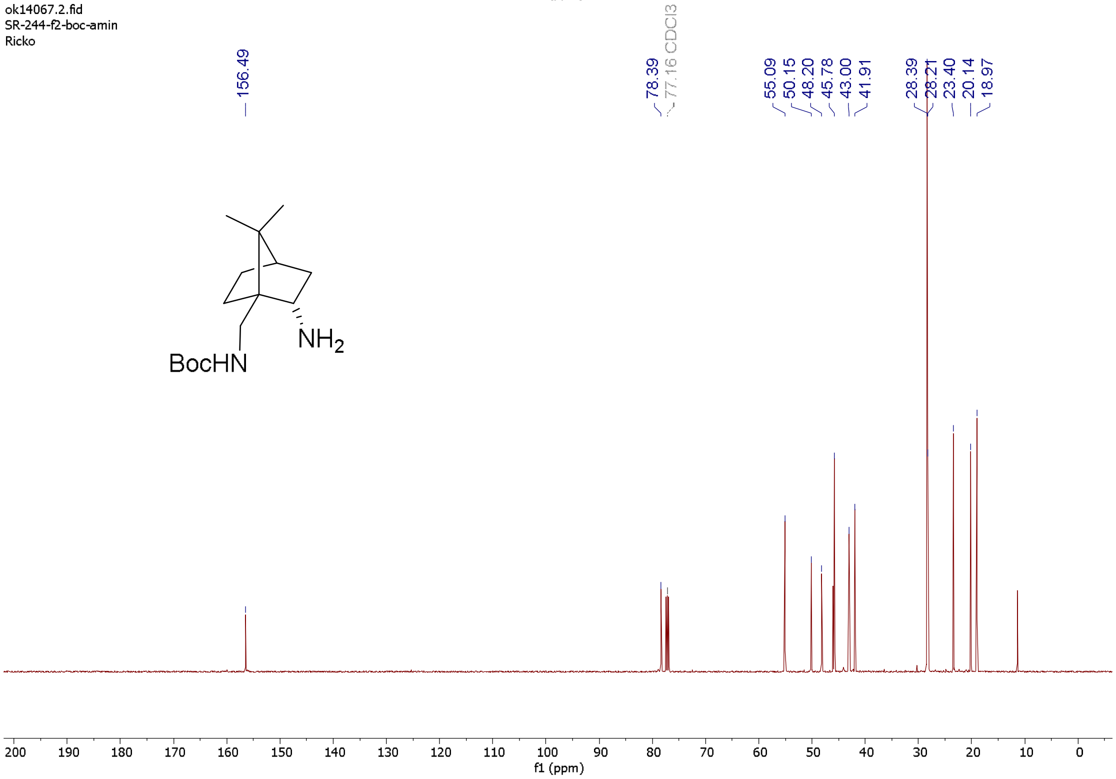

tert-Butyl (((1*R*,2*R*,4*R*)-7,7-dimethyl-2-(pyrrolidin-1-yl)bicyclo[2.2.1]heptan-1-yl)methyl)carbamate (**31**)

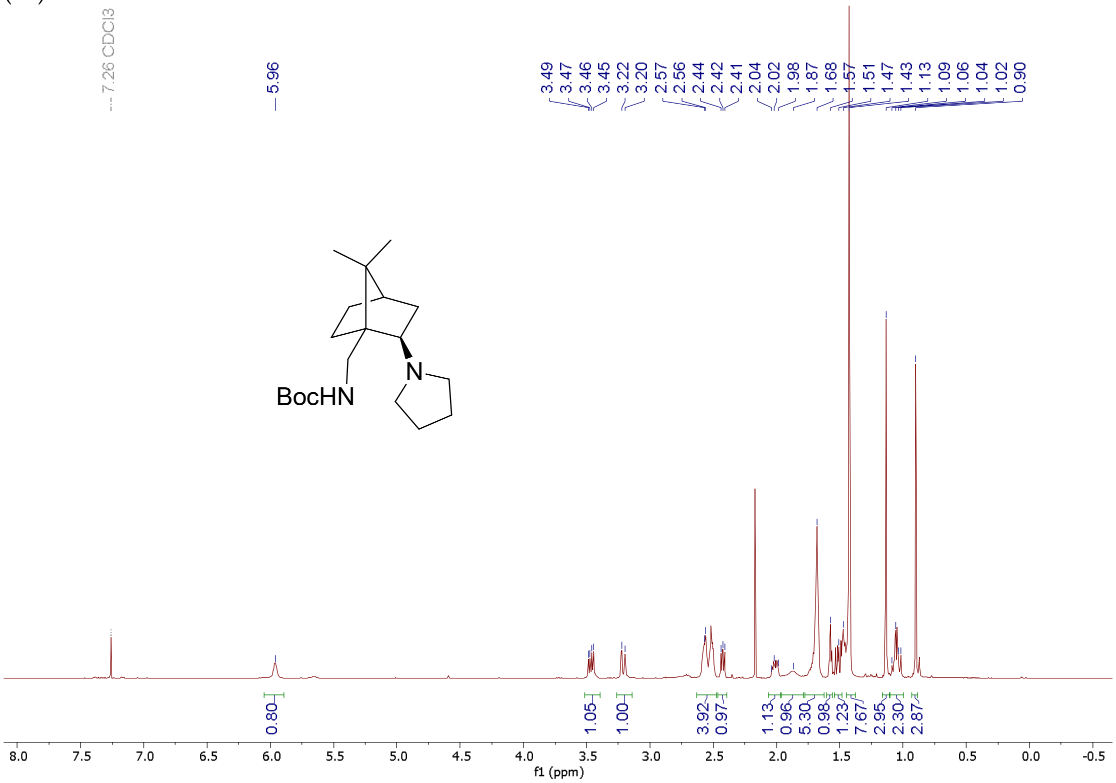

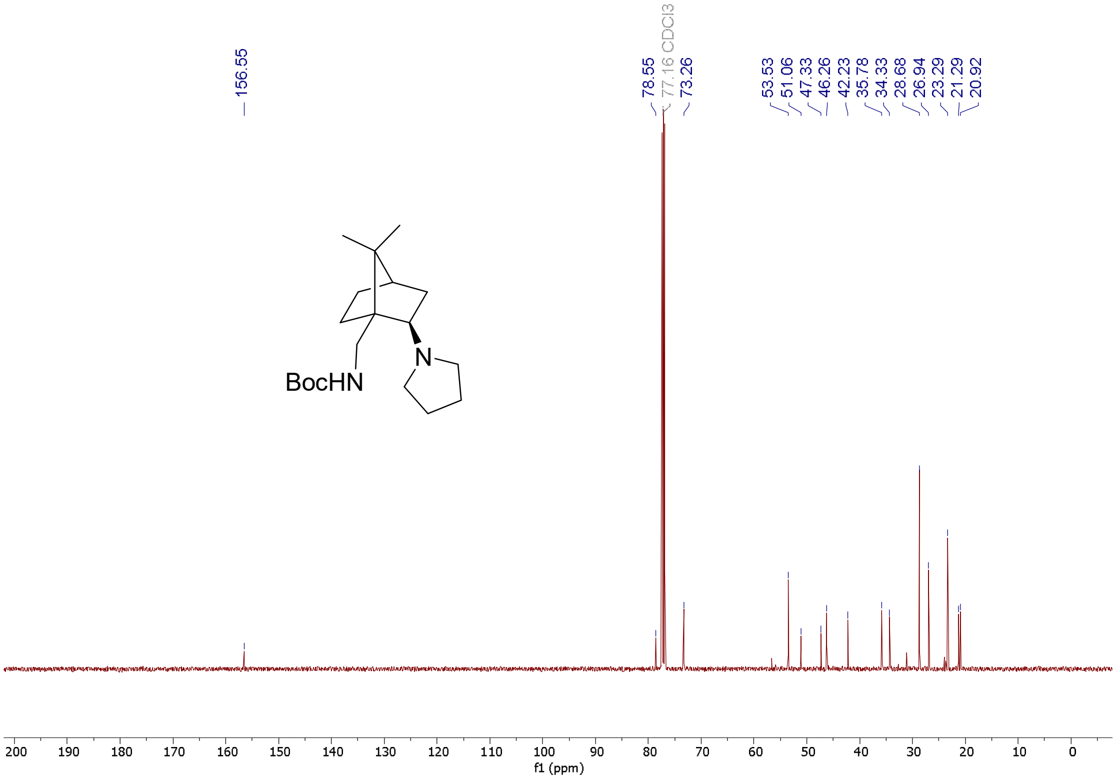

tert-Butyl (((1*R*,2*S*,4*R*)-7,7-dimethyl-2-(pyrrolidin-1-yl)bicyclo[2.2.1]heptan-1-yl)methyl)carbamate (**32**)

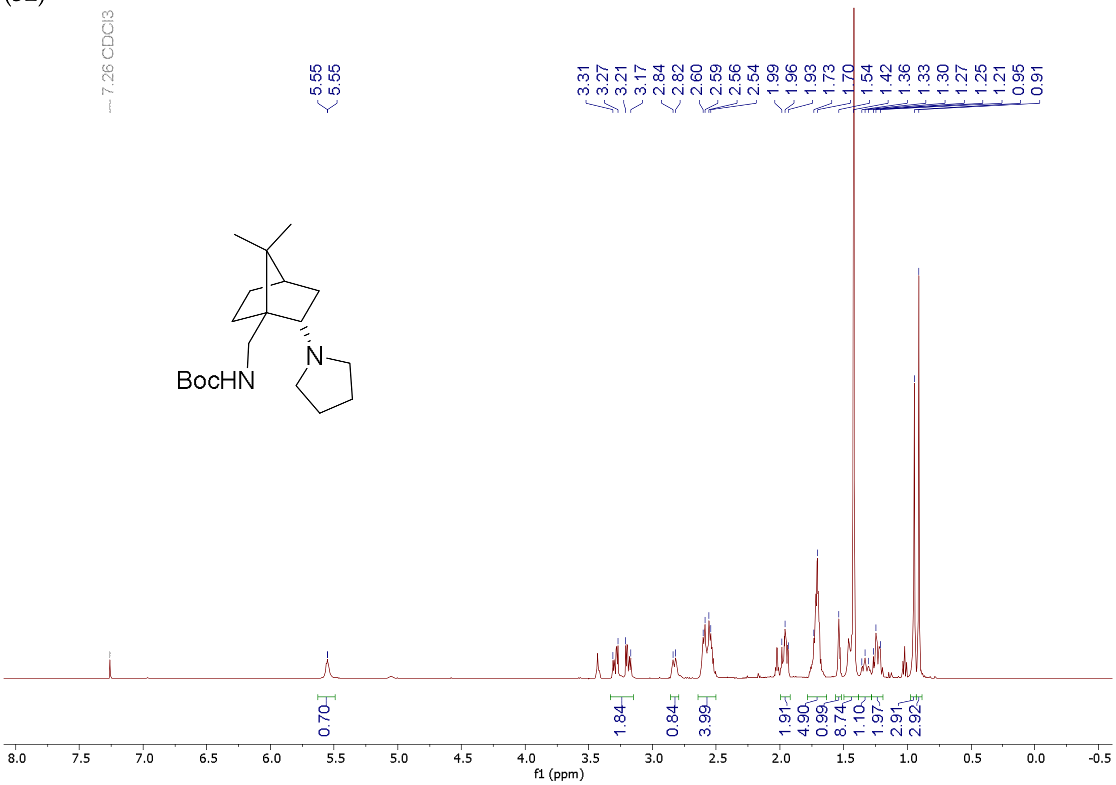

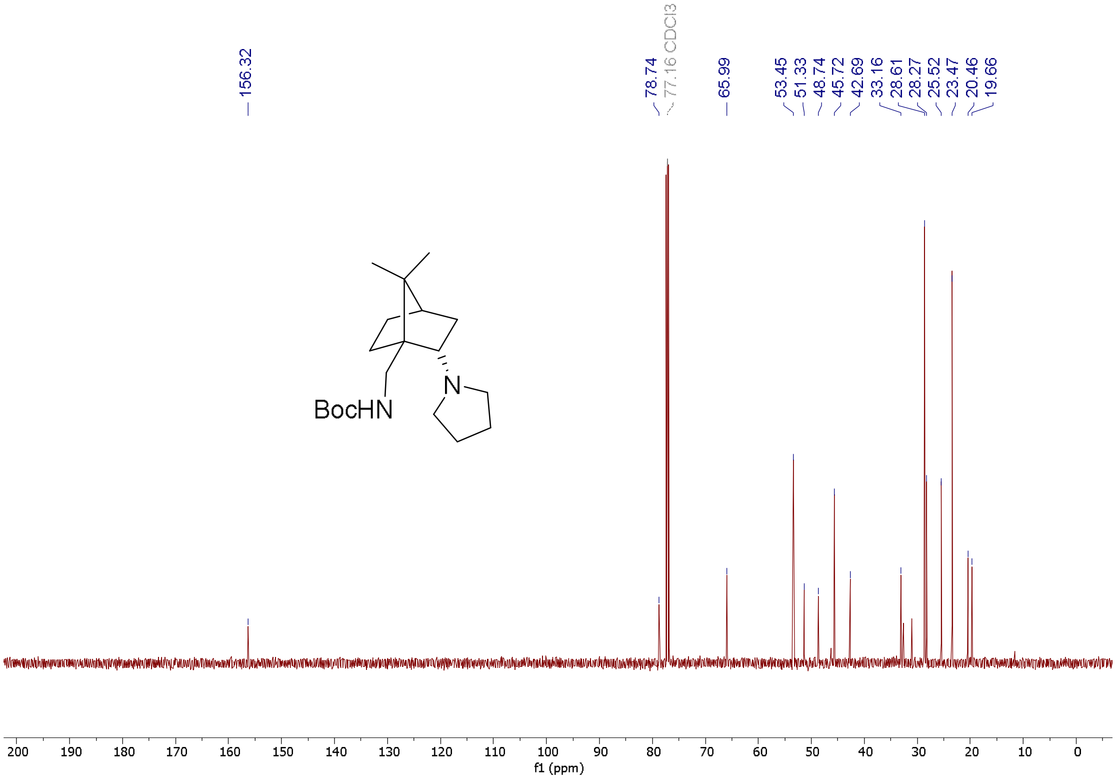

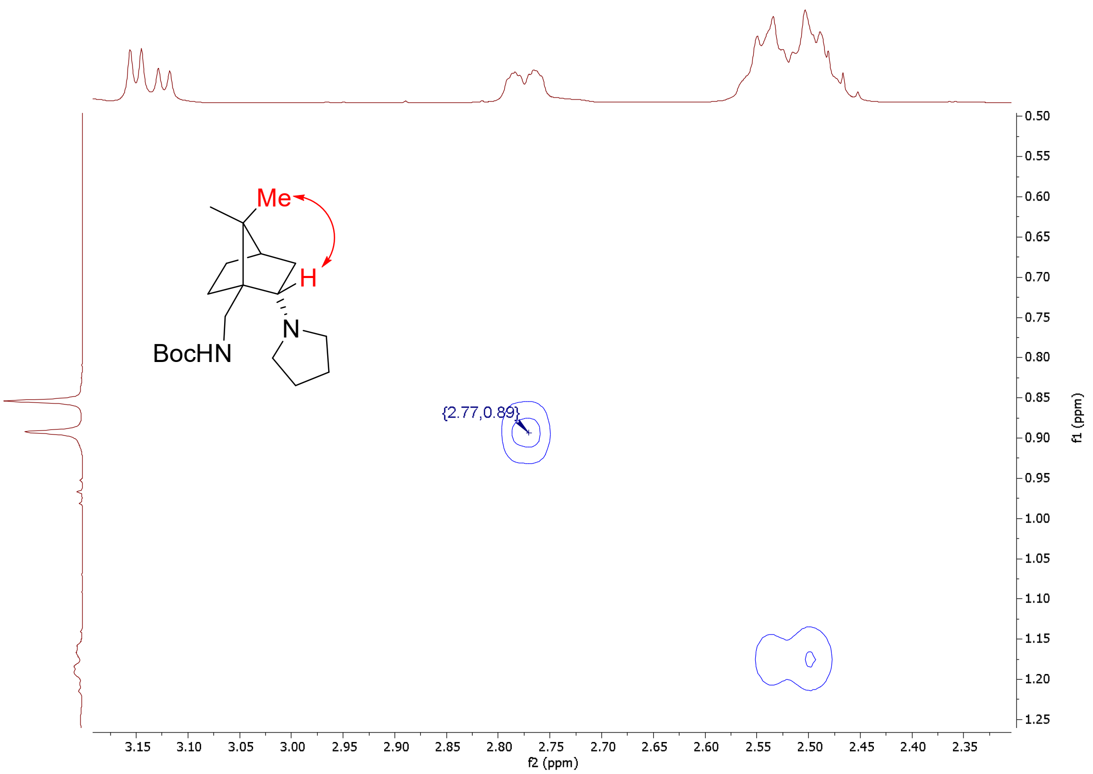

1090
1091 2D NOESY
1092
1093

((1*R*,2*R*,4*R*)-7,7-Dimethyl-2-(pyrrolidin-1-yl)bicyclo[2.2.1]heptan-1-yl)methanamine (**33**)

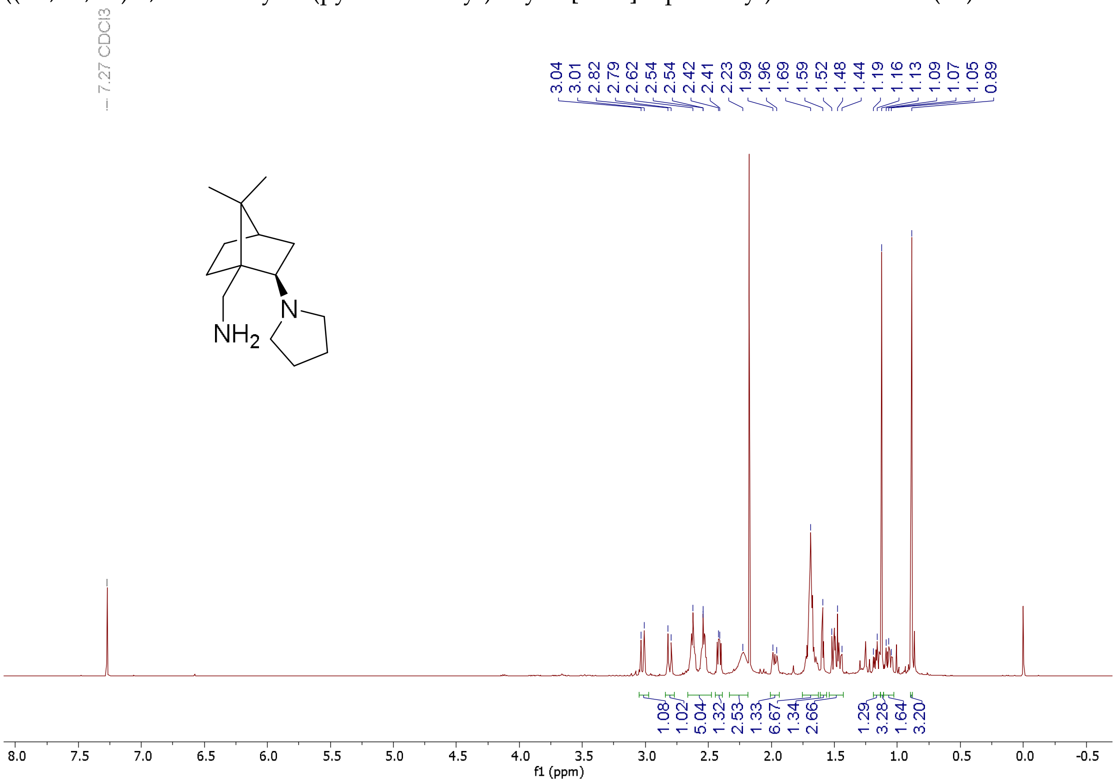

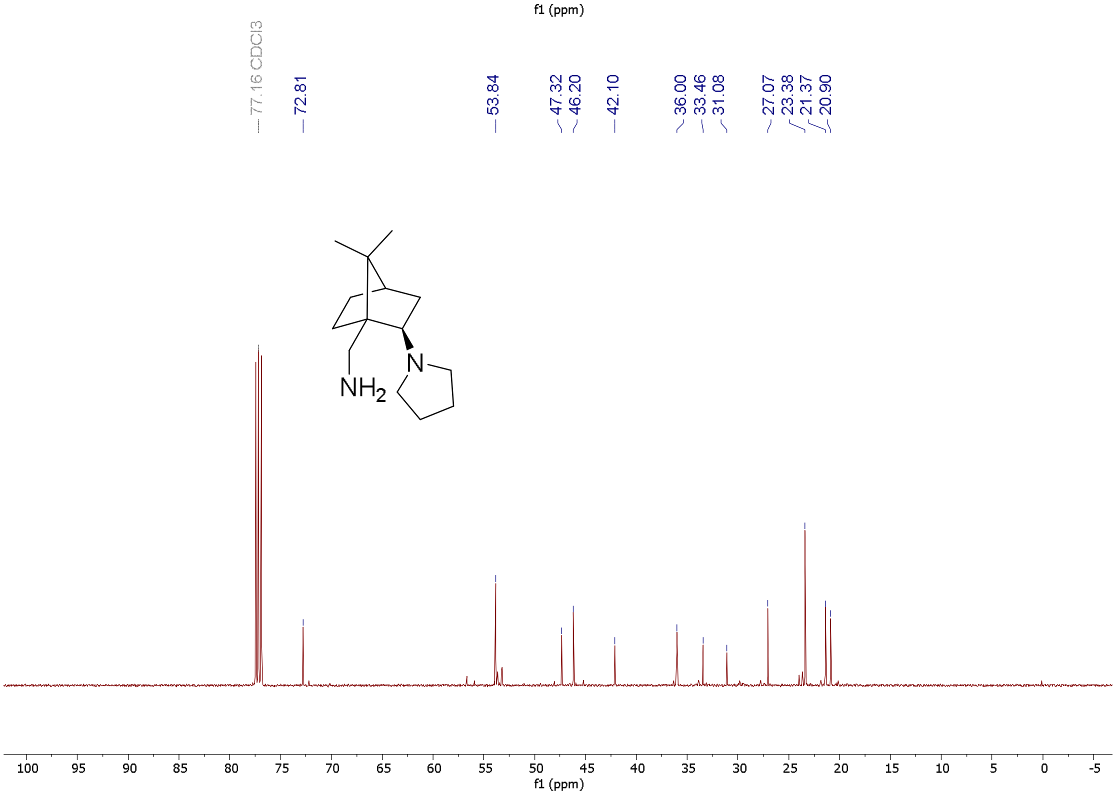

1098 ((1*R*,2*S*,4*R*)-7,7-Dimethyl-2-(pyrrolidin-1-yl)bicyclo[2.2.1]heptan-1-yl)methanamine (**34**)

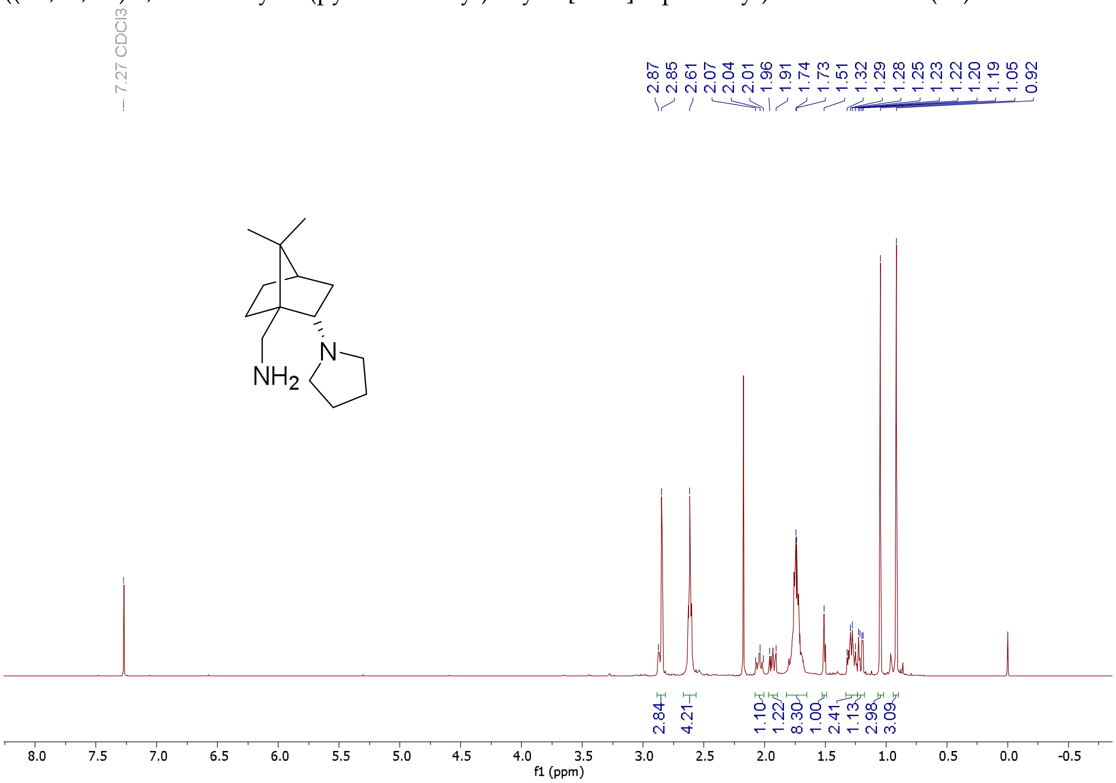

1099

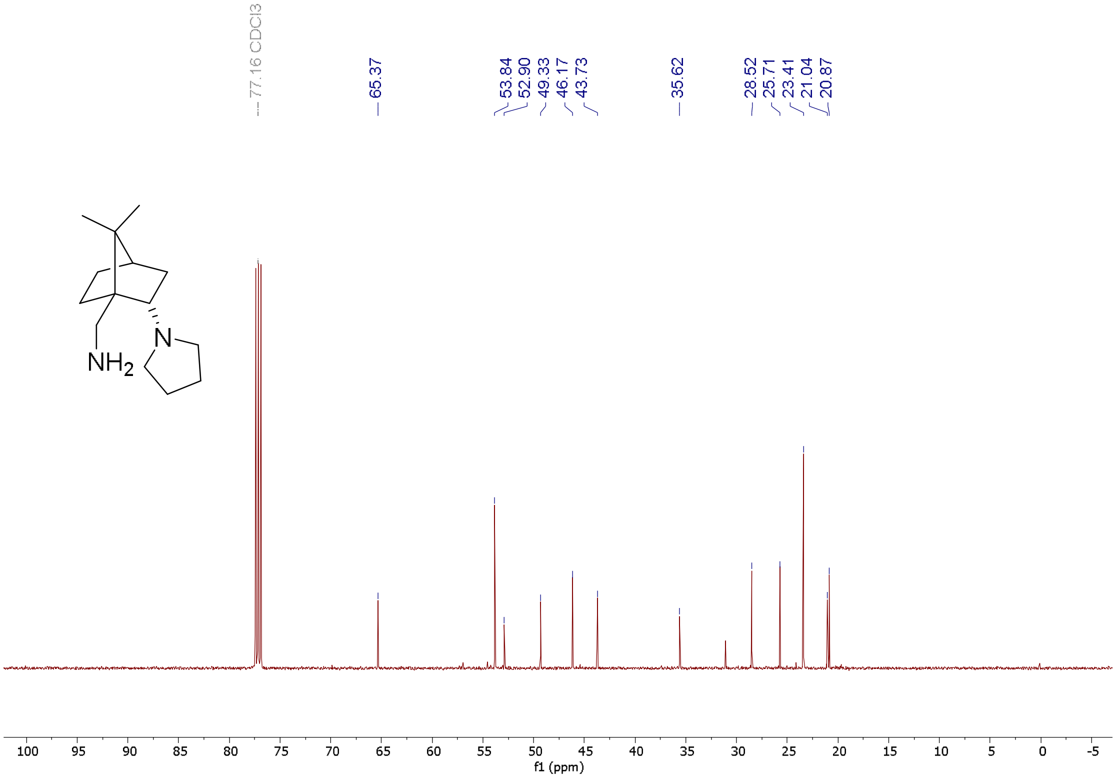

1100
1101

2-((1*S*,4*R*,*E*)-2-(Hydroxyimino)-7,7-dimethylbicyclo[2.2.1]heptan-1-yl)acetonitrile (**35**)

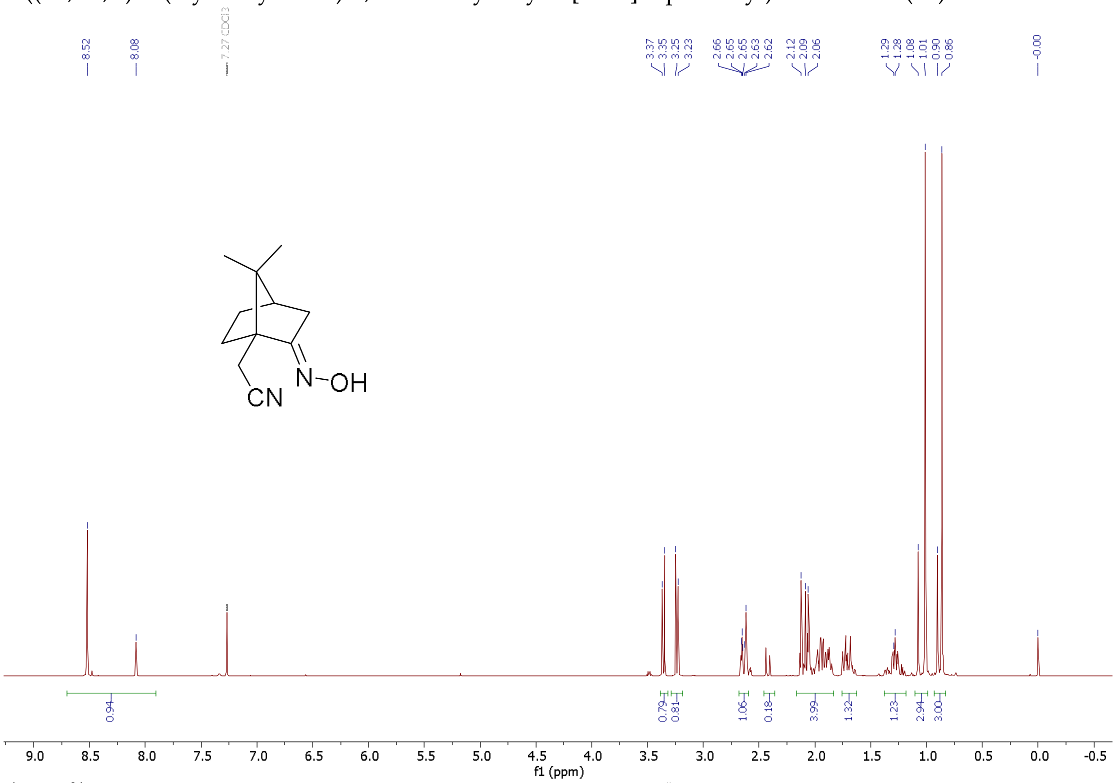

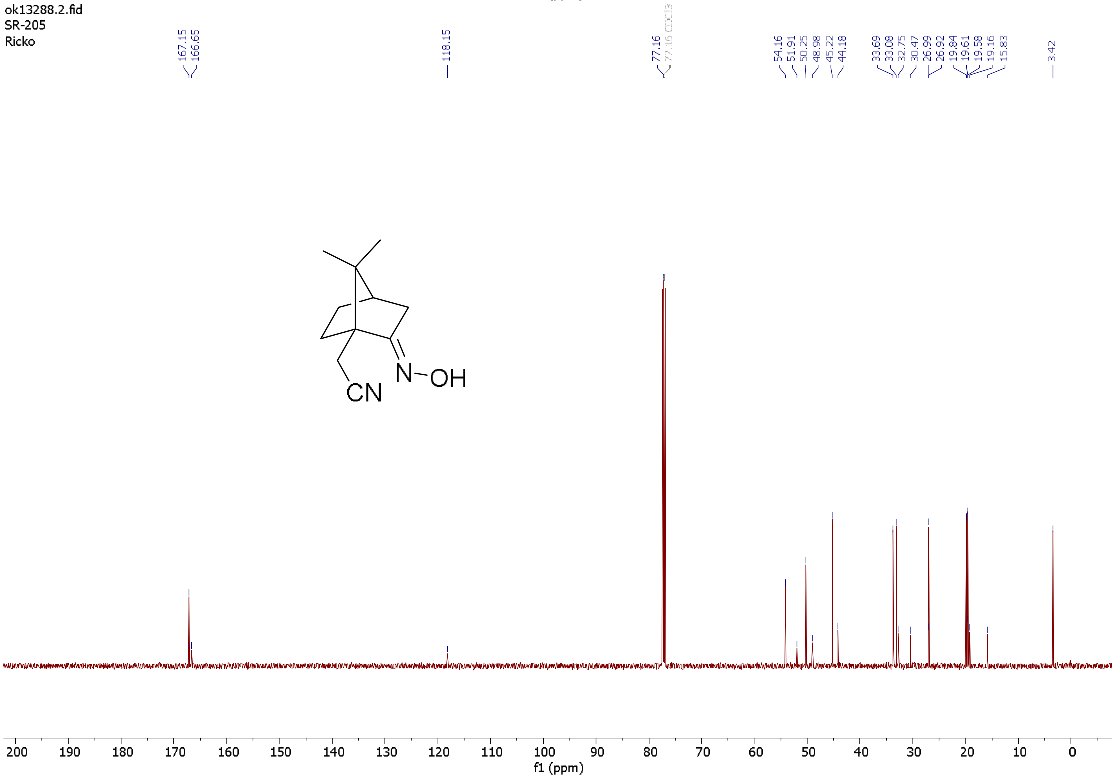

(1*S*,4*R*,*E*)-1-(2-Aminoethyl)-7,7-dimethylbicyclo[2.2.1]heptan-2-one oxime (**36**)

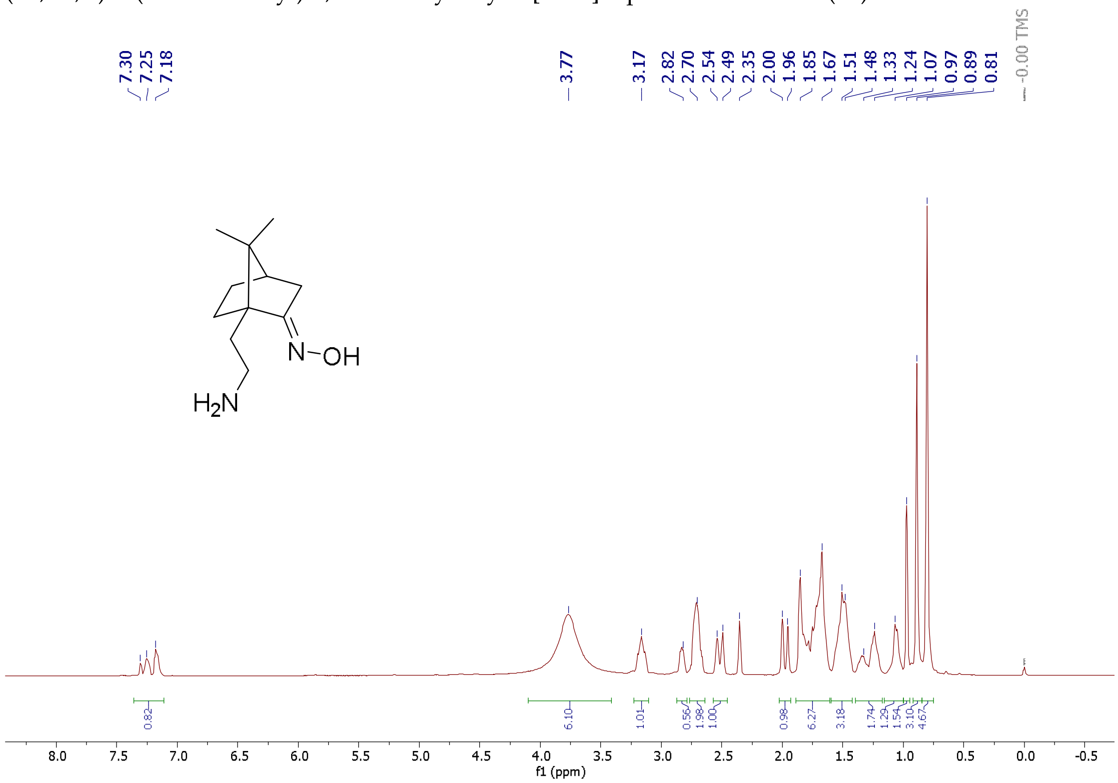

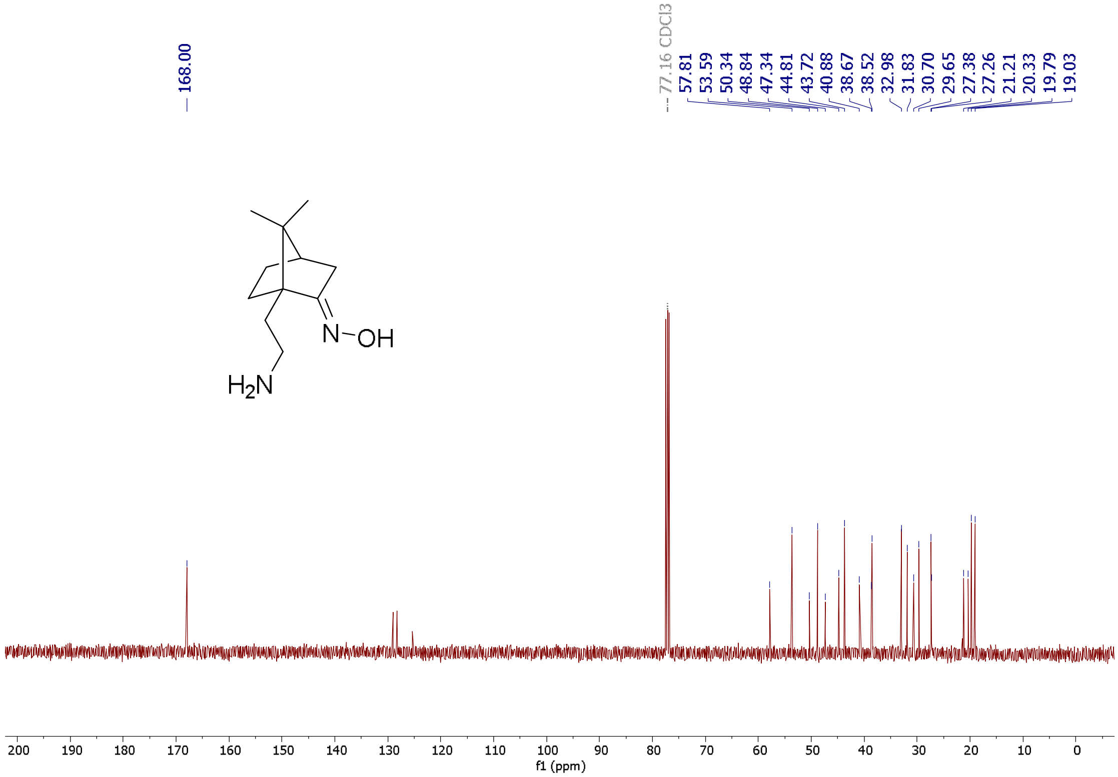

(1*S*,4*R*,*E*)-7,7-Dimethyl-1-(2-(pyrrolidin-1-yl)ethyl)bicyclo[2.2.1]heptan-2-one oxime (**37**)

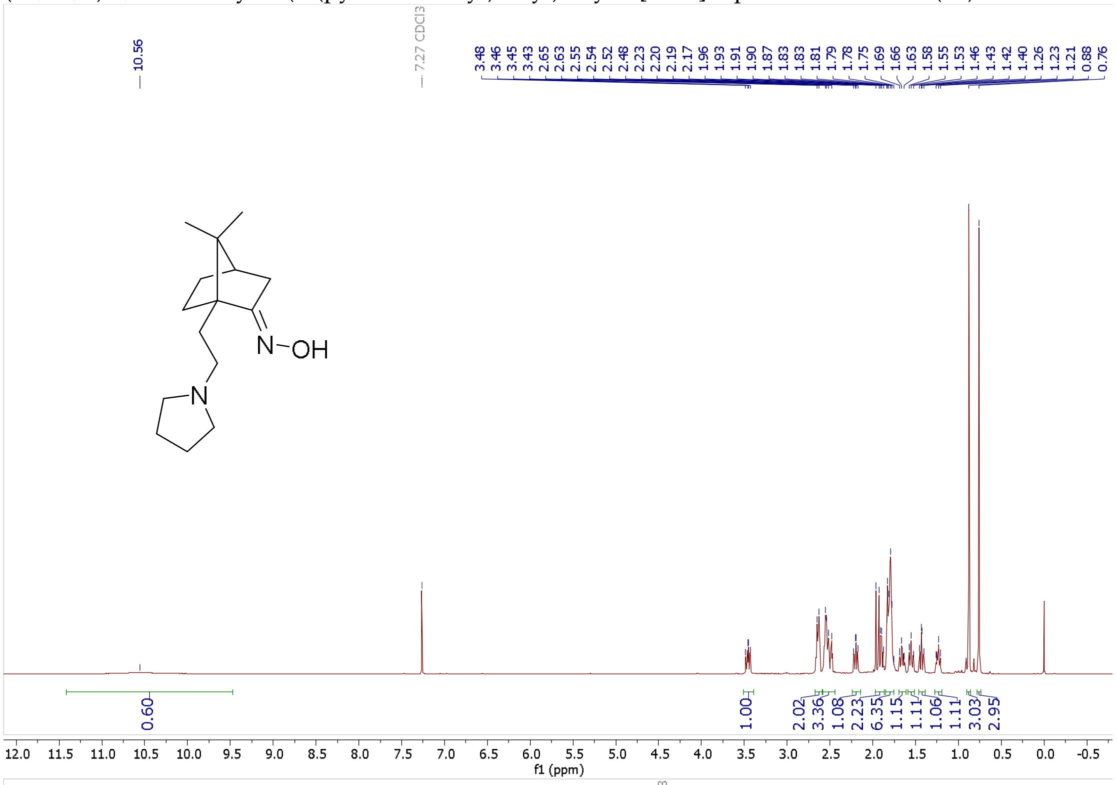

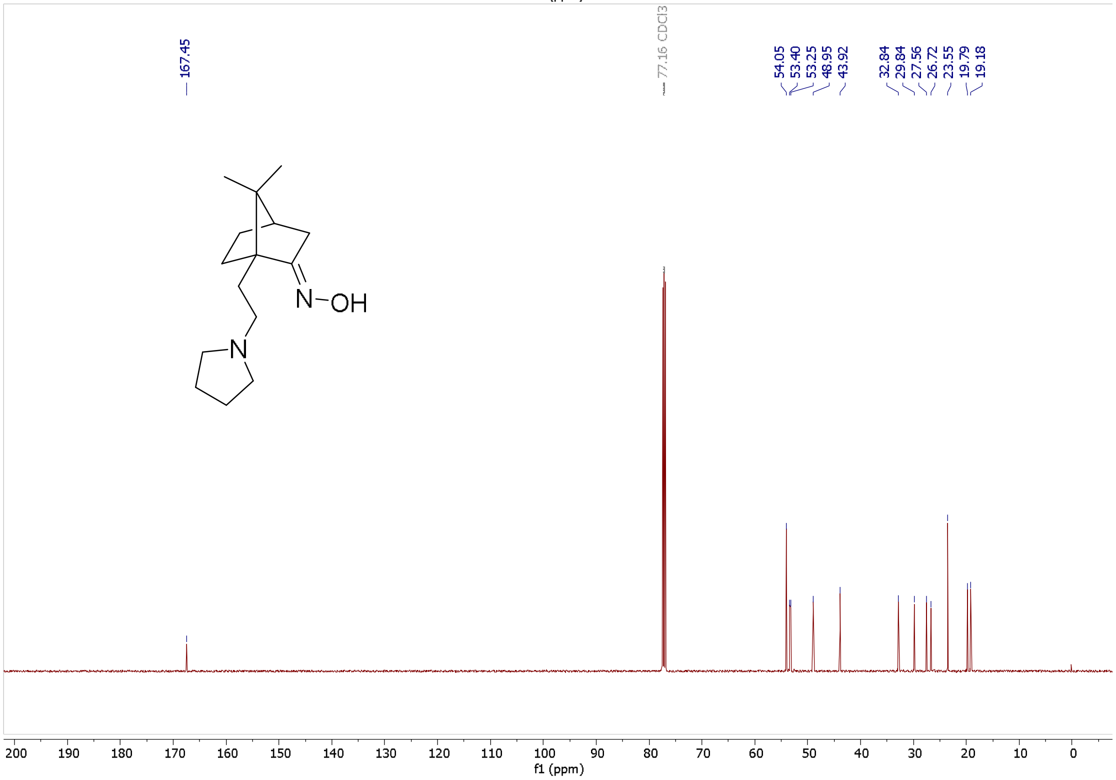

(1*S*,2*S*,4*R*)-7,7-Dimethyl-1-(2-(pyrrolidin-1-yl)ethyl)bicyclo[2.2.1]heptan-2-amine (**38**)

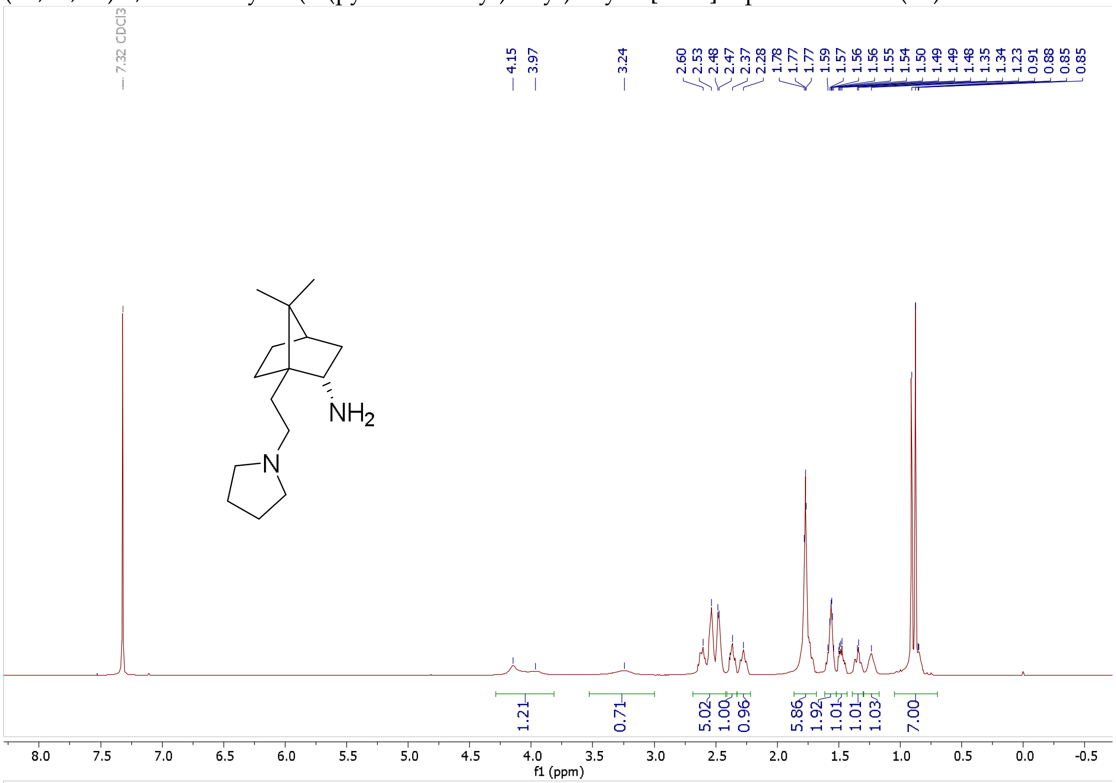

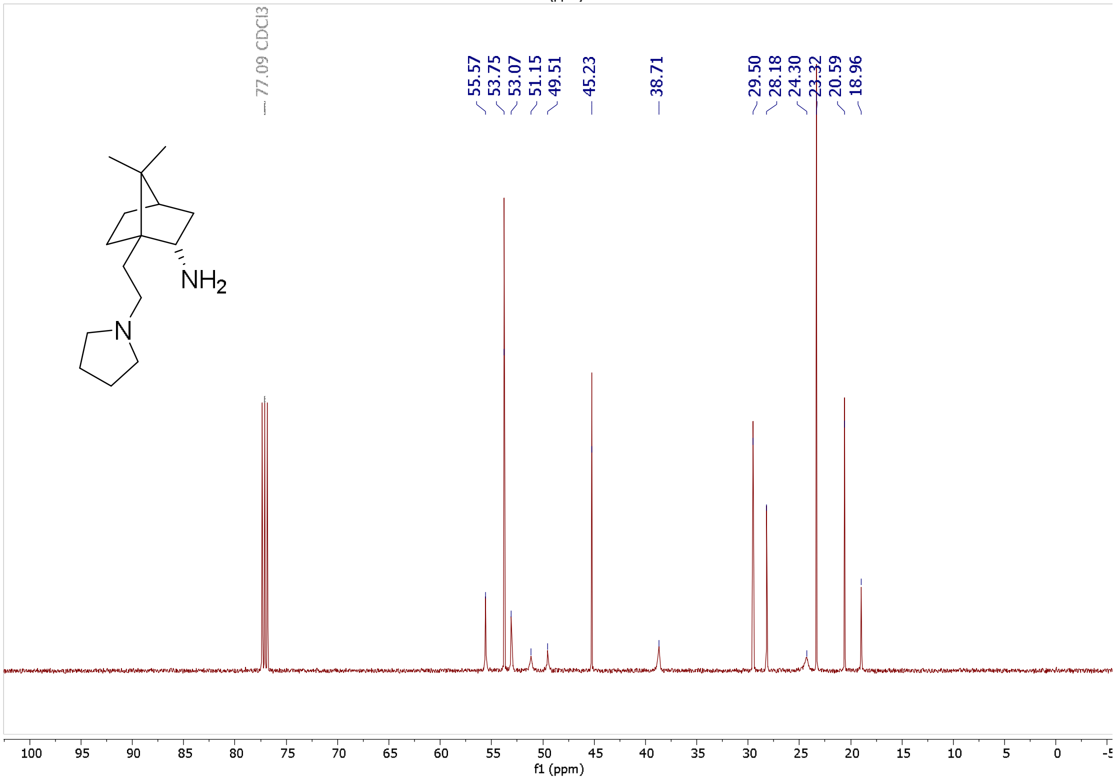

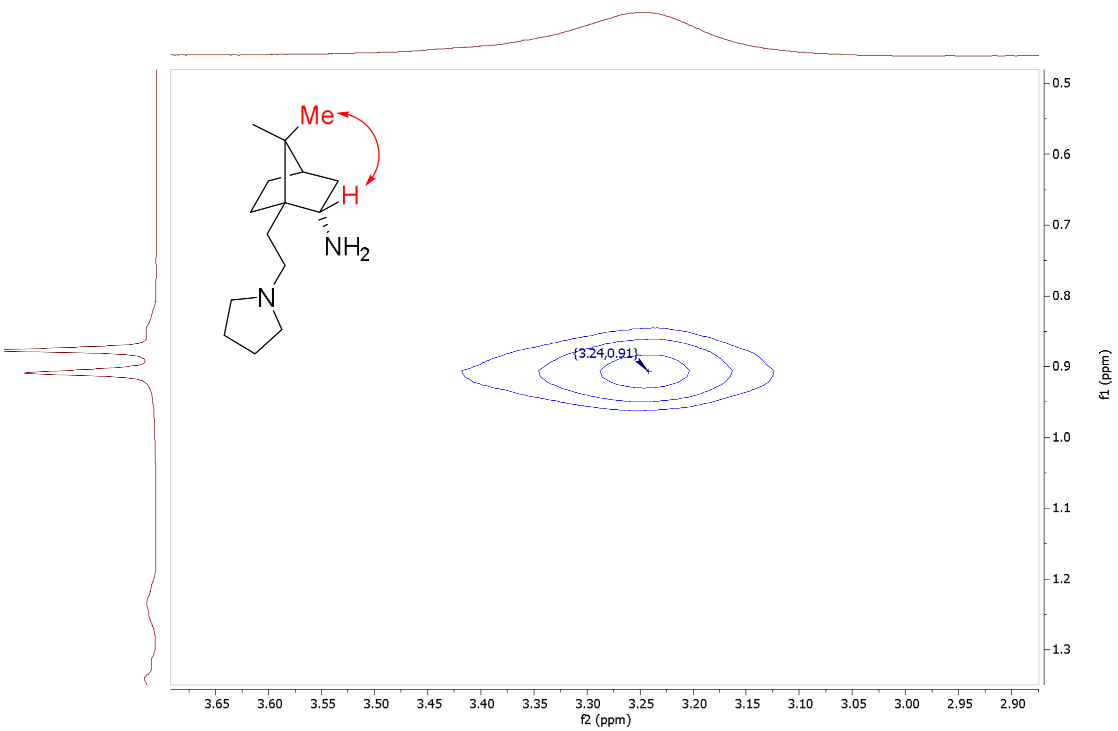
Me
H
NH2
N
{3.24,0.91}
f2 (ppm)
f1 (ppm)
3.65 3.60 3.55 3.50 3.45 3.40 3.35 3.30 3.25 3.20 3.15 3.10 3.05 3.00 2.95 2.90
0.5 0.6 0.7 0.8 0.9 1.0 1.1 1.2 1.3

2D NOESY

(1*S*,2*R*,4*R*)-7,7-Dimethyl-1-(2-(pyrrolidin-1-yl)ethyl)bicyclo[2.2.1]heptan-2-amine (**39**)

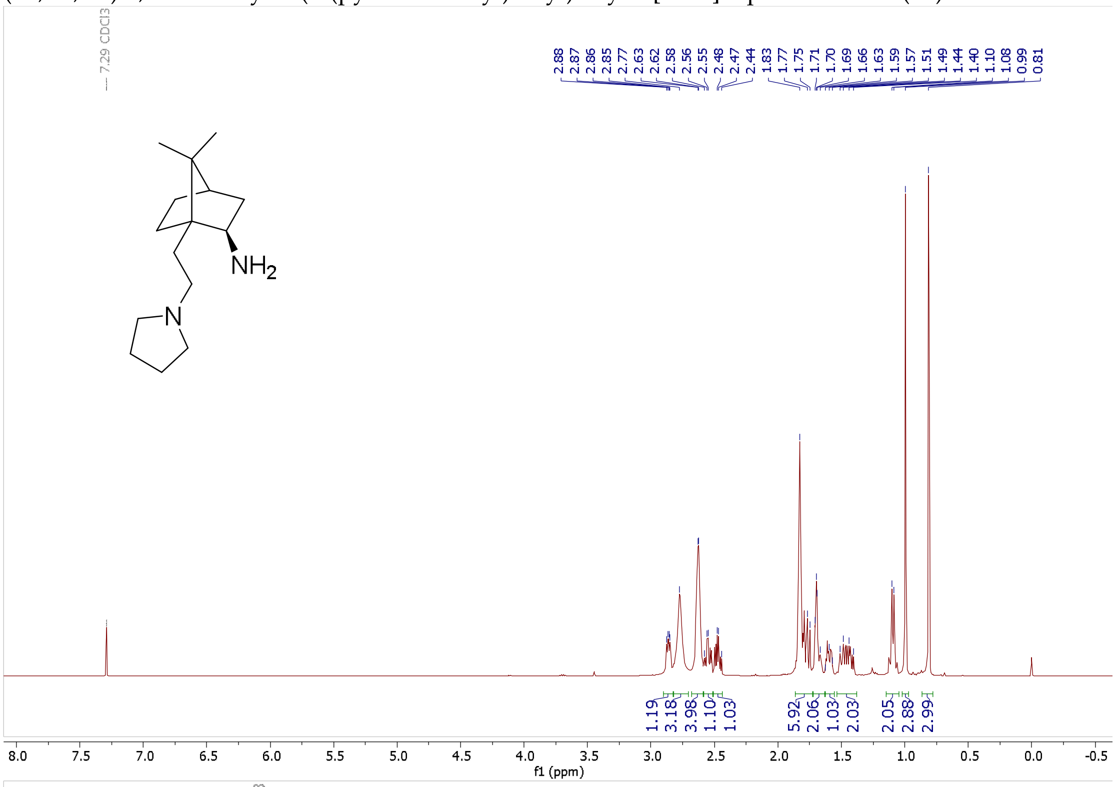

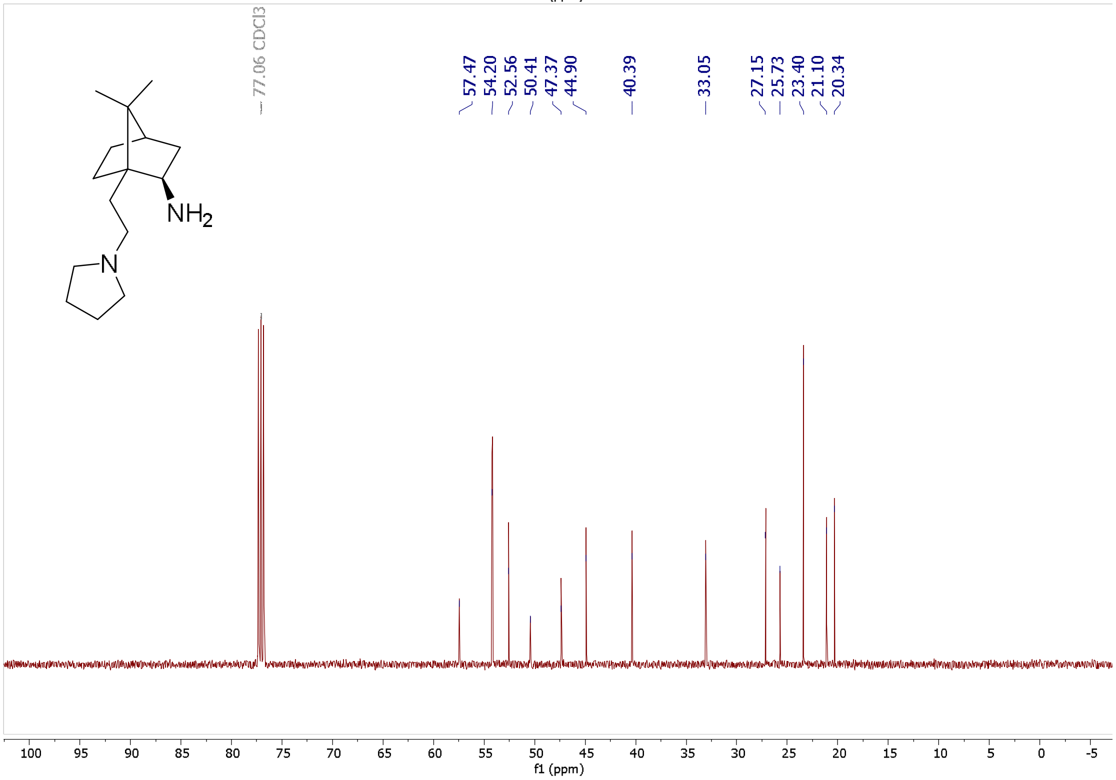

tert-Butyl (2-((1*S*,4*R*,*E*)-2-(hydroxyimino)-7,7-dimethylbicyclo-[2.2.1]heptan-1-yl)ethyl)carbamate (**40**)

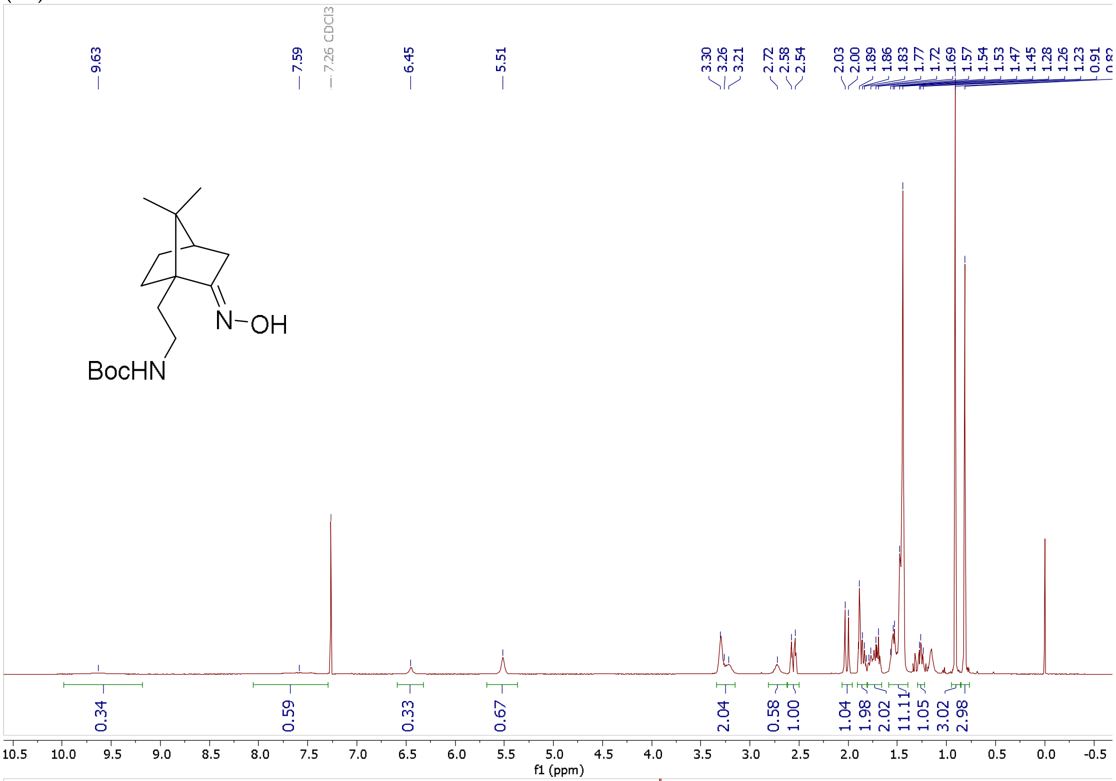

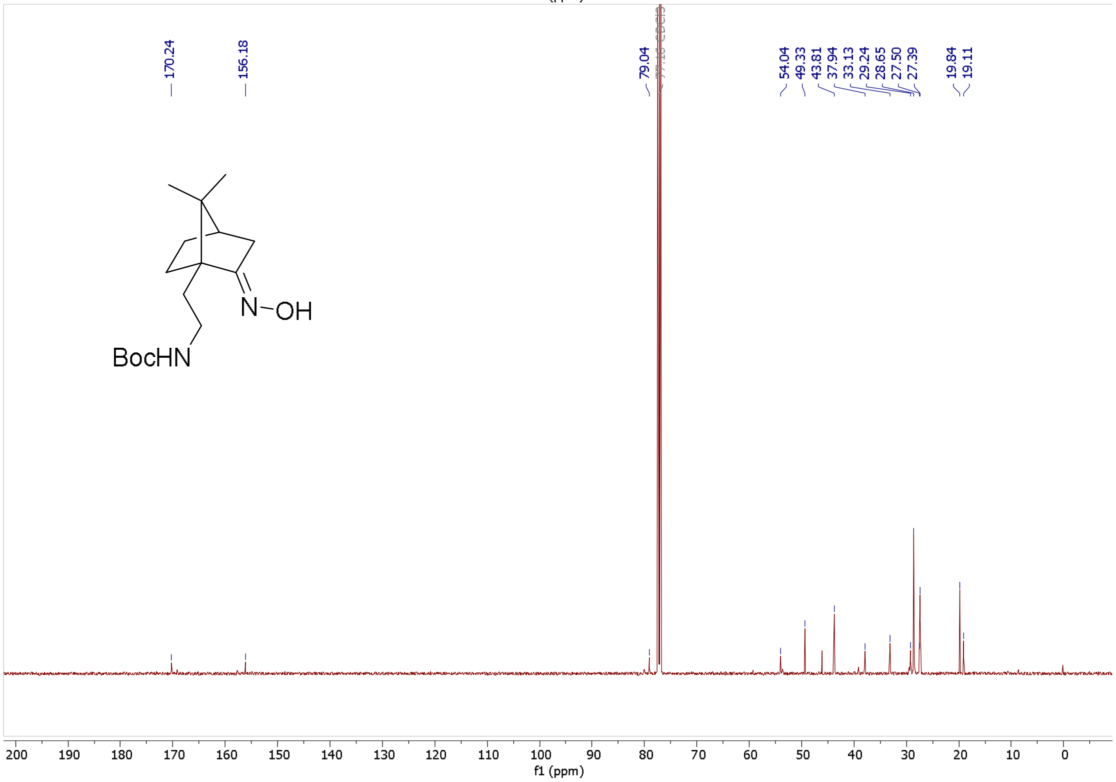

tert-Butyl (2-((1*S*,4*R*,*E*)-2-(((tert-butoxycarbonyl)oxy)imino)-7,7-dimethylbicyclo[2.2.1]heptan-1-yl)ethyl)carbamate (**41**)

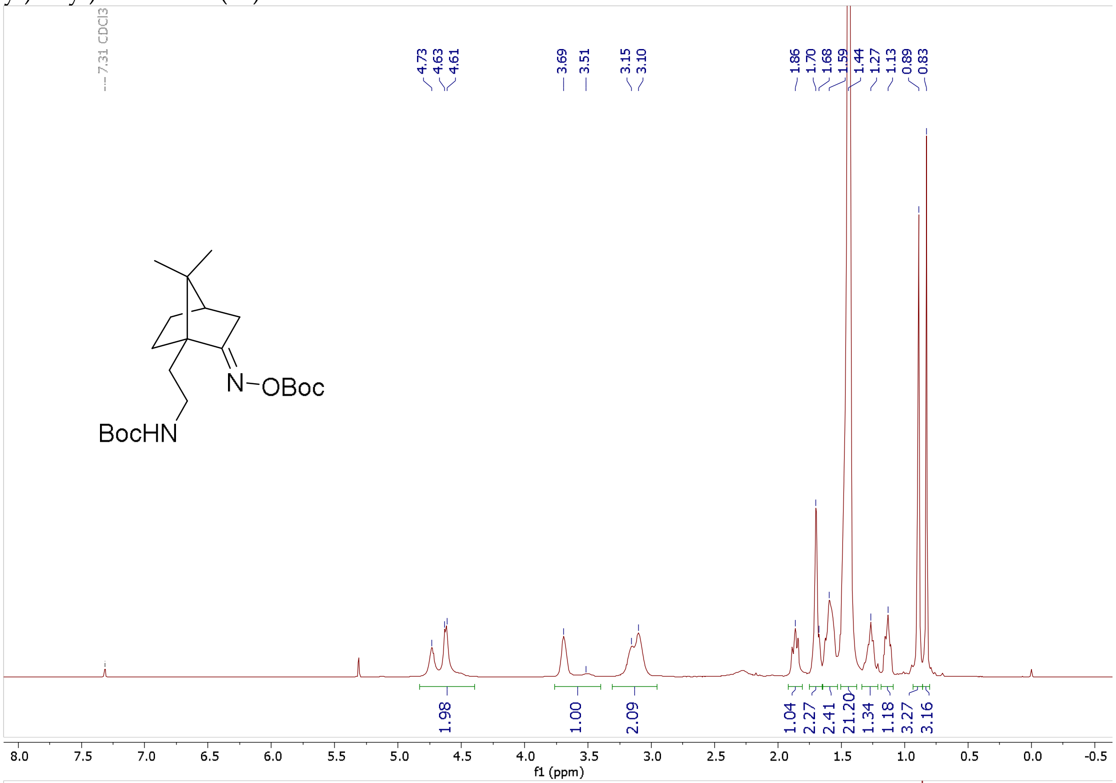

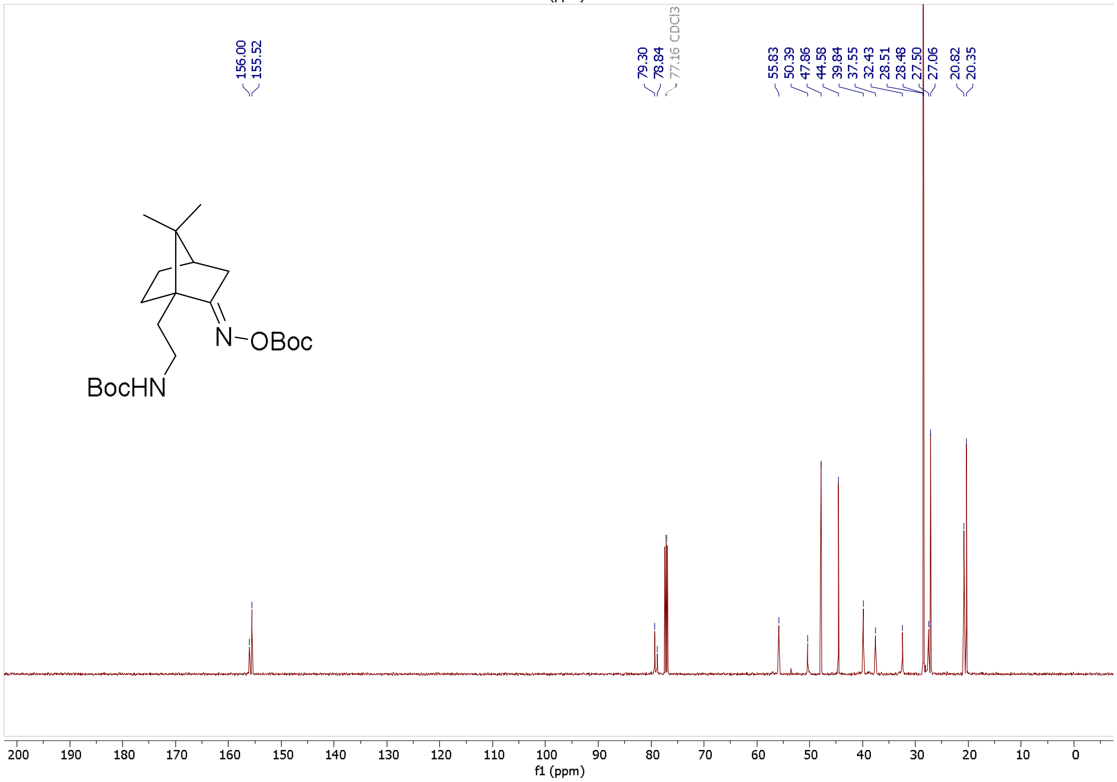

tert-Butyl (2-((1*S*,2*R*,4*R*)-2-amino-7,7-dimethylbicyclo[2.2.1]heptan-1-yl)ethyl)carbamate (**42**)

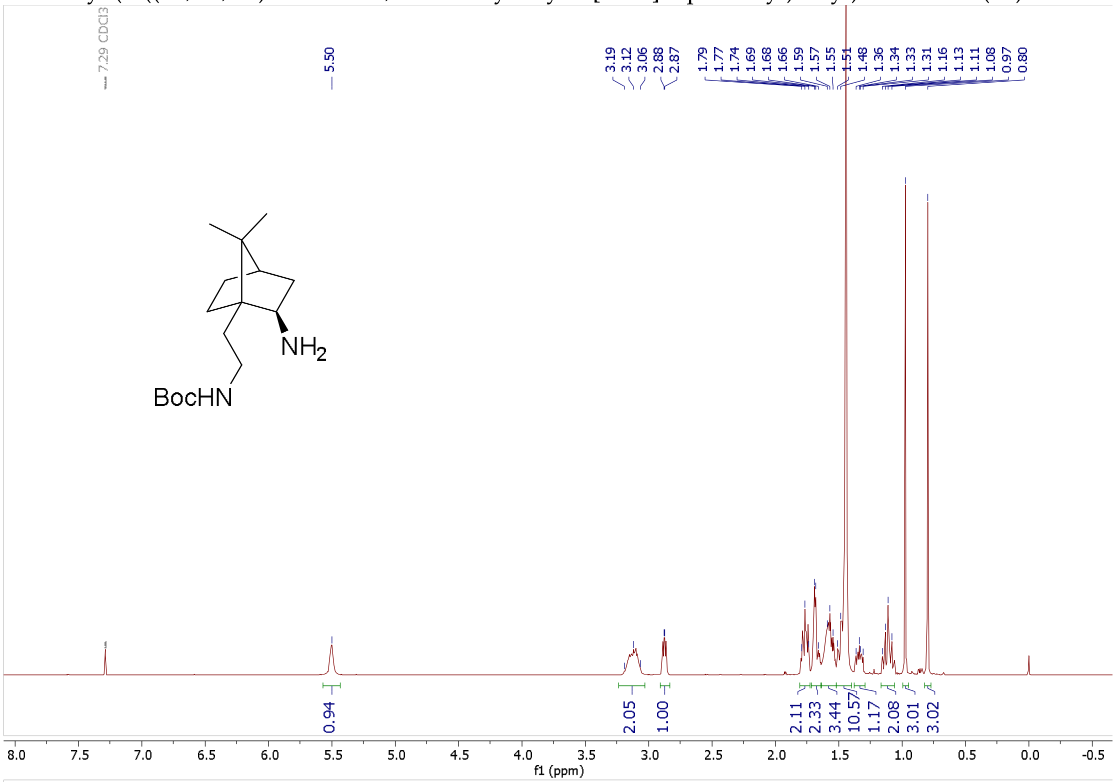

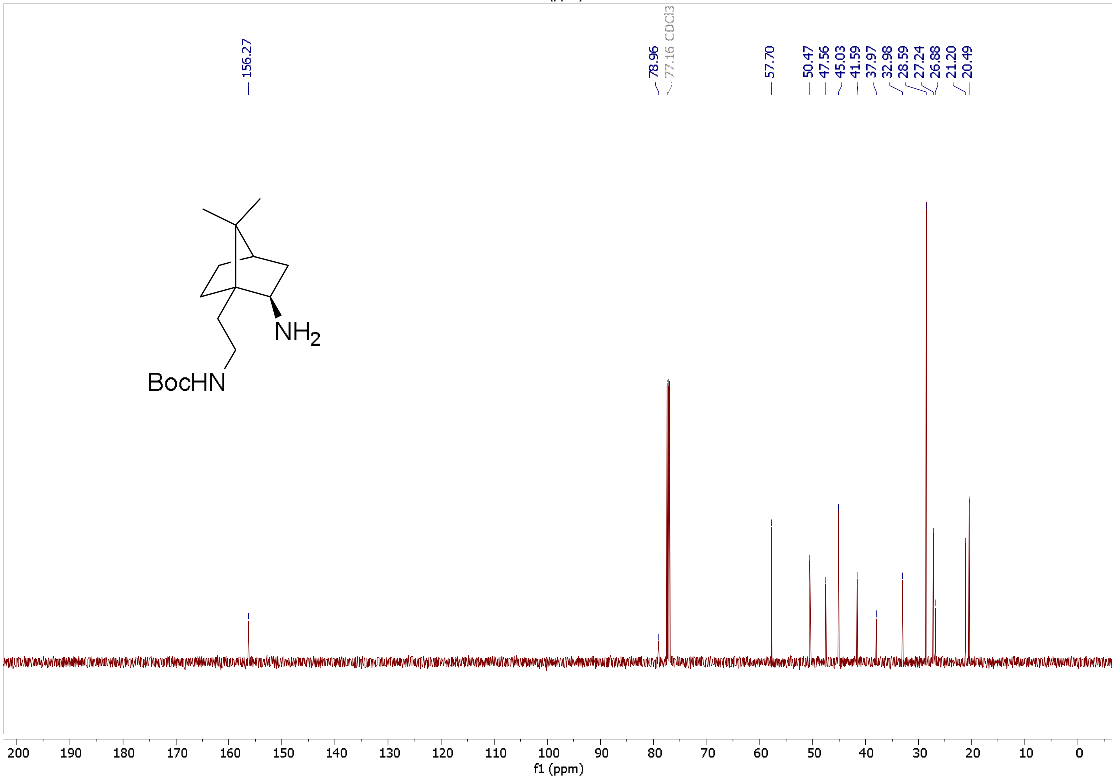

tert-Butyl (2-((1*S*,2*S*,4*R*)-2-amino-7,7-dimethylbicyclo[2.2.1]heptan-1-yl)ethyl)carbamate (**43**)

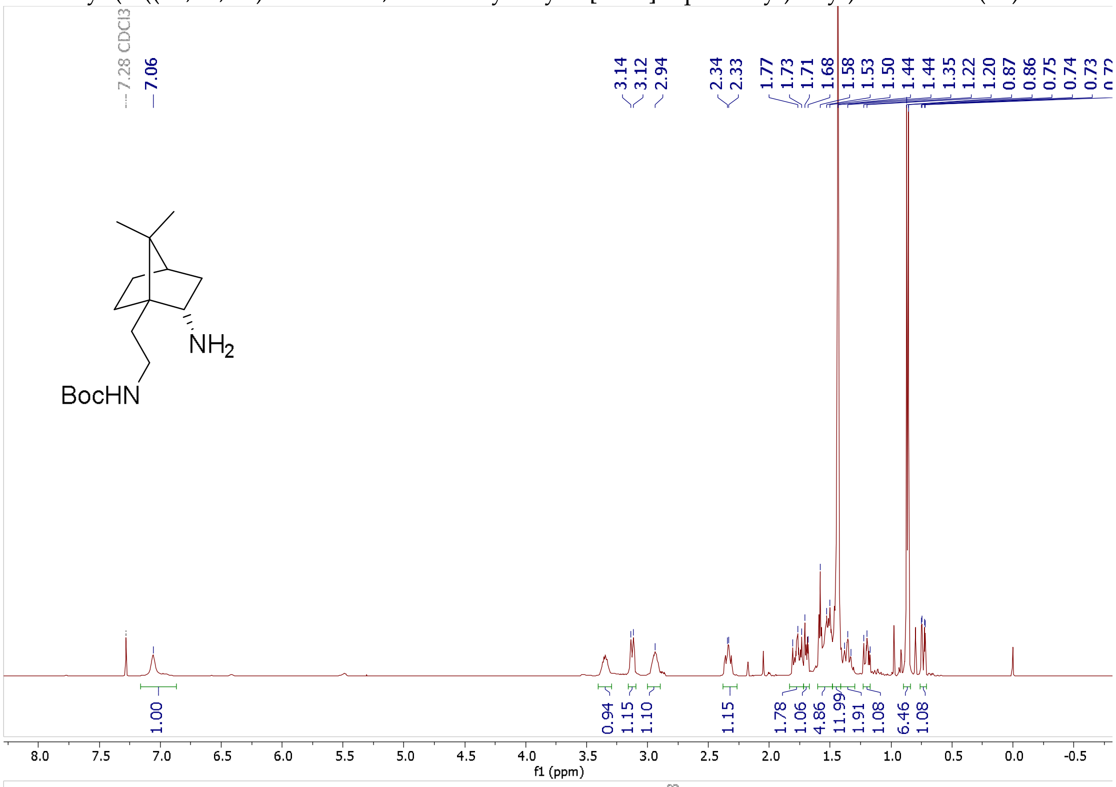

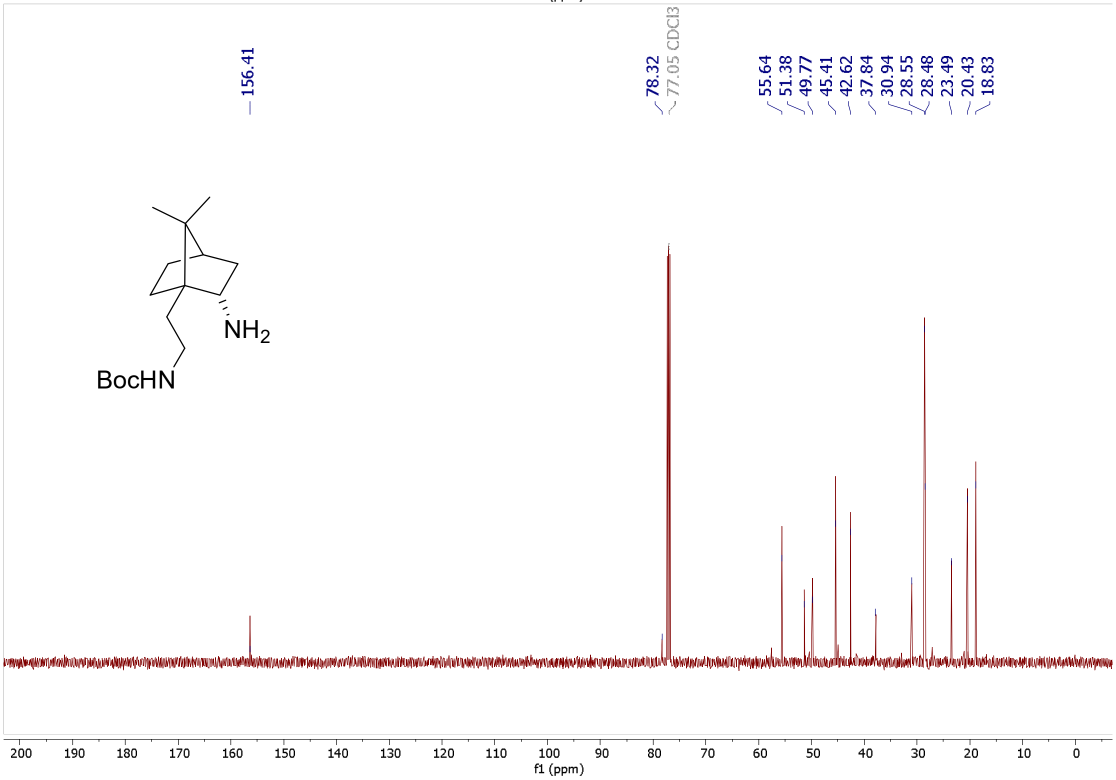

tert-Butyl (2-((1S,2S,4R)-7,7-dimethyl-2-(pyrrolidin-1-yl)bicyclo[2.2.1]heptan-1-yl)ethyl)carbamate (**45**)

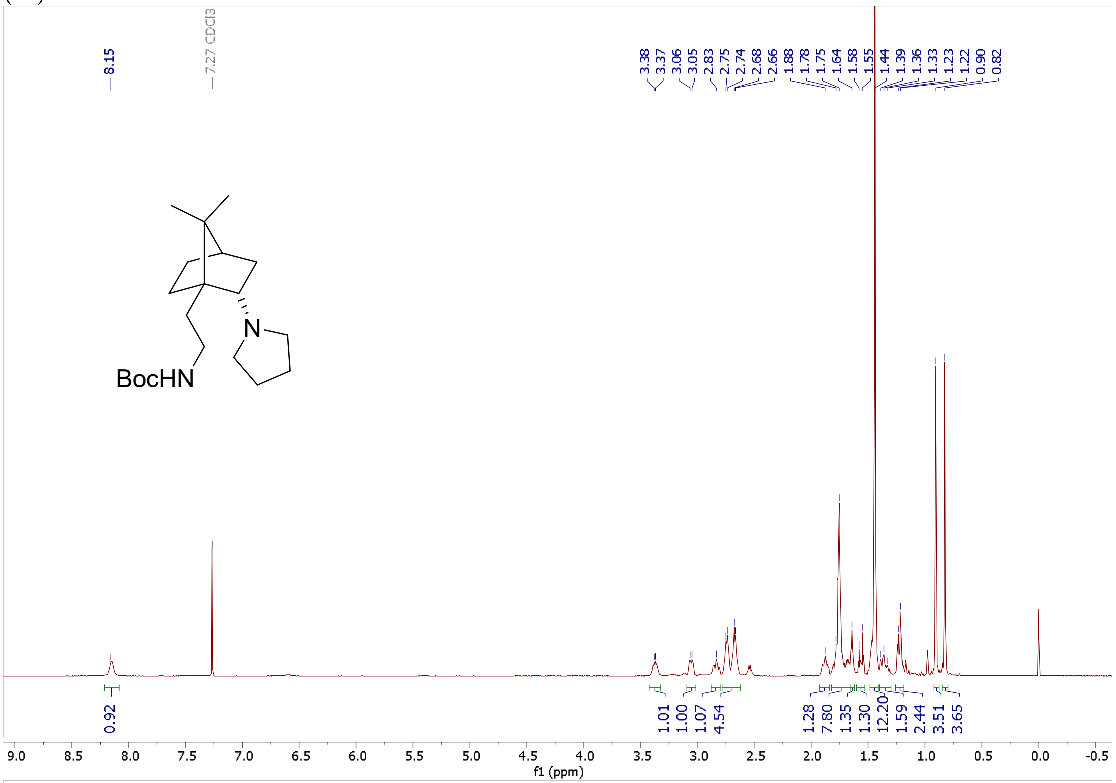

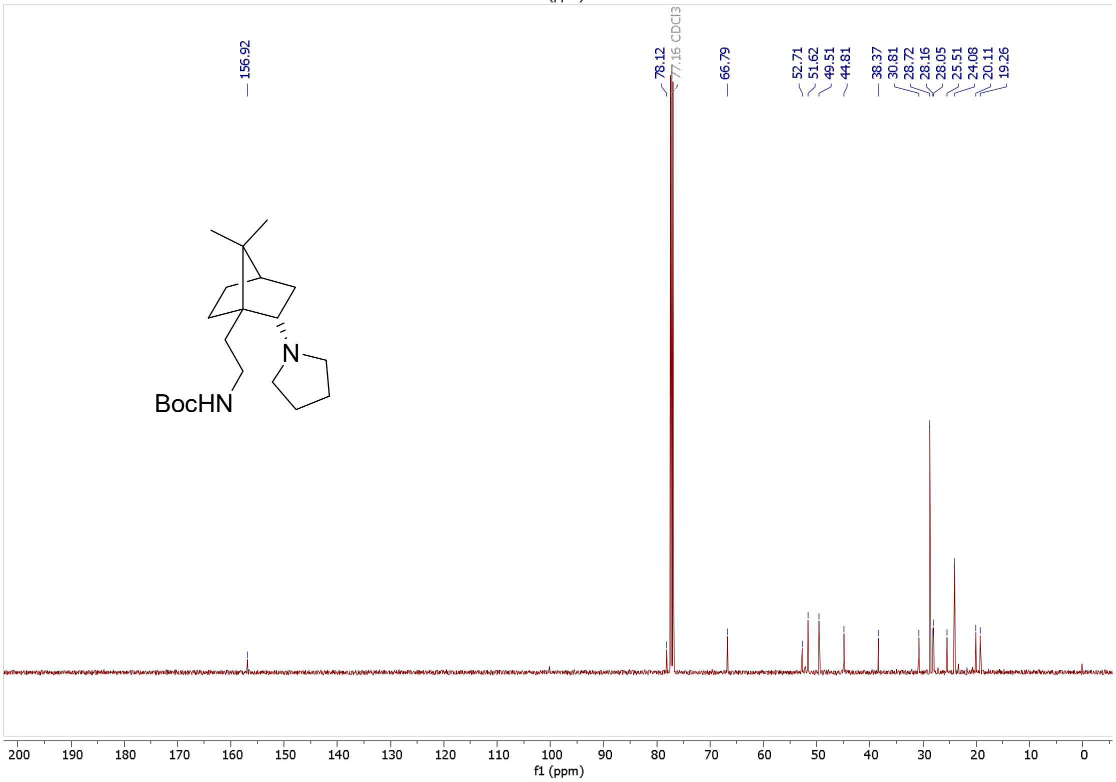

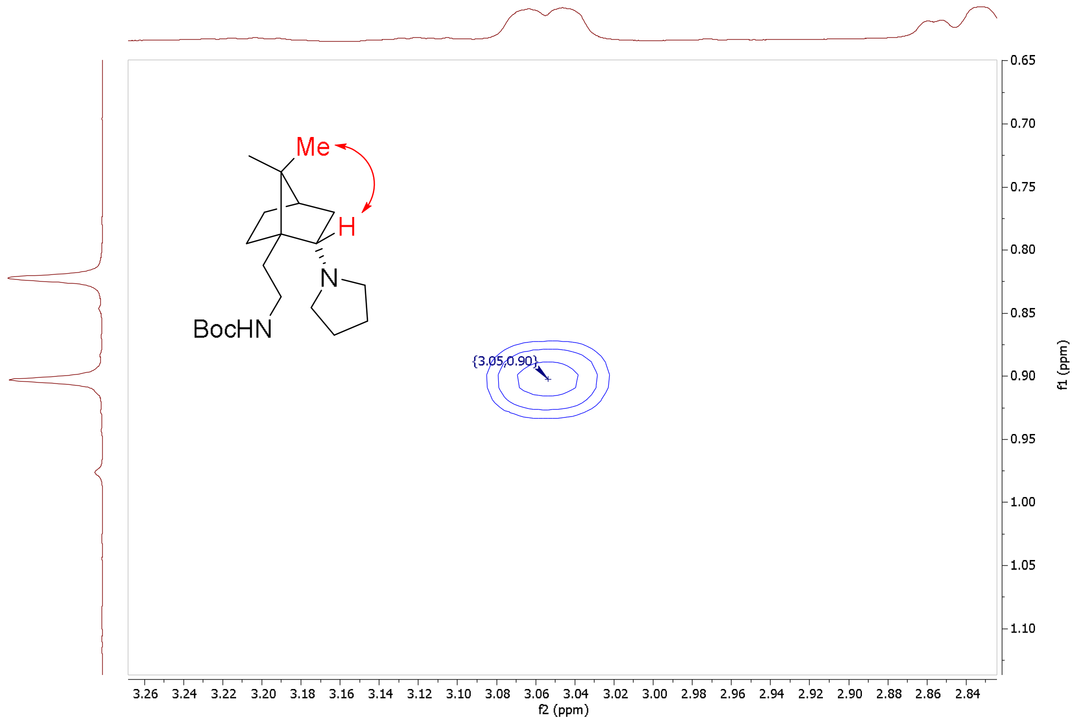
Me
H
N
BocHN
{3.05,0.90}
f1 (ppm)
f2 (ppm)
0.65
0.70
0.75
0.80
0.85
0.90
0.95
1.00
1.05
1.10
3.26 3.24 3.22 3.20 3.18 3.16 3.14 3.12 3.10 3.08 3.06 3.04 3.02 3.00 2.98 2.96 2.94 2.92 2.90 2.88 2.86 2.84

2D NOESY

1-(3,5-Bis(trifluoromethyl)phenyl)-3-((1*S*,2*R*,4*R*)-7,7-dimethyl-1-(pyrrolidin-1-yl)bicyclo[2.2.1]heptan-2-yl)thiourea (**48**)

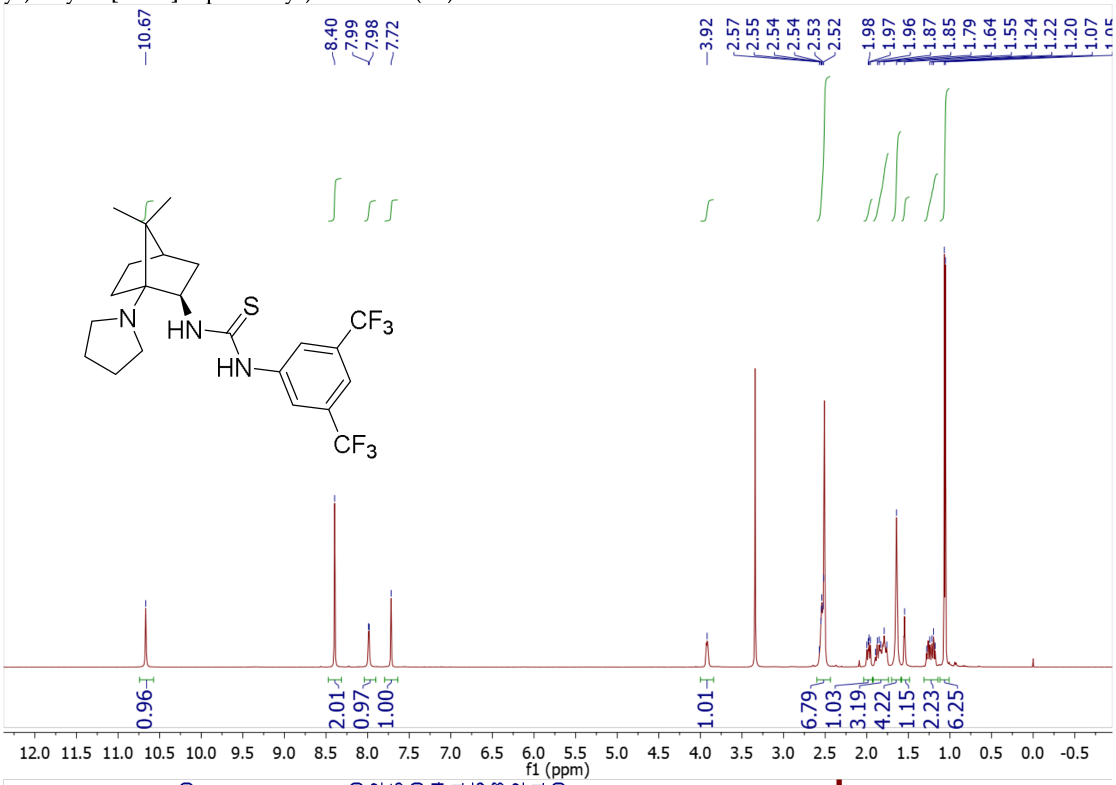

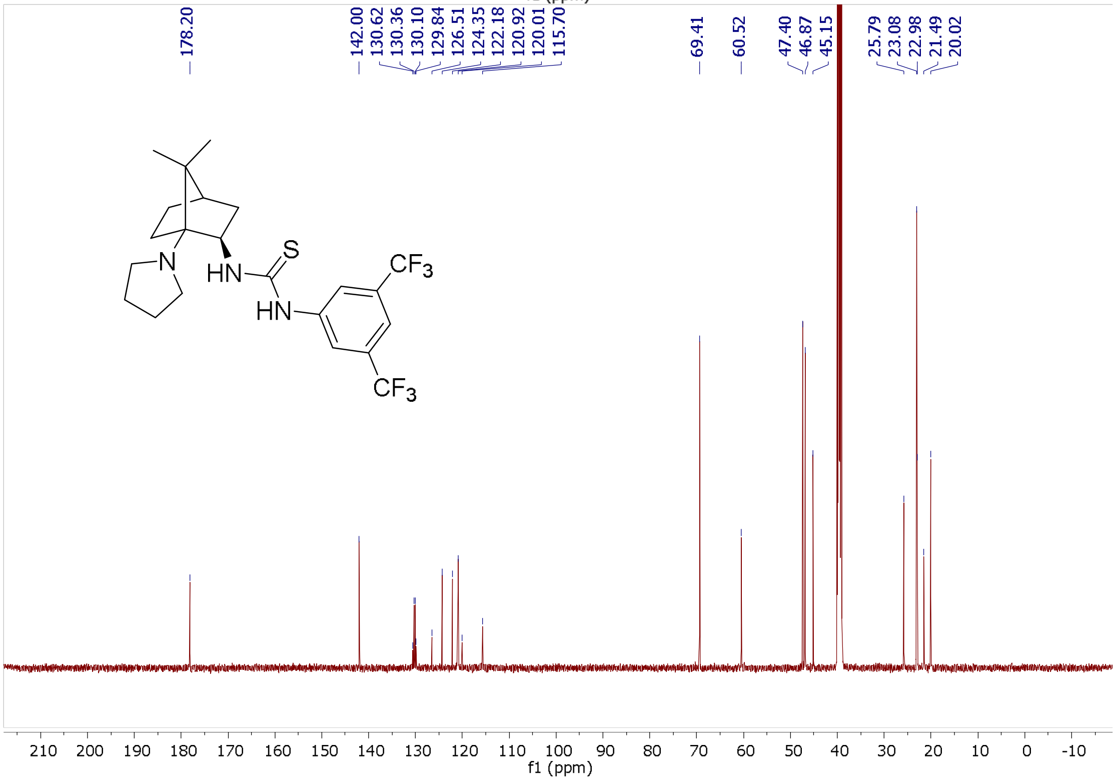

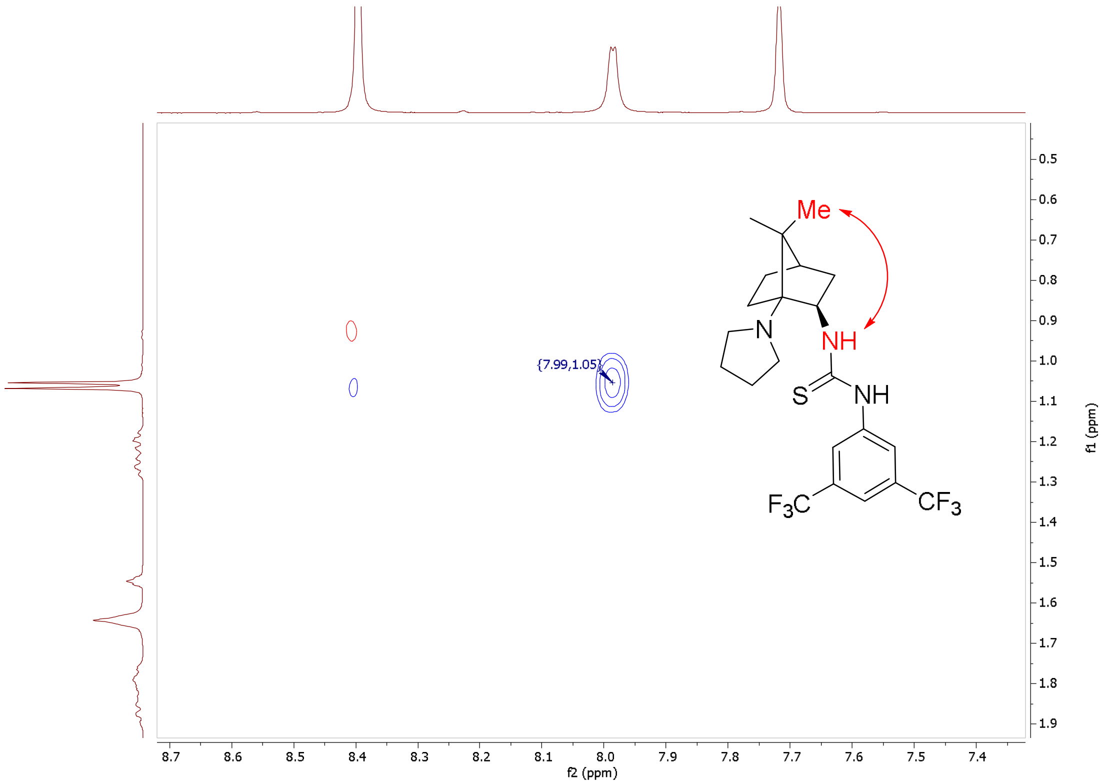

2D NOESY

1-(3,5-Bis(trifluoromethyl)phenyl)-3-((1*S*,2*S*,4*R*)-7,7-dimethyl-1-(pyrrolidin-1-yl)bicyclo[2.2.1]heptan-2-yl)thiourea (**49**)

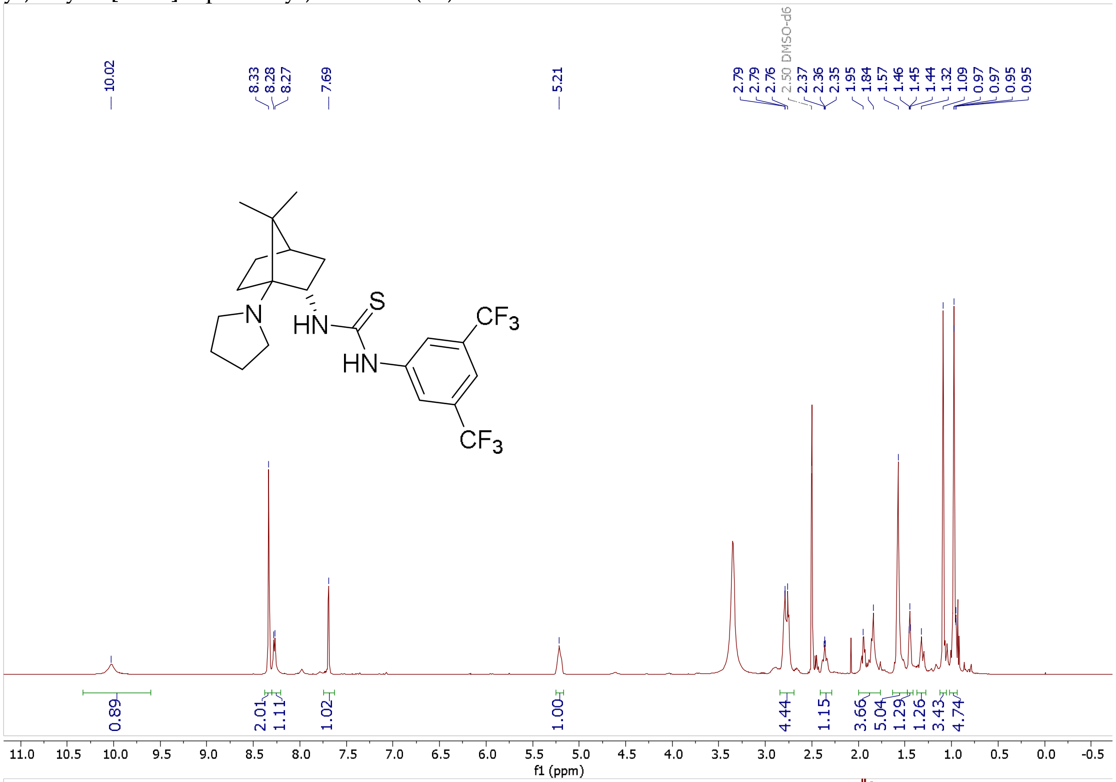

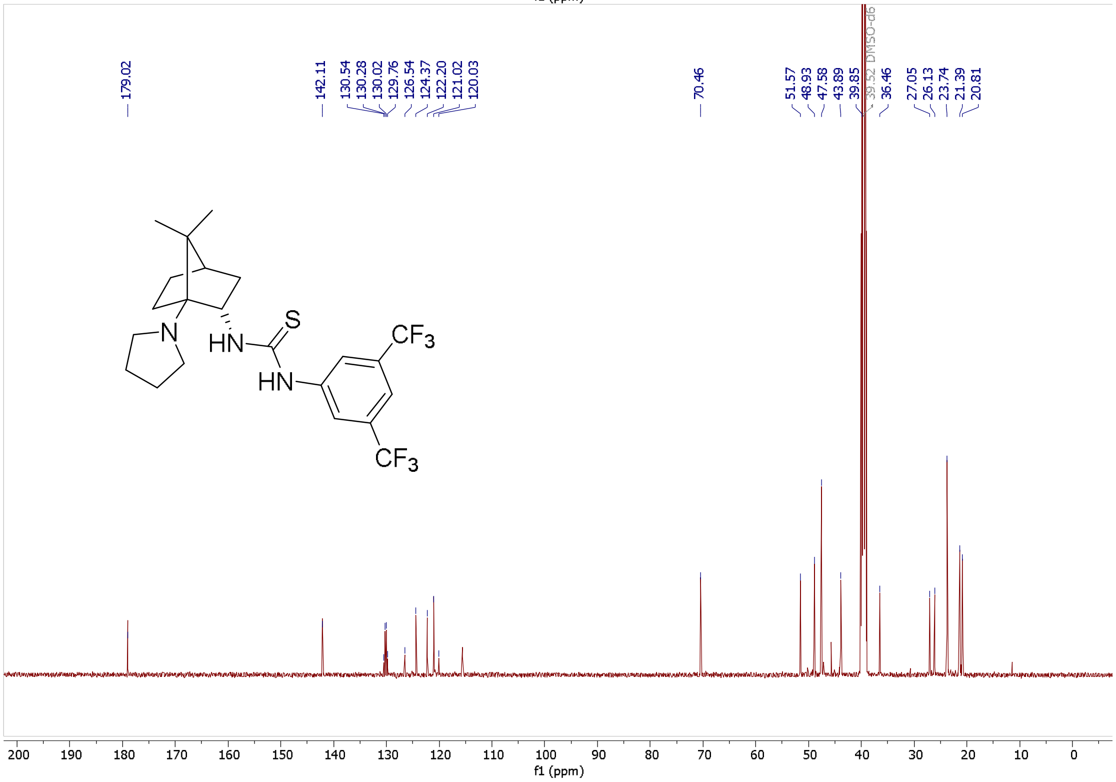

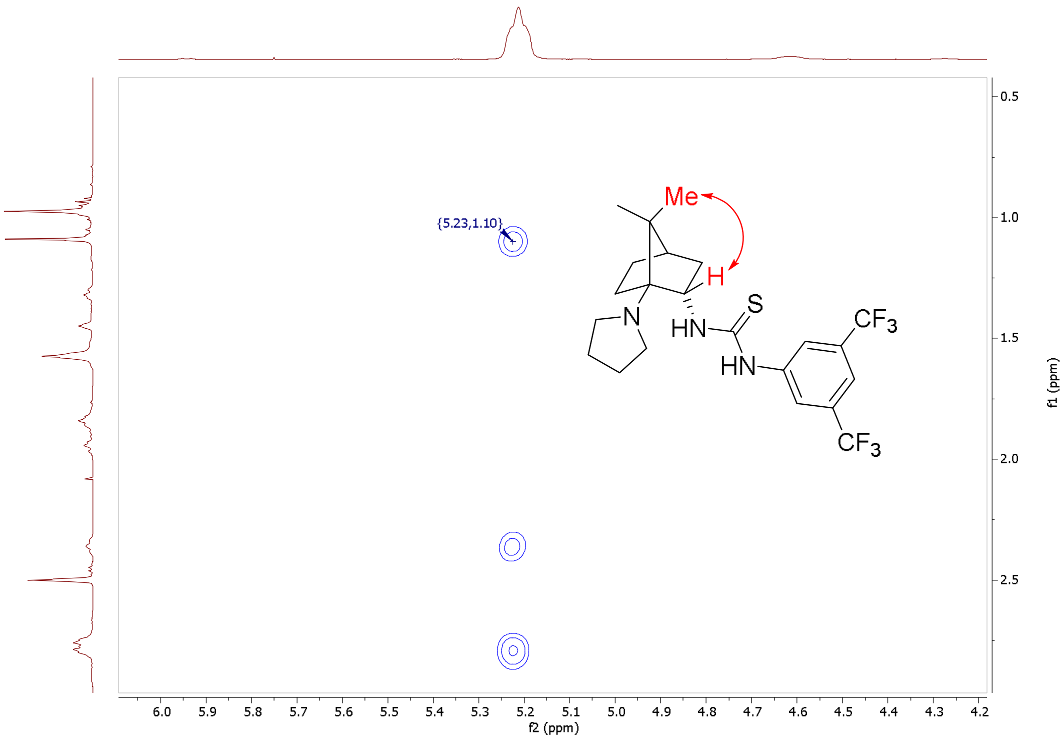

2D NOESY

1-(3,5-Bis(trifluoromethyl)phenyl)-3-((1*S*,2*R*,4*R*)-7,7-dimethyl-2-(pyrrolidin-1-yl)bicyclo[2.2.1]heptan-1-yl)thiourea (**50**)

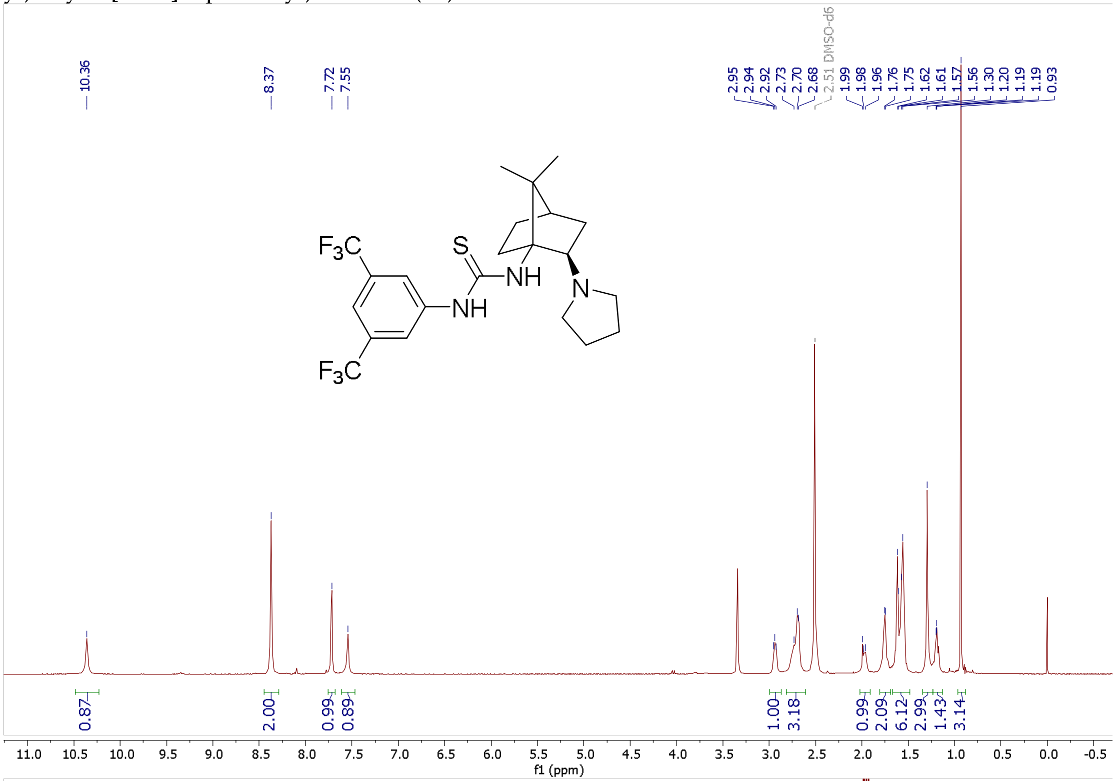

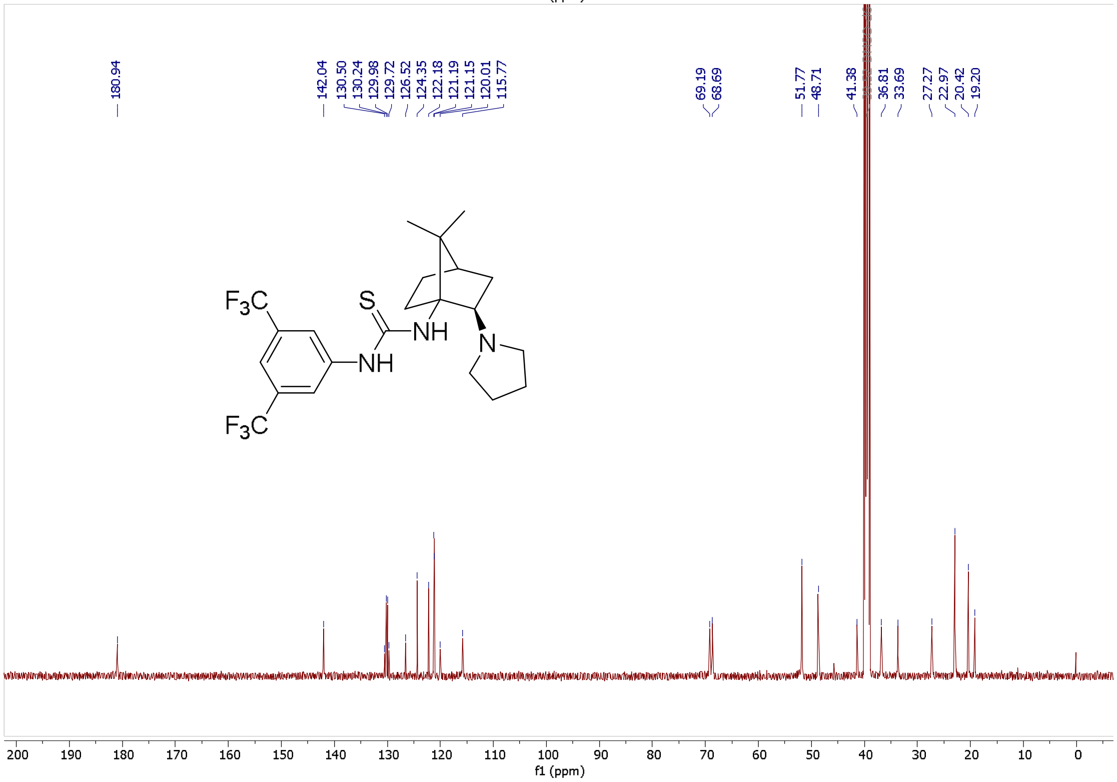

1-(3,5-Bis(trifluoromethyl)phenyl)-3-((1*S*,2*S*,4*R*)-7,7-dimethyl-2-(pyrrolidin-1-yl)bicyclo[2.2.1]heptan-1-yl)thiourea (**51**)

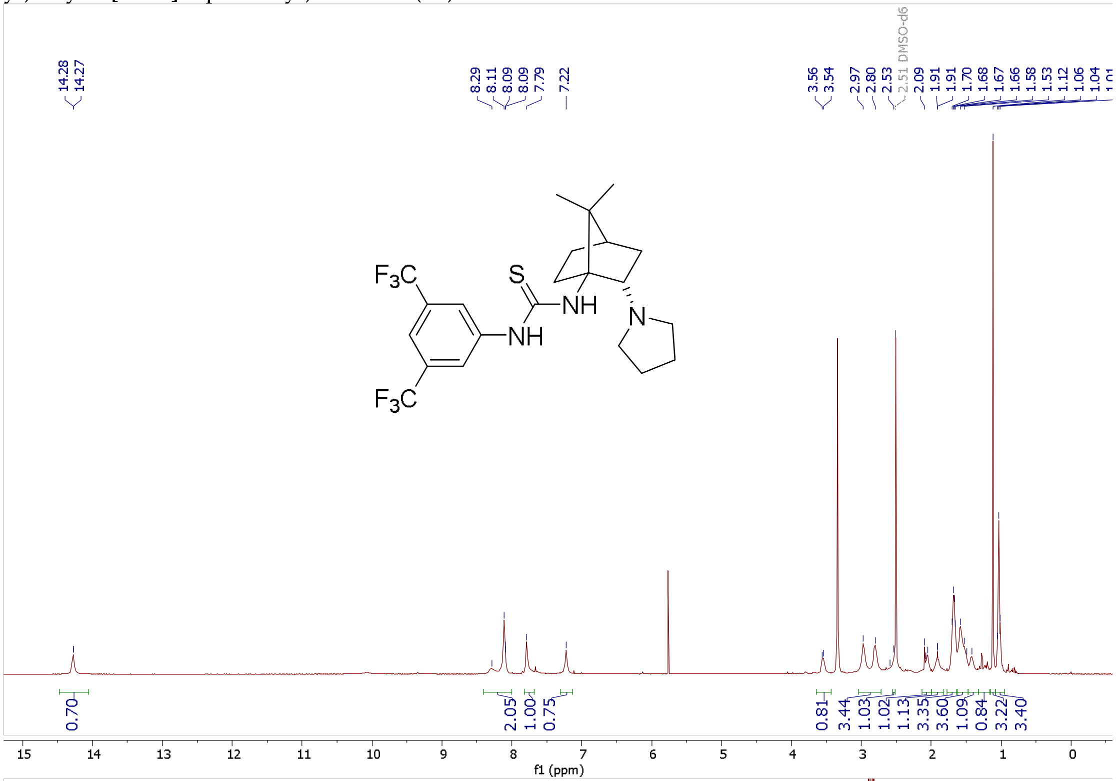

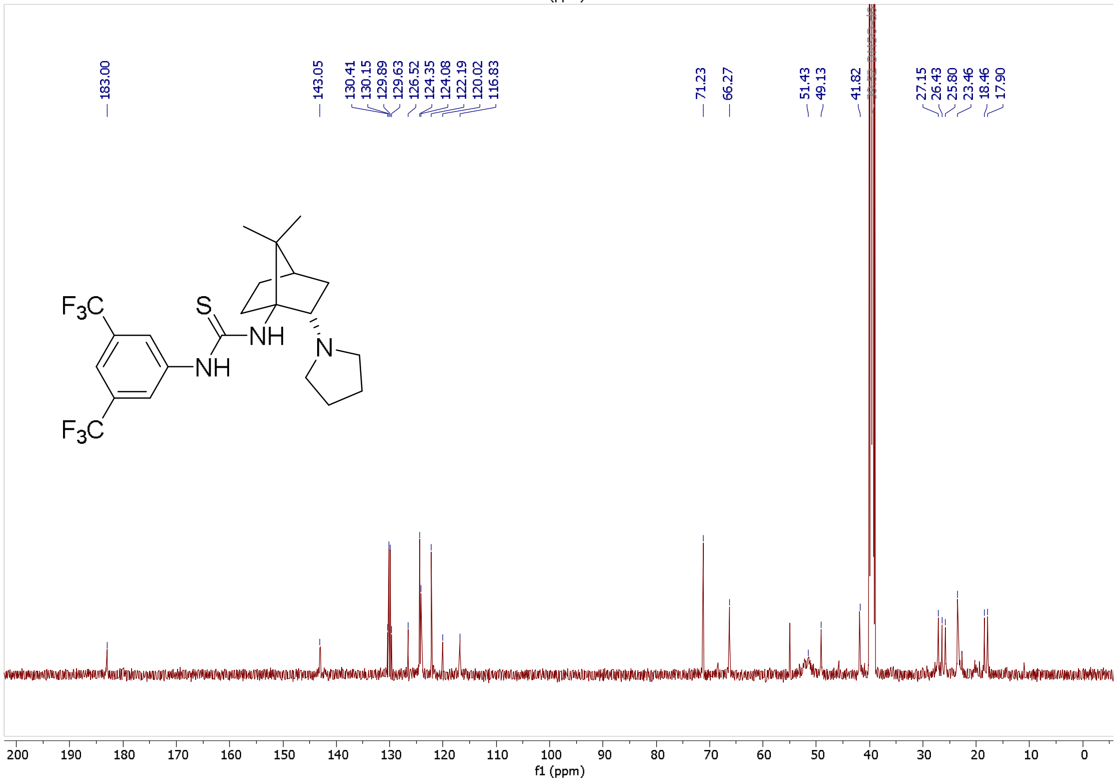

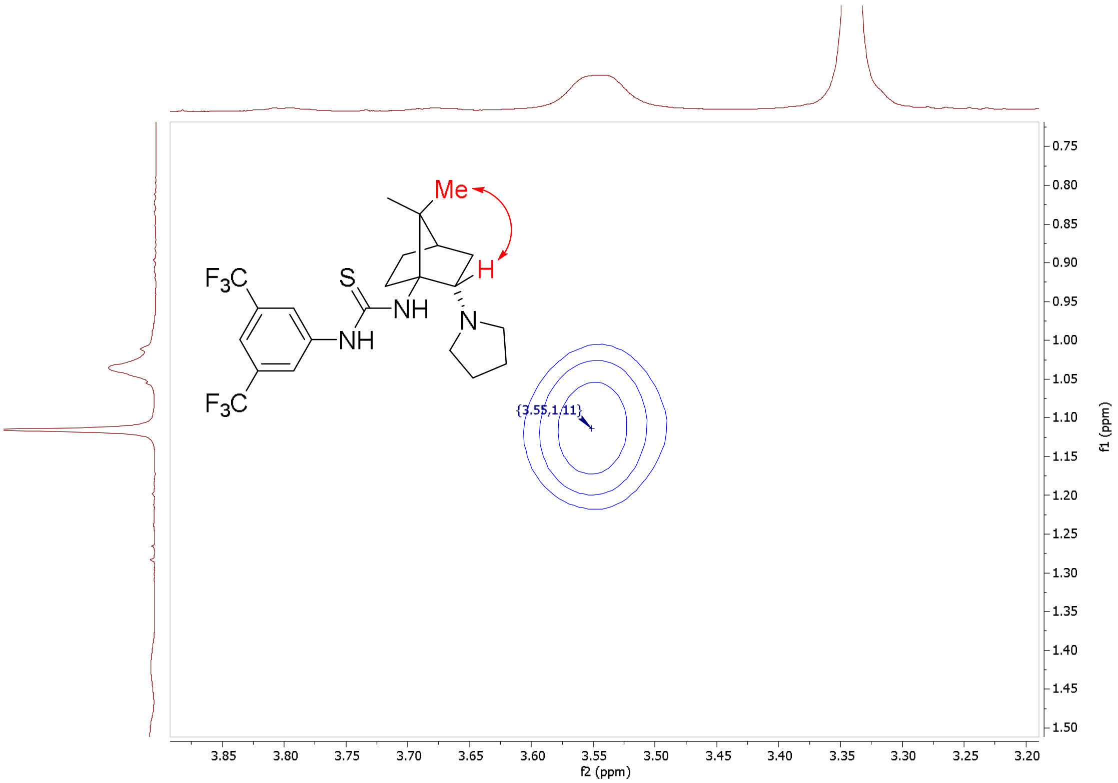

2D NOESY

1-(3,5-Bis(trifluoromethyl)phenyl)-3-((1*R*,2*S*,4*R*)-7,7-dimethyl-1-(pyrrolidin-1-ylmethyl)bicyclo[2.2.1]heptan-2-yl)thiourea (**52**)

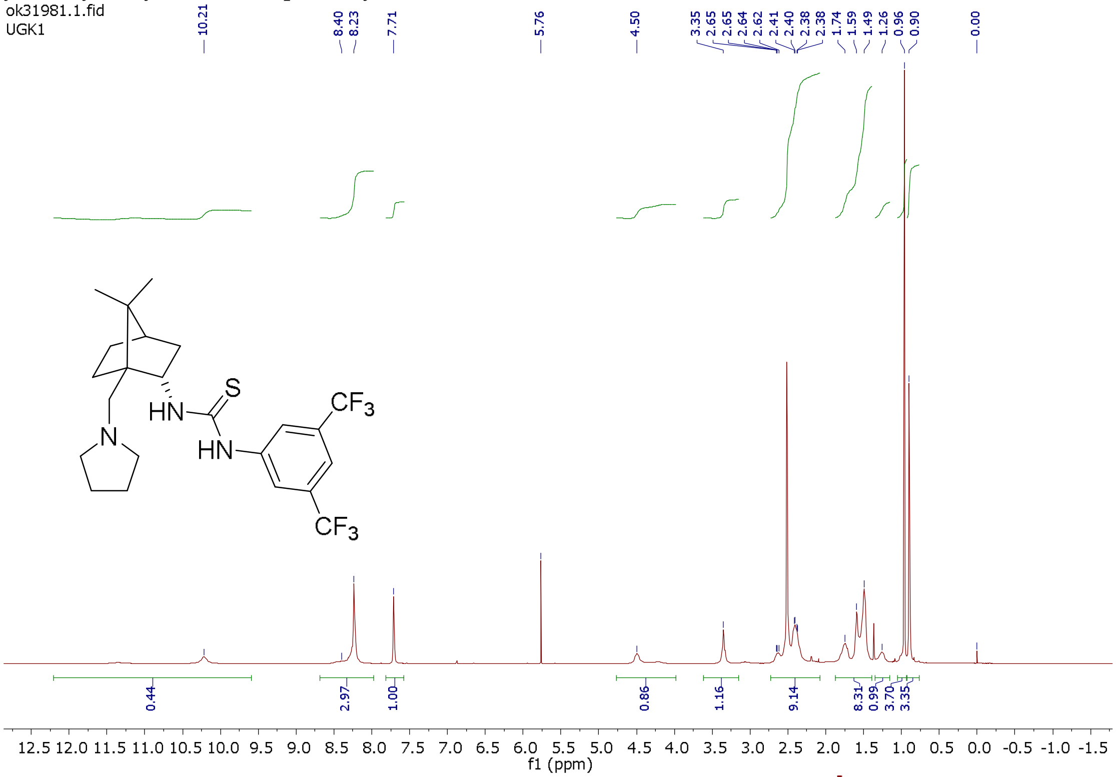

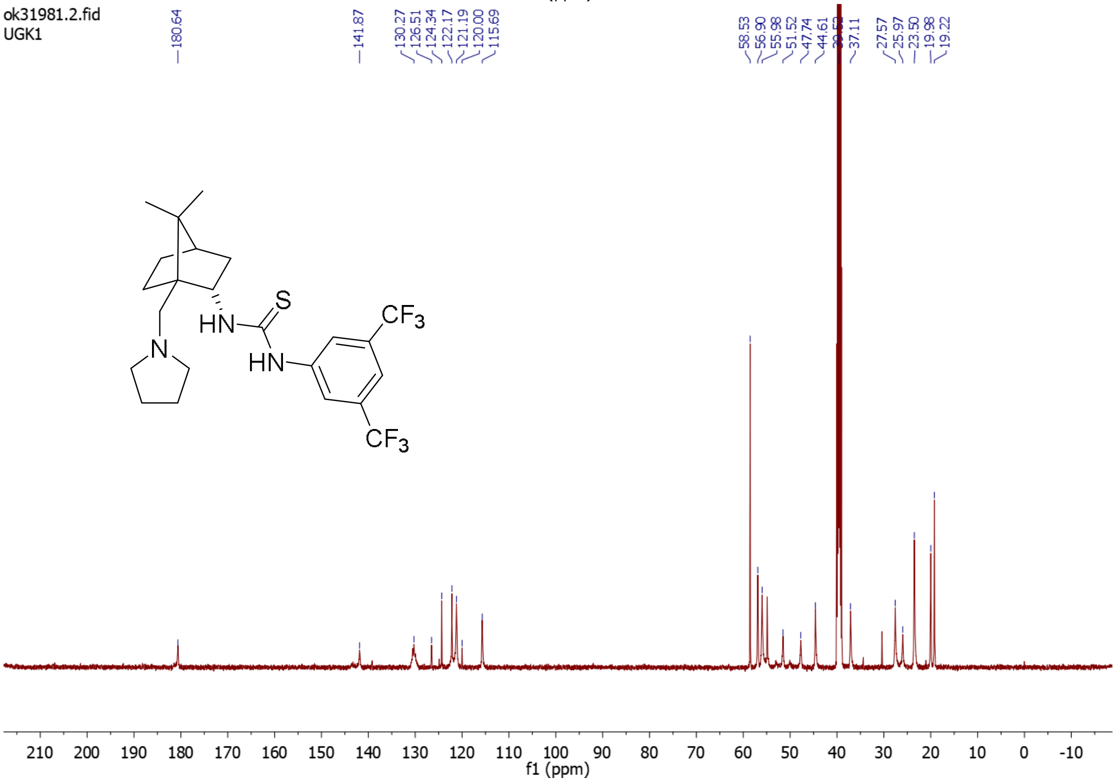

1-(3,5-Bis(trifluoromethyl)phenyl)-3-((1*R*,2*R*,4*R*)-7,7-dimethyl-1-(pyrrolidin-1-ylmethyl)bicyclo[2.2.1]heptan-2-yl)thiourea (**53**)

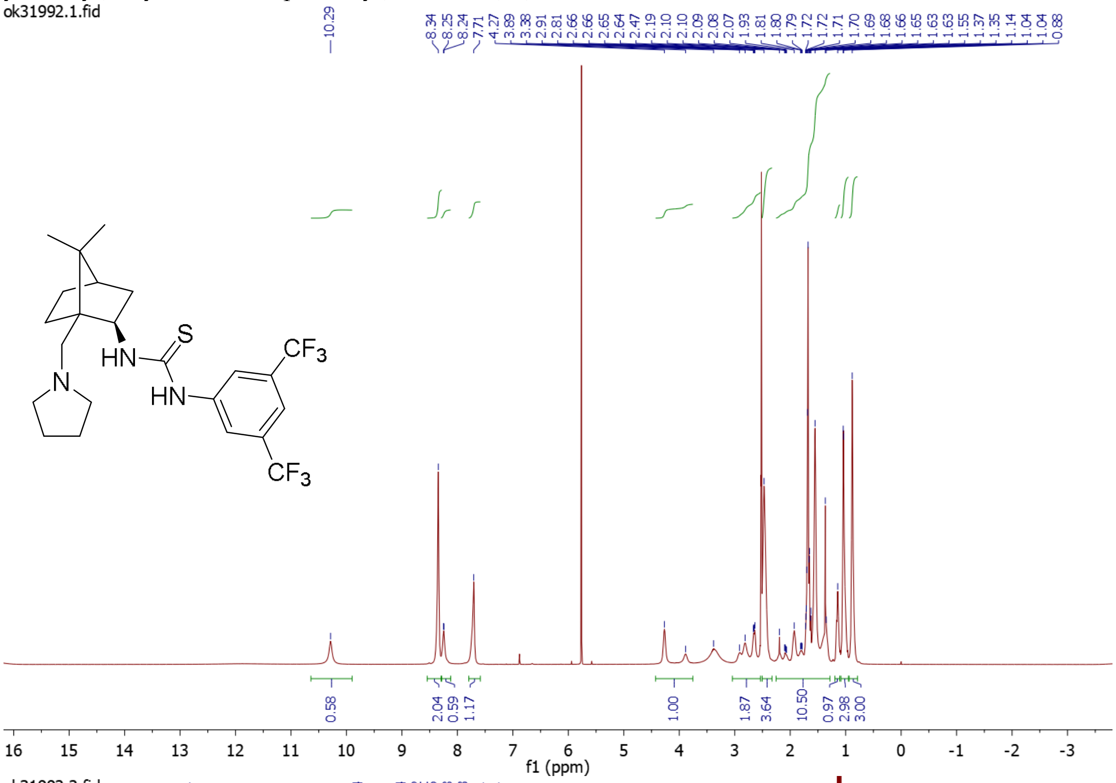

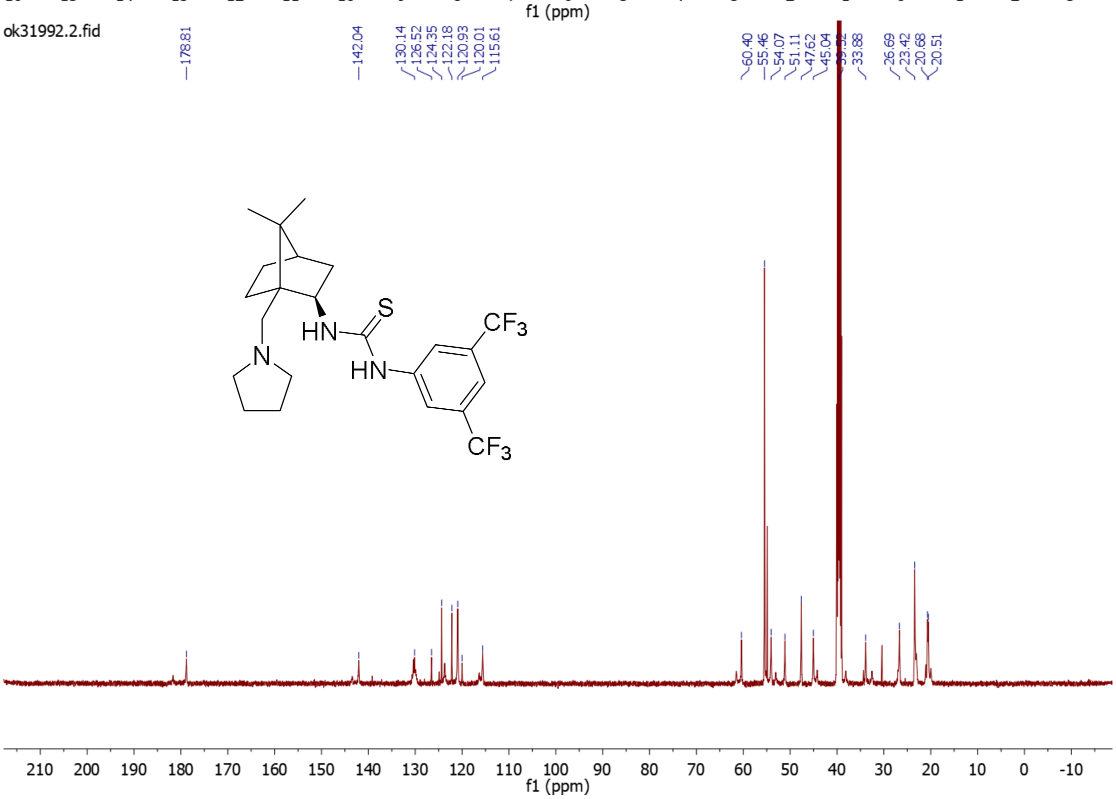

1-(3,5-Bis(trifluoromethyl)phenyl)-3-((1*R*,2*S*,4*R*)-7,7-dimethyl-1-(piperidin-1-ylmethyl)bicyclo[2.2.1]heptan-2-yl)thiourea (**54**)

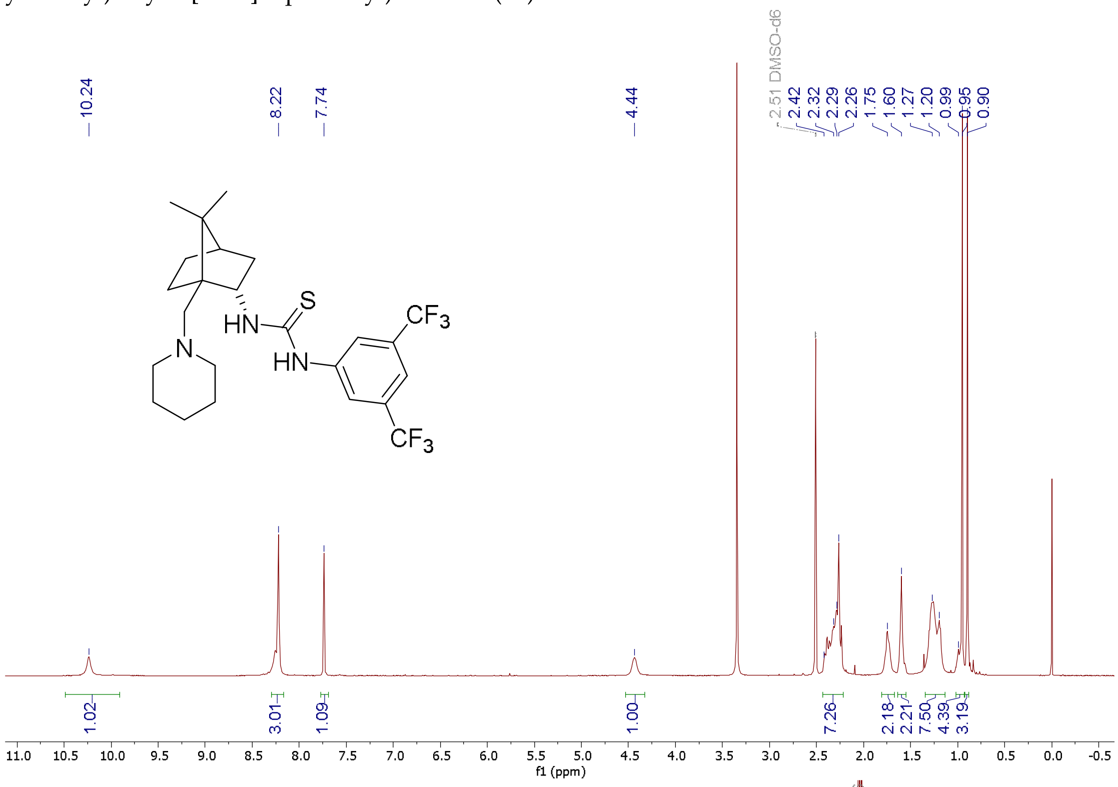

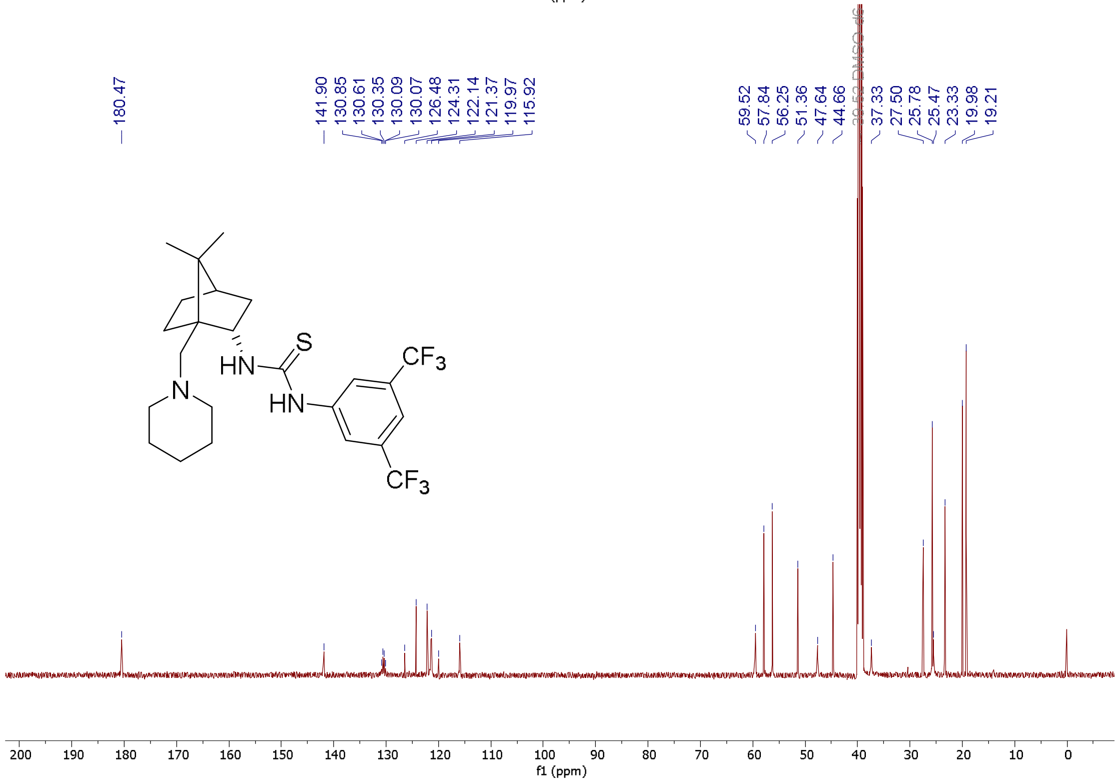

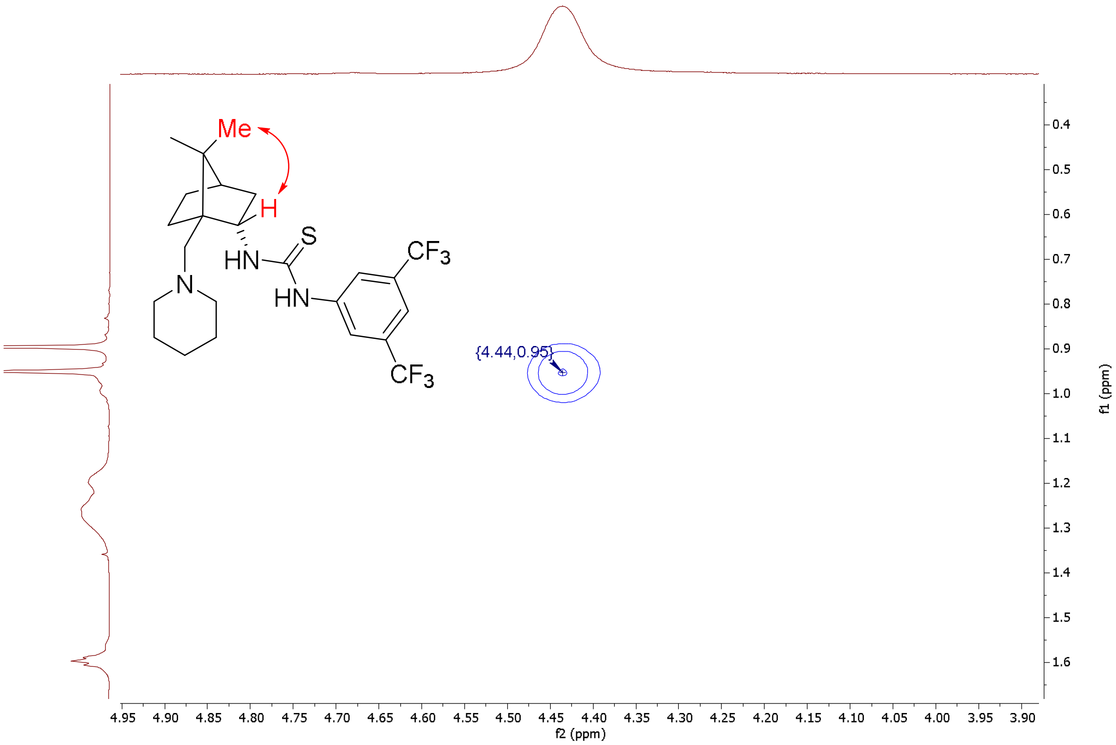

2D NOESY

1-(3,5-Bis(trifluoromethyl)phenyl)-3-((1*R*,2*S*,4*R*)-7,7-dimethyl-1-(morpholinomethyl)bicyclo[2.2.1]heptan-2-yl)thiourea (**55**)

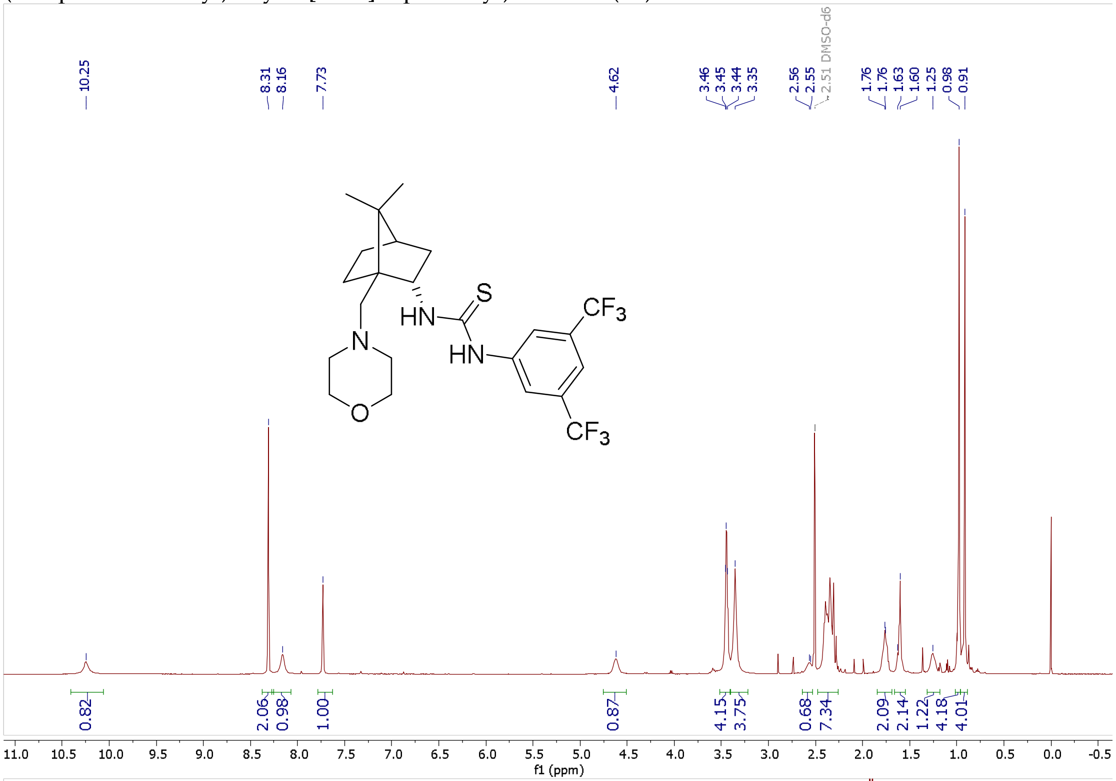

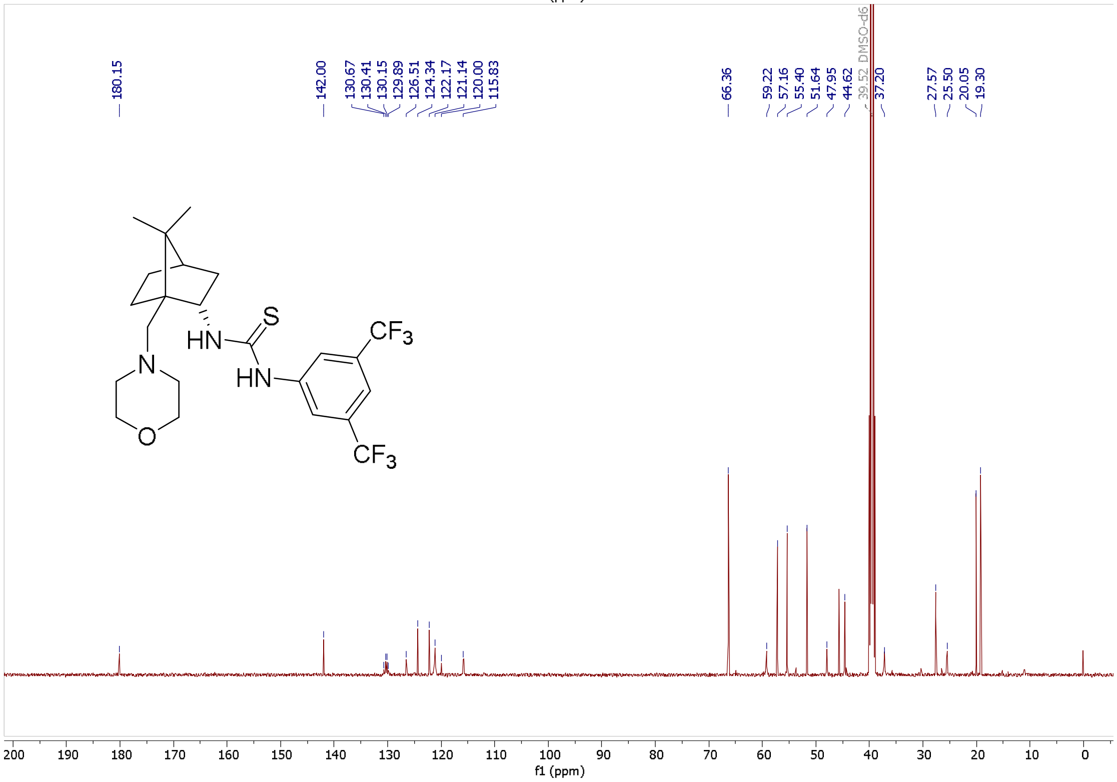

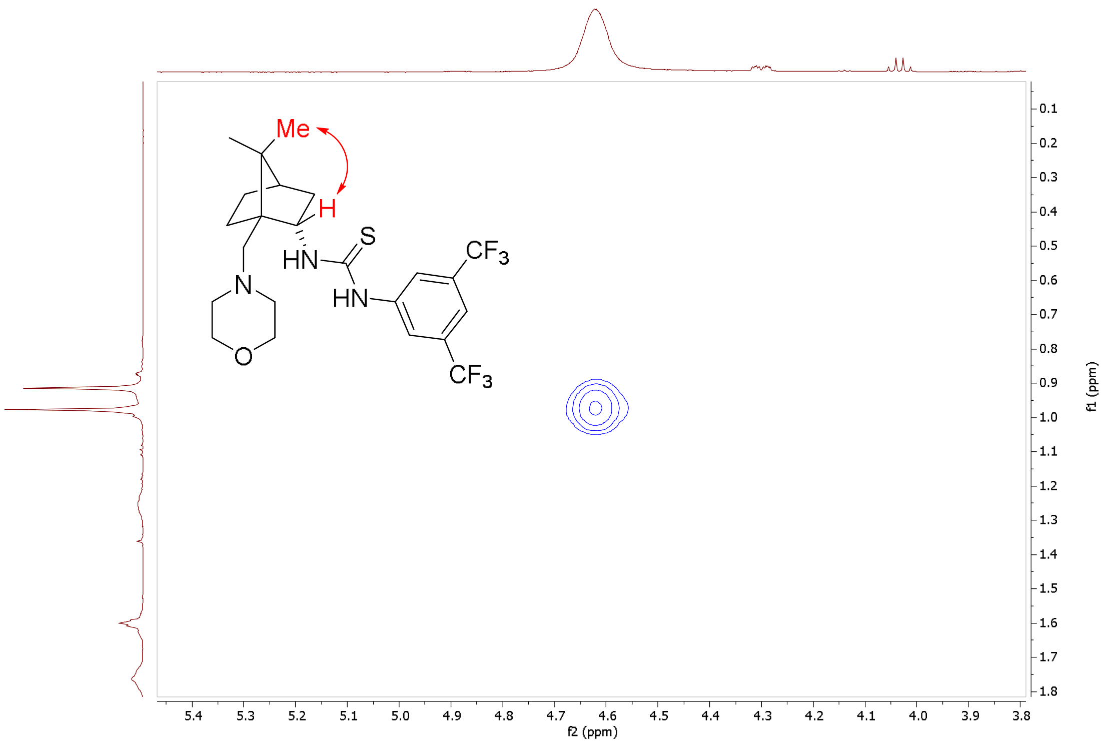

2D NOESY

1-(3,5-Bis(trifluoromethyl)phenyl)-3-((1*R*,2*S*,4*R*)-1-((dimethylamino)methyl)-7,7-dimethylbicyclo[2.2.1]heptan-2-yl)thiourea (**56**)

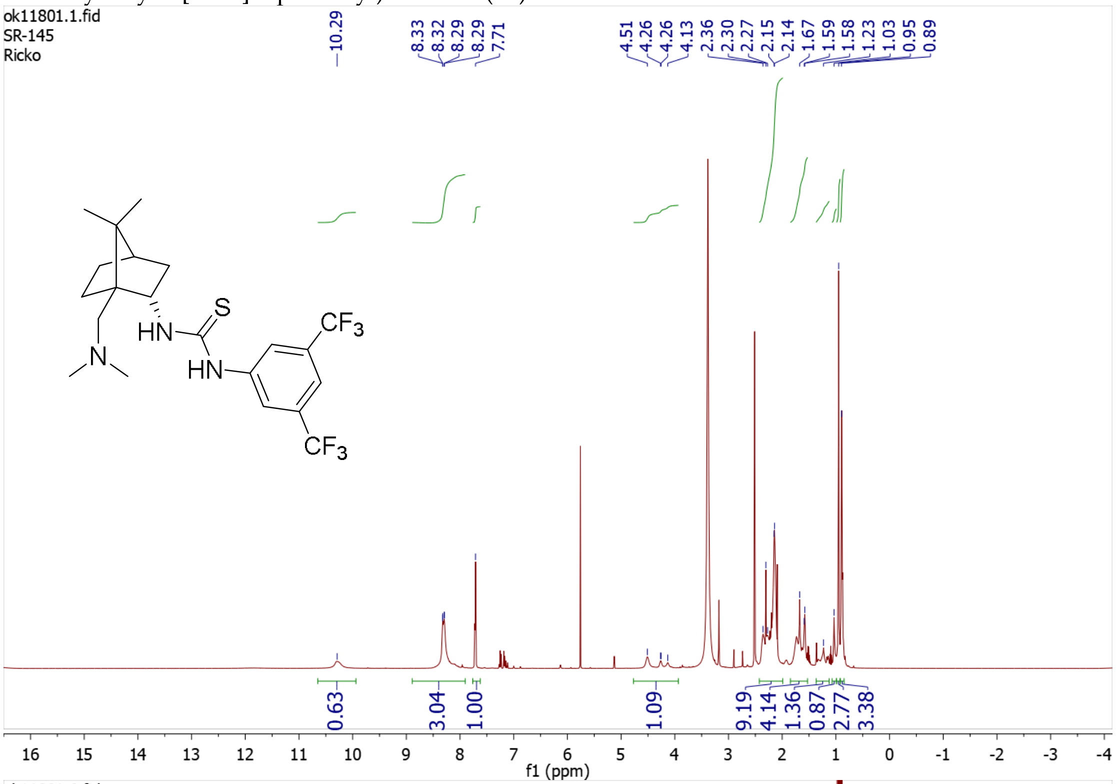

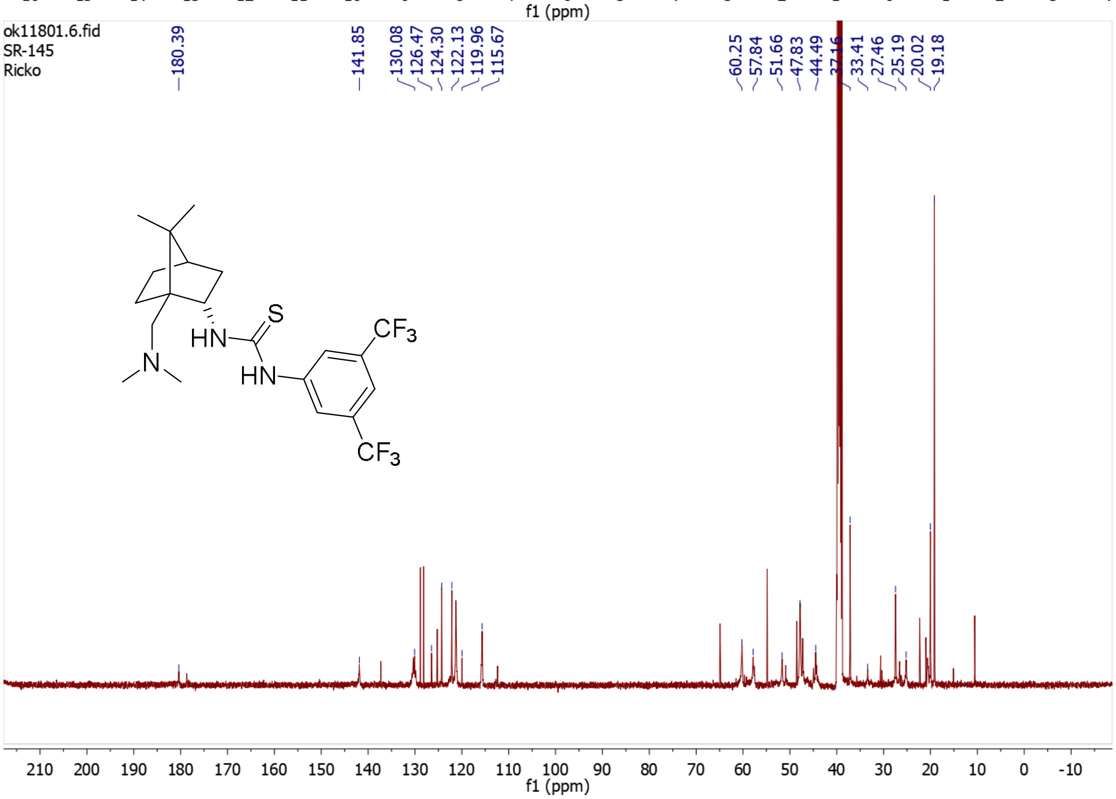

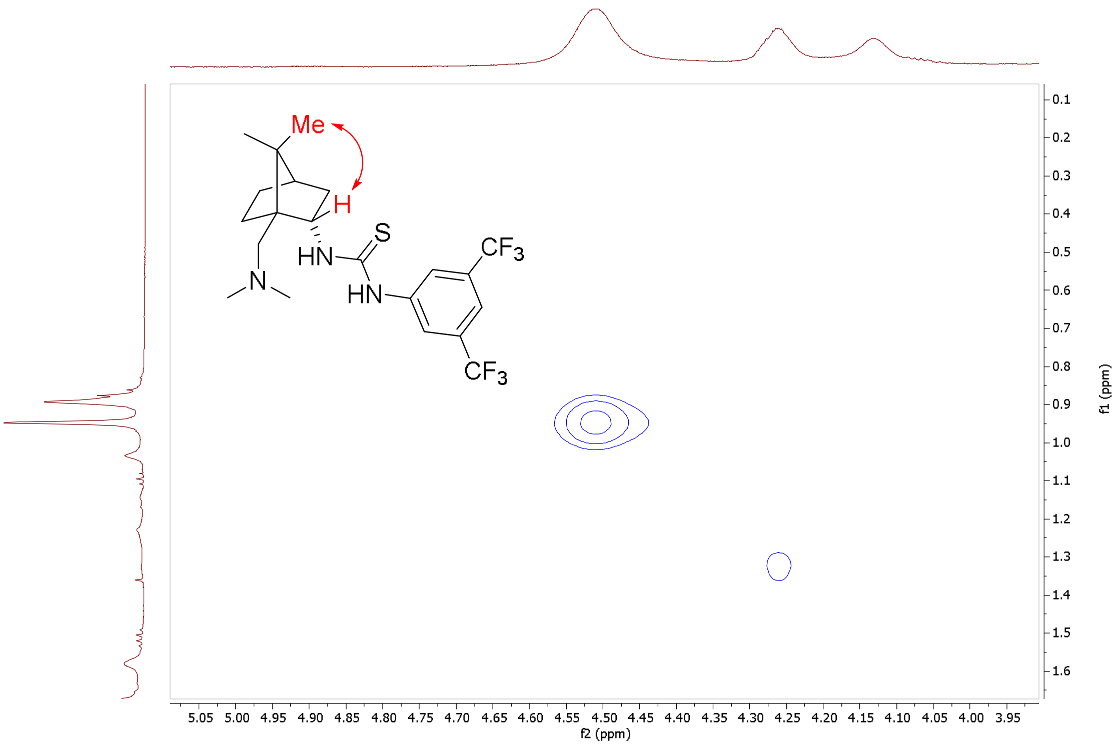

2D NOESY

1-(*tert*-Butyl)-3-((1*R*,2*S*,4*R*)-7,7-dimethyl-1-(pyrrolidin-1-ylmethyl)bicyclo[2.2.1]heptan-2-yl)thiourea (**57**)

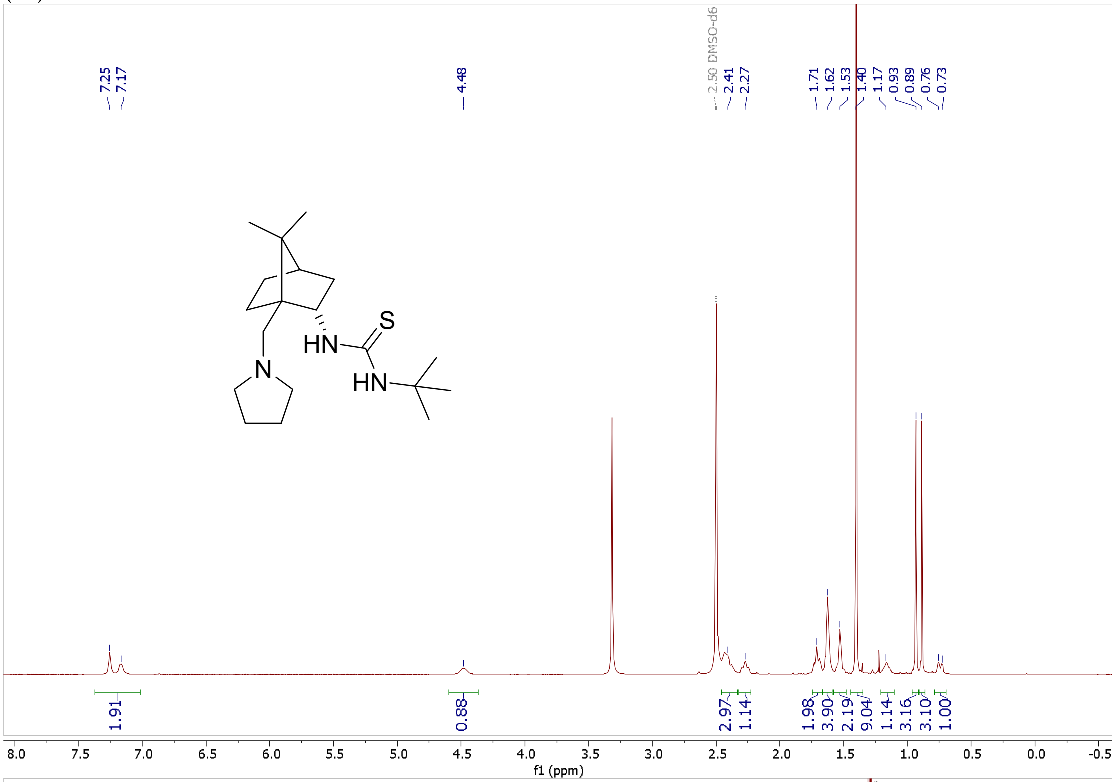

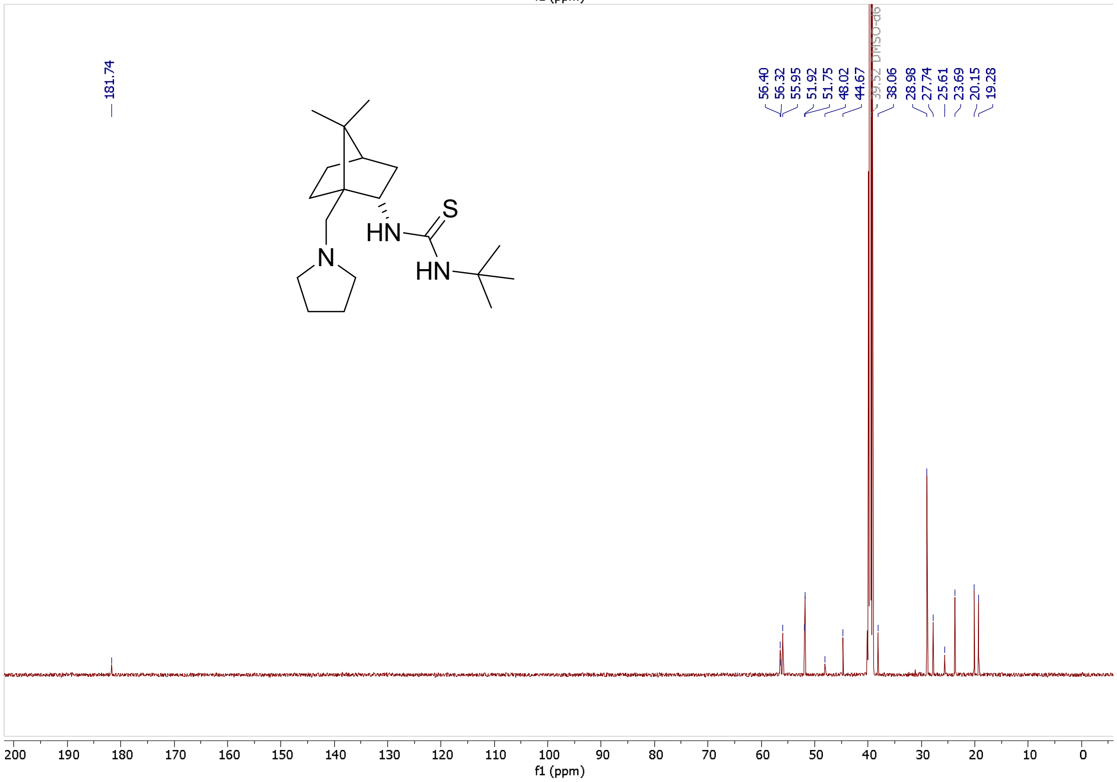

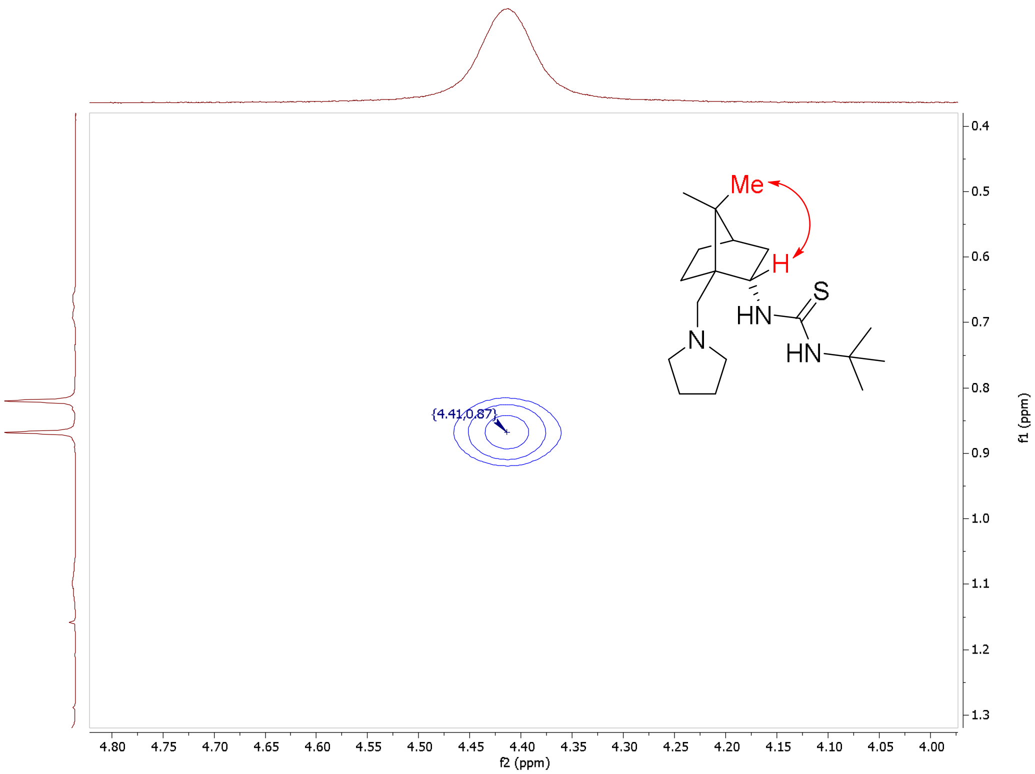
Me
H
S
HN
HN
N
{4.41,0.87}
f2 (ppm)
f1 (ppm)
4.80 4.75 4.70 4.65 4.60 4.55 4.50 4.45 4.40 4.35 4.30 4.25 4.20 4.15 4.10 4.05 4.00
0.4 0.5 0.6 0.7 0.8 0.9 1.0 1.1 1.2 1.3

2D NOESY

1-(Adamantan-1-yl)-3-((1*R*,2*S*,4*R*)-7,7-dimethyl-1-(pyrrolidin-1-ylmethyl)bicyclo[2.2.1]heptan-2-yl)thiourea (**58**)

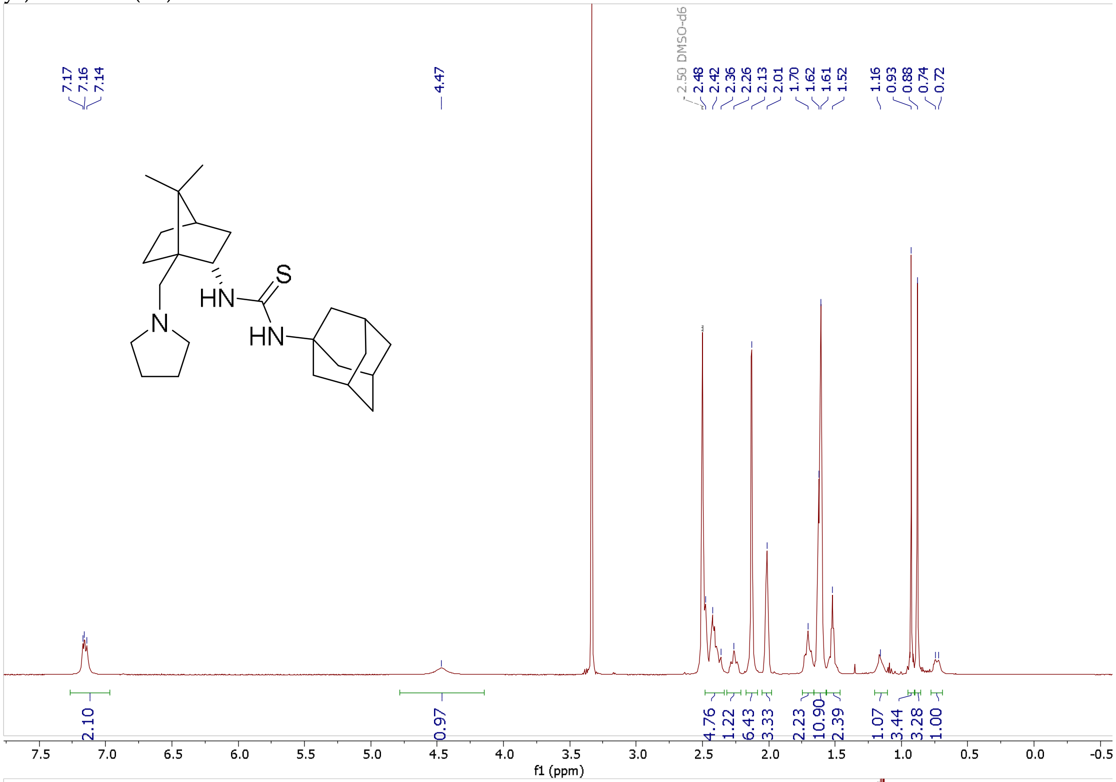

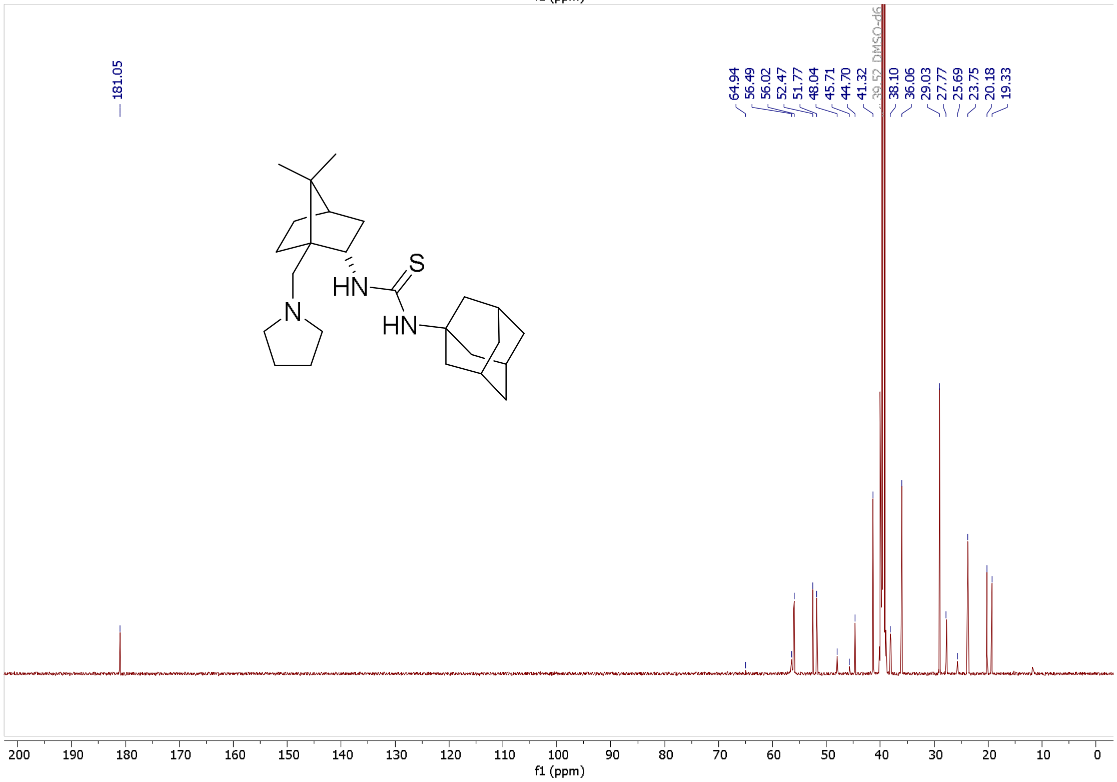

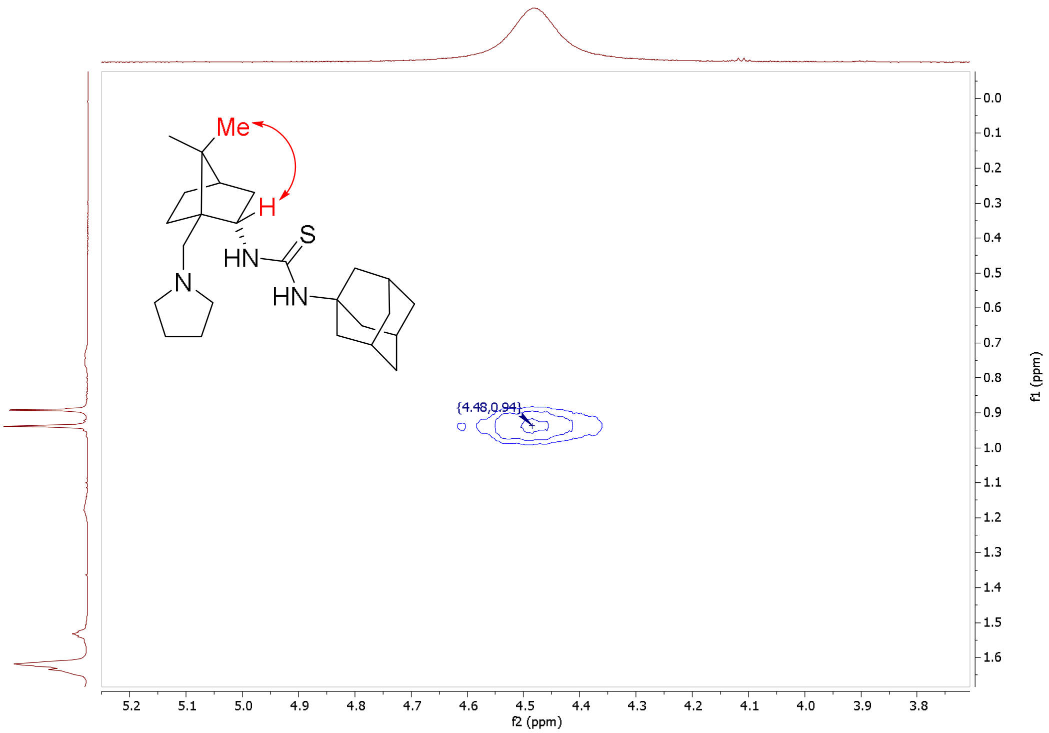

2D NOESY

1-(3,5-Bis(trifluoromethyl)phenyl)-3-(((1*R*,2*S*,4*R*)-7,7-dimethyl-2-(pyrrolidin-1-yl)bicyclo[2.2.1]heptan-1-yl)methyl)thiourea (**59**)

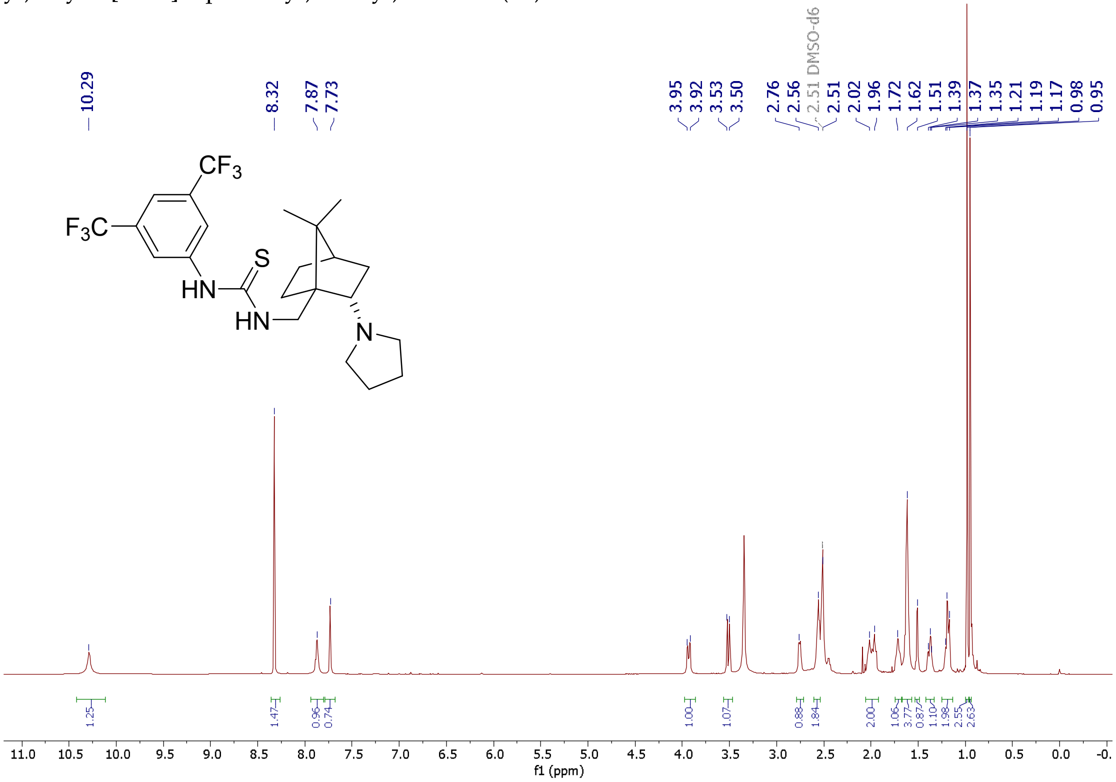

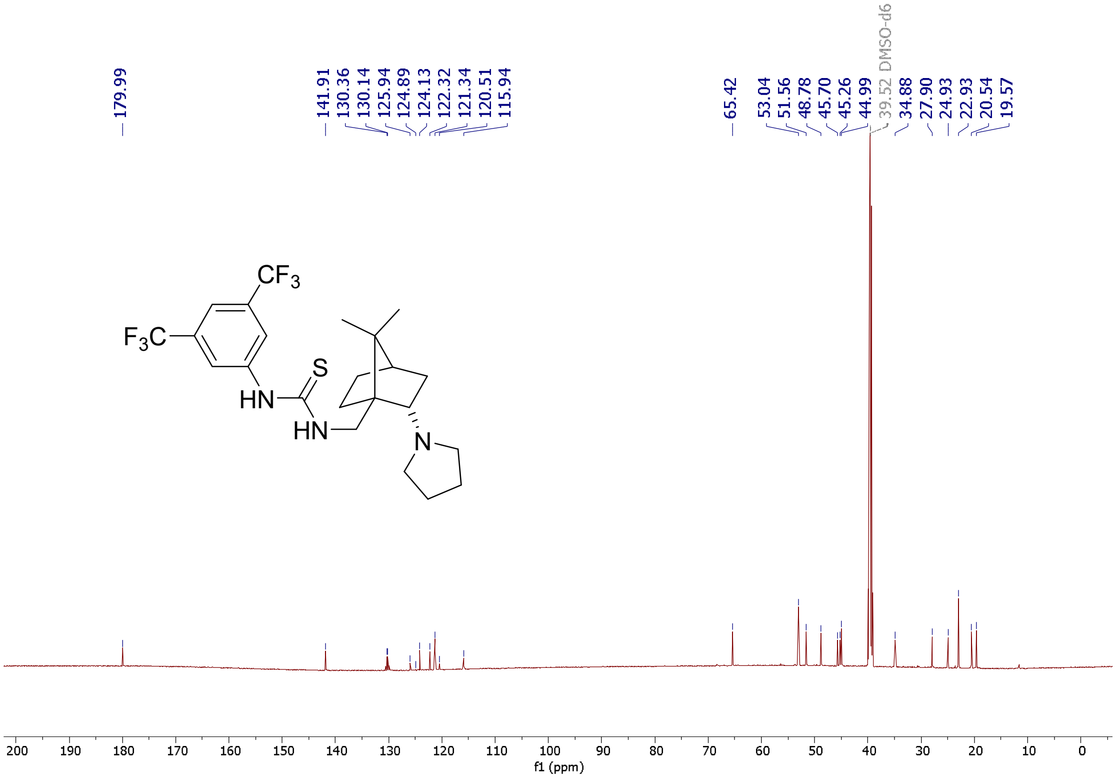

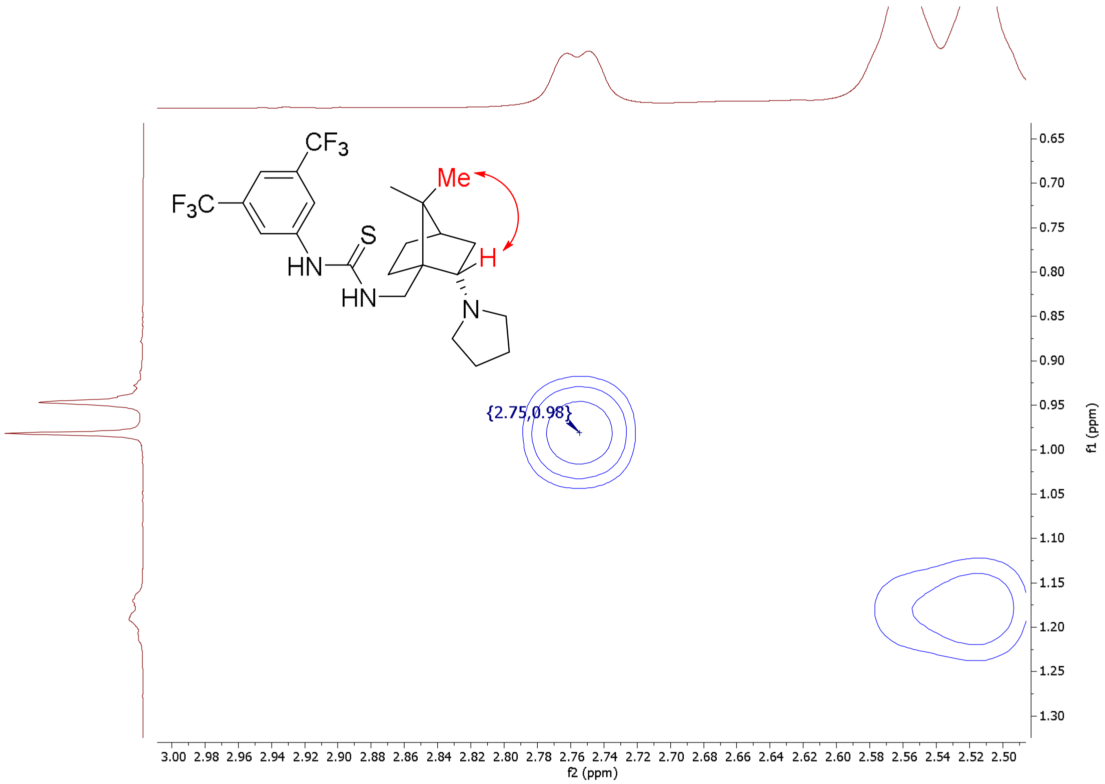

2D NOESY

1-(3,5-Bis(trifluoromethyl)phenyl)-3-(((1*R*,2*R*,4*R*)-7,7-dimethyl-2-(pyrrolidin-1-yl)bicyclo[2.2.1]heptan-1-yl)methyl)thiourea (**60**)

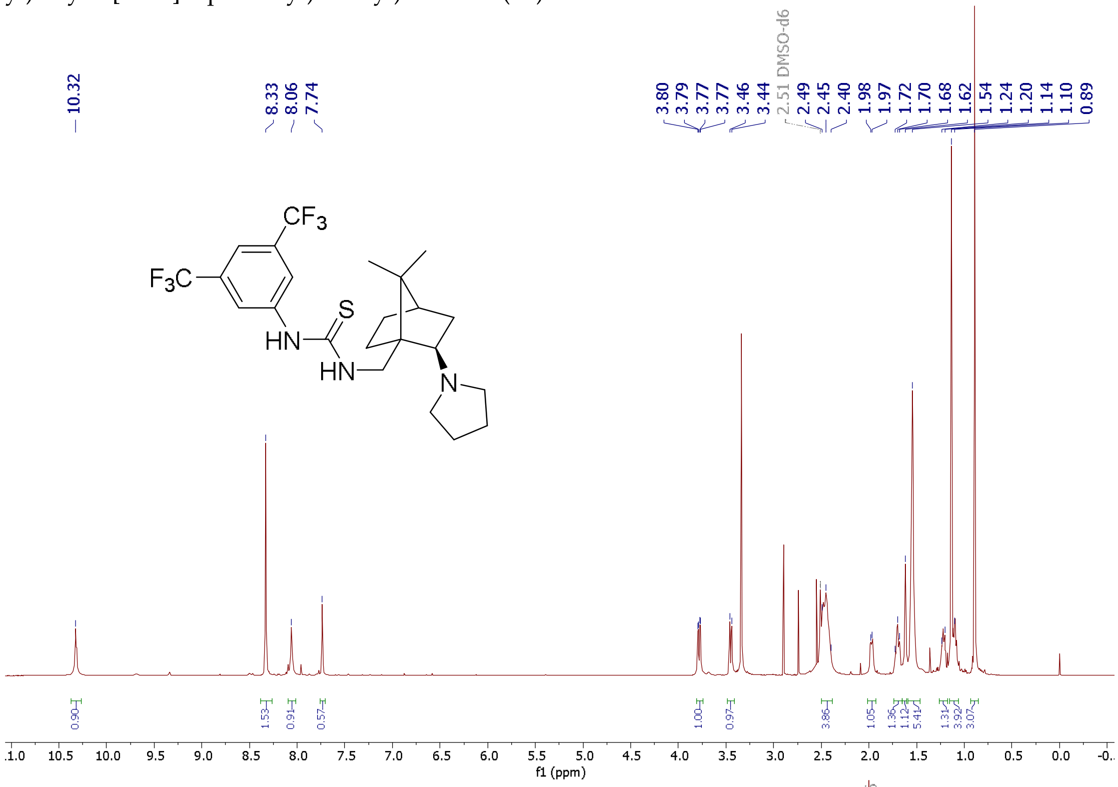

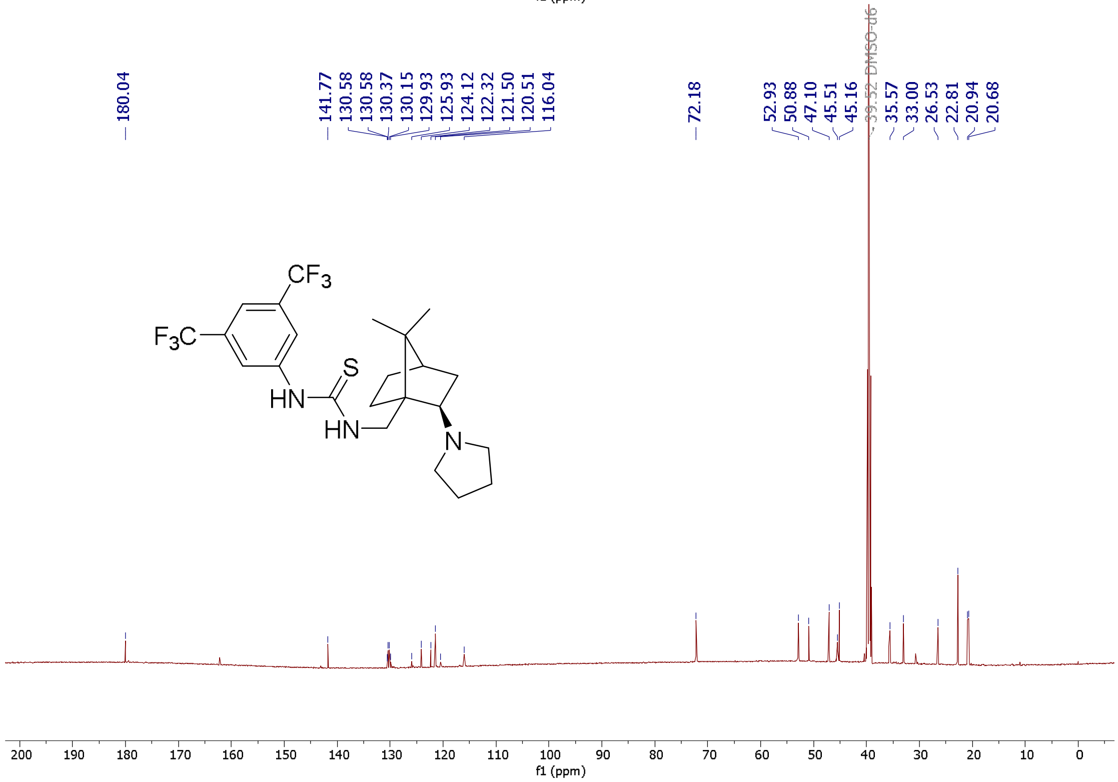

1-(3,5-Bis(trifluoromethyl)phenyl)-3-((1*S*,2*S*,4*R*)-7,7-dimethyl-1-(2-(pyrrolidin-1-yl)ethyl)bicyclo[2.2.1]heptan-2-yl)thiourea (**61**)

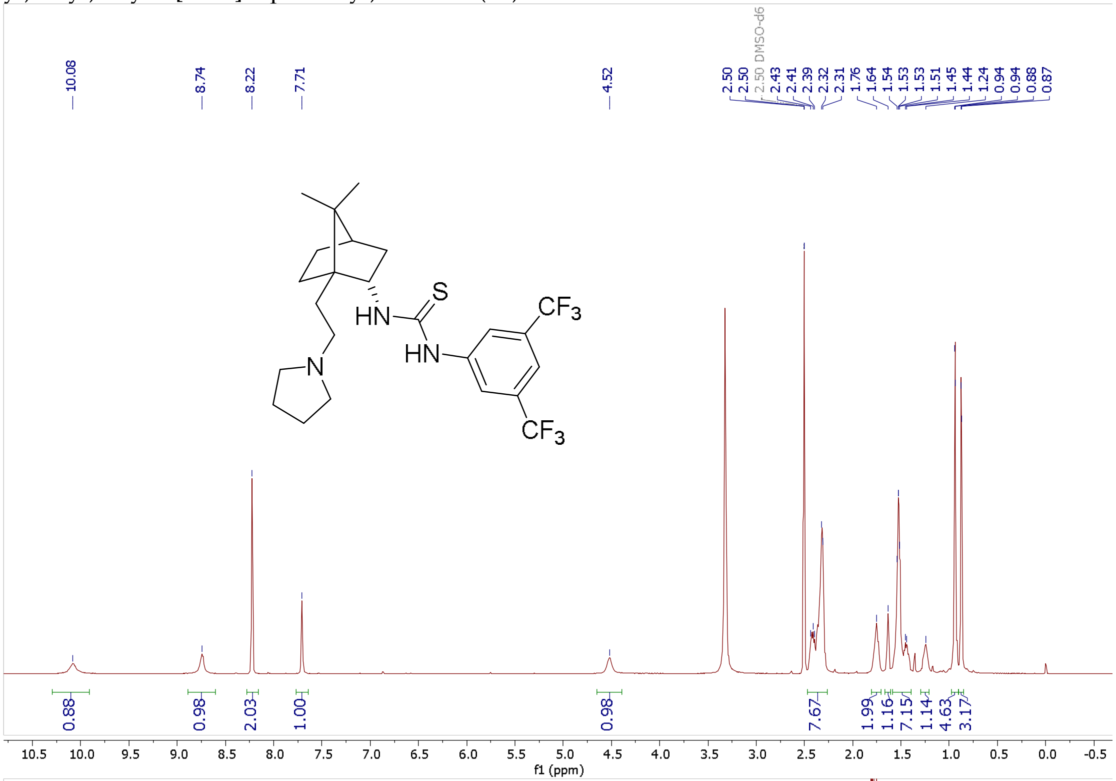

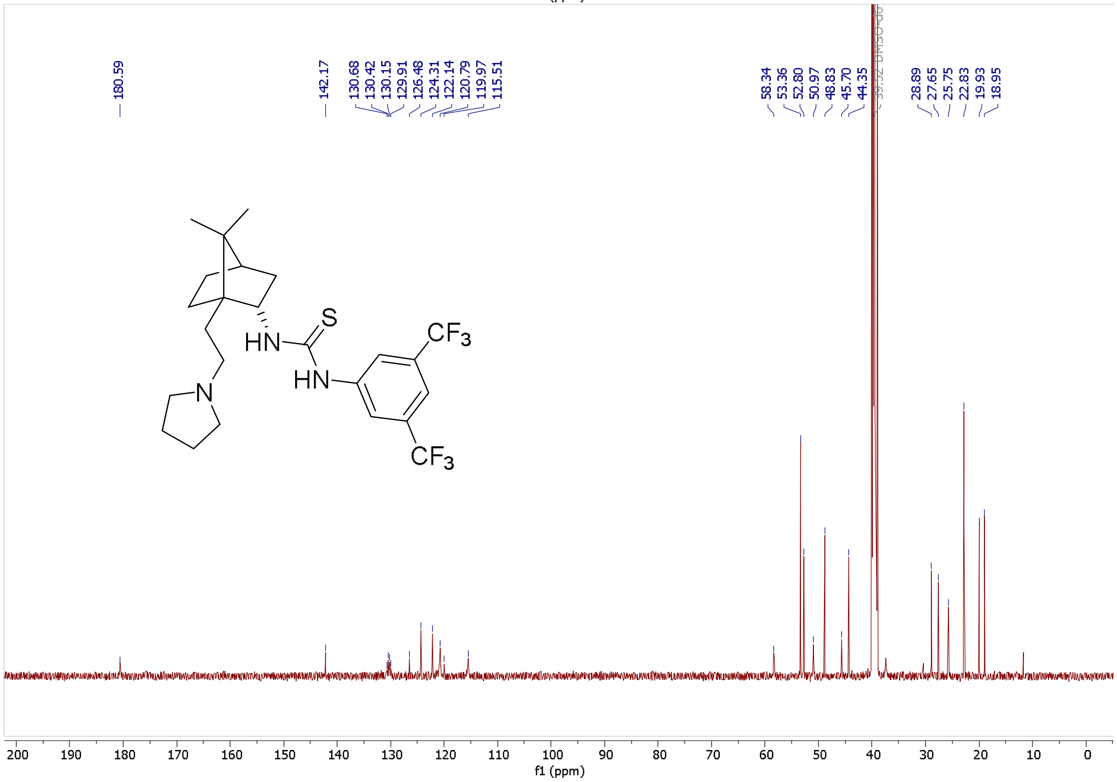

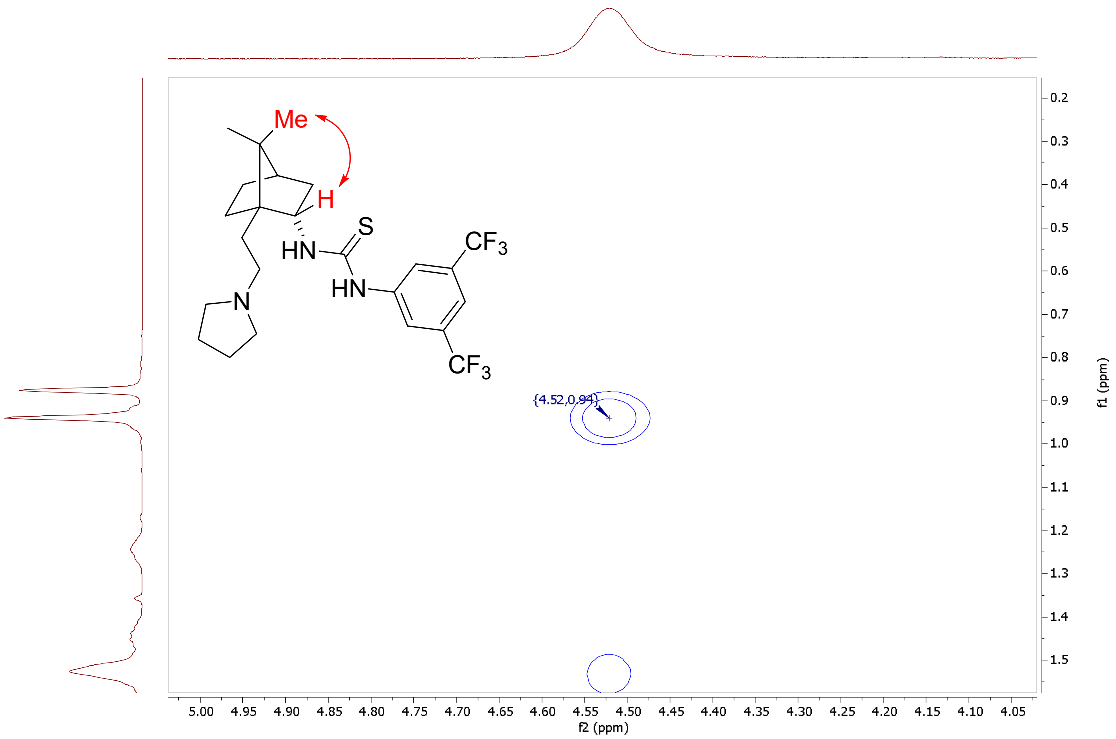
Me
H
S
HN
HN
N
CF_3
CF_3
{4.52,0.94}
f2 (ppm)
f1 (ppm)
5.00 4.95 4.90 4.85 4.80 4.75 4.70 4.65 4.60 4.55 4.50 4.45 4.40 4.35 4.30 4.25 4.20 4.15 4.10 4.05
0.2 0.3 0.4 0.5 0.6 0.7 0.8 0.9 1.0 1.1 1.2 1.3 1.4 1.5

2D NOESY

1-(3,5-Bis(trifluoromethyl)phenyl)-3-(2-((1*S*,2*R*,4*R*)-7,7-dimethyl-2-(pyrrolidin-1-yl)bicyclo[2.2.1]heptan-1-yl)ethyl)thiourea (**62**)

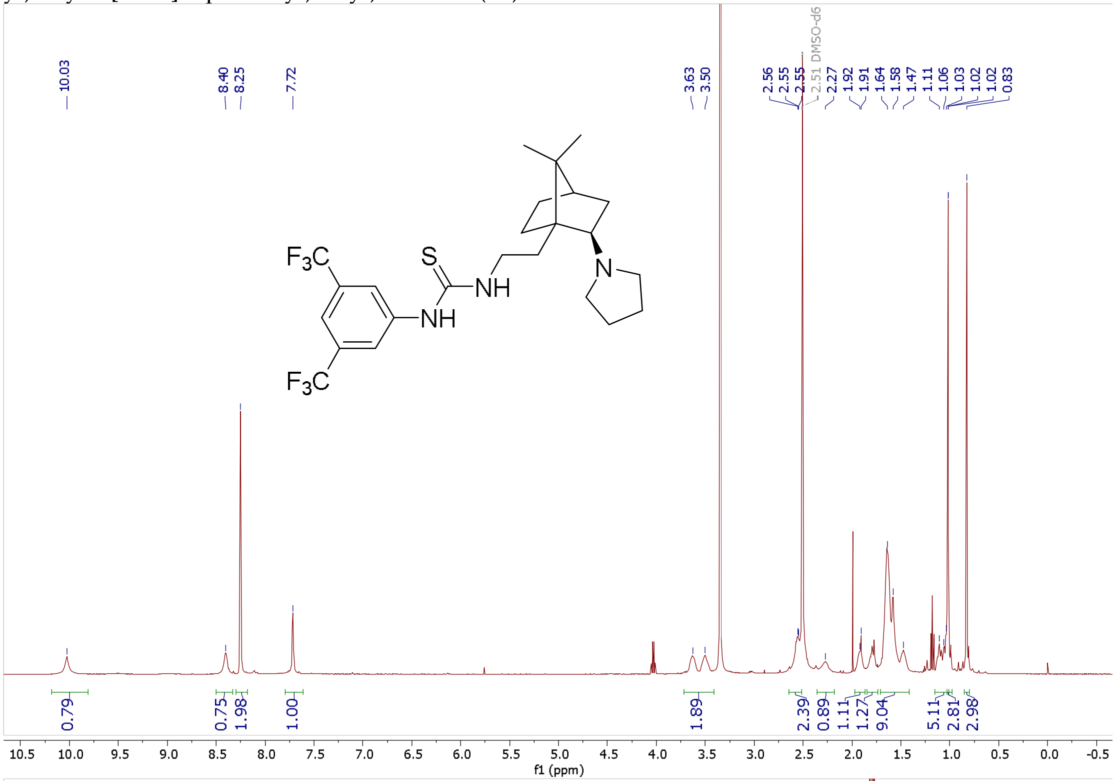

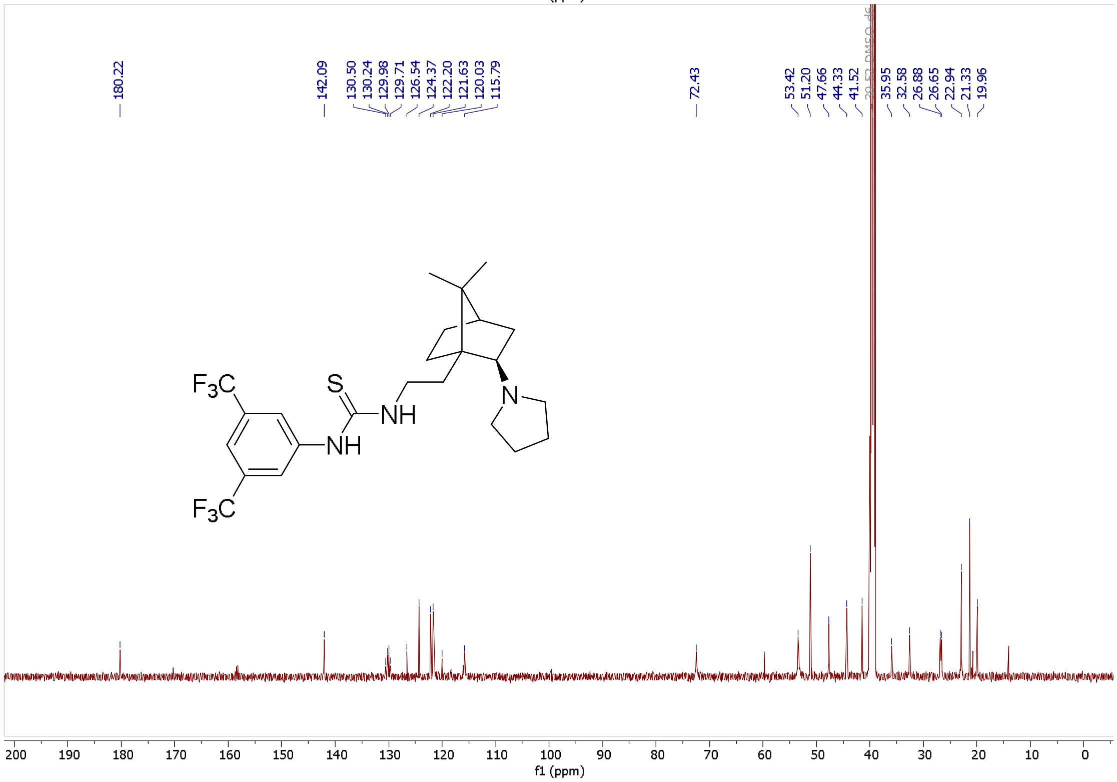

1-(3,5-Bis(trifluoromethyl)phenyl)-3-(2-((1*S*,2*S*,4*R*)-7,7-dimethyl-2-(pyrrolidin-1-yl)bicyclo[2.2.1]heptan-1-yl)ethyl)thiourea (**63**)

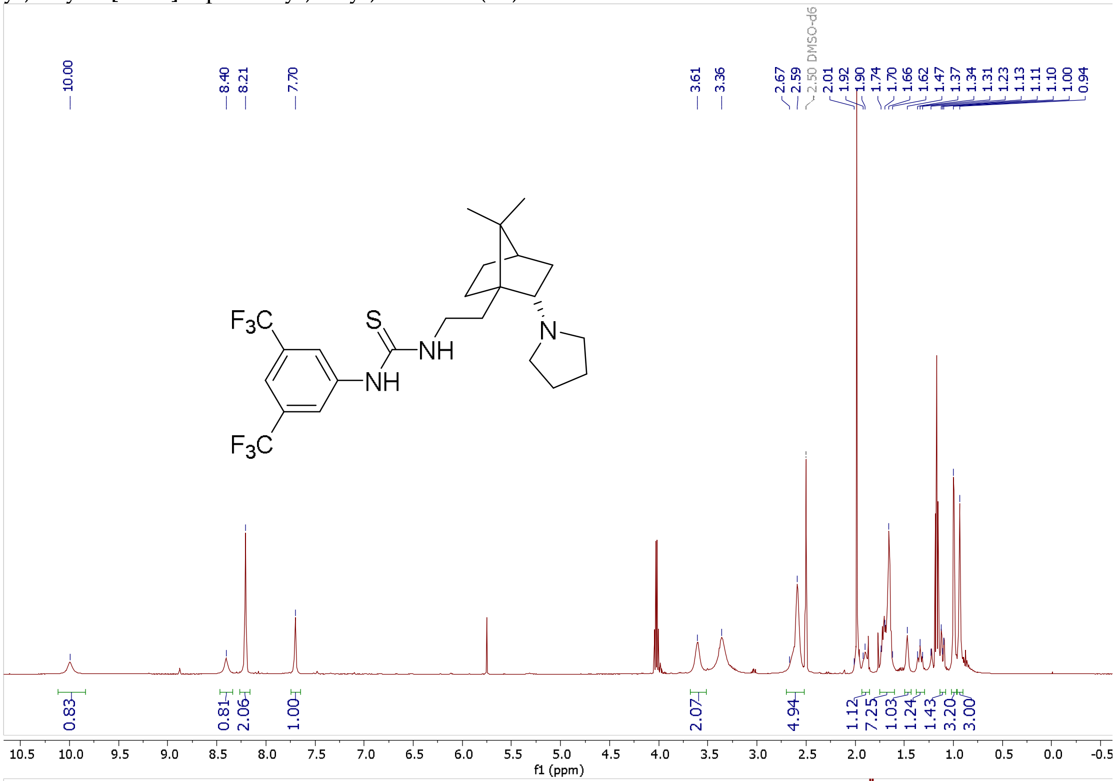

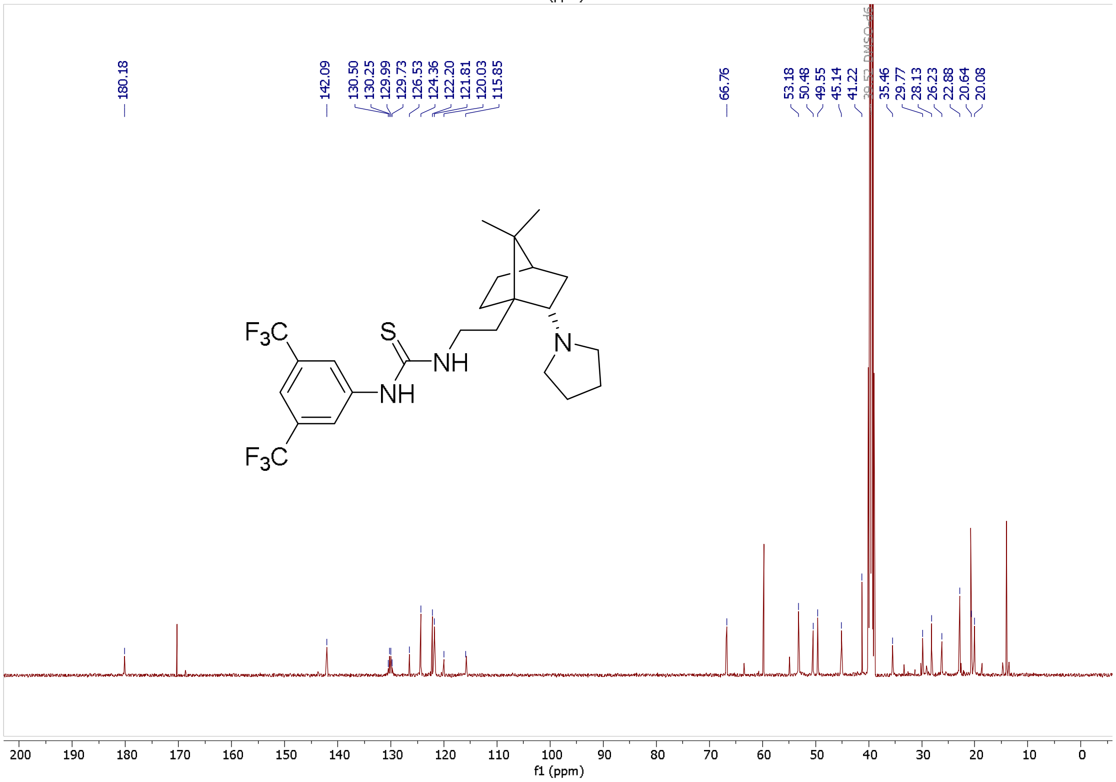

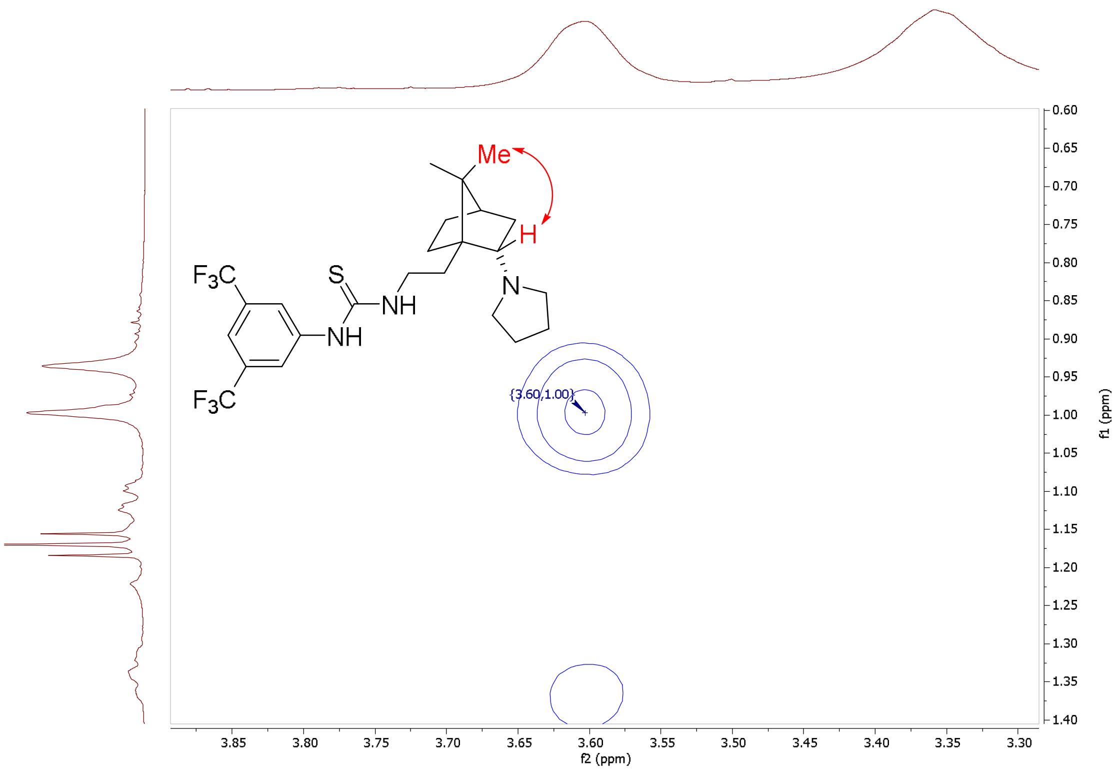
Me
H
F_3C
S
NH
NH
F_3C
N
{3.60,1.00}
0.60
0.65
0.70
0.75
0.80
0.85
0.90
0.95
1.00
1.05
1.10
1.15
1.20
1.25
1.30
1.35
1.40
f1 (ppm)
3.85
3.80
3.75
3.70
3.65
3.60
3.55
3.50
3.45
3.40
3.35
3.30
f2 (ppm)

2D NOESY

5. Selected HPLC data

Table 1, Entry 9

Product	Catalyst	t [days]<br>T [°C]	Conv. [%]	*Er*
9		3<br>25	>99	78.5:21.5<br>(*R*)

(Ar = 3,5-bis(trifluoromethyl)phenyl)
HPLC: Chiralpak AD-H, *n*-Hexane/*i*-PrOH = 90:10, flow rate 1.0 mL/min, λ = 210 nm.
Product: tR = 9.2 minutes (minor enantiomer); 13.3 minutes (major enantiomer).
Racemate:

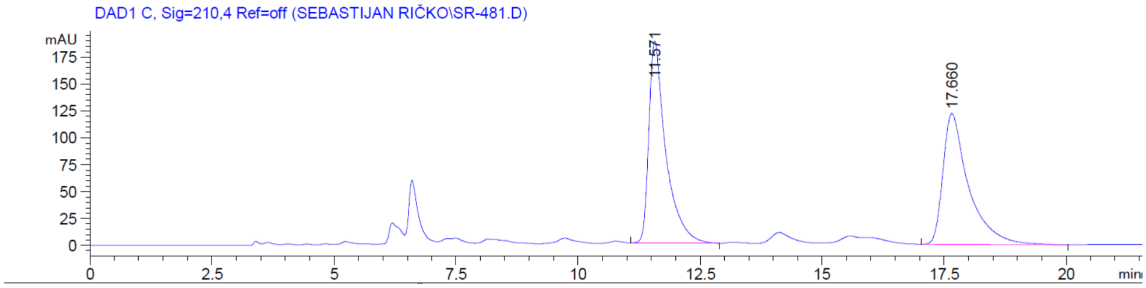

Organocatalyzed reaction:

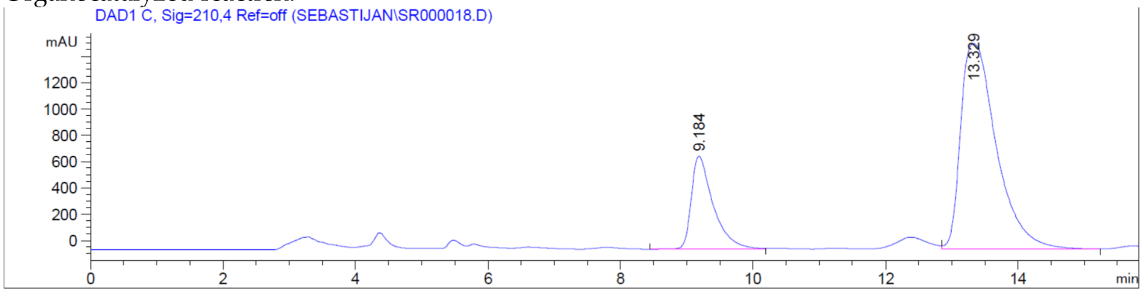

Signal 3: DAD1 C, Sig=210,4 Ref=off

Peak #	RetTime [min]	Type	Width [min]	Area [mAU*s]	Height [mAU]	Area %
1	9.184	BV	0.3354	1.62459e4	708.87122	21.7545
2	13.329	VB	0.5687	5.84326e4	1561.06042	78.2455
Totals :				7.46785e4	2269.93164	

Table 2, Entry 4

Product	Catalyst	t [days]<br>T [°C]	Conv. [%]	*Er*
4		3<br>-25	>99	91.5:8.5<br>(*S*)

(Ar = 3,5-bis(trifluoromethyl)phenyl)
HPLC: Chiralpak AD-H, n-Hexane/i-PrOH = 90:10, flow rate 1.0 mL/min, λ = 210 nm.
Product: tR = 8.5 minutes (major enantiomer); 11.0 minutes (minor enantiomer).
Racemate:

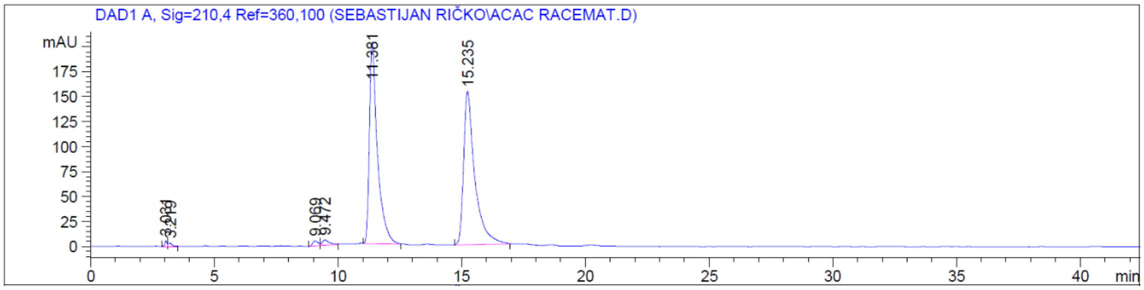

Organocatalyzed reaction:

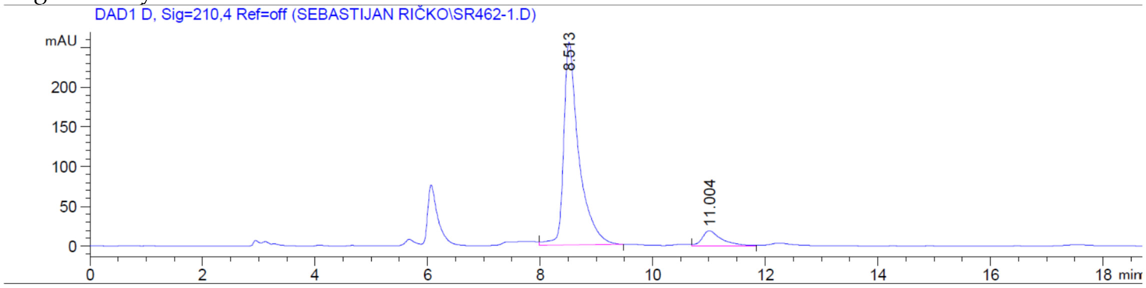

Signal 4: DAD1 D, Sig=210,4 Ref=off

Peak #	RetTime [min]	Type	Width [min]	Area [mAU*s]	Height [mAU]	Area %
1	8.513	VB	0.2658	4764.30811	255.18253	91.5797
2	11.004	VV	0.3346	438.05460	18.74840	8.4203
Totals :				5202.36270	273.93093	

Table 2, Entry 7

Product	Catalyst	t [days]<br>T [°C]	Conv. [%]	*Er*
7		3<br>25	>99	87:13<br>(*S*)

(Ar = 3,5-bis(trifluoromethyl)phenyl)
HPLC: Chiralcel OD-H, *n*-Hexane/*i*-PrOH = 80:20, flow rate 1.0 mL/min, λ = 210 nm.
Product: tR = 9.6 minutes (major enantiomer); 22.4 minutes (minor enantiomer).
Racemate:

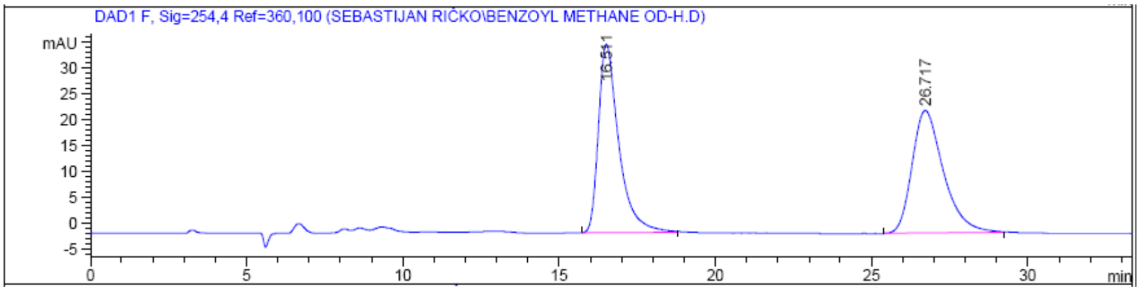

Organocatalyzed reaction:

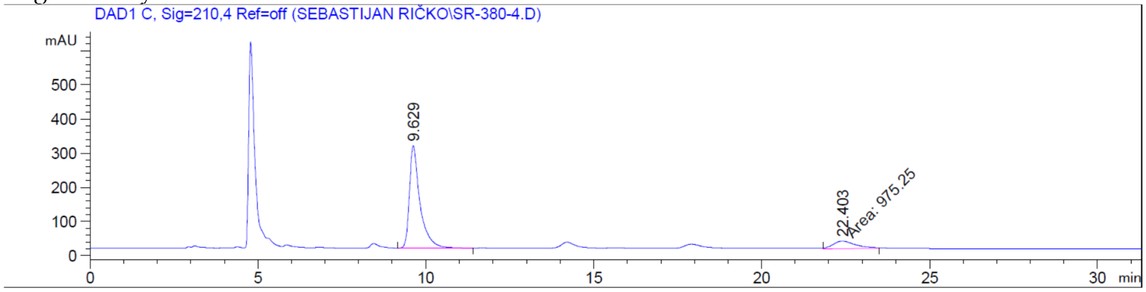

Signal 3: DAD1 C, Sig=210,4 Ref=off

Peak #	RetTime [min]	Type	Width [min]	Area [mAU*s]	Height [mAU]	Area %
1	9.629	VV	0.3181	6637.05762	300.05334	87.1885
2	22.403	MM	0.7579	975.25018	21.44743	12.8115

Totals : 7612.30780 321.50077

6. References

1. Ričko, S.; Svete, J.; Štefane, B.; Perdih, A.; Golobič, A.; Meden, A.; Grošelj, U. 1,3-Diamine-Derived Bifunctional Organocatalyst Prepared from Camphor. *Adv. Synth. Catal.* **2016**, *358*, 3786–3796.
2. Grošelj, U.; Ričko, S.; Svete, J.; Stanovnik, B. Synthesis of 2-(3-(3,5-Bis(trifluoromethyl)phenyl)thioureido)-3-((dimethylamino)methyl)camphor Organocatalysts. *Chirality* **2012**, *24*, 412–419.
3. Ričko, S.; Golobič, A.; Svete, J.; Stanovnik, B.; Grošelj, U. Synthesis of Novel Camphor-Derived Bifunctional Thiourea Organocatalysts. *Chirality* **2015**, *27*, 39–52.
4. Page, P. C. B.; Murrell, V. L.; Limousin, C.; Laffan, D. D. P.; Bethell, D.; Slawin, A. M. Z.; Smith, T. A. D. The first stable enantiomerically pure chiral N-H oxaziridines: synthesis and reactivity. *J. Org. Chem.* **2000**, *65*, 4204–4207.
5. Ju, Y.; Varma, R. S. An Efficient and Simple Aqueous N-Heterocyclization of Aniline Derivatives: Microwave-Assisted Synthesis of N-Aryl Azacycloalkanes. *Org. Lett.* **2005**, *7*, 2409–2411.
6. Spino, C.; Joly, M.-A.; Godbout, C.; Arbour, M. Ti-Catalyzed Reactions of Hindered Isocyanates with Alcohols. *J. Org. Chem.* **2005**, *70*, 6118–6121.
7. Polonski, T.; Dauter, Z. Chiroptical studies of bicyclic α-diketones. *J. Chem. Soc., Perkin Trans. 1* **1986**, 1781–1788.
8. Ishidate, M.; Kawada, A. Cleavage of the camphor ring. V. 7-Hydroxy-π-apocamphane and 1-hydroxy-10-apocamphor. *Pharm. Bull.* **1956**, *4*, 483–486.
9. Denmark, S. E.; Forbes, D. C.; Hays, D. S.; DePue, J. S.; Wilde, R. G. Catalytic Epoxidation of Alkenes with Oxone. *J. Org. Chem.* **1995**, *60*, 1391–1407.
10. Wu, Z.-L.; Wu, H.-L.; Wu, P.-Y.; Uang, B.-J. Asymmetric addition of diethylzinc to aldehydes catalyzed by a camphor-derived β-amino alcohol. *Tetrahedron: Asymmetry* **2009**, *20*, 1556–1560.
11. Garcia Martínez, A.; Teso Vilar, E.; Garcia Fraile, A.; de la Moya Cerero, S.; Diaz Oliva, C.; Subramanian, L. R.; Maichle, C. Synthesis of homochiral cyclopentane derivatives by Beckmann fragmentation of 1-substituted 2-norbornanones. *Tetrahedron: Asymmetry* **1994**, *5*, 949–954.
12. Garcia Martínez, A.; Teso Vilar, E.; Garcia Fraile, A.; de la Moya Cerero, S.; Ruiz, P. M.; Subramanian, L. R. Synthesis of homochiral 3-substituted cyclopentanones from 2-norbornanones. *Tetrahedron: Asymmetry* **1996**, *7*, 2177–2180.
13. García Martinez, A.; Teso Vilar, E.; García Fraile, A.; de la Moya Cerero, S.; Díaz Morillo, C. Enantiospecific access to 10-N-substituted camphors. *Tetrahedron* **2005**, *61*, 599–601.
14. Schenone, P.; Tasca, A.; Bignardi, G.; Mosti, L. *N,N*-disubstituted 10-amino-2-*exo*-bornanols and related esters. *Eur. J. Med. Chem. - Chim. Ther.* **1975**, *10*, 412–417.
15. Helliwell, M.; Thomas, E. J.; Townsend, L. A. Synthesis of chiral organotin reagents: synthesis of enantiomerically enriched bicyclo[2.2.1]hept-2-yl tin hydrides from camphor. X-Ray crystal structures of (dimethyl)[(1*R*,2*S*,4*R*)-1,7,7-trimethylbicyclo[2.2.1]hept-2-yl]tin chloride and methyl(phenyl)bis[(1*R*,2*S*,4*R*)-1,7,7-trimethylbicyclo[2.2.1]hept-2-yl]stannane. *J. Chem. Soc., Perkin Trans. 1* **2002**, 1286–1296.
16. Loudon, J. D. Mercury derivatives of camphor. I. The constitution of Reychler's acid. *J. Chem. Soc.* **1933**, 823–825.
17. Agilent Technologies. CrysAlis PRO. Version 1.171.35.11; Agilent Technologies: Yarnton, Oxfordshire, England, 2011.
18. Altomare, A.; Burla, M. C.; Camalli, M.; Cascarano, G. L.; Giacovazzo, C.; Guagliardi, A.; Moliterni, A. G. G.; Polidori, G.; Spagna, R. *SIR97*: a new tool for crystal structure determination and refinement. *J. Appl. Crystallogr.* **1999**, *32*, 115–119.
19. Sheldrick, G. M. A short history of SHELX. *Acta Crystallogr., Sect. A* **2008**, *A64*, 112–122.
20. Farrugia, L. J. ORTEP-3 for windows - a version of ORTEP-III with a graphical user interface (GUI) *J. Appl. Crystallogr.* **1997**, *30*, 565–565.
